# Supplementary material for: The phased pan-genome of tetraploid European potato
Source: Nature. 2025 Apr 16;642(8067):389–97. doi: 10.1038/s41586-025-08843-0 (PMC12158759; doi:10.1038/s41586-025-08843-0)
Supplement: Supplementary file 1 — This file contains a Supplementary Notes section, which explains the initial genome assembly and analysis of genomes, a Supplementary methods section, which explains methods for cultivar selection, genome sequencing, assembly and annotation, genetic variation analysis, pan-genome construction, and construction and application of haplotype graph, and 47 (sets of) Supplementary figures. Note, all supplementary figures are provided within a zip file. [file 41586_2025_8843_MOESM1_ESM.docx]

Supplementary Information

**The phased pan-genome of tetraploid European potato**

Sun and Tusso *et al.*, 2024

# **Supplementary Notes**

***Initial assembly and analysis of genomes.*** While the initial assemblies feature high sequence contiguity, the haplotype-specific sequences are not phased to individual haplotypes. In addition, large differences of 0.5 to 1.0 Gb between the assembly sizes and *k*-mer estimates of genome size indicated that there were substantial amounts of collapsed genomic regions within the *de novo* assemblies (Extended Fig. 1a). To identify these regions, we aligned the whole-genome short reads to the initial contigs, and using read coverage, categorized them into haplotigs, diplotigs, triplotigs, tetraplotigs representing (collapsed) contigs from one, two, three and four haplotypes, as done previously^1^. The percentages of haplotigs ranged from 68.9% to 84.4% (average: 77.7%), diplotigs from 12.3% to 22.5% (average: 17.5%), triplotigs from 1.7% to 6.6% (average: 3.5%), and tetraplotigs from 0.1% to 2.9% (average: 1.1%), while contigs that represented collapsed repeats were negligibly (<0.4%) (Supplementary Fig. 30; Supplementary Table 2). In contrast to 'Otava', a genome that was assembled earlier^1^, the selected founder varieties exhibited significantly more haplotigs, while diplotigs to tetraplotigs were noticeably less implying that the founder lines were less inbred that the recently developed breeding line ‘Otava’.

To phase the haplotypes, we developed a novel Hi-C based pipeline (Fig. 1c; Supplementary Methods). We tested the pipeline with genome sequencing data of ’Otava’ and found that the haplotyping accuracy was 98.8% (Supplementary Fig. 31), comparable to 99.6% which was achieved using over 700 pollen genomes^1^.

Having evaluated the performance of the pipeline, we applied it to phase the haplotypes of the ten founder genomes, and performed *de novo* assembly for each haplotype-specific chromosome. In general, more than 99.0% of the initial contigs of each variety were assigned to chromosomes (Supplementary Table 2; Supplementary Methods). Interestingly, we found that the total sizes of the contigs of many cultivars grouped to chromosomes 3, 6 and 10 were smaller (than references), indicating a lower level of sequence divergence or a higher level of haplotype sharing in these chromosomes (Extended Fig. 1b). In the following analysis, unless specifically clarified, all related analysis were performed with the first version of the genome assembly and annotation as given here: *10.5281/zenodo.14053896*.

**Supplementary Methods**

##

## **Selection of ten potato varieties for genome assembly**

## We first analyzed all 9,599 cultivars with pedigree records in the Wageningen University Potato Pedigree Database. The cultivars that were found in the pedigrees of at least nine other varieties and that were introduced before 1960, were selected (Supplementary Fig. 32; Supplementary Table 1). In addition, old or previously widely grown varieties like ‘Yam’ (introduced before 1787), ‘Lumper’ (introduced before 1810), ‘Edzell Blue’ (introduced before 1890) and ‘Papa Bonita’ (NA) were also considered. Of the 164 cultivars in the database that fitted our criteria, we found 19 in the Gross Lüsewitz Potato Collections (GLKS) of the IPK Gene Bank, where such material is being preserved long-term (Supplementary Table 2).

To figure out the relatedness of the 19 varieties, we sequenced the genomes with short reads (Supplementary Table 2), and called single-nucleotide polymorphisms (SNPs) by aligning the reads to the reference genome *DM1–3 516 R44 v6.1*^2^ (or just *DM*) using *bowtie2* (version 2.2.8)^3^ and *shore*^4^, respectively for each cultivar. We further selected SNPs only within genic regions for each cultivar, where the alternative base score was 40 (maximum) and the alternative allele frequency was around 0.25 (or [0.18, 0.32]), 0.50 (or [0.43, 0.57]) and 0.75 (or [0.68, 0.82]), as calculated by *shore.* Then we merged 3,166,093 selected SNP positions to create a set of markers, on which each cultivar was genotyped. This led to a genotype matrix for the 19 cultivars with which we performed PCA analyses using *prcomp* function with default settings in *R*. *K*-means clustering with the first two components led to two clearly-separated groups separating *S. tuberosum* ssp. *andigena* und ssp. *tuberosum*. Finally, we selected the ten cultivars (introduced from ~1810 to 1932).

## **Sequencing of ‘Kenva’**

Genomic DNA from the cultivar ‘Kenva’ (KKS 11553 Gatersleben) was extracted using the NucleoSpin Plant II Mini kit for DNA from plants (Item number: 740770.50), starting from Step 5 of the NucleoSpin® Plant II protocols. We used 250 mg of fresh young leaf material, which was harvested, flash-frozen in liquid nitrogen, and homogenized in liquid nitrogen using a mortar. The protocol's "Cell lysis using Buffer PL2" step was followed. The genomic DNA concentration was measured at 104 ng/µl with the Qubit™ (Invitrogen), resulting in a total DNA yield of 2,080 ng. The sample was sent on dry ice to BGI for short-read sequencing using the DNBseq™ platform.

## **Genome size estimation**

For each of the ten cultivars, we used between 390 to 568 Gb of short read data for *k*-mer counting (*k*=21) with *Jellyfish*^6^ (version 2.2.10). The *k*-mer histogram was provided to *findGSE*^7^ (version 1.0) to estimate the haploid/tetraploid genome sizes using the “heterozygous mode” (Supplementary Fig. 1).

## **Initial tetraploid genome assembly and purging**

First, each of the PacBio HiFi read sets were trimmed with *longQC*^9^ with default settings. Initial genome assemblies were generated with *hifiasm*^8^ (version 0.7) with default settings. The unitigs (i.e., locally haplotype-resolved contigs) were selected for further processing. Then the whole-genome short reads were aligned to the unitigs using *bowtie2* (version 2.2.8)^3^. A purging process based on the amounts of aligned reads was repeated for five rounds to generate a new assembly for subsequent analysis (Supplementary Fig. 30).

## **Classification of initial contigs based on sequencing coverage**

For each cultivar, whole-genome short reads were aligned to the assemblies using *bowtie2* (version 2.2.8)^3^ and *minimap2* (version 2.20-r1061)^10^. Duplicated short reads were removed using *picard* *MarkDuplicates* ([*http://broadinstitute.github.io/picard/*](http://broadinstitute.github.io/picard/)). The depth along each contig was calculated with *samtools depth* function (version 1.9)^11^. Each contig was split into 10 kb windows and the average sequencing depth per base was calculated within each window. According to the average genome-wide depth *d* per haplotype, windows with depths [0, 1.5*d*], [1.5*d*+1, 2.5*d*], [2.5*d*+1, 3.5*d*], [3.5*d*,4.5*d*] and [4.5*d*+1, INF] were determined as haplotig, diplotig, triplotig, tetraplotig and replotig (Supplementary Fig. 30).

## **Hi-C based haplotype phasing**

Non-haplotigs can introduce false connections between haplotypes when separating the contigs to haplotypes by Hi-C data (Supplementary Fig. 33). To overcome this issue, we developed a new method for haplotype phasing (main text Fig. 1c). First, we aligned the initial contig-level assembly to the potato reference genome^2^ to group contigs into 12 major clusters using *minimap2* (version 2.20-r1061)^10^, each representing a chromosome with a mixture of four haplotypes. Second, for each cluster, we grouped the haplotigs into four sub-clusters using Hi-C contact, and subsequently assigned diplotigs, triplotigs and tetraplotigs to two, three or four of the sub-clusters formed by haplotigs using the Hi-C contact signals. After these steps, each sub-cluster represents a haplotype-resolved chromosome. Third, we aligned the HiFi reads to the clustered contigs, *i.e.*, assigning long reads to haplotypes. Fourth, we re-assembled each haplotype-specific chromosome using the haplotype-specific HiFi reads and scaffolded the respective contigs to chromosome-scale using Hi-C (method details are given below), independently. Fifth, using Hi-C contact maps including all four haplotypes, we search for mis-phased regions, and corrected haplotype switch errors (see below).

## **Hi-C based scaffolding and haplotype switch error correction**

All Hi-C reads were mapped to haplotype-specific chromosomes. For each cultivar, all Hi-C reads were aligned to the contig assemblies using *bowtie2* (version 2.2.8)^3^. If a read pair could be aligned to coverage-categorized contigs (diplotigs, triplotigs or tetraplotigs) from the same group, then it was assigned to that group, otherwise the read pair was removed from the analysis. If a read pair could be aligned to coverage-categorized contigs of multiple groups, it was randomly assigned to one of the groups.

We indexed the contig-level assembly of each haplotype with *bwa index* (with -*a bwtsw*) (version 0.7.15-r1140)^12^ and *samtools faidx*. Next, we aligned the haplotype-specific Hi-C read pairs to the contig assemblies using *bwa aln* and *bwa sampe*. We then converted the aligned read pairs into BAM files using *samtools view* with options of “*-b -F12”*. The BAM files were filtered with *filterBAM_forHiC.pl* (from *ALLHiC*^13^ package, version 0.9.13) to remove non-uniquely mapped reads. Then, for each haplotype, *ALLHiC_partition* was run with “*-e GATC -k 1 -m 25”*, *allhic extract* was run with “--*RE GATC”*, *allhic optimize* and *ALLHiC_build* were run with default settings. The chromosome contact maps were visualized with *ALLHiC_plot* at 1 Mb resolution, with which mis-placed or falsely orientated contigs were visually identified. In addition, by aligning the initial scaffolds to the *DM* reference genome, we manually corrected the scaffolding order with the help of the Hi-C contact maps using *Juicer* (version 1.6) and *Juicebox*^14^ (version 2.13.07) (Supplementary Figs. 34-44).

After individual chromosome-level scaffolds had been generated, Hi-C contact maps of the four haplotypes of each chromosome were built with *ALLHiC_plot* at 1 Mb resolution. Each Hi-C contact map was manually examined, and if a genomic region in one haplotype showed higher contact with another haplotype, the genomic regions were swapped between the haplotypes to correct haplotype switch errors. An example for correcting haplotype switch errors is shown in Supplementary Fig. 45.

## **Completeness and base quality assessment of the genome assemblies**

Short reads of each cultivar were used to create a *k*-mer database (*short_read.meryl*) with *meryl* (version 1.3)^15^ with options “*k=21 count threads=4 memory=8g*”. For each cultivar, the complete assembly was compared with its *k*-mer database to investigate the completeness and the base accuracy (QV), using *Merqury* (version 1.3)^15^ using “*merqury.sh short_read.meryl assembly.fa full*”.

## **Gene annotation and BUSCO analysis**

To capture all of the gene models that are present in each haplotype, we initially annotated genes in the unphased contig-level assemblies of each variety and subsequently transferred the gene annotations to the respective phased individual haplotype assemblies. Prior to gene annotation, we generated *de novo* repeat libraries for the contig assemblies by running *EDTA* (v2.1.0)^16^, providing a FASTA file containing coding sequences of the DM reference genome annotation (high confidence gene models only) to the “*--cds*” parameter as input to prevent genes from being annotated as repeats. We ran *EDTA* with default parameters except for “*--overwrite 1 --anno 1 --threads 25*”. The resulting repeat libraries were subsequently used to soft-mask repeats in each contig assembly using *Repeatmasker* (version 4.1.2-p1)^17^ with parameters “*-xsmall -gff -cutoff 200*”.

We annotated genes in the soft-masked contig assemblies using *BRAKER* (v2.1.6)^18-20^. We first ran the *BRAKER1* workflow^21-22^, which uses RNA-seq alignments as input. RNA-seq alignments were generated by running *HISAT2*^23^ (version 2.2.1) using 20 RNA-seq datasets of the C88 potato^24^ variety as input. The resulting SAM file was converted into an indexed, coordinate-sorted BAM file using *samtools* (version 1.11) and provided to *BRAKER* to generate a GFF file containing gene models predicted using RNA-seq evidence only. We subsequently ran the *BRAKER2*^25-29^ workflow, which uses proteins as input. The input protein sequences consisted of translated gene models of the *DM* (high confidence models only) and tomato ITAG4.0^30^ annotations, and the orthoDBv10^31^ plant protein set. The orthoDBv10 plant protein set was excluded for the ‘Edzell Blue’ contig assembly as the *BRAKER2* workflow would not finish otherwise. The output GFF files of the *BRAKER1* and *BRAKER2* workflow were combined using *TSEBRA* (v1.0.3)^32^, retaining transcript models of which the introns, start site, and stop site were supported by at least one RNA-seq or protein hint. The output files of *TSEBRA* represent the final gene annotations for each contig assembly.

Gene annotations of each contig assembly were transferred to their respective assemblies using *liftoff* (v1.6.2)^33^, only keeping transcript models if they aligned over their complete length (parameter “*-a 1*”) without mismatches (parameter “*-s 1*”). These strict alignment parameters prevent mis-mapping of transcript models to homologs present on different haplotypes. We removed transcript models with internal stop codons, invalid start codons, and/or invalid stop codons using *AGAT* (v1.0.0)^34^. We aligned protein sequences derived from *DM* (high confidence models only) and tomato ITAG4.0 annotations to the haplotype-resolved assemblies using *miniprot* (v0.11)^35^, and used *bedtools* intersect (v2.29.1)^36^ to identify transcript models that fully overlap with at least one protein alignment. Transcript models that fulfilled this criterion were classified as high-confidence models, otherwise they were classified as low-confidence models. We assessed the completeness of the gene annotations using *BUSCO* (v5.2.2)^37^ with the parameters “*-l solanales_odb10 –augustus*”. Finally, we identified gaps in each haplotype assembly using *seqtk cutN* with parameters "*-gp10000000 -n1*" (*https://github.com/lh3/seqtk*), used *bedtools* intersect to identify gene models overlapping these gaps, and removed such gene models from the annotations using *AGAT*.

To understand the origin of the differences in gene number between the individual haplotype annotations, we used *liftoff* with default parameters to identify putatively missing gene models. In brief, we mapped the gene models of the genome with the largest number of genes (‘Flava’) to all haplotype genomes. Large differences in gene numbers between the haplotype annotations remained (minimum of 38,703 genes, maximum of 46,312 genes, excluding the ‘Flava’ haplotype annotations), implying that they are not a result of gene annotation artefacts.

Note, two versions of genome annotation are available at 10.5281/zenodo.10617012 (v2) and 10.5281/zenodo.14053896 (v2.1), where genome annotation v2.1 was performed for genome assembly v2.1 which was improved by a few haplotyping corrections over v2.

## **Detection of centromeric repeat**

*TRASH* (version 1.2)^38^ was used to detect tandem repeats for each haplotype-specific sequence of the ten varieties, which provided candidates of centromeric repeats. The parameters of *TRASH* were set as “*--k15 --w 5000 --m 4000*” to search for tandem repeats up to 4 kb. Distribution of tandem repeats are provided in Supplementary Figs. 3-14.

## **Detection of pericentromeric regions**

Each haplotype-specific sequence of all eleven assemblies was aligned to the dAg1_v1.0^39^ reference genome assembly using *nucmer3* (version 3.1)^40^. Then using *SyRI* (version 1.6)^41^, the syntenic blocks were detected between each of the sequences and the reference sequence. Regions of the sequence with syntenic relationships to the pericentromeric region of the reference sequence were determined as pericentromeric. Distribution of tandem repeats were provided in Supplementary Figs. 3-14.

## **Detection of rDNA, tDNA**

*tRNAscan-SE* (version 2.0.5)^42^, *barrnap* (version 0.9, *https://github.com/tseemann/barrnap*), and *infernal* (version 1.1.4)^43^ were used to detect rDNA and tDNA, with Rfam as the database (*ftp://*[*ftp.ebi.ac.uk/pub/databases/Rfam/14.3/Rfam.cm.gz*](http://ftp.ebi.ac.uk/pub/databases/Rfam/14.3/Rfam.cm.gz)). The script *infernal-tblout2gff.pl* (version 1.1.4) was used to convert the result of *infernal* into GFF format. Distribution of rDNA and tDNA were provided in Supplementary Figs. 3-14.

## **Analysis of local diversity and linkage disequilibrium**

The haplotype-specific sequences of each cultivar were aligned to *DM* using *nucmer3* (version 3.1)^40^ with options *“--maxmatch -c 100 -l 80 -b 500”*. The result was processed using *delta-filter* with options *“-m -i 85 -l 100”*, and further with *show-coords* with option *“-THrd”*. The coordinate file was used as input for *SyRI* (version 1.6)^41^ to call SNPs, SVs, and syntenic regions. The distribution of SV along the genome was determined by identifying syntenic regions in all pairwise comparisons to *DM* using *Msyd* (*https://github.com/schneebergerlab/msyd*). Genetic diversity (π)^44^ of SVs was calculated in 1 Mb windows. The contribution of SVs within a window was weighted, taking into account the proportion of the window covered by each of the SVs.

Genetic variants obtained from pairwise comparisons between each haplotype and *DM* were merged into a single genotype table. For this, only genetic variants annotated as deletion, insertion, or SNPs were included and merged when start position, end position and the reference allele matched between samples. Multiple alleles per variant were considered. For every genomic region in the reference genome, we computed the number of haplotypes that successfully aligned, which was used as sampling size. Then we calculated population genetic parameters in 10 kb windows including minor allele frequency, pairwise nucleotide diversity (π)^44^, and Watterson theta (*θ_w_*)^45^. Subsequently, the number of segregating sites per 10 kb window and pairwise haplotype comparison was used to cluster identical haplotypes. Two haplotypes were clustered when the number of variants between them was below 10 (less than one SNP per 1 kb). This threshold was defined by identifying the inflection point in the genome-wide distribution, where the initial peak - corresponding to comparisons of nearly identical haplotypes - declines and the second peak, representing comparisons of more divergent haplotypes, begins to rise (Supplementary Fig. 46). Clustering of haplotypes per window was used to plot the number of unique haplotypes, major allele frequency and number samples sharing haplotypes along the genome.

We calculated linkage disequilibrium (LD) between all pairwise genetic variants of all chromosomes (Supplementary Fig. 23). For each pair of variants, we calculated standardized LD as the squared *Pearson*'s correlation coefficient (*R^2^*)^46-47^. Differences in allele frequencies are considered in this measurement. LD was calculated using the frequency of the major allele for each variant site (*p*) versus other alleles (*q*). The expected value of *R^2^* (*E(R^2^)*) can be approximated by Hill & Weir (1988)^48^:

$E{(R}^{2})=\left( \frac{10+C}{(2+C)*(11+C)} \right)*\left( 1+\frac{(3+C)*(12+12C+C^{2})}{n*(2+C)*(11+C)} \right)$,

where *C* is the product between the genetic distance (bp) and the population recombination rate (*ρ*) for a given sampling of size *n*. Three million LD measurements per chromosome were randomly subsampled to fit a nonlinear model and to obtain least squares estimates of *ρ*. Decay of LD with physical distance can be described with this model^49^ (predicted values in Supplementary table 6 and LD decay in Supplementary Fig. 22). Additionally, mean LD values within 500 kb windows were calculated to plot variation of local LD along the genome.

## **Detection of introgression from wild potato species**

HiFi reads of 20 wild potato species^50^ were downloaded from NCBI SRA archive. Then for each species, the reads were aligned to *DM* and to each individual cultivar haplotype using *minimap2* (version 2.20-r1061)^10^. Variant calling was performed in the alignments to the *DM* reference genome using *DeepVariant* (v1.4.0)^51^. The generated GVCF files were then merged using *GLnexus* (v1.2.7)^52^ to create a unified variant dataset including both cultivar and wild samples. Low-quality variants, specifically those with a quality score less than or equal to 30, were filtered out using *bcftools* (v1.10.2)^11,53^. Then, with the alignments to the cultivar genmomes, *mosdepth* (version 0.3.1)^54^ was used to calculate the mean read depth in 100 kb windows along the chromosomes. In the heatmap visualisations of read depths across 20 species for each haplotype, depths above 50x were all set as 50x (Supplementary Figs. 3-14).

General relationship between samples was analysed using a phylogenetic approach. This analysis was performed first along the genome in 100 kb windows. VCF files were split into windows using *jvarkit* (v2024.04.20) (*https://github.com/lindenb/jvarkit*). Maximum likelihood phylogenetic trees per window were constructed using *IQ-TREE* (v2.1.2)^55^ with the GTR model and 1000 bootstrap replicates. Consensus trees were built including all window trees per chromosome and for the whole genome using *ASTRAL* (v5.7.8)^56^ (main text Fig. 3a; Supplementary Fig. 24).

Admixture analyses were performed to identify potential genetic admixture between wild and cultivar samples, providing insights into the genetic structure and history of these populations. For this analyses, genetic variants were filtered to only include SNPs using *VCFtools* (0.1.16)^53^, and pruned by LD using *PLINK* (1.90b6.18)^57^, using a window size of 50 SNPs, a step size of 25 SNPs, and an LD threshold of 0.3. Admixture analysis was conducted independently for each chromosome using *ADMIXTURE* (1.3.0)^58^ (Fig. 3b; Supplementary Fig. 25). To identify the optimal number of ancestral populations (*K*), the analysis was performed for *K* values ranging from 2 to 10, and 5 replicate runs for each case. For each *K* value, only the replicate with the highest likelihood was included. *ADMIXTURE*'s cross-validation procedure was employed to determine the *K* value with the lowest cross-validation error.

To identify introgressions in the cultivated samples, we conducted D-statistic (ABBA-BABA^59^) and f4 statistic tests^60-61^. We explicitly tested for introgression between individual cultivar haplotypes and species belonging to the clade C4S. This analysis was performed both per chromosome to assess statistical significance and in sliding windows to assess the distribution of D-statistics along the genome. We selected two species from the C4S clade and one cultivar to test for introgression between C4S and the cultivars. The analyses were performed independently for each cultivar haplotype, and wild species within clades were permutated to test for introgression with different combinations of species (Fig. 3d). The package *Dsuite* (version 0.5)^62^ was used to calculate D-statistics, f4-ratio, and associated p-values at the chromosome level (Supplementary Fig. 28). Significance was tested using a jack knife test using block size of 100 kb. Other block sizes up to 4 Mb showed similar results. For each chromosome, we calculated the number of tests supporting introgression from a particular ancestral clade. To identify candidate regions of introgression along the genome, we applied the same ABBA-BABA and f4 tests in sliding windows across each chromosome. Windows of 200 kb were used with a step size of 50 kb.

## **Sequence-level pan-genome construction**

The pan-genome was constructed with 40 haplotypes from the ten selected cultivars. The graph was constructed using *minigraph-0.20-r559*^63^ iteratively, with parameters “*-cxggs -t 20*”. Each haplotype was added to the initial graph one at a time. This process was iterated 11 times, each time with a different randomized order of haplotypes. The pangenome growth curve was derived from the average sizes per haplotype addition along those 11 permutations. The curve follows a saturation growth model with equation *y=a_1_*x / (x+a_2_) + a_3_*. To fit the model, *i.e.* to optimize the values of *a_1_*, *a_2_* and *a_3_*, the distances between each point were measured based on a root-square-mean function and optimized using the BFGS method in *R 4.3.0*^64^. The model was then used to estimate the number of tetraploid genomes required to capture 85%, 95% and 99% of the genomic diversity in the potato species. This was done with the second version of the assembly given here: 10.5281/zenodo.14053896.

## **Gene-level pan-genome construction**

All protein-coding genes annotated in the 40 haploid genomes were clustered using *OrthoFinder* (version 2.5.5)^65^, *diamond* (version 2.0.13)^66^, and *Blast* (version 2.12.0+)^67^. *OrthoFinder* was run with parameters *“-op -t 48 -a 48 -S diamond_ultra_sens -f”* in the first phase, then *diamond blastp* was ran in parallel with parameters of *“-d -q -o --ultra-sensitive -p 1 --quiet -e 0.001 --compress 1”*, again followed by *orthofinder* using *“-t 48 -a 48 -S diamond_ultra_sens -b”* parameters. Based on the clustering, orthologous groups or gene families shared by all 40 haplotypes were defined as core genes, gene families shared by 37-39 haplotypes were defined as softcore genes, gene families shared by 2-36 haplotypes were defined as dispensable genes, and gene families present in only one haplotype were defined as private genes. Gene families defined by *OrthoFinder* were used to build up the pan-genome and core-genome, where up to 2,000 random samplings from the 40 haploid genomes were performed for each sample size within [2, 40].

## **Simulation of tetraploid potato genomes**

Tetraploid genomes were simulated by randomly sampling 4 of the 40 haplotypes. This was repeated for 5,000 times, leading to 5,000 simulated tetraploid genomes. The number of gene families in the four selected haplotypes was calculated for each simulated tetraploid genome like for the actual cultivars.

## **Construction of a haplotype-graph for haplotype-phasing short-read data**

To create a haplotype-graph from the pan-genome, we aligned the 40 haplotypes to *DM* using *minimap2*^10^ and identified genomic variants using *SyRI* (version 1.6)^41^. Single-nucleotide polymorphisms in syntenic regions were selected from the VCF files using *vcftools* and the individual VCF files were merged using *bcftools*^11,53^. The reference genome coordinates were binned into non-overlapping 100 kb windows. Haplotypes in each window were clustered if the edit distance between their SNV profiles was less than 10% of the number of SNVs. Each cluster of haplotypes constituted a node at a window. Nodes in adjacent windows were connected by edges if they were linked in any of the contributing haplotypes. Taking inspiration from *Pangenie*^68^, for each node, *marker k-mers* (with *k*=51) were identified as *k*-mers in the syntenic regions, compared to *DM*, in the contributing haplotypes and unique to the focal node. As each of the marker *k*-mers was specific to a single node, they uniquely genotyped individual nodes. This was done with the second version of the assembly given here: 10.5281/zenodo.14053896.

## **Identifying copy-number of nodes in the haplotype-graph from short-read data**

In a given WGS sample, we counted all 51-mers that were unique to a node in the haplotype-graph using *Jellyfish* (version v2.2.6)^6^ (command: count -m 51 -s 240M -C --if <list of 51-mers unique to nodes in the graph>). We then fit a model to the histogram of *k*-mer counts, to approximate the distributions of 0-copy, 1-copy, 2-copy, 3-copy and 4-copy *k*-mers (Supplementary Fig. 47). The model consisted of an exponential decay curve intersecting the x-axis at 0 (representing sequencing errors of non-existent *k*-mers) and 4 Guassian distributions (representing the *k*-mers which are between 1-copy and 4-copy in the WGS sample). Additionally, the model assumed that the mean of the distribution 2/3/4-copy *k*-mers was a multiple of the 1-copy distribution; and that the standard deviation of these subsequent distributions increased as 1/sqrt(n) of the 1-copy peak (consistent with increases in standard deviation from multiple measurements). For any given *k*-mer count, the relative probability of a *k*-mer being 0/1/2/3/4-copy in the sample could then be estimated (Supplementary Fig. 47).

We then used Expectation Maximisation (EM) to call nodes in the haplotype-graph as being 0/1/2/3/4 copy, using 51-mers from the WGS sample. To get a prior for each node, we took the *k*-mers specific to that node and re-fit the amplitudes of our model, and then took the relative amplitudes of the 0/1/2/3/4-copy distributions as the priors for the EM.

After each iteration step of the EM, nodes would update their neighbours (such that a node with many informative *k*-mers could smooth over a noisy adjacent node with relatively few *k*-mers). This neighbour-update was according to a distance-based exponential-decaying penalty: in practice, a node would consider the *k*-mers of an adjacent node (100 kb distant) with a weight of 0.46 (where the full schedule of distance: penalty was – 0:1.000, 1:0.464, 2:0.215, 3:0.100, 4:0.046, 5:0.020, 6:0.010, continuing out to 10 nodes per 1 Mb). A further penalty was applied here if the adjacent nodes did not share identical underlying haplotypes: this was implemented as the fraction of shared haplotypes between nodes (out of all haplotypes seen at either node) to the power of 10 (e.g. if two nodes had 4 contributing haplotypes each, and the overlap between them was 3, then the penalty applied would be 0.75^10^ =0.05); in practice, this meant that only nearby nodes containing practically identical haplotypes would influence each other during the EM. The EM finished when all nodes had converged (minimum step size < 0.001) or 100 iterations had been reached.

## **Calling haplotype-phased pseudo-contigs**

Paths consisting of nodes present in the sample (as per EM) that were connected in the haplotype-graph were selected as pseudo-contigs. Each of these pseudo-contigs corresponded to an assembled sequence for the sample. To identify pseudo-contigs, we masked all nodes that were called as 0-copy or had no informative *k*-mers. For each node, we recursively extended the pseudo-contigs through the edges of the haplotype-graph from left to right – given that no more than one change in ploidy occurred along the path, and that the path did not go through windows which already contained four paths. If no edges would support continuation, we imputed across the next window, given that there was a node in the subsequent window (2 windows downstream) which had the same ploidy and at least 80% of the contributing haplotypes; and a node in-between which shared at least 50% of the same haplotypes. If the path encountered a fork, it would only continue if one of the subsequent nodes contained all of the most-seen haplotypes observed in nodes of the path so far. Additionally, if any of the most-seen genomes in a thread forked into a node with no informative *k*-mers, then we did not allow the path to progress except through imputation. Once a maximal path had been found, the ploidy of all contributing (non-imputed) nodes was reduced by one. Path-finding was repeated until no appropriate starting node could be found anymore.

## **Scoring accuracy of pseudo-molecules from White Rose short reads**

For each pseudo-contig, we counted the assembled haplotypes contributing to each node in the respective path, and selected the most frequently occurring ‘White Rose’ haplotype. This haplotype was then removed, such that it could not be claimed twice for this region. Any node in the pseudo-contig not containing this particular haplotype was called inaccurate. Haplotype switches were defined as any stretch of 2 or more nodes which were inaccurate and additionally contained another incorrect ‘White Rose’ haplotype. Visible nodes were defined as any node containing informative *k*-mers. To test the effects of structural rearrangements on pseudo-contig generation, a second accuracy score was calculated, where nodes overlapping genomic inversions in ‘White Rose’ were filtered out.

**Calculating pseudo-contig N50**

Pseudo-contig N50 is defined as the minimum thread length in a set of pseudo-contigs which cover 50% of the possible nodes (either considering or ignoring pericentromeric regions). The number of possible nodes was calculated as the number of 100 kb windows in the graph multiplied by 4 (the four expected haplotypes from a tetraploid sample). Pseudo-contig length was calculated with each node accounting for 100 kb.

## **Pseudo-contig construction of ‘Russet Burbank’ and evaluation**

We created a pseudo-genome assembly of ‘Russet Burbank’. For the pseudo-contigs, one of the 40 haplotypes was selected as the representative haplotype based on the number of nodes supporting the haplotype and the length of syntenic regions the haplotype contributed to the nodes. Genomic sequence between the first and the last syntenic base in the thread of the selected haplotype was selected as the pseudo-contig sequence. The pseudo-contig sequences for ‘Russet Burbank’ were aligned to the ‘Russet Burbank’ *de novo* assembly using *minimap2* (-cx asm5 –eqx)^10^. The percentage of a pseudo-contig sequence aligned to a haplotype-specific chromosome was calculated as the total size of regions aligned to a haplotype (after removing alignment overlaps) divided by the length of the pseudo-contig sequence, and if the percentage was greater than or equal to 95%, the pseudo-contig sequence was considered as fully aligned and correctly phased (Supplementary Fig. 29).

**Supplementary Figures**


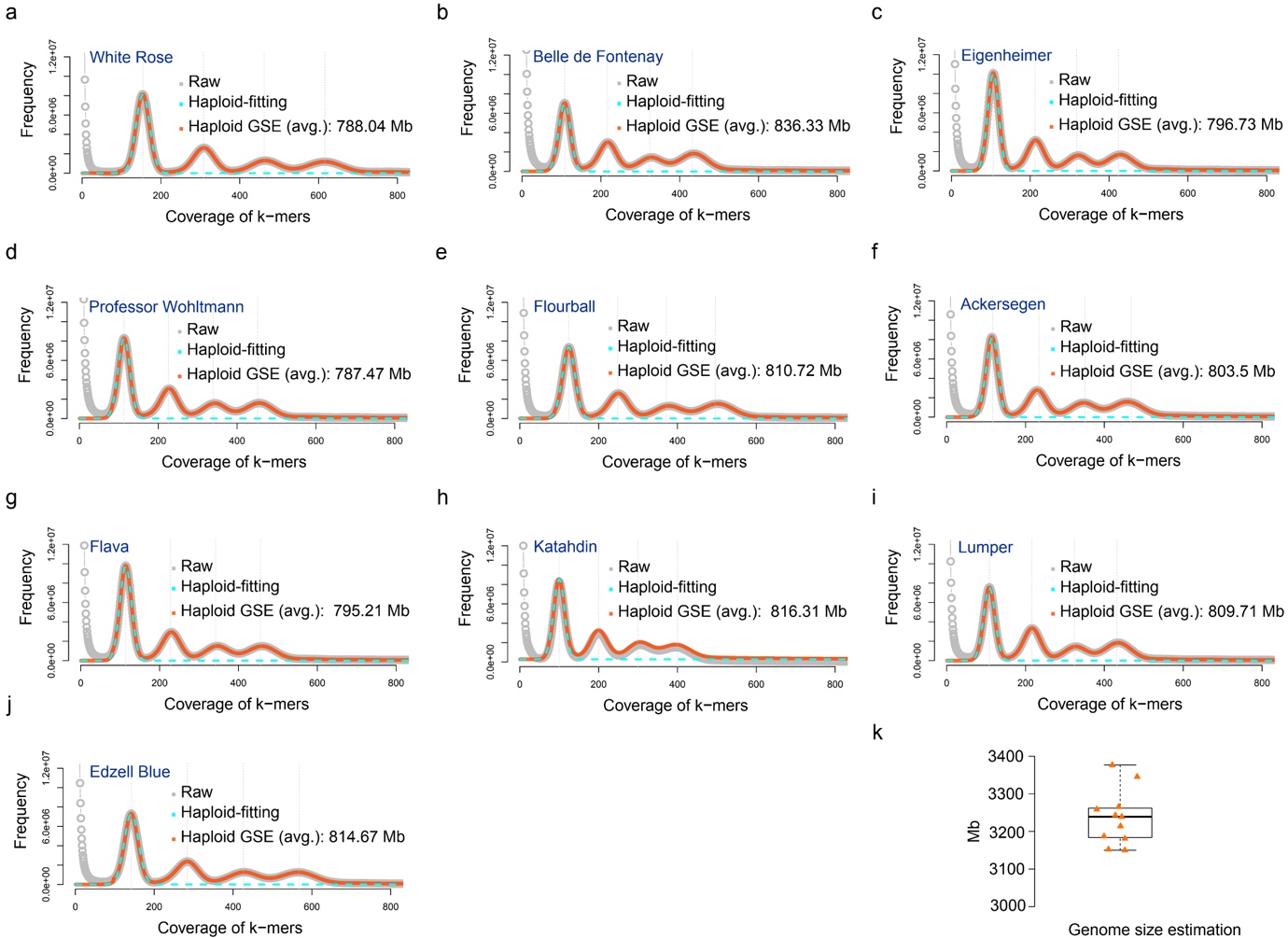


**Supplementary Figure 1. Genomic features. a-j**. Genome size estimation using *k*-mers for ‘White Rose’, ‘Belle de Fontenay’, ‘Eigenheimer’, ‘Professor Wohltmann’, ‘Flourball’, ‘Ackersegen’, ‘Flava’, ‘Katahdin’, ‘Lumper’, ‘Edzell Blue’. **k**. Box plot of genome size estimates of the ten varieties. The box shows the 25th-quantial, median and 75th-quantile. Whiskers extend to the point that is within 1.5 times the inter-quantile range.

**
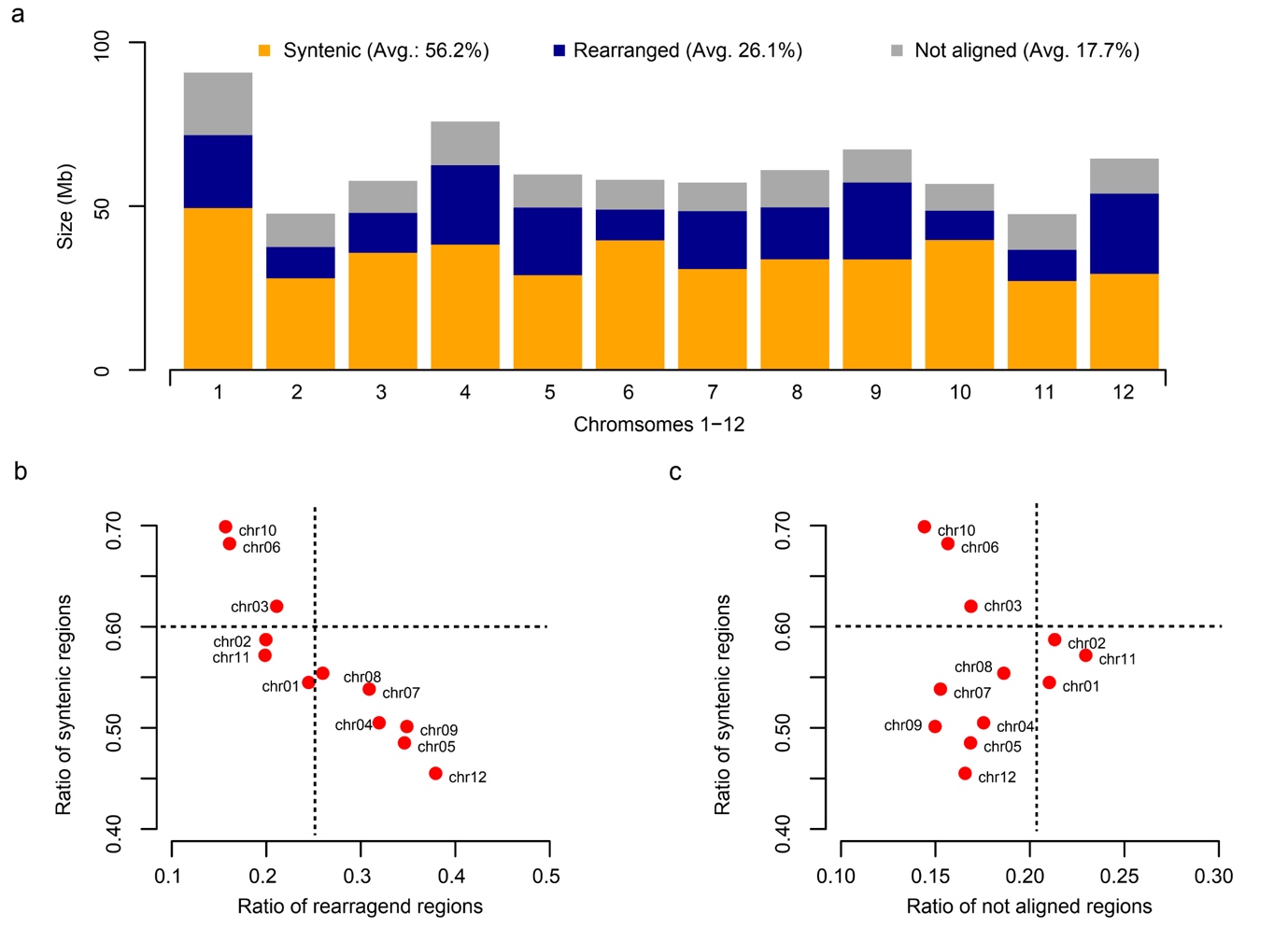
Supplementary Figure 2. Structural comparison of haplotype-specific chromosomes. a.** The size of the genomic regions that were syntenic, structurally rearranged or not aligned when comparing all four haplotypes of a chromosome against each other. **b**. The ratio of rearranged regions was highly variable among chromosomes. Chromosomes 3, 6, and 10 were more structurally conserved than others. **c**. The ratio of not-aligned regions was highly variable among chromosomes. Haplotypes of chromosomes 3, 6, and 10 were more similar to each other.

***See additional figures file.***

**Supplementary Figure 3-14. Features of the 40 haplotypes of chromosome 1-12 of each cultivar.** Each subplot **aa-bn** shows one haplotype of one chromosome (1-12) of one cultivar. For each plot **aa-bn**, the bottom panel shows the densities of genes (in red), transposable elements (TE in orange) and non-aligned regions of pairwise comparisons of all haplotype sequences of each chromosome (in gray, with the average given in black), within 2 Mb windows. Note, for genes, ‘1’ at y-axis means 120 genes per 2 Mb; for TEs and non-aligned regions, the density refers to the total length (bp) of all related features divided by 2,000,000 bp. Tandem repeats (including known centromeric repeats) in cyan, rDNA in blue, contigs are shown in purple, with peri-centromeric region labelled in yellow (Supplementary Methods). The top panel shows the heatmap of read coverage when aligning HiFi reads of 20 wild potato species to the current chromosome sequence, where bars were coloured according to alignment coverage of 0x (yellow) to 50x (red). Note, ‘WhR’, ‘BdF’, ‘EgH’, ‘PrW’, ‘Flo’, ‘Ack’, ‘Fla’, ‘Kat’, ‘Lum’, ‘EdB’ refer to ‘White Rose’, ‘Belle de Fontenay’, ‘Eigenheimer’, ‘Professor Wohltmann’, ‘Flourball’, ‘Ackersegen’, ‘Flava’, ‘Katahdin’, ‘Lumper’, ‘Edzell Blue’ respectively.

**
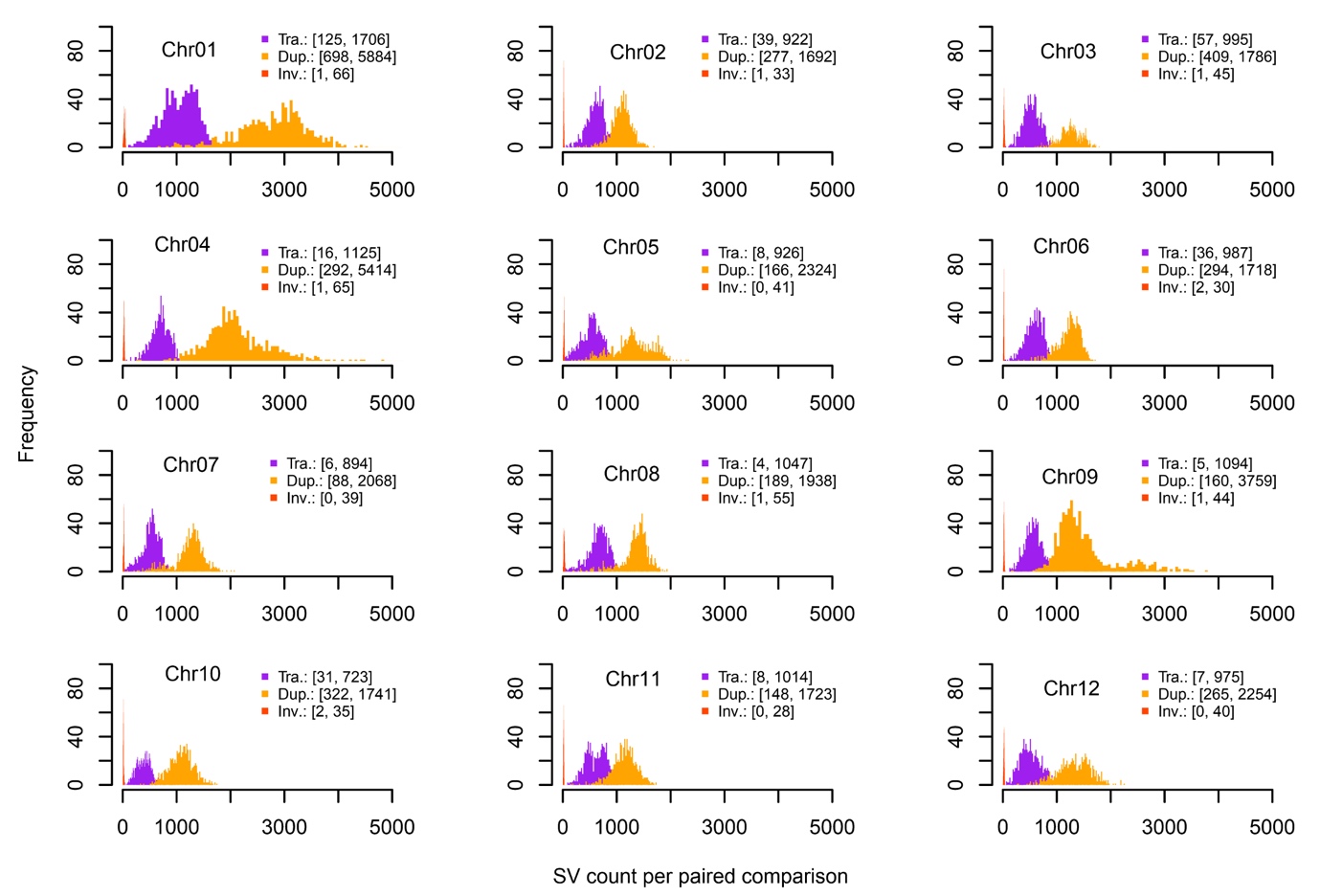
Supplementary Figure 15. Distribution of counts of structural variations (SV) at chromosomes 1-12, including translocations, duplications and inversions**. Note, for each chromosome, there were 780 pairwise comparisons of all 40 haplotypes.

**
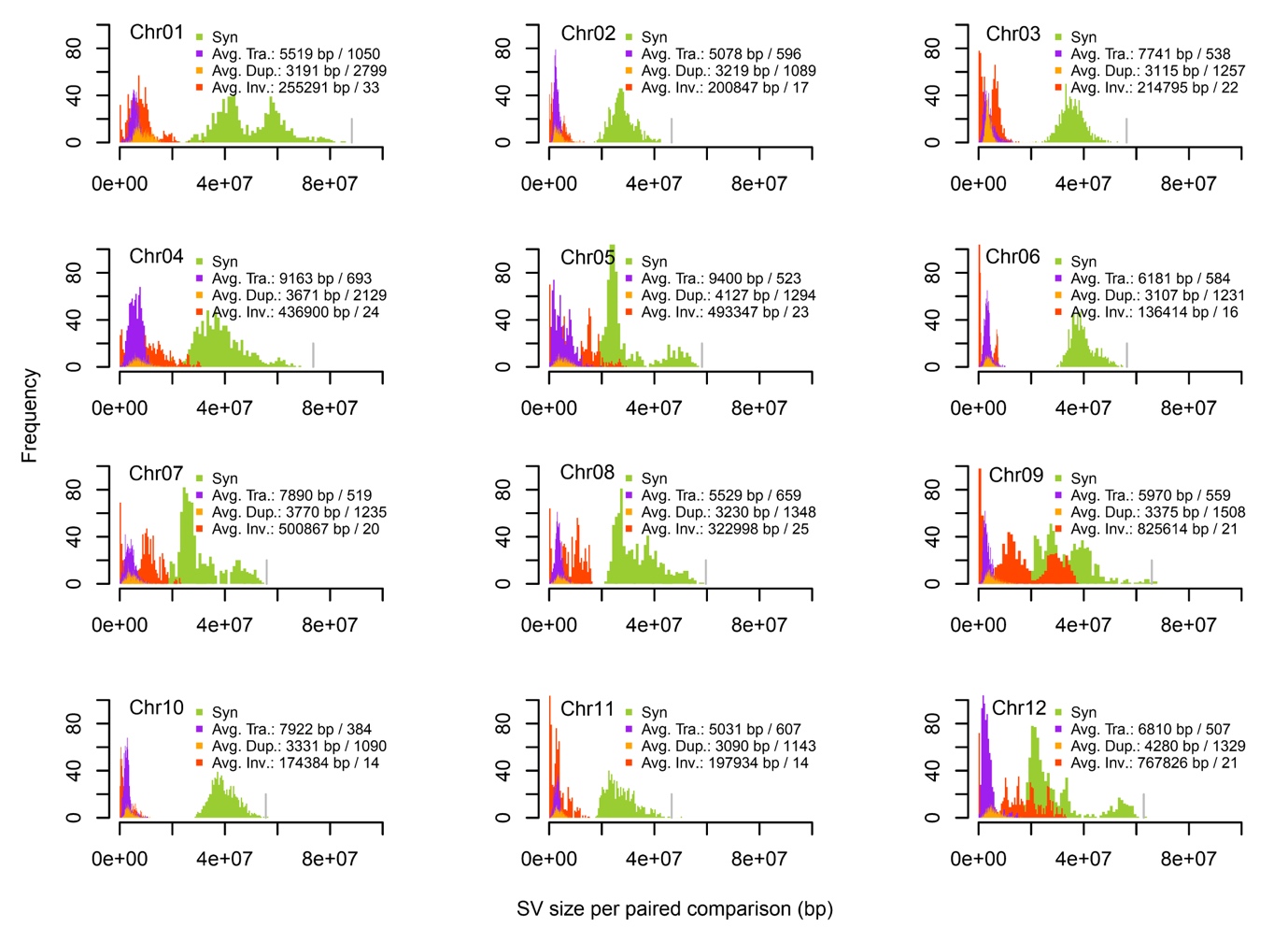
Supplementary Figure 16. Distribution of sizes of structural variations (SV) at chromosomes 1-12, including translocations, duplications and inversions**. Note, for each chromosome, there were 780 pairwise comparisons of all 40 haplotypes.

**
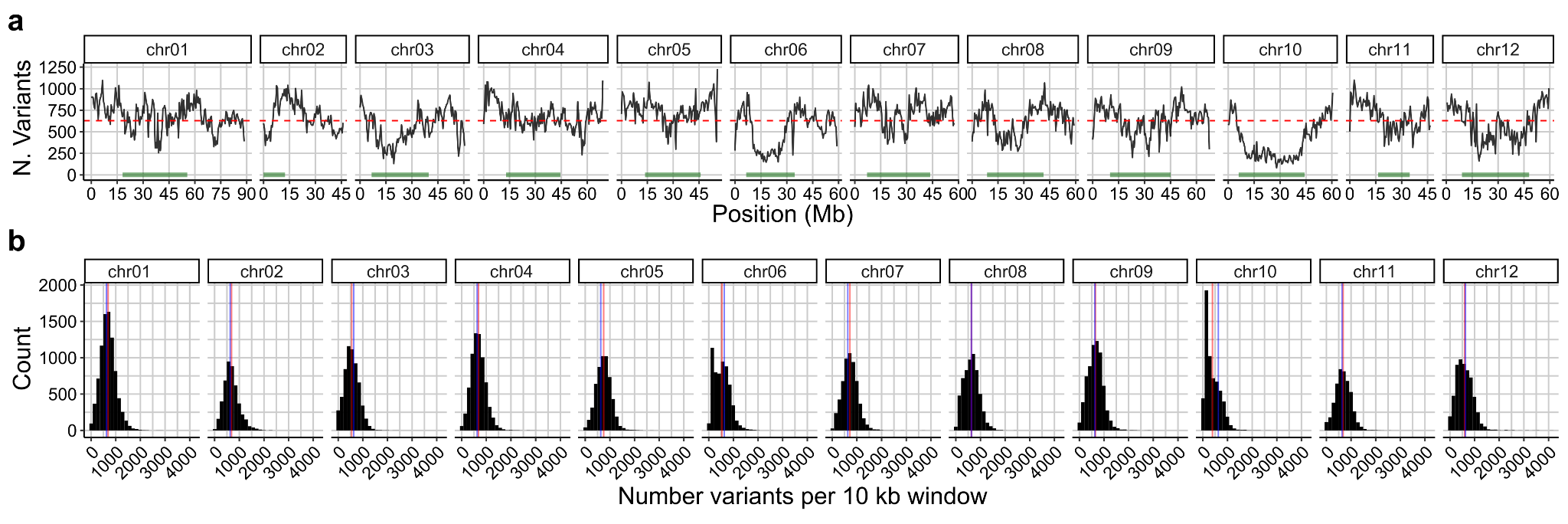
**

**Supplementary Figure 17. Nucleotide-level comparison of the individual haplotypes of each chromosome. a.** Mean estimate of number of variants (x-axis: N. variants) sites per 10 kb window along the genome (y-axis: Position (Mb)). Panels are split by chromosome. Values calculated from 40 haplotypes. Genome-wide mean is indicated with a red dotted line. Pericentromeres are indicated with green bars. **b.** Distribution of the number of variant sites per 10 kb window per chromosome. Mean and median values per chromosome are indicated with red and blue lines respectively.

**
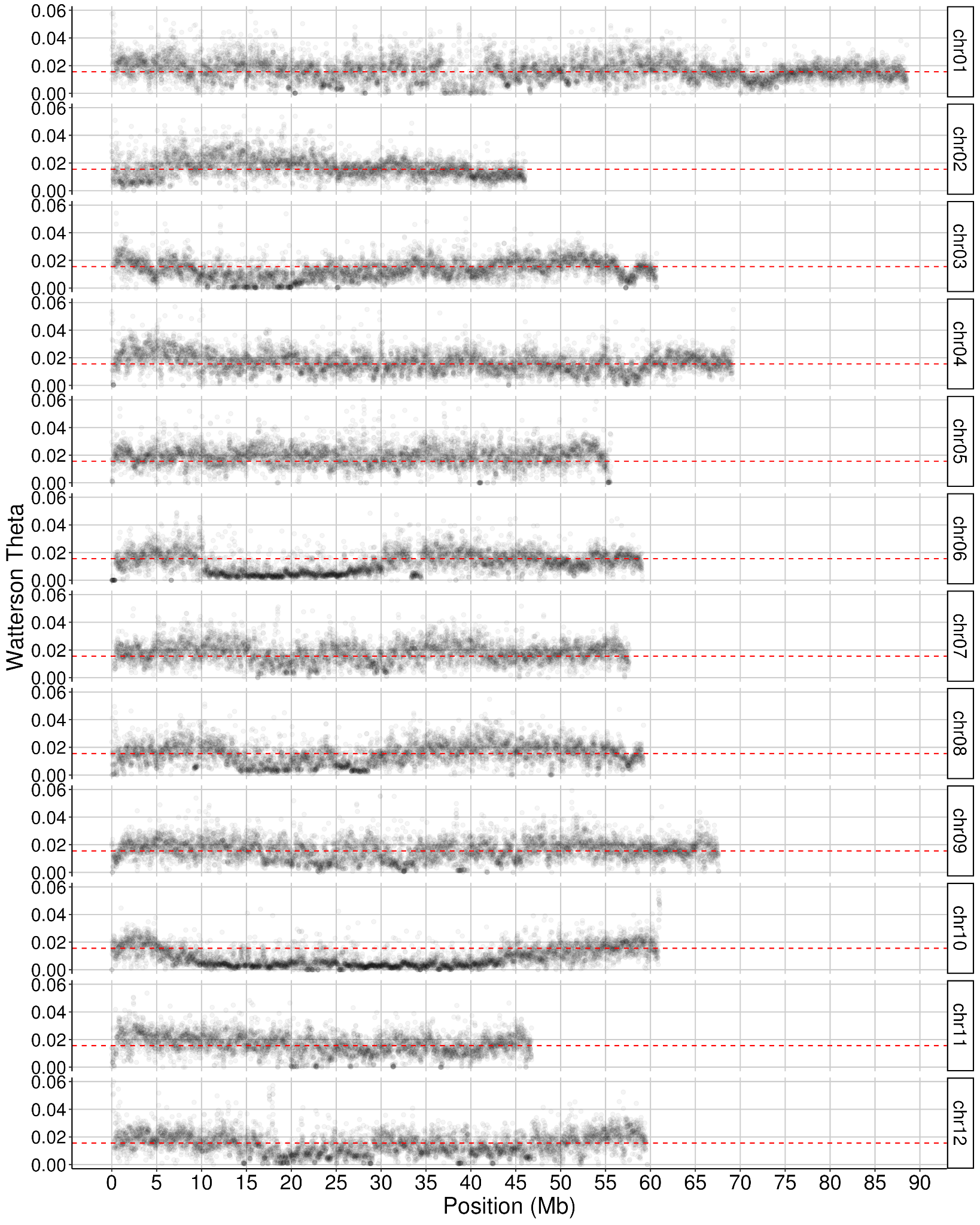
**

**Supplementary Figure 18. Mean estimate of genetic diversity (Watterson theta; θ_w_) per 10 kb window along the genome.** Values calculated per 10kb window from 40 haplotypes. Panels are split by chromosome. Genome-wide means are indicated by red dotted lines.

**
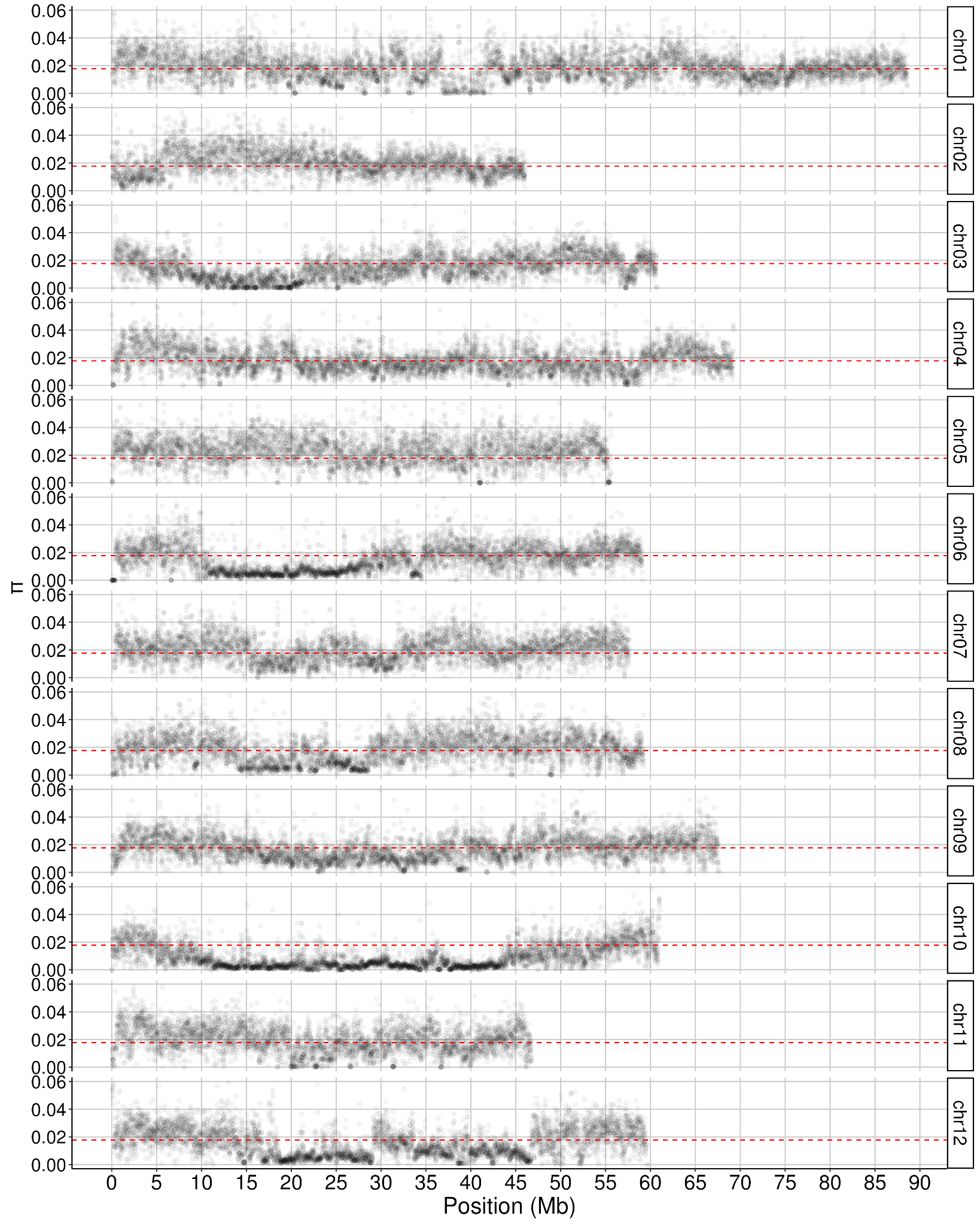
**

**Supplementary Figure 19. Mean estimate of genetic diversity (π) per 10 kb window along the chromosomes.** Values calculated per 10kb window from 40 haplotypes. Panels are split by chromosome. Genome-wide means are indicated with red dotted lines.

***See additional figures file.***

**Supplementary Figure 20. a-l: heatmap of the number of shared haplotypes along chromosomes 1-12.** Each row represents one haplotype. Haplotype names are grouped by sample. ‘WhR’, ‘BdF’, ‘EgH’, ‘PrW’, ‘Flo’, ‘Ack’, ‘Fla’, ‘Kat’, ‘Lum’, ‘EdB’ refer to ‘White Rose’, ‘Belle de Fontenay’, ‘Eigenheimer’, ‘Professor Wohltmann’, ‘Flourball’, ‘Ackersegen’, ‘Flava’, ‘Katahdin’, ‘Lumper’ and ‘Edzell Blue’. Gaps are shown as grey areas.

***See additional figures file.***

**Supplementary Figure 21. a-l: haplotype block sharing at chromosomes 1-12.** Each row represents one haplotype, where individual haplotype blocks are differently coloured. The same colour illustrates matching/identical haplotype block (for each of the twelve chromosomes separately). The haplotypes are clustered by their sharing pattern implying that more similar haplotypes are close to each other (note, chromosome 12 is also shown in Main text Fig. 2f). ‘WhR’, ‘BdF’, ‘EgH’, ‘PrW’, ‘Flo’, ‘Ack’, ‘Fla’, ‘Kat’, ‘Lum’, ‘EdB’ refer to ‘White Rose’, ‘Belle de Fontenay’, ‘Eigenheimer’, ‘Professor Wohltmann’, ‘Flourball’, ‘Ackersegen’, ‘Flava’, ‘Katahdin’, ‘Lumper’, ‘Edzell Blue’ respectively. Gaps are shown as grey areas.

**
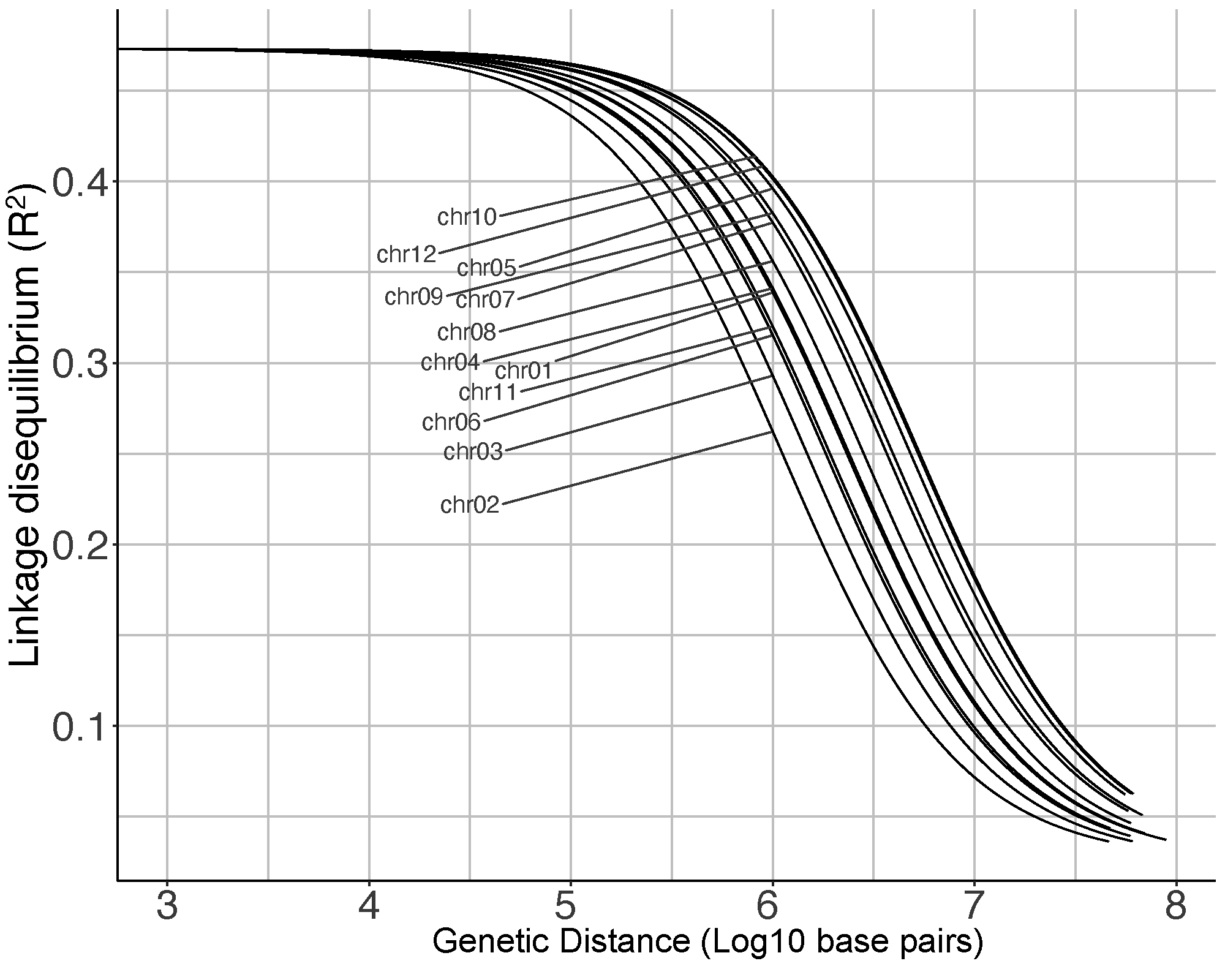
**

**Supplementary Figure 22. Decay in linkage disequilibrium (LD) with genetic distance**. Relationship between LD (R^2^) and physical distance (log_10_) is shown for each chromosome. Lines show non-linear regression models based on Hill & Weir (1988)^48^ and Remington et al. (2001)^49^. Regression produced using genetic variants from 40 haplotypes.

***See additional figures file.***

**Supplementary Figure 23. a-l: linkage disequilibrium between pairs of genomic windows at chromosomes 1-12.** Heatmap of linkage disequilibrium (R^2^) for all comparisons between genomic windows. Values calculated using genotypes from 40 cultivar haplotypes.

***See additional figures file.***

**Supplementary Figure 24. a-m: multispecies coalescence phylogeny of whole genome or a separate chromosome 1-12.** Multispecies coalescence phylogeny for 40 haplotypes of cultivated and wild potato species. Each chromosome was split into 100 kb windows, and maximum likelihood phylogenies were constructed for these windows. A consensus tree for each chromosome was produced using *ASTRAL*. Branch support values are indicated on the trees.

***See additional figures file.***

**Supplementary Figure 25. Admixture analysis for potato haplotypes by chromosome.** Admixture proportions for the 40 haplotypes of cultivated and wild potato species. Each panel **a-l** represents a different chromosome 1-12. Samples are grouped by known ancestry clades (C1+2, C3, C4S, C4N, Cultivar). Each colour represents a different ancestral population (*K* = number of ancestral populations).

***See additional figures file.***

**Supplementary Figure 26. Sequence similarity among potato haplotypes by chromosome**. Sequence similarity among 40 haplotypes of cultivated and wild potato species is illustrated for each chromosome. Each panel **a-l** represents a different chromosome 1-12, showing the genetic relationships among haplotypes. Colours denote the clade to which the closest wild species belong. Cultivar haplotypes were clustered by sequence similarity as in Supplementary Fig. 21.

***See additional figures file.***

**Supplementary Figure 27.** **Introgression analysis using D statistics by chromosome**. Introgression analysis for the 40 haplotypes of cultivated and wild potato species using the f4 statistic. Each panel **a-l** represents a different chromosome 1-12. Colours denote D statistics for introgression tests from clade C4S. For each chromosome, cultivar haplotypes were clustered in the same way as shown in Supplementary Fig. 21.

**Supplementary Figure 28. F-statistics results for tests of introgression with clade C4S.** Each panel **a-l** represents a chromosome 1-12. Bar plots show results for individual tests, with each color representing a specific four-taxon configuration (P1, P2 as sister species; P3 as the candidate introgressed species; O as the outgroup), as detailed in the bottom panel **m**. Species names are consistent with Figure 3. Statistical significance was assessed using f4-statistics, calculated with Dsuite. P-values were computed using a block-jackknife resampling method with 100 kb blocks to account for linkage disequilibrium. Significance thresholds are indicated above the bars: *p-value<0.05, **p-value<0.01, ***p-value<0.001. No adjustments for multiple comparisons were applied. The results highlight chromosome-level patterns of introgression for these clades.

**
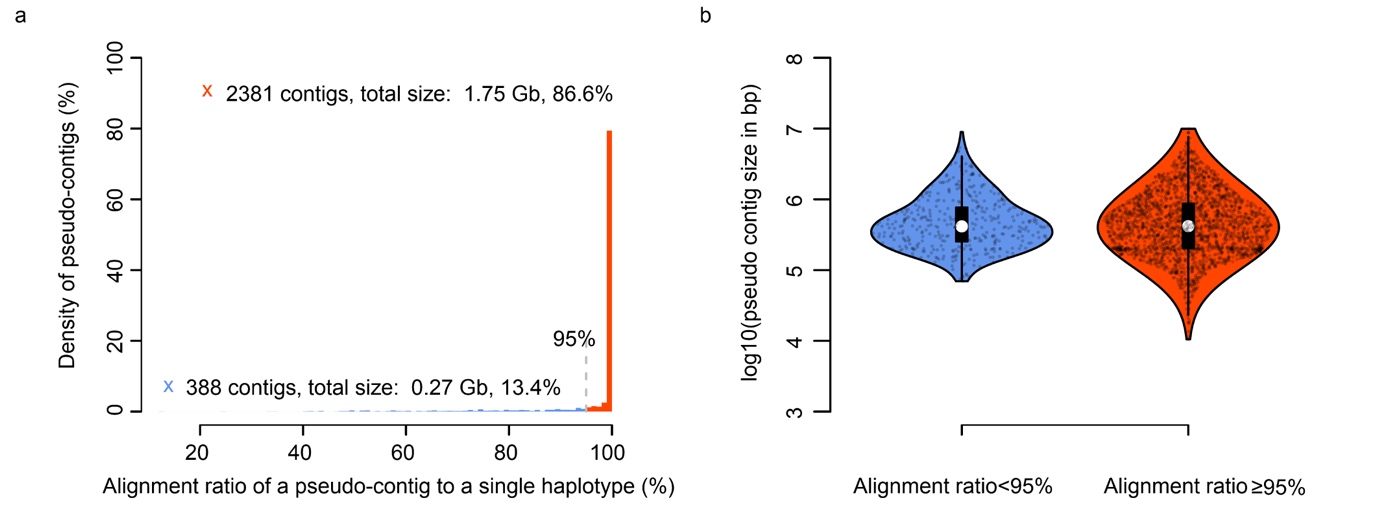
Supplementary Figure 29. Evaluation of phasing accuracy of pseudo-contigs of ‘Russet Burbank’ which were generated by the haplotype-graph approach based on alignments to haplotype-specific chromosomes**. **a.** Density of the highest alignment ratio of a pseudo-contig to a single haplotype-specific chromosome. Overall, 86.6% (1.75 Gb) of all the pseudo-contigs were aligned to a single haplotype, implying high accuracy by the approach. **b.** Correlation of the size of a pseudo-contig with the highest alignment ratio of the pseudo-contig to a single haplotype. Pseudo-contigs shorter than 100 kb were easier to phase than longer ones, while longer ones up to 9.9 Mb were correctly phased.

**
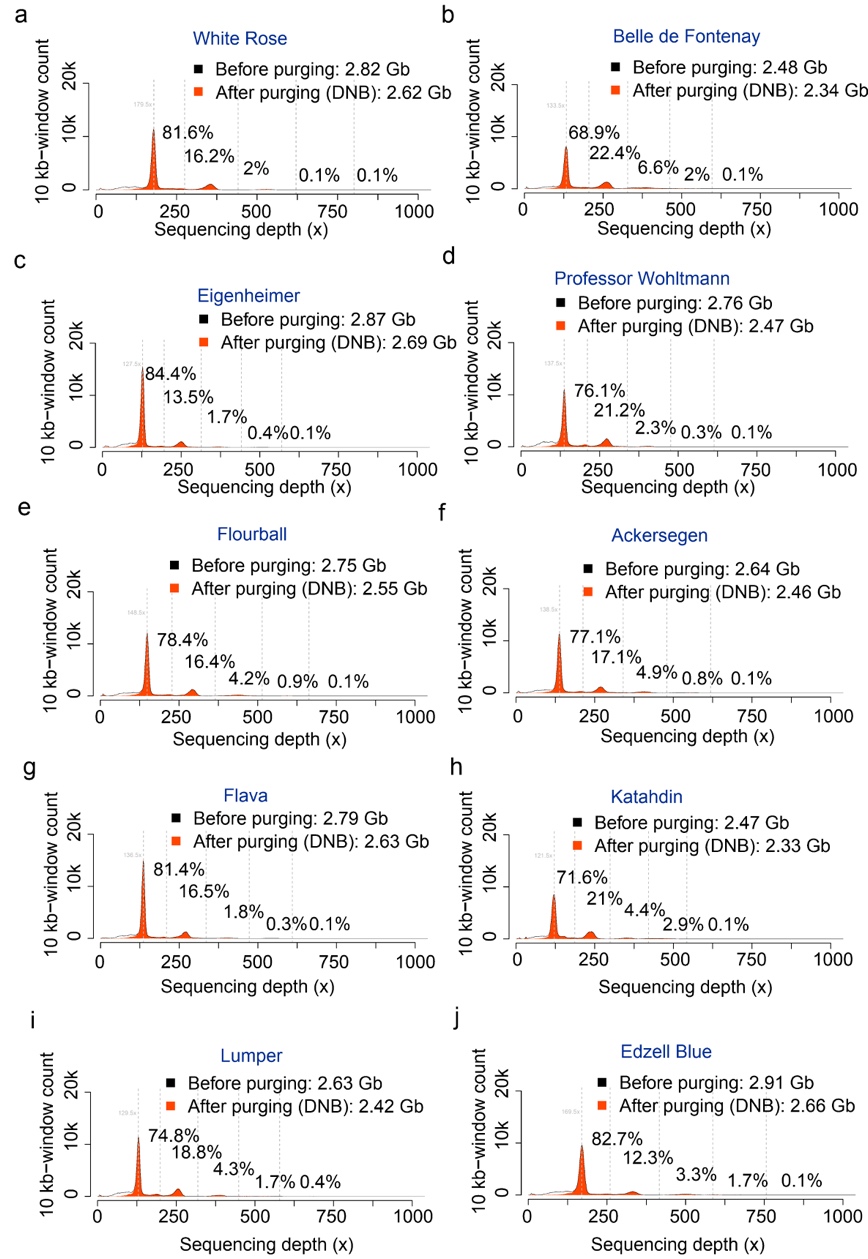
**

**Supplementary Figure 30. Coverage analysis along raw and purged assemblies.** **a-j.** ‘White Rose’, ‘Belle de Fontenay’, ‘Eigenheimer’, ‘Professor Wohltmann’, ‘Flourball’, ‘Ackersegen’, ‘Flava’, ‘Katahdin’, ‘Lumper’, ‘Edzell Blue’. Percentage of coverage-categorized haplotigs, diplotigs, triplotigs and tetraplotigs of the purged assemblies were also collected in Supplementary Table 3.

**
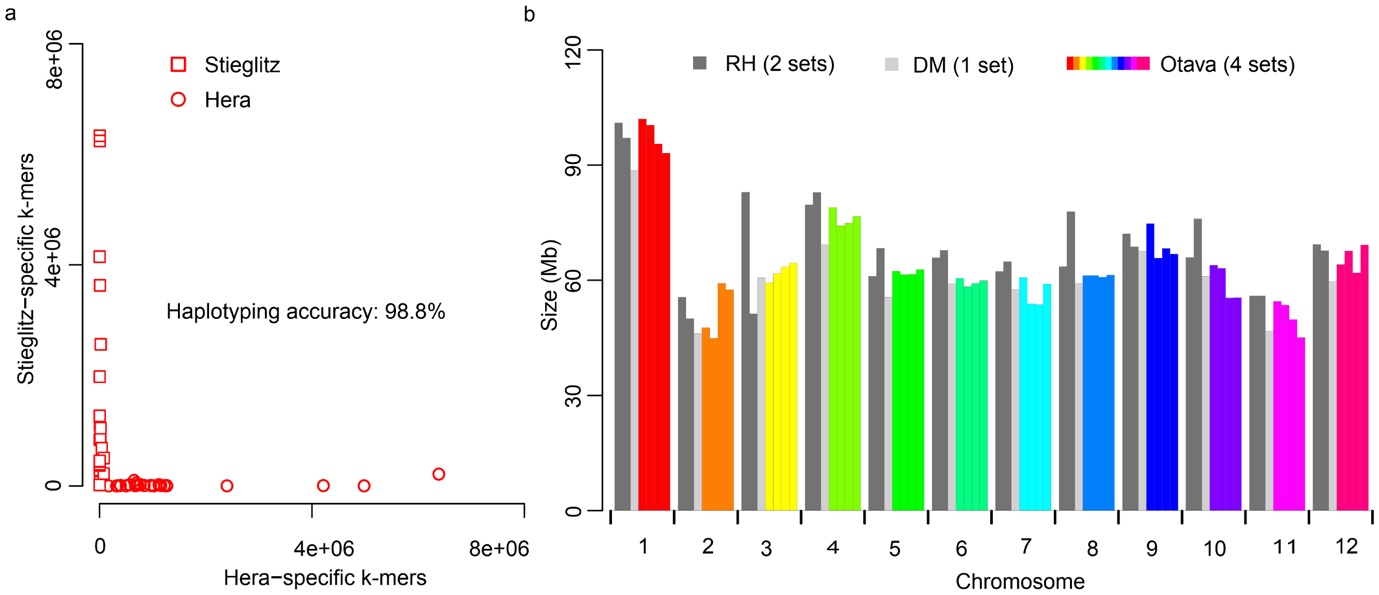
**

**Supplementary Figure 31.**  **Evaluation of the novel Hi-C-based haplotype phasing pipeline using the sequencing data of ‘Otava’**^1^**.** **a**. *K*-mer analysis with parental genome sequencing, as done previously^1^, showed that the method achieved a 98.8% accuracy in phasing the haplotype-specific variants. **b**. The size distribution of the 48 haplotype-specific chromosomes, consistent with DM and RH reference assemblies^2,69^.

**
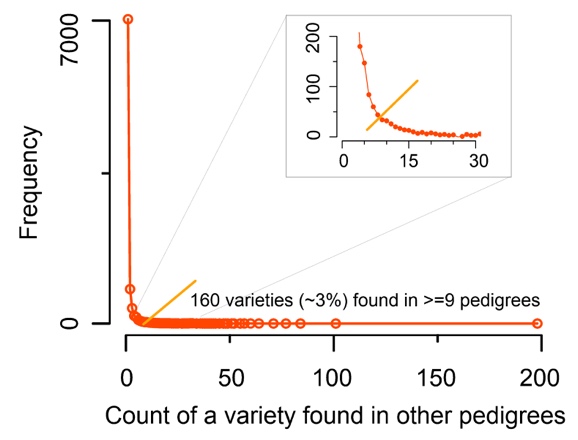
**

**Supplementary Figure 32. Histogram of the number of pedigrees potato cultivars are included in.** There were 160 varieties found in no less than 9 different pedigrees.

**
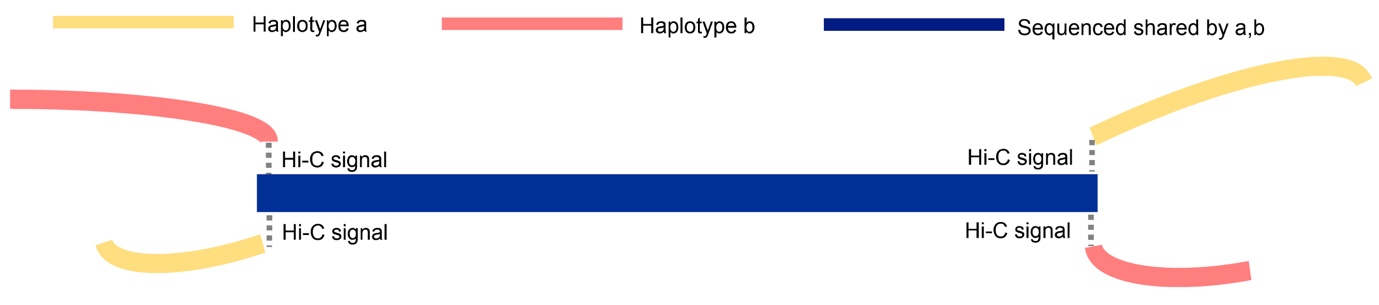
**

**Supplementary Figure 33. Illustration of how haplotype sharing could result in mis-joining of contigs by Hi-C contacts during the assembly process.** Here haplotype-specific sequences in yellow and pink will be linked by Hi-C contacts to the sequence in dark blue which is shared by the two haplotypes, resulting in a mixture of two haplotypes.

***See additional figures file.***

**Supplementary Figures 34-44. a-l: Hi-C contact map of haplotype-specific chromosomes 1-12 of each cultivar.** 34_A: ‘White Rose’, 35_B:‘Belle de Fontenay’, 36_C:‘Eigenheimer’, 37_D:‘Professor Wohltmann’, 38_E:‘Flourball’, 39_F:‘Ackersegen’, 40_G:‘Flava’, 41_H:‘Katahdin’, 42_I:‘Lumper’, 43_J:‘Edzell Blue’ and 44_R:‘Russet Burbank’.

**
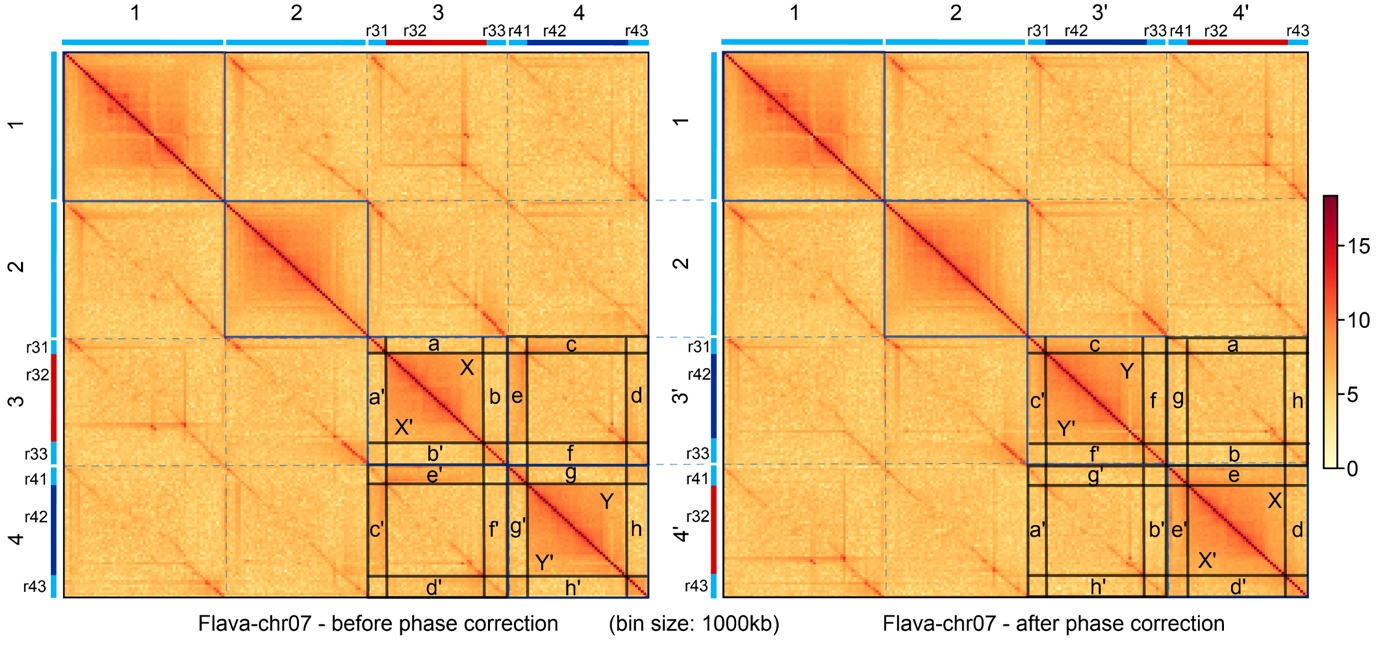
**

**Supplementary Figure 45. Example: identification and correction of haplotype switch errors using Hi-C heatmap of the four haplotypes of a chromosome (here chromosome 7 of ‘Flava’).** The left panel shows the Hi-C heatmap (haplotypes indicated in cyan) before correction, while the right panel shows that the heatmap after correction. In the left panel, the Hi-C heatmap of haplotype 3 was divided into three major blocks a, X and b (or symmetrically a’, X’, b’) following the clear borders between them (i.e., the non-smooth changes in contact intensity between these blocks that reveal haplotype switch errors). These patterns imply that the sequence of haplotype 3 should be divided into three regions r31, r32 and r33. Similarly, also haplotype 4 shows similar patterns, which suggest that its sequence should be divided into three segmental regions r41, r42, and r43. The strong contact intensity in block c/c’ implied that region r31 in haplotype 3 was in strong contact with region r42 in haplotype 4, while block e/e’ implied that region r41 in haplotype 4 was in strong contact with region r32 in haplotype 3. Similarly, the contact intensity in block f/f’ suggested that region r33 in haplotype 3 was in strong contact with region r42 in haplotype 4, while the patterns in block d/d’ implied that region r43 in haplotype 4 was in strong contact with region r32 in haplotype 3. Together, these patterns suggested that r32 in current haplotype 3 (bar in red) and r42 in current haplotype 4 (bar in dark blue) need to be switched and that the original assembly included a haplotype switch error. After correction, as shown in the right panel, a new haplotype 3’ was formed by integrating r31-r42-r33 and a new haplotype 4’ was formed by integrating r41-r32-43. The contact intensity patterns were rearranged correspondingly leading to more smooth contact intensities within the new haplotypes as compared to the original version.

**
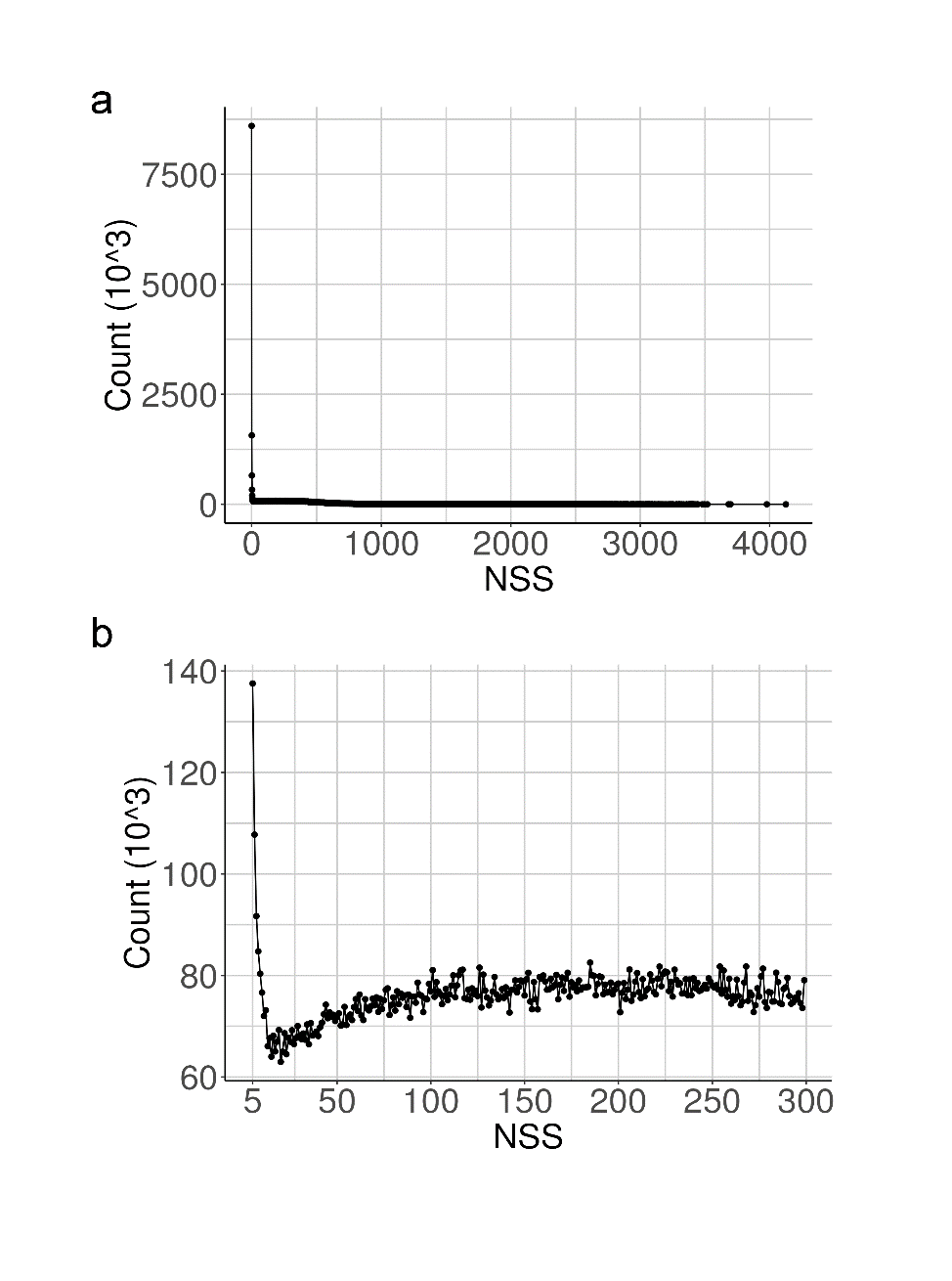
**

**Supplementary Figure 46. Distribution of the number of segregating sites (NSS) between haplotype blocks across 10 kb windows. a.** Distribution of NSS values, showing the overall pattern with a high frequency of low-difference comparisons and a long tail of more divergent comparisons. Values calculated per 10kb window from 40 haplotypes. **b**. A zoomed-in view of the distribution for NSS values ranging from 5 to 300, highlighting the rapid decrease in pairwise comparisons with very low differences and the subsequent stabilization around 10 segregating sites, which guided the threshold selection for defining identical haplotypes.

**
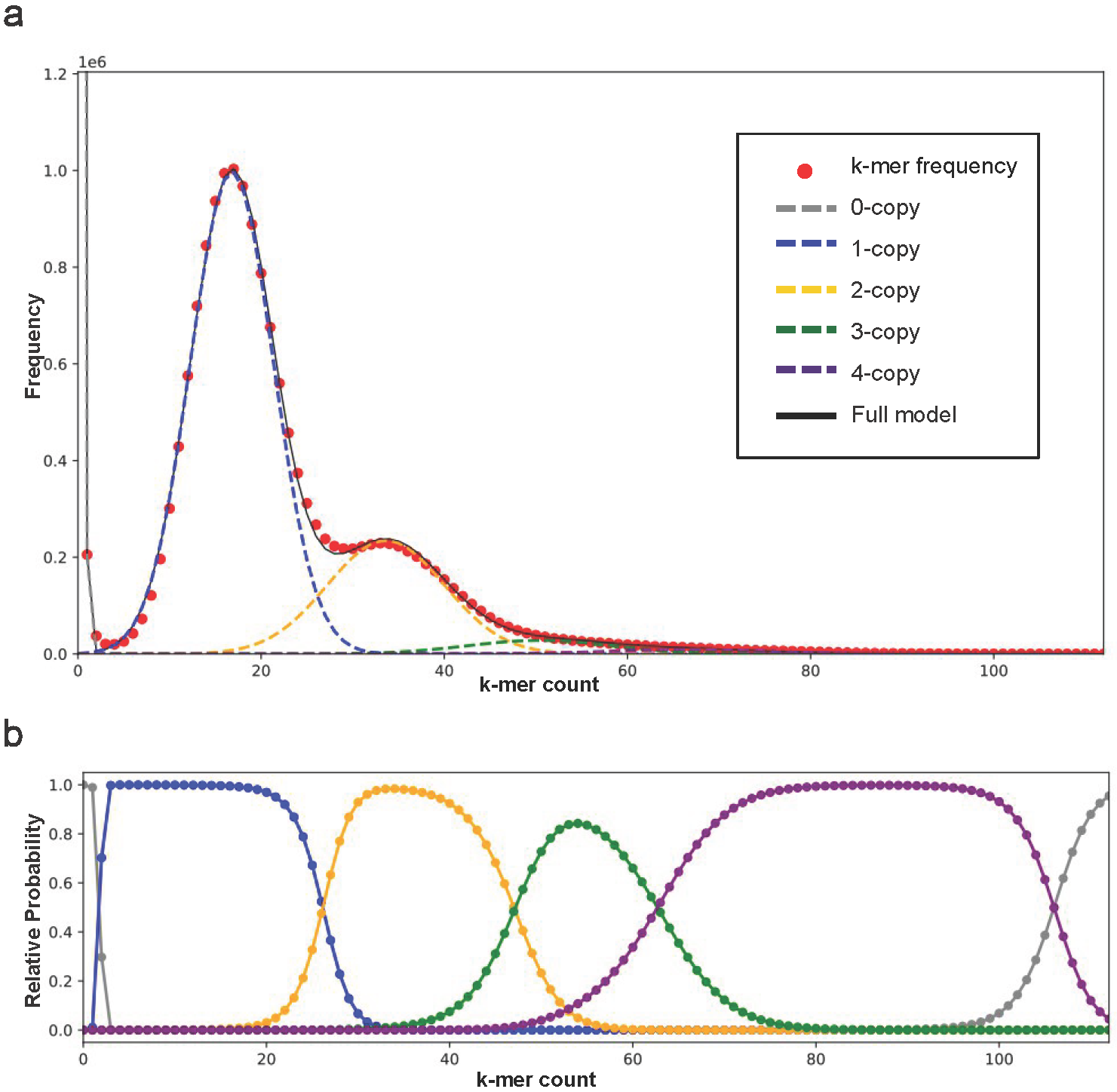
**

**Supplementary Figure 47. Extracting copy-number from *k*-mer count histograms**. **a.** Curve fitting of distributions of *k*-mer counts occurring 0-copy, 1-copy, 2-copy, 3-copy and 4-copy. The model (black line) fit to histogram data (red circles), the underlying distributions of the model are shown in solid grey and dashed coloured lines. **b**. A *k*-mer of any given count is then assigned a relative probability of being 0/1/2/3/4-copy based on the relative heights of the underlying distributions at that position.

# **Supplementary References**

1. Sun, H. et al*.* Chromosome-scale and haplotype-resolved genome assembly of a tetraploid potato cultivar. *Nature Genetics*, **54**, 342-348 (2022).
2. Pham, G. M. et al*.* Construction of a chromosome-scale long-read reference genome assembly for potato. *GigaScience*, **9**, giaa100 (2020).
3. Langmead, B. & Salzberg, S. L. Fast gapped-read alignment with Bowtie 2. *Nature Methods*, **9**, 357-359 (2012).
4. Ossowski, S. et al. Sequencing of natural strains of *Arabidopsis thaliana* with short reads. *Genome Research*, **18**, 2024-2033 (2008).
5. Rowan, B.A. et al. An ultra high-density *Arabidopsis thaliana* crossover map that refines the influences of structural variation and epigenetic features. *Genetics*, **213**, 771-787 (2019).
6. Marçais, G. & Kingsford, C. A fast, lock-free approach for efficient parallel counting of occurrences of k-mers. *Bioinformatics*, **27**, 764-770 (2011).
7. Sun, H., Ding, J., Piednoël, M. & Schneeberger, K. *findGSE*: estimating genome size variation within human and *Arabidopsis* using *k*-mer frequencies. *Bioinformatics*, **34**, 550-557 (2018).
8. Cheng, H., Concepcion, G.T., Feng, X., Zhang, H. & Li, H. Haplotype-resolved de novo assembly using phased assembly graphs with *hifiasm*. *Nature Methods*, **18**, 170-175 (2021).
9. Fukasawa, Y., Ermini, L., Wang, H., Carty, K. & Cheung, M.-S. *LongQC*: a quality control tool for third generation sequencing long read data. *G3 Genes|Genomes|Genetics*, **10**, 1193-1196 (2020).
10. Li, H. *Minimap2*: Pairwise alignment for nucleotide sequences. *Bioinformatics*, **34**, 3094-3100 (2018).
11. Li, H. et al. The sequence alignment/map format and *SAMtools*. *Bioinformatics*, **25**, 2078-2079 (2009).
12. Li, H. & Durbin, R. Fast and accurate short read alignment with Burrows-Wheeler transform. *Bioinformatics*, **25**, 1754-1760 (2009).
13. Zhang, X., Zhang, S., Zhao, Q., Ming, R. & Tang, H. Assembly of allele-aware, chromosomal-scale autopolyploid genomes based on Hi-C data. *Nature Plants*, **5**, 833-845 (2019).
14. Durand, N. C. et al*.* *Juicer* provides a one-click system for analyzing loop-resolution Hi-C experiments. *Cell Syst.*, **3**, 95-98 (2016).
15. Rhie, A., Walenz, B.P., Koren, S. & Phillippy, A.M. *Merqury*: reference-free quality, completeness, and phasing assessment for genome assemblies. *Genome Biology*, **21**, 245 (2020).
16. Ou, S. et al. Benchmarking transposable element annotation methods for creation of a streamlined, comprehensive pipeline. *Genome Biology*, **20**, 275 (2019).
17. Smit, A., Hubley, R. & Green, P. *RepeatMasker* Open-4.0. (2013).
18. Stanke, M., Schöffmann, O., Morgenstern, B. & Waack, S. Gene prediction in eukaryotes with a generalized hidden Markov model that uses hints from external sources. *BMC Bioinformatics*, **7**, 62 (2006).
19. Stanke, M., Diekhans, M., Baertsch, R. & Haussler, D. Using native and syntenically mapped cDNA alignments to improve de novo gene finding. *Bioinformatics*, **24**, 637-644 (2008).
20. Hoff, K.J., Lomsadze, A., Borodovsky, M. & Stanke, M. Whole-Genome Annotation with *BRAKER*. *Methods in Molecular Biology*, **1962**, 65-95 (2019).
21. Lomsadze, A., Burns, P.D. & Borodovsky, M. Integration of mapped RNA-Seq reads into automatic training of eukaryotic gene finding algorithm. *Nucleic Acids Research*, **42**, e119 (2014).
22. Hoff, K.J., Lange, S., Lomsadze, A., Borodovsky, M. & Stanke, M. *BRAKER1*: Unsupervised RNA-Seq-Based Genome Annotation with *GeneMark-ET* and *AUGUSTUS*. *Bioinformatics*, **32**, 767-769 (2016).
23. Kim, D., Paggi, J.M., Park, C., Bennett, C. & Salzberg, S.L. Graph-based genome alignment and genotyping with *HISAT2* and *HISAT*-genotype. *Nature Biotechnology*, **37**, 907-915 (2019).
24. Bao, Z. et al. Genome architecture and tetrasomic inheritance of autotetraploid potato. *Molecular Plant*, **15**, 1211-1226 (2022).
25. Lomsadze, A., Ter-Hovhannisyan, V., Chernoff, Y.O. & Borodovsky, M. Gene identification in novel eukaryotic genomes by self-training algorithm. *Nucleic Acids Research*, **33**, 6494-6506 (2005).
26. Iwata, H. & Gotoh, O. Benchmarking spliced alignment programs including *Spaln2*, an extended version of *Spaln* that incorporates additional species-specific features. *Nucleic Acids Research*, **40**, e161 (2012).
27. Gotoh, O., Morita, M. & Nelson, D.R. Assessment and refinement of eukaryotic gene structure prediction with gene-structure-aware multiple protein sequence alignment. *BMC Bioinformatics*, **15**, 189 (2014).
28. Buchfink, B., Xie, C. & Huson, D.H. Fast and sensitive protein alignment using *DIAMOND*. *Nature Methods*, **12**, 59-60 (2015).
29. Brůna, T., Hoff, K.J., Lomsadze, A., Stanke, M. & Borodovsky, M. *BRAKER2*: automatic eukaryotic genome annotation with *GeneMark-EP+* and *AUGUSTUS* supported by a protein database. *NAR Genomics and Bioinformatics*, **3**, lqaa108 (2021).
30. Hosmani, P.S. et al*.* An improved *de novo* assembly and annotation of the tomato reference genome using single-molecule sequencing, Hi-C proximity ligation and optical maps. *Preprint*, https://doi.org/10.1101/767764 (2019).
31. Kriventseva, E.V. *et al.* *OrthoDB* v10: sampling the diversity of animal, plant, fungal, protist, bacterial and viral genomes for evolutionary and functional annotations of orthologs. *Nucleic Acids Research*, **47**, D807-D811 (2019).
32. Gabriel, L., Hoff, K.J., Brůna, T., Borodovsky, M. & Stanke, M. *TSEBRA*: transcript selector for BRAKER. *BMC Bioinformatics*, **22**, 566 (2021).
33. Shumate, A. & Salzberg, S.L. *Liftoff*: accurate mapping of gene annotations. *Bioinformatics*, **37**, 1639-1643 (2021).
34. Dainat, J. et al. *NBISweden/AGAT*: *AGAT-v1.2.0*. Zenodo (2023). https://doi.org/10.5281/zenodo.8178877.
35. Li, H. Protein-to-genome alignment with *miniprot*. *Bioinformatics*, **39**, btad014 (2023).
36. Quinlan A.R. *BEDTools*: The Swiss-army tool for genome feature analysis. Curr Protoc *Bioinformatics*, **47**, 11.12.1-34 (2014).
37. Manni, M., Berkeley, M.R., Seppey, M., Simão, F.A. & Zdobnov, E.M. *BUSCO* Update: Novel and Streamlined Workflows along with Broader and Deeper Phylogenetic Coverage for Scoring of Eukaryotic, Prokaryotic, and Viral Genomes. *Molecular Biology Evolution*, **38**, 4647-4654 (2021).
38. Wlodzimierz, P., Hong, M., Henderson, I.R. *TRASH*: Tandem Repeat Annotation and Structural Hierarchy. *Bioinformatics*, **39**, btad308 (2023).
39. Freire, R. et al*.* Chromosome-scale reference genome assembly of a diploid potato clone derived from an elite variety. *G3 Genes|Genomes|Genetics*, **11**, jkab330 (2021).
40. Kurtz, S. *et al.* Versatile and open software for comparing large genomes. *Genome biology*, **5**, R12 (2004).
41. Goel, M., Sun, H., Jiao, W.-B. & Schneeberger, K. *SyRI*: finding genomic rearrangements and local sequence differences from whole-genome assemblies. *Genome Biology*, **20**, 277 (2019).
42. Chan, P.P., Lin, B.Y., Mak, A.J. & Lowe, T.M. *tRNAscan-SE 2.0*: improved detection and functional classification of transfer RNA genes. *Nucleic Acids Research*, **49**, 729 9077-9096 (2021).
43. Nawrocki, E.P. & Eddy, S.R. *Infernal* 1.1: 100-fold faster RNA homology searches. *Bioinformatics*, **29**, 2933-5 (2013).
44. Nei, M. & Li, W.H. Mathematical model for studying genetic variation in terms of restriction endonucleases. *PNAS*, **76**, 5269-5273 (1979).
45. Watterson, G.A. On the number of segregating sites in genetical models without recombination. *Theoretical Population Biology*, **7**, 256-276 (1975).
46. Hill, W.G. & Robertson, A. Linkage disequilibrium in finite populations. *Theoretical and Applied Genetics*, **38**, 226-231 (1968).
47. Weir, B.S. Inferences about linkage disequilibrium. *Biometrics*, **35**, 235 (1979).
48. Hill, W.G. & Weir, B.S. Variances and covariances of squared linkage disequilibria in finite populations. *Theoretical Population Biology*, **33**, 54-78 (1988).
49. Remington, D.L. et al*.* Structure of linkage disequilibrium and phenotypic associations in the maize genome. *PNAS*, **98**, 11479-11484 (2001).
50. Tang, D. et al*.* Genome evolution and diversity of wild and cultivated potatoes. *Nature*, **606**, 535-541 (2022).
51. Poplin, R. et al. A universal SNP and small-indel variant caller using deep neural networks. *Nature Biotechnology*, **36**, 983-987 (2018).
52. Yun, T. et al. Accurate, scalable cohort variant calls using *DeepVariant* and *GLnexus*. *Bioinformatics*, **36**, 5582-5589, (2021).
53. Danecek, P. et al. The variant call format and *VCFtools*. *Bioinformatics*, **27**, 2156-2158 (2011).
54. Pedersen, B.S., Quinlan, A.R. *Mosdepth*: quick coverage calculation for genomes and exomes. *Bioinformatics*, **34**, 867-868 (2018).
55. Nguyen, L.T., Schmidt, H.A., von Haeseler, A., Minh, B.Q. *IQ-TREE*: a fast and effective stochastic algorithm for estimating maximum-likelihood phylogenies. *Molecular Biology Evolution*, **32**, 268-74 (2015).
56. Zhang, C., Rabiee, M., Sayyari, E., Mirarab, S. *ASTRAL-III*: polynomial time species tree reconstruction from partially resolved gene trees. *BMC Bioinformatics*, **19**, 153 (2018).
57. Purcell, S. et al. *PLINK*: a tool set for whole-genome association and population-based linkage analyses. *The American Journal of Human Genetics*, **81**, 559-75 (2007).
58. Alexander, D.H., Novembre, J., Lange, K. Fast model-based estimation of ancestry in unrelated individuals. *Genome Research*, **19**, 1655-64 (2009).
59. Durand, E.Y., Patterson, N., Reich, D., Slatkin, M. Testing for ancient admixture between closely related populations. *Molecular Biology Evolution*, **28**, 2239-52 (2011).
60. Reich, D. N. et al. Reconstructing Indian population history. *Nature*, **461**, 489-494 (2009).
61. Patterson, N. et al. Ancient Admixture in Human History. *Genetics*, **192**, 3, 1065-1093 (2012).
62. Malinsky, M., Matschiner, M., Svardal, H. *Dsuite* - Fast D-statistics and related admixture evidence from VCF files. *Molecular Ecology Resources*, **21**, 584-595 (2021).
63. Li, H., Feng, X. & Chu, C. The design and construction of reference pangenome graphs with *minigraph*. *Genome Biology*, **21**, 265 (2020).
64. R Core Team. *R: A Language and Environment for Statistical Computing*. (*R* Foundation for Statistical Computing, Vienna, Austria, 2021).
65. Emms, D. M. & Kelly, S. *OrthoFinder*: phylogenetic orthology inference for comparative genomics. *Genome Biology*, **20**, 238 (2019).
66. Buchfink, B., Reuter, K. & Drost, H.-G. Sensitive protein alignments at tree-of-life scale using *DIAMOND*. *Nature Methods*, **18**, 366-368 (2021).
67. Camacho, C. & Madden, T. *BLAST+* Release Notes. in *BLAST® Help [Internet]* (National Center for Biotechnology Information (US), 2023).
68. Ebler, J. et al. Pangenome-based genome inference allows efficient and accurate genotyping across a wide spectrum of variant classes. *Nature Genetics*, **54**, 518-525 (2022).
69. Zhou, Q. et al. Haplotype-resolved genome analyses of a heterozygous diploid potato. *Nature Genetics*, **52**, 1018-1023 (2020).
