# Supplementary material for: The phased pan-genome of tetraploid European potato
Source: Nature. 2025 Apr 16;642(8067):389–97. doi: 10.1038/s41586-025-08843-0 (PMC12158759; doi:10.1038/s41586-025-08843-0)
Supplement: Supplementary file 2 — Reporting Summary [file 41586_2025_8843_MOESM2_ESM.pdf]

nature portfolio

Corresponding author(s): Korbinian Schneeberger
Last updated by author(s): Feb 3, 2025

Reporting Summary

Nature Portfolio wishes to improve the reproducibility of the work that we publish. This form provides structure for consistency and transparency in reporting. For further information on Nature Portfolio policies, see our Editorial Policies and the Editorial Policy Checklist.

Statistics

For all statistical analyses, confirm that the following items are present in the figure legend, table legend, main text, or Methods section.

n/a	Confirmed	
☐	☒	The exact sample size (*n*) for each experimental group/condition, given as a discrete number and unit of measurement
☒	☐	A statement on whether measurements were taken from distinct samples or whether the same sample was measured repeatedly
☐	☒	The statistical test(s) used AND whether they are one- or two-sided<br>*Only common tests should be described solely by name; describe more complex techniques in the Methods section.*
☒	☐	A description of all covariates tested
☐	☒	A description of any assumptions or corrections, such as tests of normality and adjustment for multiple comparisons
☐	☒	A full description of the statistical parameters including central tendency (e.g. means) or other basic estimates (e.g. regression coefficient) AND variation (e.g. standard deviation) or associated estimates of uncertainty (e.g. confidence intervals)
☐	☒	For null hypothesis testing, the test statistic (e.g. *F*, *t*, *r*) with confidence intervals, effect sizes, degrees of freedom and *P* value noted<br>*Give P values as exact values whenever suitable.*
☒	☐	For Bayesian analysis, information on the choice of priors and Markov chain Monte Carlo settings
☒	☐	For hierarchical and complex designs, identification of the appropriate level for tests and full reporting of outcomes
☐	☒	Estimates of effect sizes (e.g. Cohen's *d*, Pearson's *r*), indicating how they were calculated

Our web collection on statistics for biologists contains articles on many of the points above.

Software and code

Policy information about availability of computer code

Data collection: No code used for data collection.

Data analysis: All routine analysis were performed using tools publicly available. DNBseq reads were filtered with SOAPnuke (version 2.1.7). Short/long reads were aligned using bowtie2 (2.2.8)/minimap2 (2.20-r1061). SNPs were called using tools shore (v 0.7.1). BAM, VCF file processing and sequencing depth analysis were performed using samtools (v1.9;v1.11) and bedtools (v2.29.1). PacBio sequence reads were assembled using hifiasm (v0.7). Hi-C data were processed using ALLHiC (v0.9.13), juicer (v1.6) and juicebox(v2.13.07). k-mers were processed with Jellyfish (version 2.2.10) and Merqury (version 1.3). Genome annotation was performed using EDTA (version 2.1.0),Repeatmasker (version 4.1.2-p1), BRAKER (version 2.1.6), HISAT2 (version 2.2.1), TSEBRA (version 1.0.3), liftoff (version 1.6.2), AGAT (version 1.0.0), and evaluated using BUSCO (version 5.2.2) .TRASH (version 1.2) was used for tandem repeat identifiction. Sequence alignment was performed using nucmer3 (version 3.1). Structural variations were identified using SyRI (v1.6) based on nucmer3 sequence alignments.rDNA, tDNA was detected using tRNAscan-SE (version 2.0.5), barrnap (version 0.9), and infernal (version 1.1.4) . The script infernal-tblout2gff.pl (version 1.1.4) was used to convert the result of infernal into GFF format. Sequence-level pan-genome was constructed using minigraph (version0.20-r55966); gene-level pan-genome was constructed usingOrthoFinder (version 2.5.5), diamond (version 2.0.13), and Blast (version 2.12.0+). mosdepth (version 0.3.1) was used for genomic coverage analysis. The distribution of SV along the genome was determined by identifying syntenic regions in all pairwise comparisons to DM using Msyd (version 1.0; https://github.com/schneebergerlab/msyd). Variant calling with long read alignment was performed using DeepVariant (v1.4.0)54. GVCF files were merged using GLnexus (v1.2.7). VCF files were split into windows using jvarkit (v2024.04.20) (https://github.com/lindenb/jvarkit). Maximum likelihood phylogenetic trees per window were constructed using IQ-TREE (v2.1.2). Consensus trees were built including all window trees per chromosome and for the whole genome using ASTRAL (v5.7.8). Genetic variants were pruned by LD using PLINK (1.90b6.18). Admixture analysis was conducted independently for each chromosome using ADMIXTURE (1.3.0). The package Dsuite (version 0.5) was used to calculate D-statistics, f4-ratio, and associated p-values in ABBA-BABA

analysis. Customized code used in analysis supporting this work is available at https://github.com/HeQSun/tetraDecoder, https://github.com/schneebergerlab/pantohap and https://github.com/schneebergerlab/haplotype_threading.

For manuscripts utilizing custom algorithms or software that are central to the research but not yet described in published literature, software must be made available to editors and reviewers. We strongly encourage code deposition in a community repository (e.g. GitHub). See the Nature Portfolio guidelines for submitting code & software for further information.

Data

Policy information about availability of data

All manuscripts must include a data availability statement. This statement should provide the following information, where applicable:

- Accession codes, unique identifiers, or web links for publicly available datasets
- A description of any restrictions on data availability
- For clinical datasets or third party data, please ensure that the statement adheres to our policy

The haplotype-resolved genome assemblies and the gene annotation files of the 11 varieties analyzed in this study are available at Zenodo (version 2: 10.5281/zenodo.10617012; version 2.1: 10.5281/zenodo.14053896). All high-throughput sequencing data generated in this study are available at NCBI under Bioproject PRJNA1074690. PacBio long read sequencing of 20 wild species were retrieved from NCBI under BioProject PRJNA754534, and PacBio long read, short read and Omni-C sequencings of cultivar 'Otava' were retrieved from NCBI under Bioproject PRJNA726019. Reference data (v6.1) of DM 1-3 516 R44 was downloaded at Spud DB (https://spuddb.uga.edu/download.shtml), and dAg1_v1.0 data was downloaded from figshare (doi.org/10.6084/m9.figshare.14604780).

Research involving human participants, their data, or biological material

Policy information about studies with human participants or human data. See also policy information about sex, gender (identity/presentation), and sexual orientation and race, ethnicity and racism.

Reporting on sex and gender	Not applicable.
Reporting on race, ethnicity, or other socially relevant groupings	Not applicable.
Population characteristics	Not applicable.
Recruitment	Not applicable.
Ethics oversight	Not applicable.

Note that full information on the approval of the study protocol must also be provided in the manuscript.

Field-specific reporting

Please select the one below that is the best fit for your research. If you are not sure, read the appropriate sections before making your selection.

☒ Life sciences ☐ Behavioural & social sciences ☐ Ecological, evolutionary & environmental sciences

For a reference copy of the document with all sections, see nature.com/documents/nr-reporting-summary-flat.pdf

Life sciences study design

All studies must disclose on these points even when the disclosure is negative.

Sample size	We searched the Wageningen University Potato Pedigree Database (with pedigree data from over 9,500 potato samples) for cultivars grown in the 19th century or those foundational to modern breeding efforts. From 164 cultivars in the database that fitted our criteria, 19 were available in the Gross Lüsewitz Potato Collections (GLKS) of the IPK Gene Bank. PCA analysis with whole-genome short-read sequencings of the 19 samples confirmed one sample as S. tuberosum spp. Andigena, and we selected the ten most diverse subset among the others (covering 90% of the variations among of the 19 samples) to generate the pan-genome of European potato (which was found to cover a high percentage (85%) of the haplotypes in tetraploid potatoes).
Data exclusions	By analyzing the variations using whole-genome short read sequencing among the nineteen varieties, we selected the ten representing over 90% of the variations of the nineteen varieties. Additionally, another cultivar 'Russet Burbank' was also assembled.
Replication	No biological or technical replicates were generated in this study, as it focused on the assembly and analysis of tetraploid potato genomes rather than experimental treatments.<br><br>However, to ensure the reproducibility and reliability of our findings, all genome assemblies were validated using multiple quality control measures, as detailed in the Supplementary Information. Assembly completeness was assessed using BUSCO, base accuracy was estimated through Merqury QV scores, and haplotype phasing accuracy was evaluated using Hi-C data. Additionally, our assembly methodology was evaluated by reconstructing a previously assembled genome, confirming the robustness of our approach. Sequence diversity analyses, haplotype reconstruction, and introgression detection were performed with clearly defined pipelines invovling existing or newly developed

bioinformatics tools, which are all publicly available. The assembled genomes and all relevant datasets generated or used in this study are also publicly available. All these will allow independent verification of our study.

Randomization: Randomization does not apply in genome sequencing and assembly

Blinding: No group allocation was needed in this study.

Reporting for specific materials, systems and methods

We require information from authors about some types of materials, experimental systems and methods used in many studies. Here, indicate whether each material, system or method listed is relevant to your study. If you are not sure if a list item applies to your research, read the appropriate section before selecting a response.

Materials & experimental systems

n/a	Involved in the study
☒	☐ Antibodies
☒	☐ Eukaryotic cell lines
☒	☐ Palaeontology and archaeology
☒	☐ Animals and other organisms
☒	☐ Clinical data
☒	☐ Dual use research of concern
☐	☒ Plants

Methods

n/a	Involved in the study
☒	☐ ChIP-seq
☒	☐ Flow cytometry
☒	☐ MRI-based neuroimaging

Dual use research of concern

Policy information about dual use research of concern

Hazards

Could the accidental, deliberate or reckless misuse of agents or technologies generated in the work, or the application of information presented in the manuscript, pose a threat to:

No	Yes
☒	☐ Public health
☒	☐ National security
☒	☐ Crops and/or livestock
☒	☐ Ecosystems
☒	☐ Any other significant area

Experiments of concern

Does the work involve any of these experiments of concern:

No	Yes
☒	☐ Demonstrate how to render a vaccine ineffective
☒	☐ Confer resistance to therapeutically useful antibiotics or antiviral agents
☒	☐ Enhance the virulence of a pathogen or render a nonpathogen virulent
☒	☐ Increase transmissibility of a pathogen
☒	☐ Alter the host range of a pathogen
☒	☐ Enable evasion of diagnostic/detection modalities
☒	☐ Enable the weaponization of a biological agent or toxin
☒	☐ Any other potentially harmful combination of experiments and agents

Plants

Seed stocks	Materials were requested from the Gross Luesewitz Potato Collections (GLKS) of the IPK Gene Bank.
Novel plant genotypes	No novel palnt genenotype generated in this study.
Authentication	Not applicable.
