# Supplementary figures and images for: The phased pan-genome of tetraploid European potato (part 2 of 2)
Source: Nature. 2025 Apr 16;642(8067):389–97. doi: 10.1038/s41586-025-08843-0 (PMC12158759; doi:10.1038/s41586-025-08843-0)

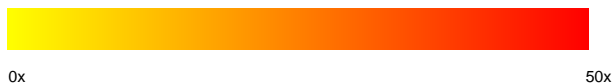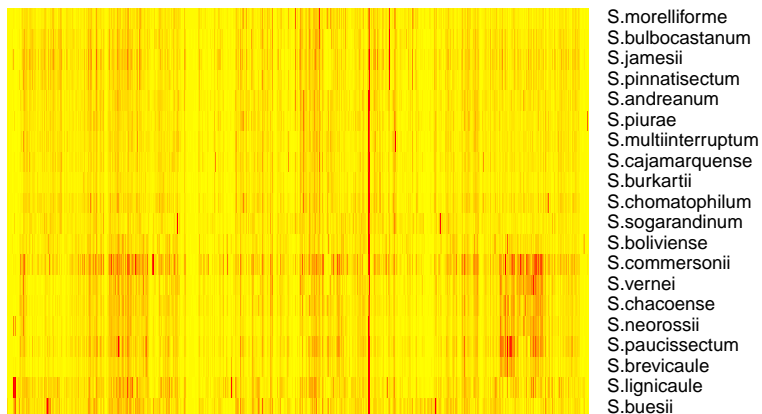

— TE — Gene — Non-aln • TanRep ♦ CenRep ■ rDNA

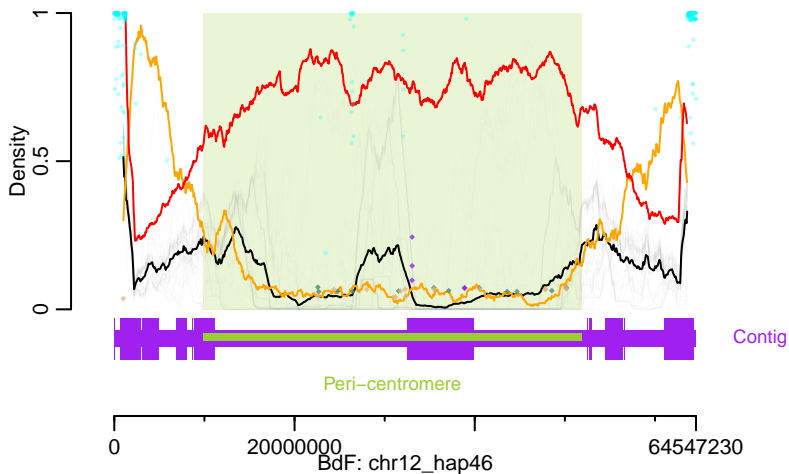

Supplement: Supplementary file 5 — Supplementary Figs. 3–14, 20, 21, 23–27 and 34–44. [file 41586_2025_8843_MOESM5_ESM.zip › suppl_figure_3_to_14/suppl_figure_14_chr12/suppl_figure_14al_46_B_BdF.pdf]

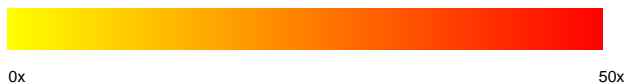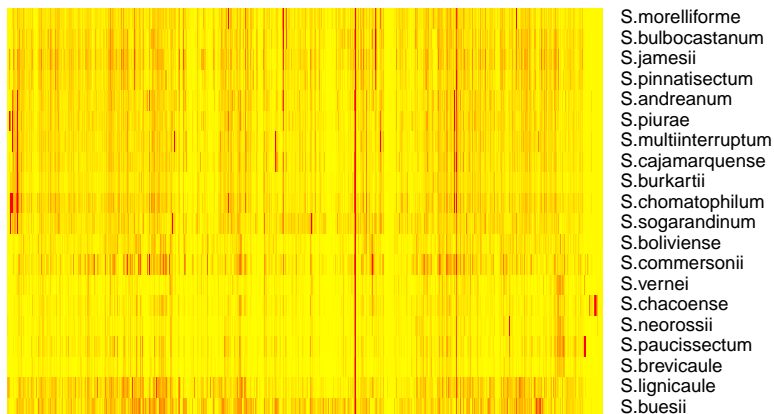

— TE — Gene — Non-aln • TanRep ♦ CenRep ■ rDNA

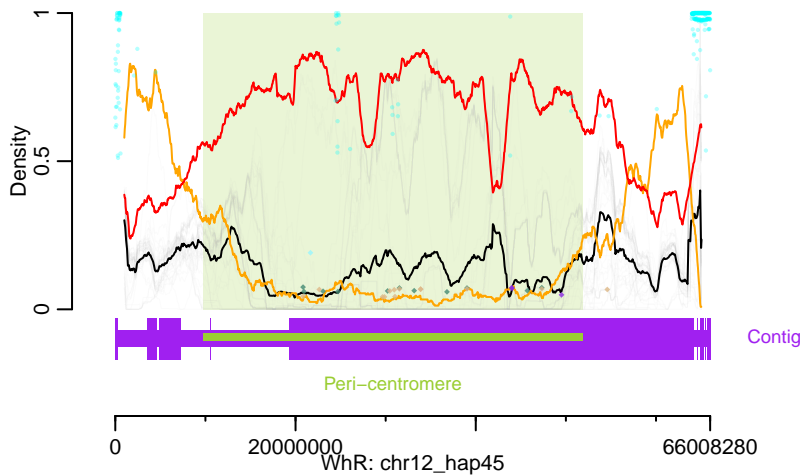

Supplement: Supplementary file 5 — Supplementary Figs. 3–14, 20, 21, 23–27 and 34–44. [file 41586_2025_8843_MOESM5_ESM.zip › suppl_figure_3_to_14/suppl_figure_14_chr12/suppl_figure_14aa_45_A_WhR.pdf]

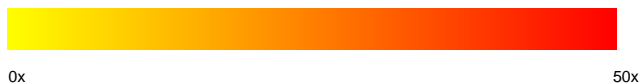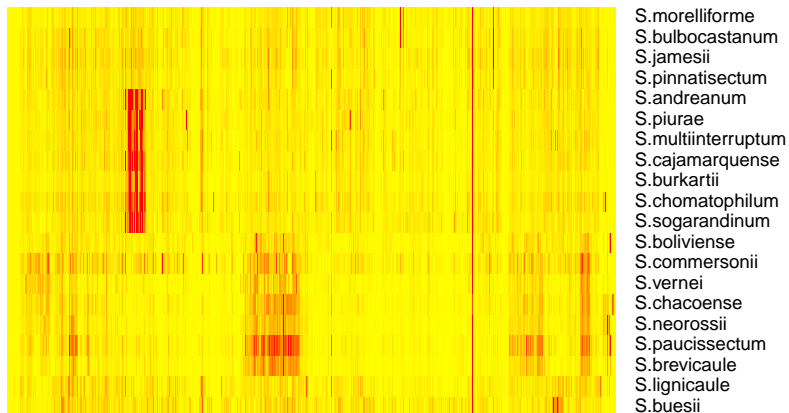

— TE — Gene — Non-aln • TanRep ♦ CenRep ■ rDNA

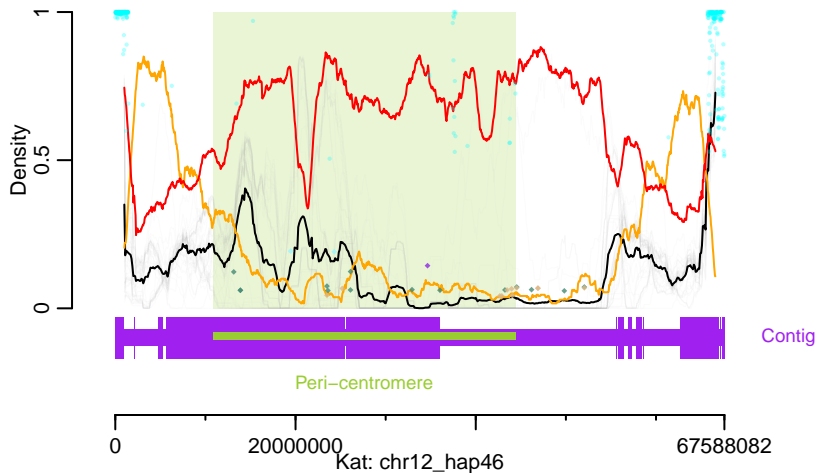

Supplement: Supplementary file 5 — Supplementary Figs. 3–14, 20, 21, 23–27 and 34–44. [file 41586_2025_8843_MOESM5_ESM.zip › suppl_figure_3_to_14/suppl_figure_14_chr12/suppl_figure_14ar_46_H_Kat.pdf]

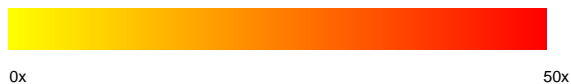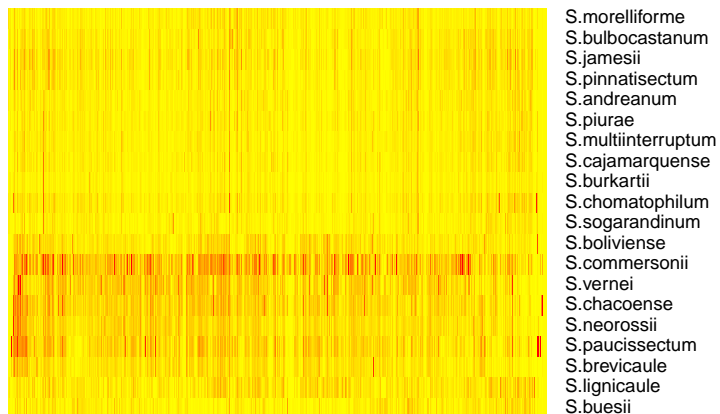

— TE — Gene — Non-aln • TanRep ♦ CenRep ■ rDNA

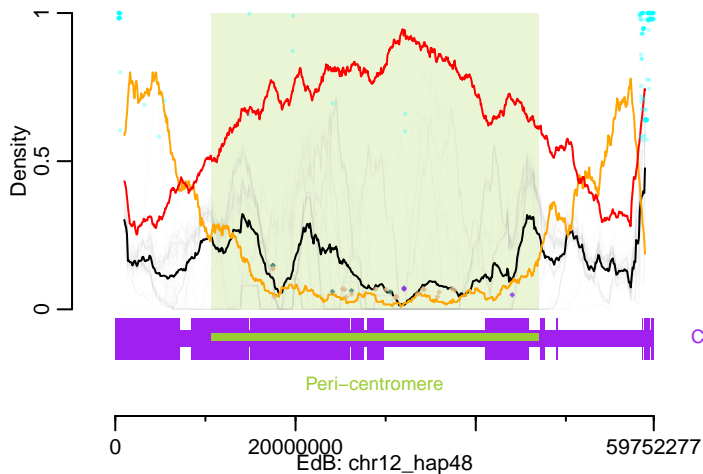

Supplement: Supplementary file 5 — Supplementary Figs. 3–14, 20, 21, 23–27 and 34–44. [file 41586_2025_8843_MOESM5_ESM.zip › suppl_figure_3_to_14/suppl_figure_14_chr12/suppl_figure_14bn_48_J_EdB.pdf]

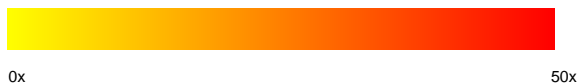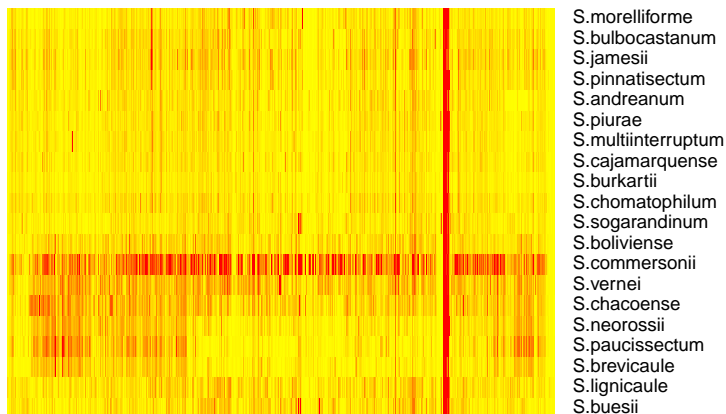

— TE — Gene — Non-aln • TanRep ♦ CenRep ■ rDNA

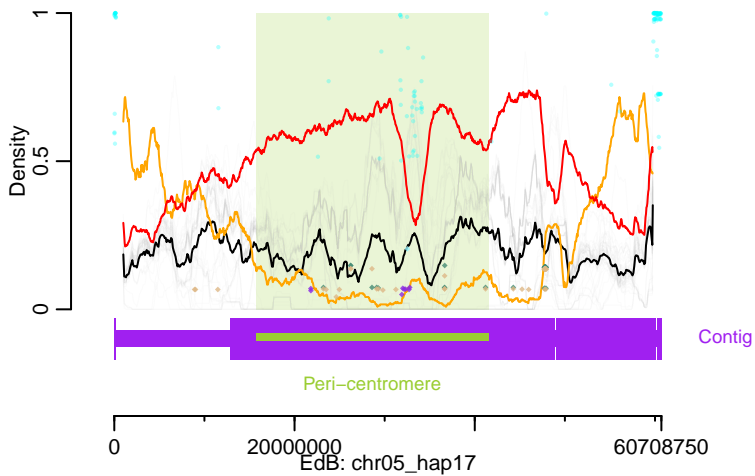

Supplement: Supplementary file 5 — Supplementary Figs. 3–14, 20, 21, 23–27 and 34–44. [file 41586_2025_8843_MOESM5_ESM.zip › suppl_figure_3_to_14/suppl_figure_7_chr05/suppl_figure_7aj_17_J_EdB.pdf]

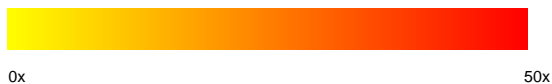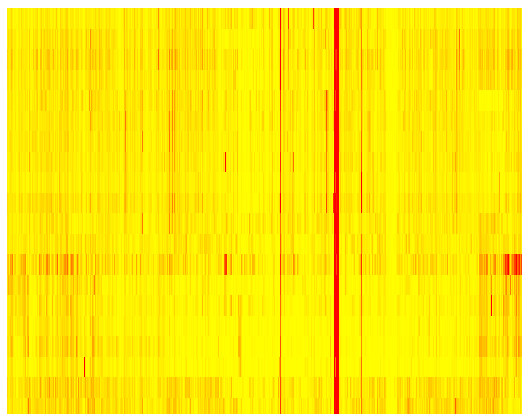

— TE — Gene — Non-aln ● TanRep ♦ CenRep ■ rDNA

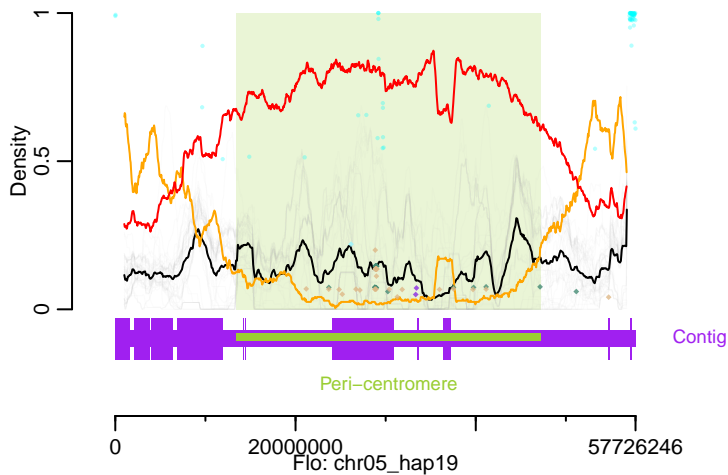

Supplement: Supplementary file 5 — Supplementary Figs. 3–14, 20, 21, 23–27 and 34–44. [file 41586_2025_8843_MOESM5_ESM.zip › suppl_figure_3_to_14/suppl_figure_7_chr05/suppl_figure_7ay_19_E_Flo.pdf]

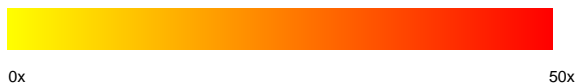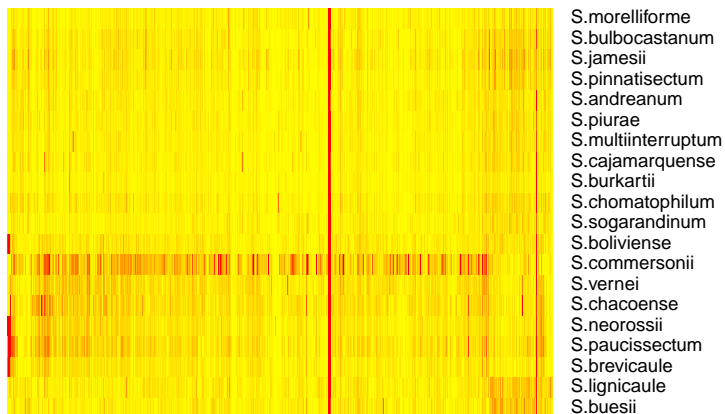

— TE — Gene — Non-aln • TanRep ♦ CenRep ■ rDNA

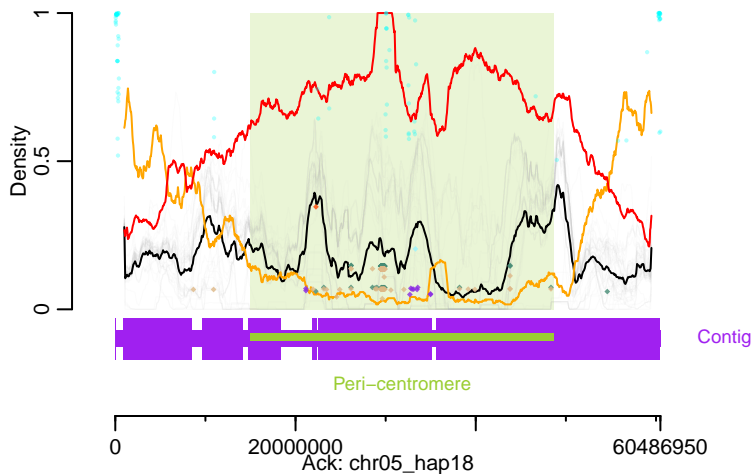

Supplement: Supplementary file 5 — Supplementary Figs. 3–14, 20, 21, 23–27 and 34–44. [file 41586_2025_8843_MOESM5_ESM.zip › suppl_figure_3_to_14/suppl_figure_7_chr05/suppl_figure_7ap_18_F_Ack.pdf]

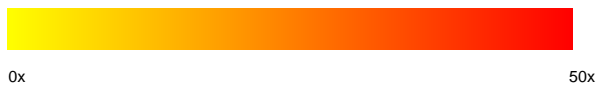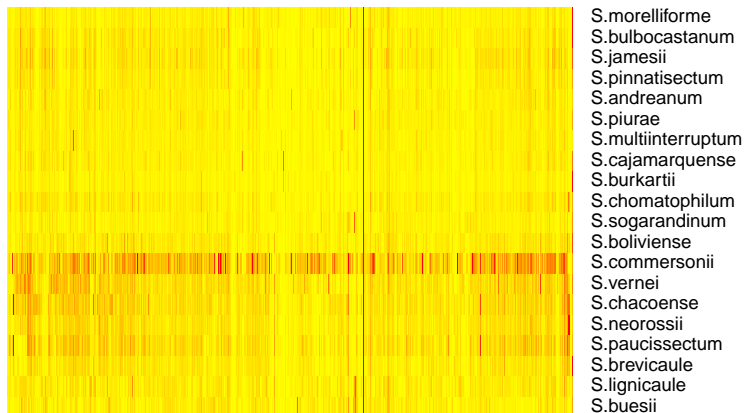

— TE — Gene — Non-aln • TanRep ♦ CenRep ■ rDNA

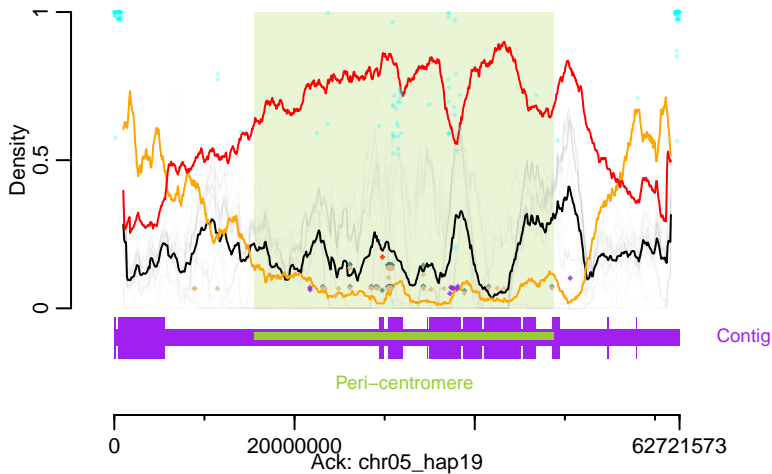

Supplement: Supplementary file 5 — Supplementary Figs. 3–14, 20, 21, 23–27 and 34–44. [file 41586_2025_8843_MOESM5_ESM.zip › suppl_figure_3_to_14/suppl_figure_7_chr05/suppl_figure_7az_19_F_Ack.pdf]

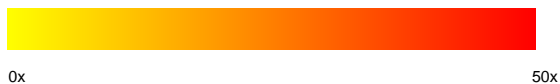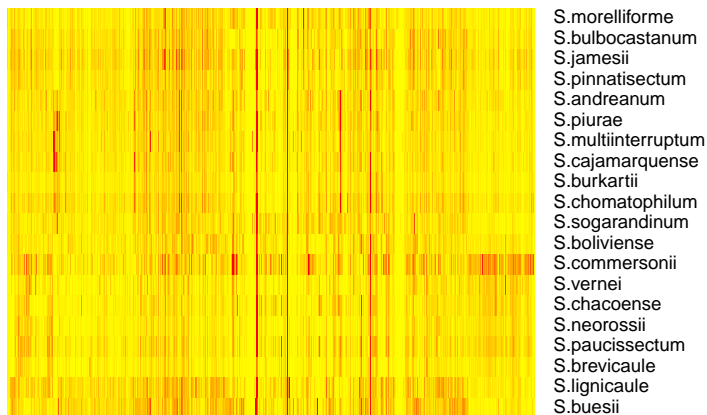

TE Gene Non-aln TanRep CenRep rDNA

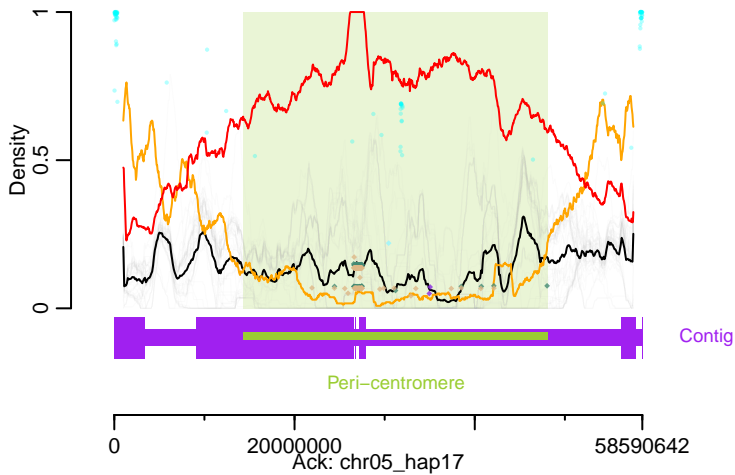

Supplement: Supplementary file 5 — Supplementary Figs. 3–14, 20, 21, 23–27 and 34–44. [file 41586_2025_8843_MOESM5_ESM.zip › suppl_figure_3_to_14/suppl_figure_7_chr05/suppl_figure_7af_17_F_Ack.pdf]

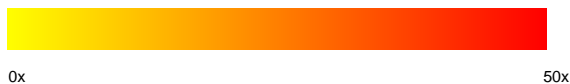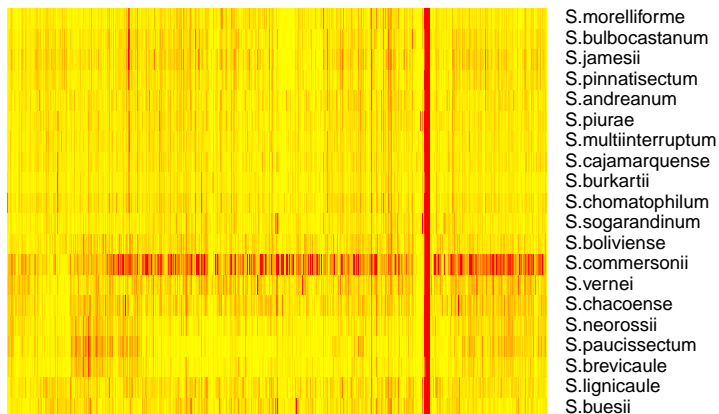

— TE — Gene — Non-aln ● TanRep ♦ CenRep ■ rDNA

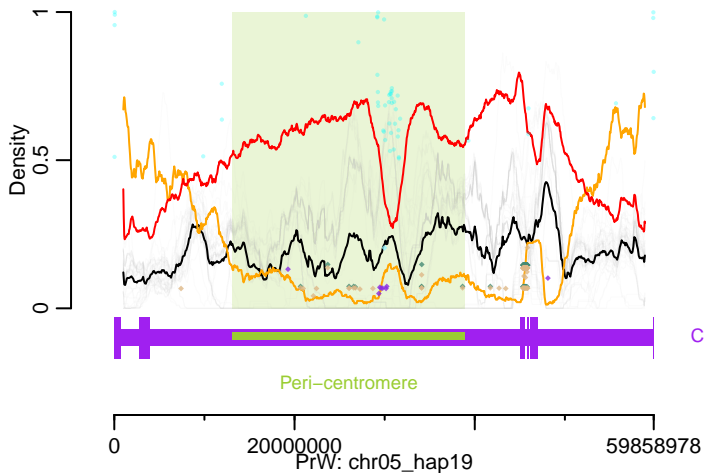

Supplement: Supplementary file 5 — Supplementary Figs. 3–14, 20, 21, 23–27 and 34–44. [file 41586_2025_8843_MOESM5_ESM.zip › suppl_figure_3_to_14/suppl_figure_7_chr05/suppl_figure_7ax_19_D_PrW.pdf]

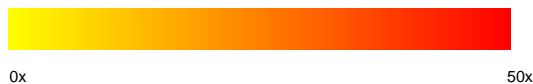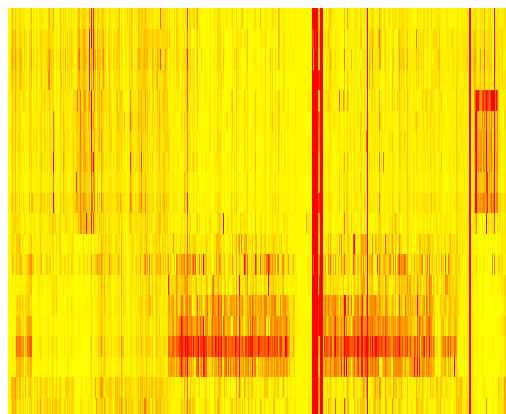

— TE — Gene — Non-aln ● TanRep ♦ CenRep ■ rDNA

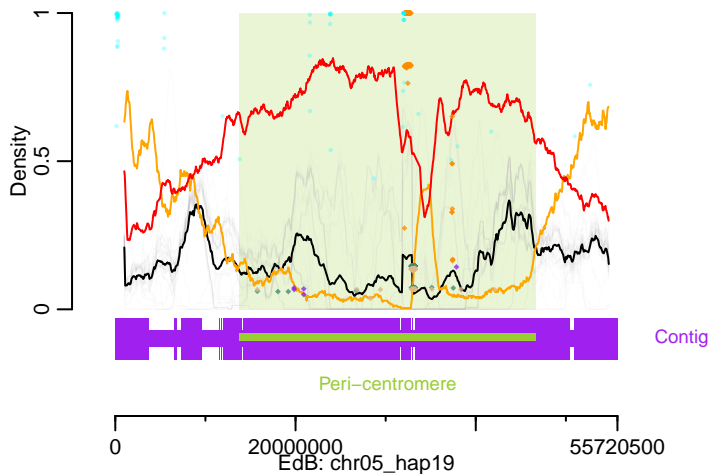

Supplement: Supplementary file 5 — Supplementary Figs. 3–14, 20, 21, 23–27 and 34–44. [file 41586_2025_8843_MOESM5_ESM.zip › suppl_figure_3_to_14/suppl_figure_7_chr05/suppl_figure_7bd_19_J_EdB.pdf]

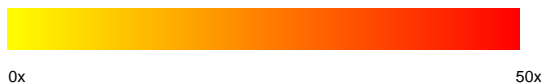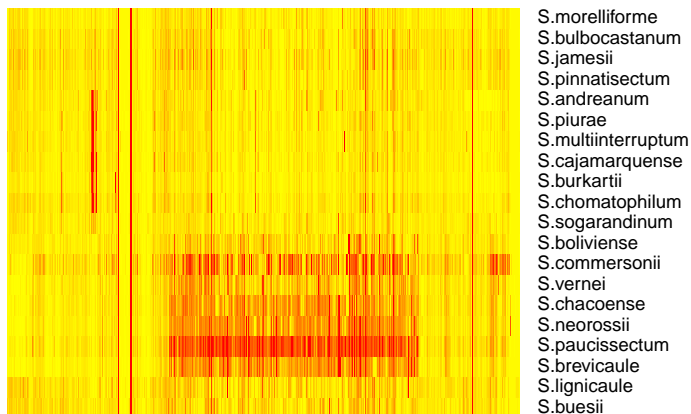

— TE — Gene — Non-aln • TanRep ♦ CenRep ■ rDNA

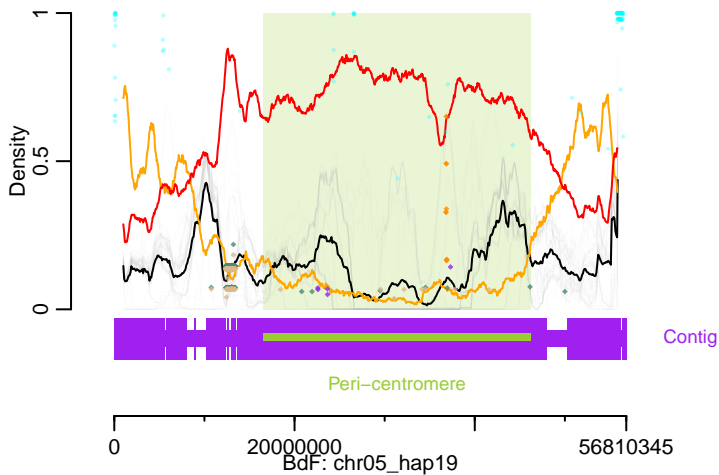

Supplement: Supplementary file 5 — Supplementary Figs. 3–14, 20, 21, 23–27 and 34–44. [file 41586_2025_8843_MOESM5_ESM.zip › suppl_figure_3_to_14/suppl_figure_7_chr05/suppl_figure_7av_19_B_BdF.pdf]

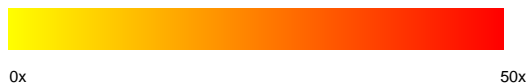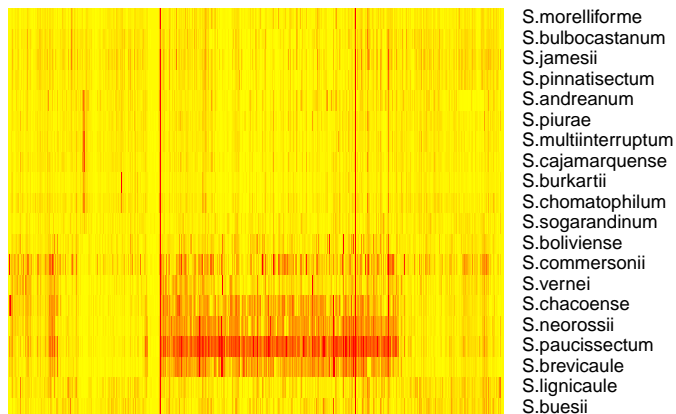

— TE — Gene — Non-aln • TanRep ♦ CenRep ■ rDNA

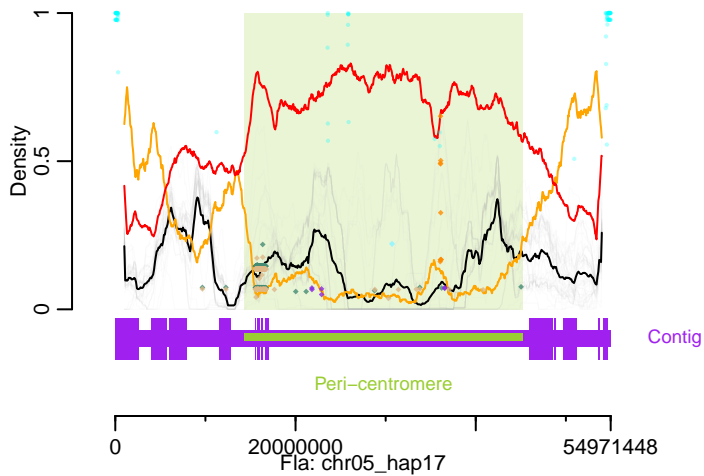

Supplement: Supplementary file 5 — Supplementary Figs. 3–14, 20, 21, 23–27 and 34–44. [file 41586_2025_8843_MOESM5_ESM.zip › suppl_figure_3_to_14/suppl_figure_7_chr05/suppl_figure_7ag_17_G_Fla.pdf]

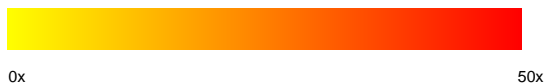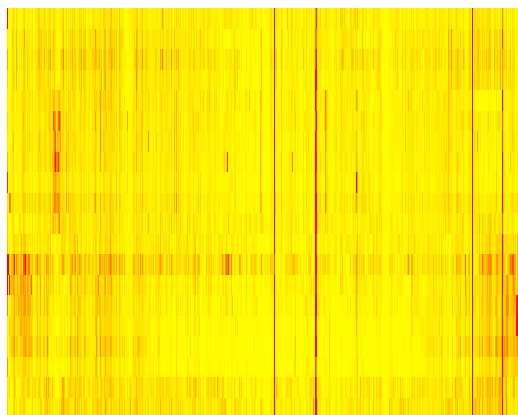

— TE — Gene — Non-aln • TanRep ♦ CenRep ■ rDNA

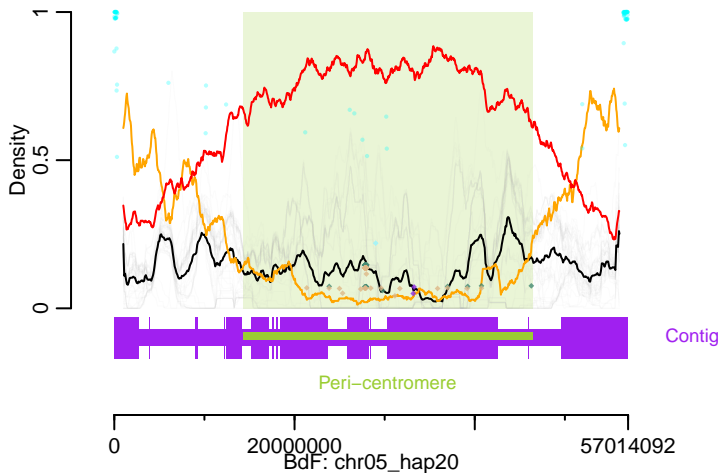

Supplement: Supplementary file 5 — Supplementary Figs. 3–14, 20, 21, 23–27 and 34–44. [file 41586_2025_8843_MOESM5_ESM.zip › suppl_figure_3_to_14/suppl_figure_7_chr05/suppl_figure_7bf_20_B_BdF.pdf]

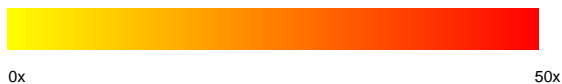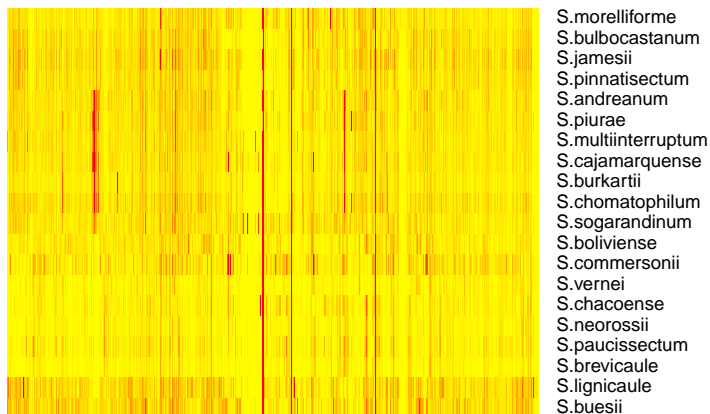

— TE — Gene — Non-aln • TanRep ♦ CenRep ■ rDNA

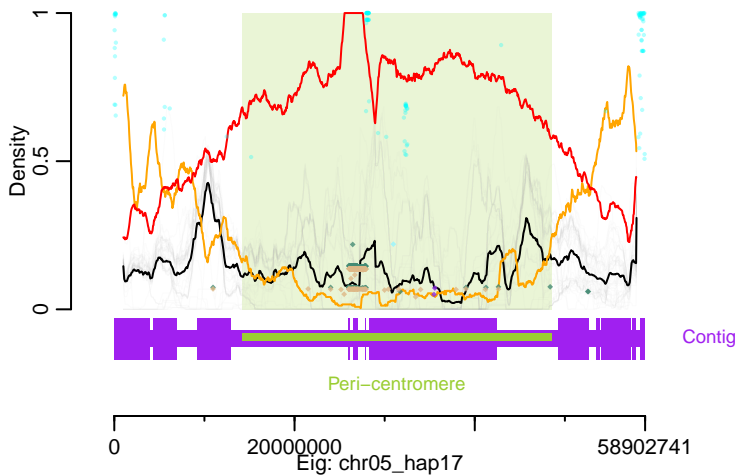

Supplement: Supplementary file 5 — Supplementary Figs. 3–14, 20, 21, 23–27 and 34–44. [file 41586_2025_8843_MOESM5_ESM.zip › suppl_figure_3_to_14/suppl_figure_7_chr05/suppl_figure_7ac_17_C_EgH.pdf]

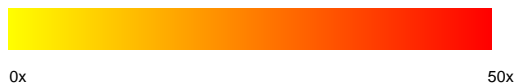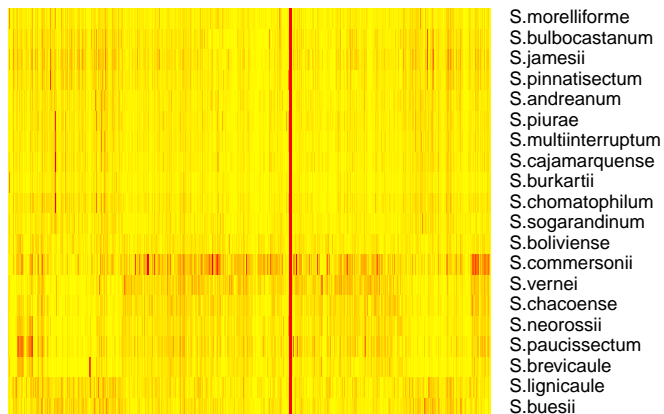

— TE — Gene — Non-aln • TanRep ♦ CenRep ■ rDNA

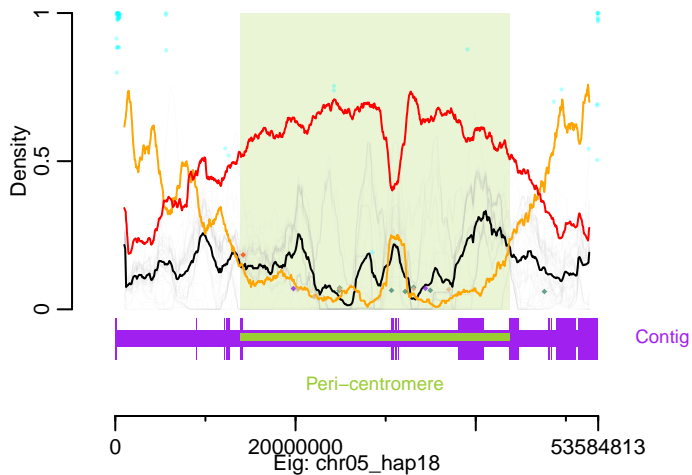

Supplement: Supplementary file 5 — Supplementary Figs. 3–14, 20, 21, 23–27 and 34–44. [file 41586_2025_8843_MOESM5_ESM.zip › suppl_figure_3_to_14/suppl_figure_7_chr05/suppl_figure_7am_18_C_EgH.pdf]

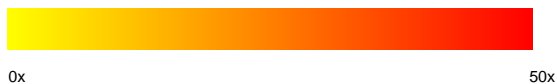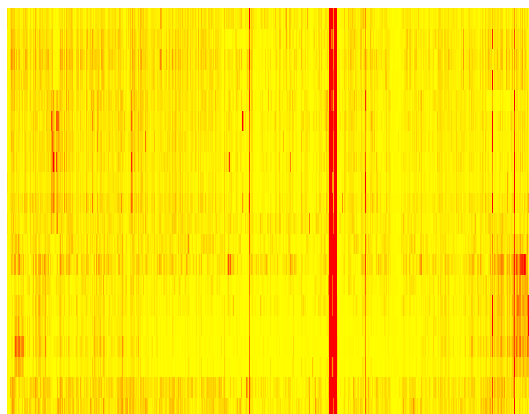

— TE — Gene — Non-aln • TanRep ♦ CenRep ■ rDNA

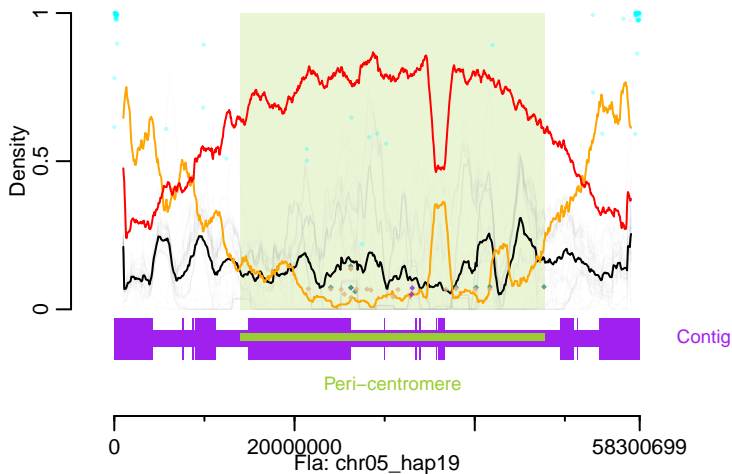

Supplement: Supplementary file 5 — Supplementary Figs. 3–14, 20, 21, 23–27 and 34–44. [file 41586_2025_8843_MOESM5_ESM.zip › suppl_figure_3_to_14/suppl_figure_7_chr05/suppl_figure_7ba_19_G_Fla.pdf]

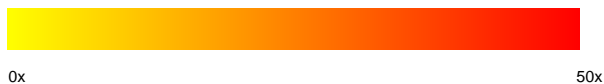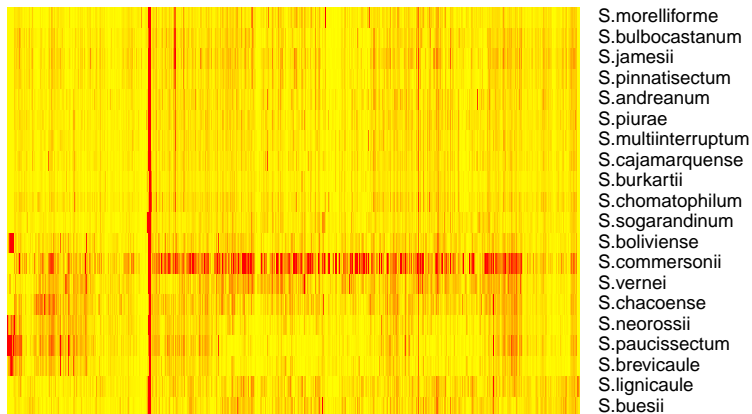

— TE — Gene — Non-aln • TanRep ♦ CenRep ■ rDNA

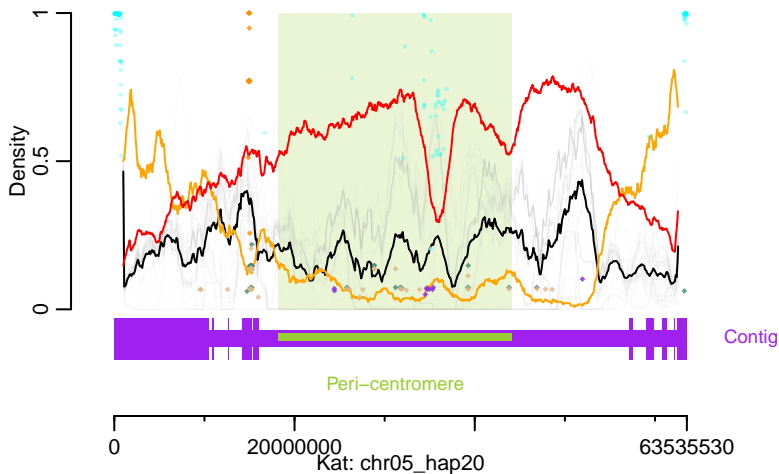

Supplement: Supplementary file 5 — Supplementary Figs. 3–14, 20, 21, 23–27 and 34–44. [file 41586_2025_8843_MOESM5_ESM.zip › suppl_figure_3_to_14/suppl_figure_7_chr05/suppl_figure_7bl_20_H_Kat.pdf]

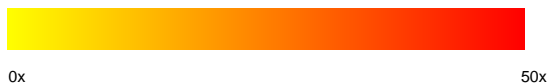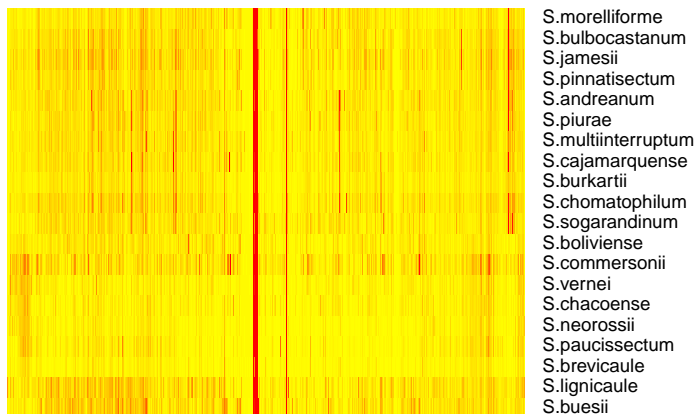

— TE — Gene — Non-aln • TanRep ♦ CenRep ■ rDNA

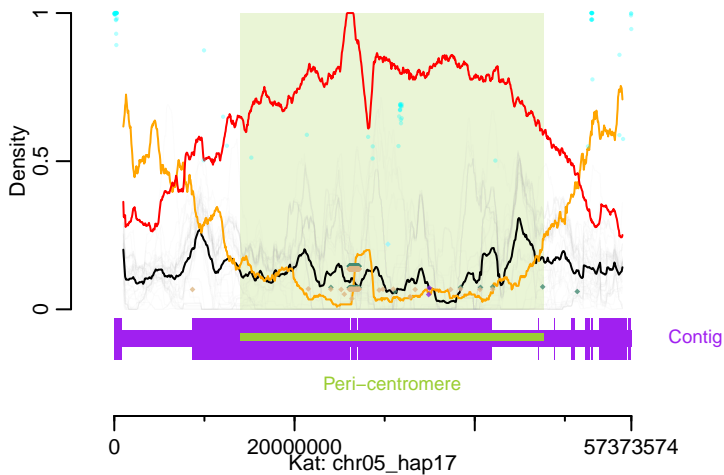

Supplement: Supplementary file 5 — Supplementary Figs. 3–14, 20, 21, 23–27 and 34–44. [file 41586_2025_8843_MOESM5_ESM.zip › suppl_figure_3_to_14/suppl_figure_7_chr05/suppl_figure_7ah_17_H_Kat.pdf]

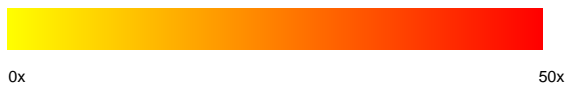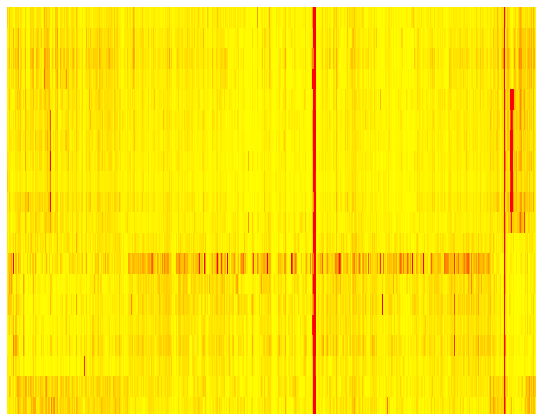

— TE — Gene — Non-aln • TanRep ♦ CenRep ■ rDNA

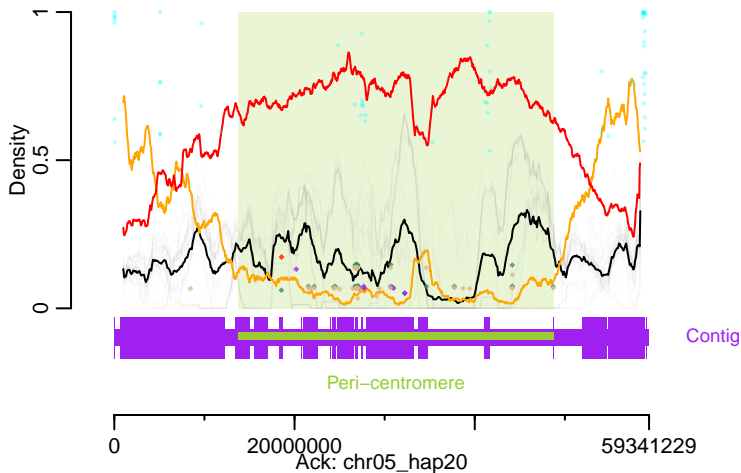

Supplement: Supplementary file 5 — Supplementary Figs. 3–14, 20, 21, 23–27 and 34–44. [file 41586_2025_8843_MOESM5_ESM.zip › suppl_figure_3_to_14/suppl_figure_7_chr05/suppl_figure_7bj_20_F_Ack.pdf]

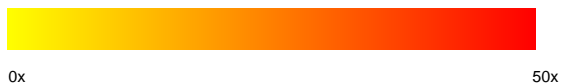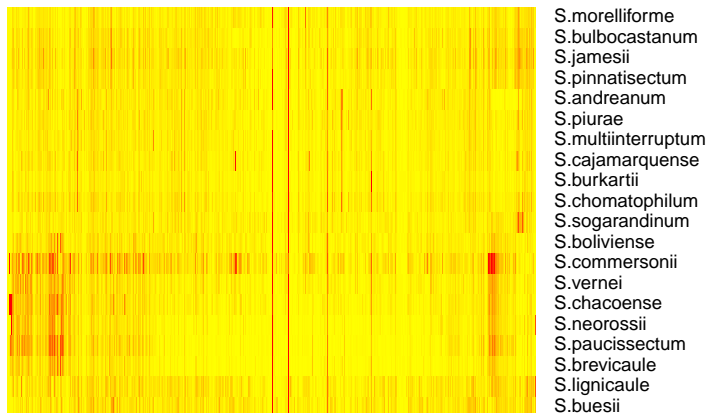

— TE — Gene — Non-aln ● TanRep ♦ CenRep ■ rDNA

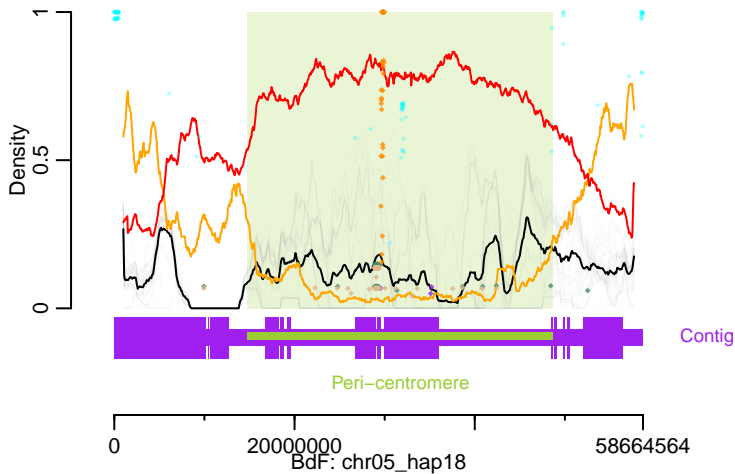

Supplement: Supplementary file 5 — Supplementary Figs. 3–14, 20, 21, 23–27 and 34–44. [file 41586_2025_8843_MOESM5_ESM.zip › suppl_figure_3_to_14/suppl_figure_7_chr05/suppl_figure_7al_18_B_BdF.pdf]

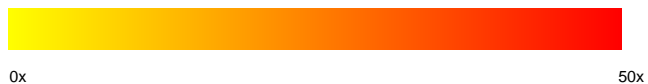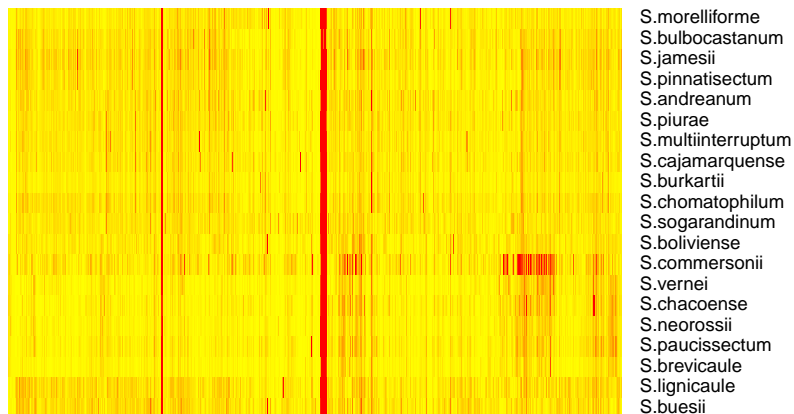

— TE — Gene — Non-aln • TanRep ♦ CenRep ■ rDNA

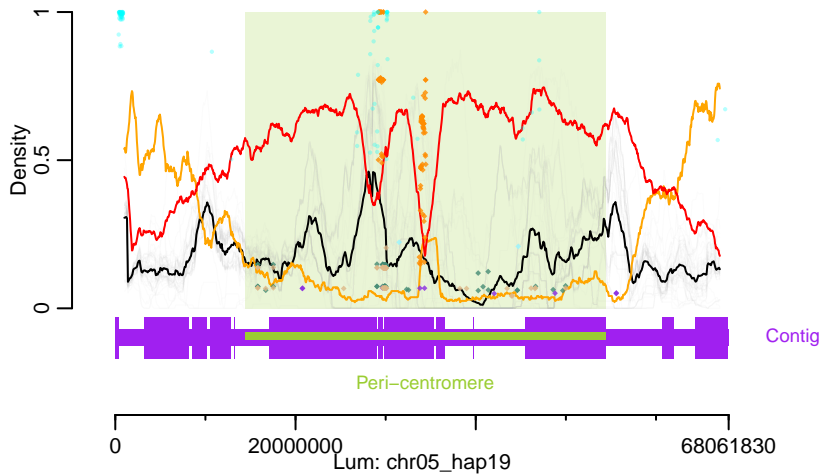

Supplement: Supplementary file 5 — Supplementary Figs. 3–14, 20, 21, 23–27 and 34–44. [file 41586_2025_8843_MOESM5_ESM.zip › suppl_figure_3_to_14/suppl_figure_7_chr05/suppl_figure_7bc_19_I_Lum.pdf]

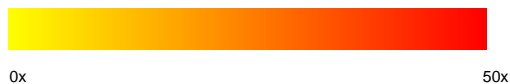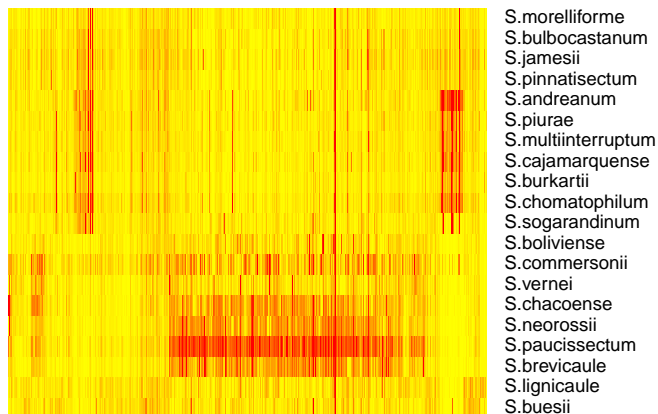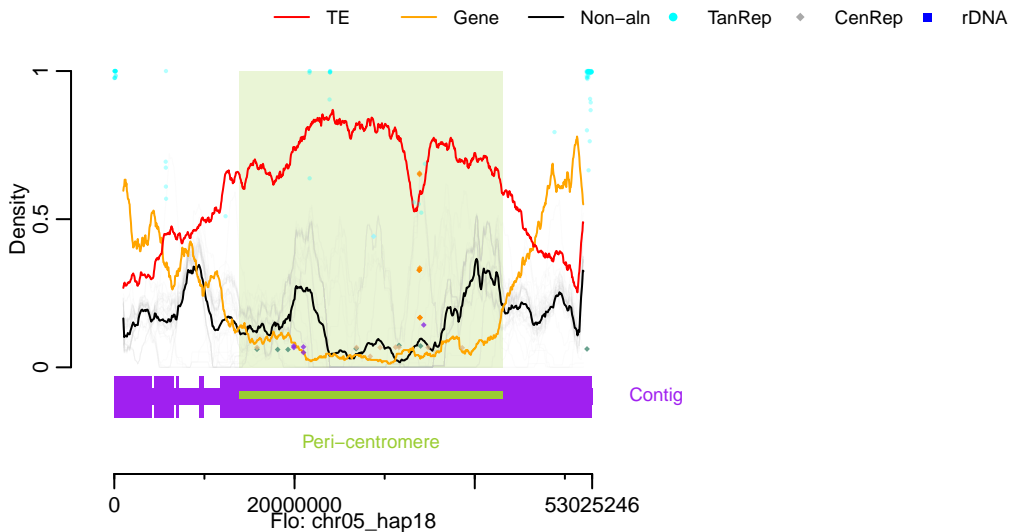

Supplement: Supplementary file 5 — Supplementary Figs. 3–14, 20, 21, 23–27 and 34–44. [file 41586_2025_8843_MOESM5_ESM.zip › suppl_figure_3_to_14/suppl_figure_7_chr05/suppl_figure_7ao_18_E_Flo.pdf]
