# Supplementary figures and images for: The phased pan-genome of tetraploid European potato (part 1 of 2)
Source: Nature. 2025 Apr 16;642(8067):389–97. doi: 10.1038/s41586-025-08843-0 (PMC12158759; doi:10.1038/s41586-025-08843-0)

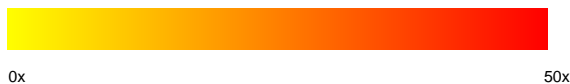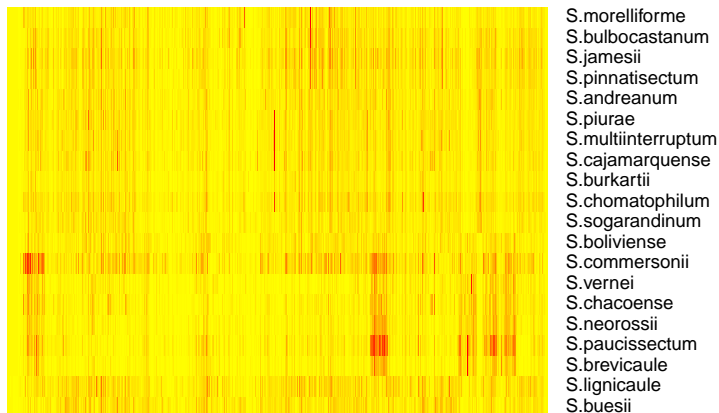

— TE — Gene — Non-aln • TanRep ♦ CenRep ■ rDNA

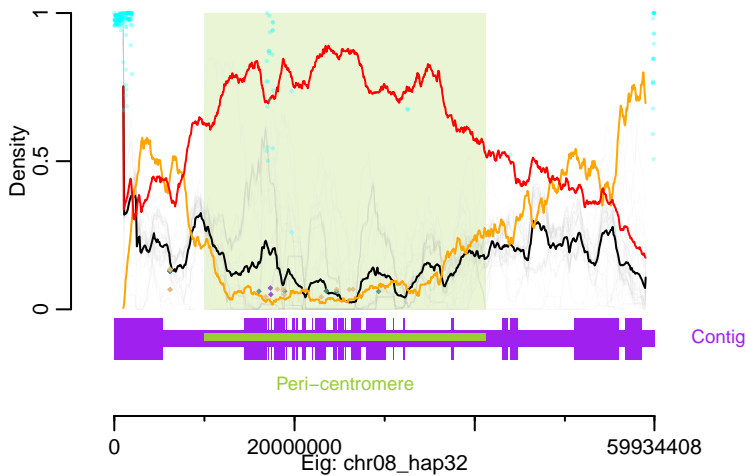

Supplement: Supplementary file 5 — Supplementary Figs. 3–14, 20, 21, 23–27 and 34–44. [file 41586_2025_8843_MOESM5_ESM.zip › suppl_figure_3_to_14/suppl_figure_10_chr08/suppl_figure_10bg_32_C_EgH.pdf]

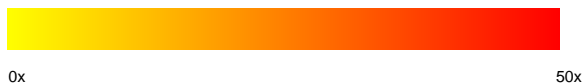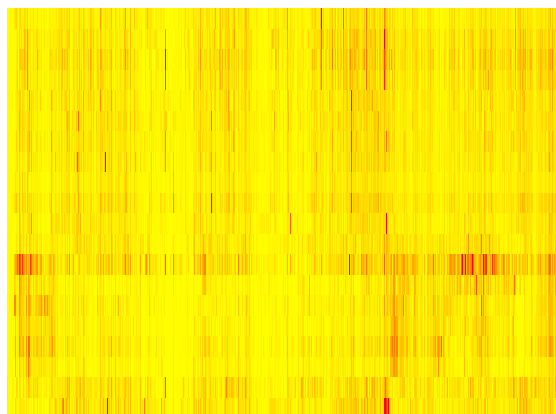

— TE — Gene — Non-aln • TanRep ♦ CenRep ■ rDNA

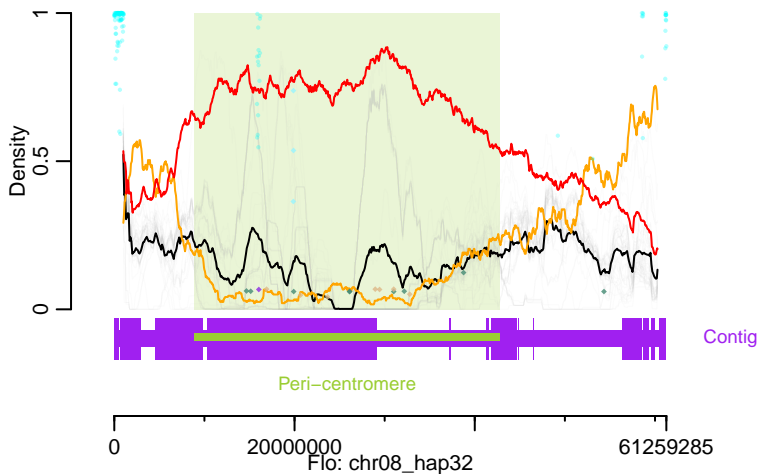

Supplement: Supplementary file 5 — Supplementary Figs. 3–14, 20, 21, 23–27 and 34–44. [file 41586_2025_8843_MOESM5_ESM.zip › suppl_figure_3_to_14/suppl_figure_10_chr08/suppl_figure_10bi_32_E_Flo.pdf]

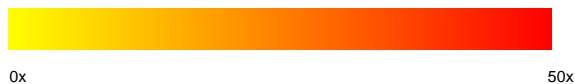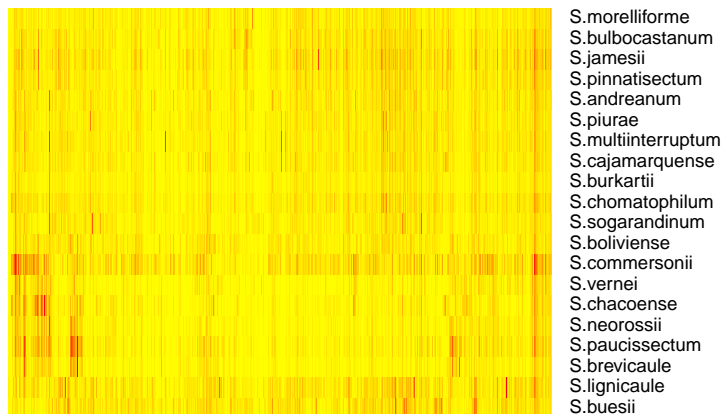

— TE — Gene — Non-aln • TanRep ♦ CenRep ■ rDNA

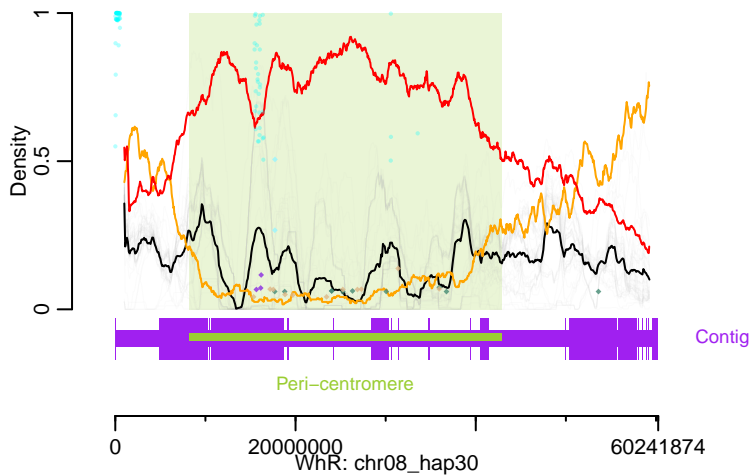

Supplement: Supplementary file 5 — Supplementary Figs. 3–14, 20, 21, 23–27 and 34–44. [file 41586_2025_8843_MOESM5_ESM.zip › suppl_figure_3_to_14/suppl_figure_10_chr08/suppl_figure_10ak_30_A_WhR.pdf]

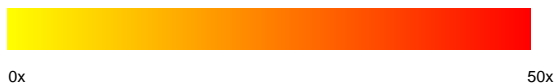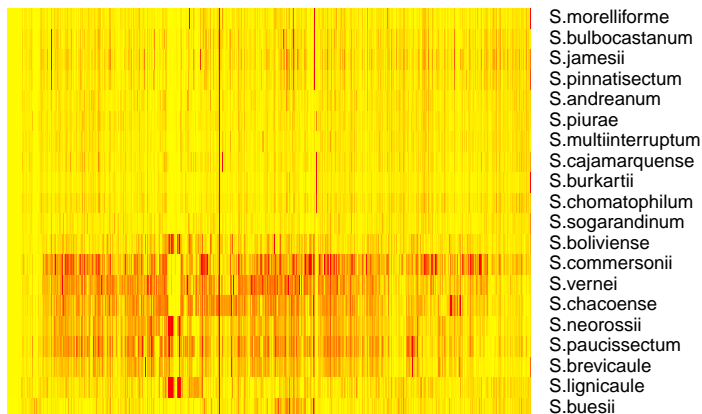

— TE — Gene — Non-aln • TanRep ♦ CenRep ■ rDNA

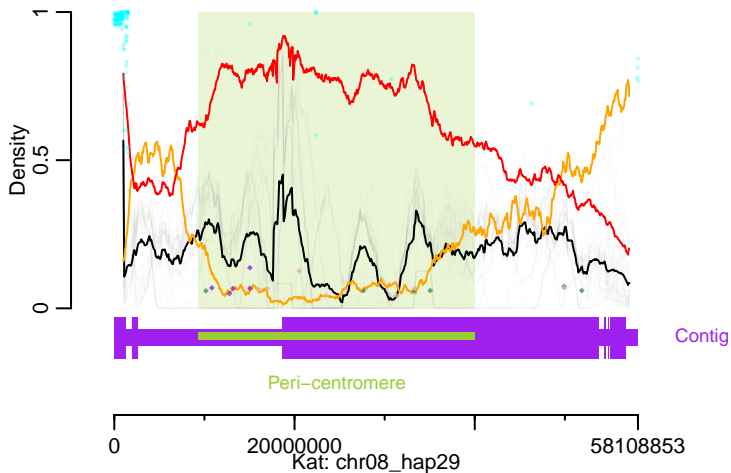

Supplement: Supplementary file 5 — Supplementary Figs. 3–14, 20, 21, 23–27 and 34–44. [file 41586_2025_8843_MOESM5_ESM.zip › suppl_figure_3_to_14/suppl_figure_10_chr08/suppl_figure_10ah_29_H_Kat.pdf]

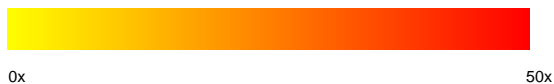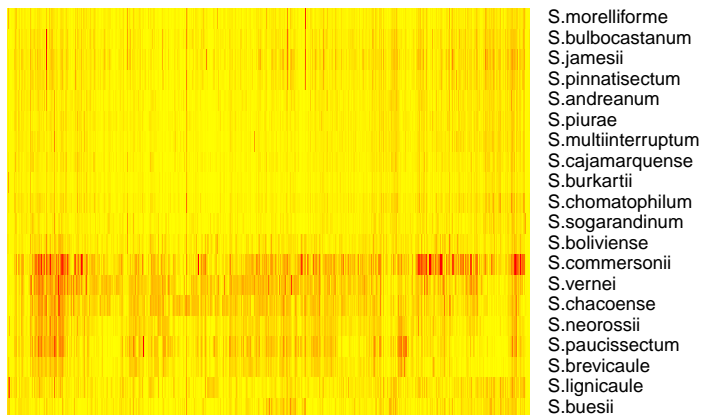

— TE — Gene — Non-aln ● TanRep ♦ CenRep ■ rDNA

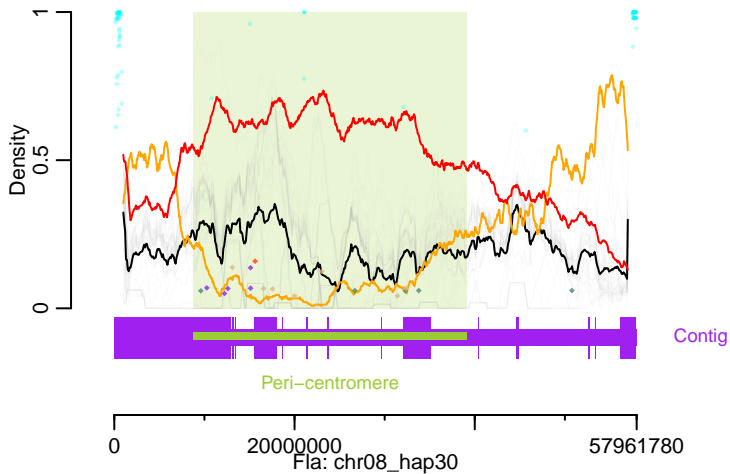

Supplement: Supplementary file 5 — Supplementary Figs. 3–14, 20, 21, 23–27 and 34–44. [file 41586_2025_8843_MOESM5_ESM.zip › suppl_figure_3_to_14/suppl_figure_10_chr08/suppl_figure_10aq_30_G_Fla.pdf]

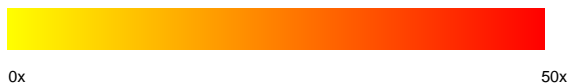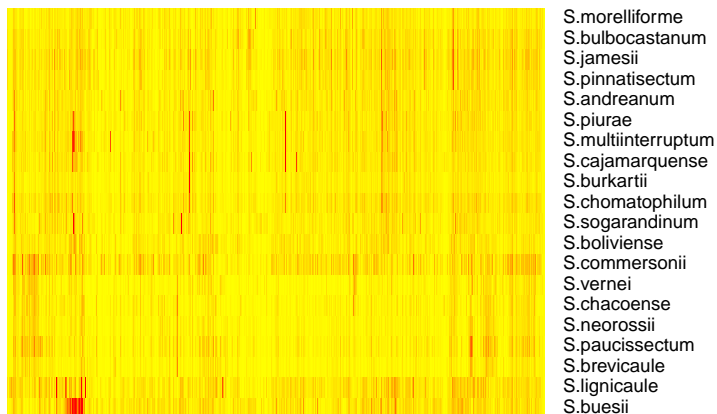

— TE — Gene — Non-aln • TanRep ♦ CenRep ■ rDNA

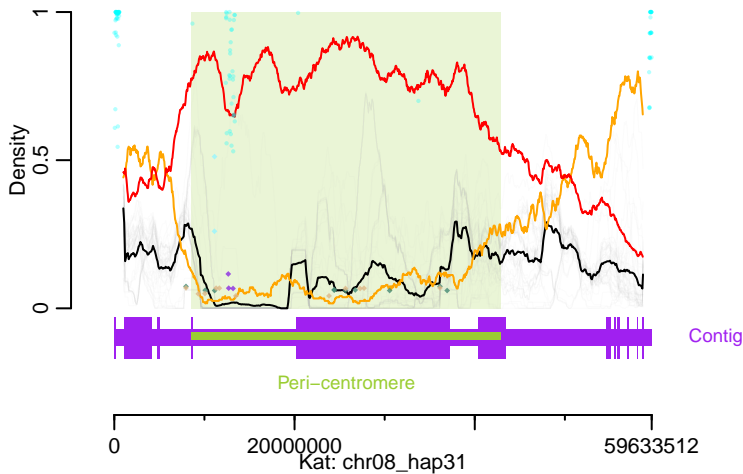

Supplement: Supplementary file 5 — Supplementary Figs. 3–14, 20, 21, 23–27 and 34–44. [file 41586_2025_8843_MOESM5_ESM.zip › suppl_figure_3_to_14/suppl_figure_10_chr08/suppl_figure_10bb_31_H_Kat.pdf]

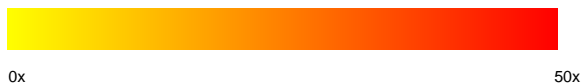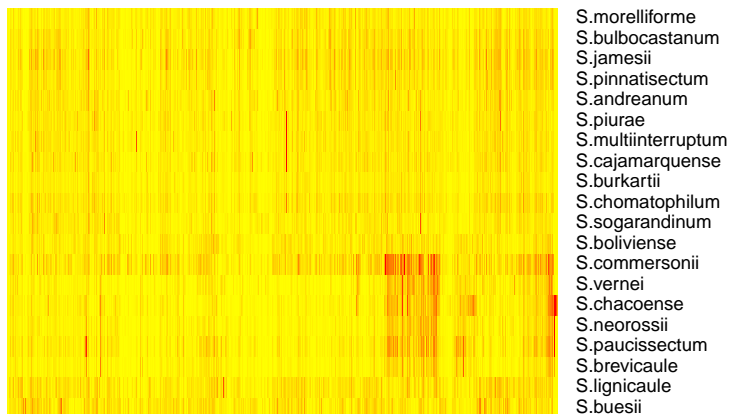

— TE — Gene — Non-aln • TanRep ♦ CenRep ■ rDNA

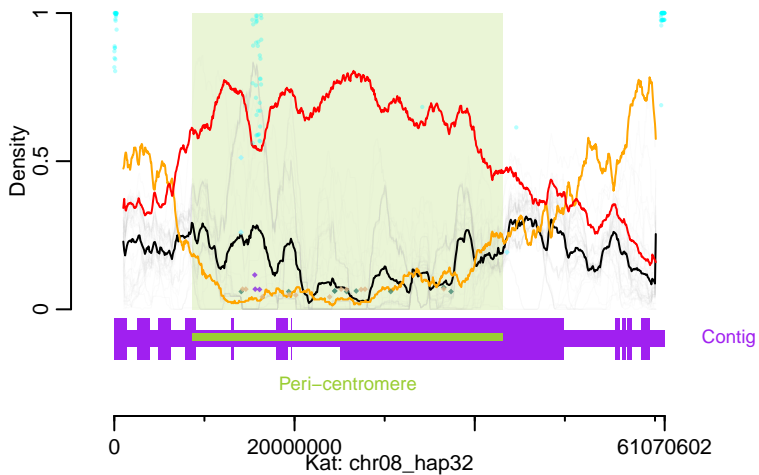

Supplement: Supplementary file 5 — Supplementary Figs. 3–14, 20, 21, 23–27 and 34–44. [file 41586_2025_8843_MOESM5_ESM.zip › suppl_figure_3_to_14/suppl_figure_10_chr08/suppl_figure_10bl_32_H_Kat.pdf]

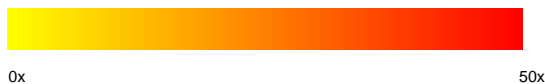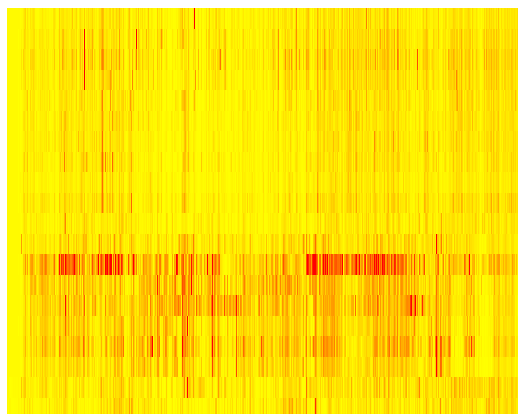

— TE — Gene — Non-aln • TanRep ♦ CenRep ■ rDNA

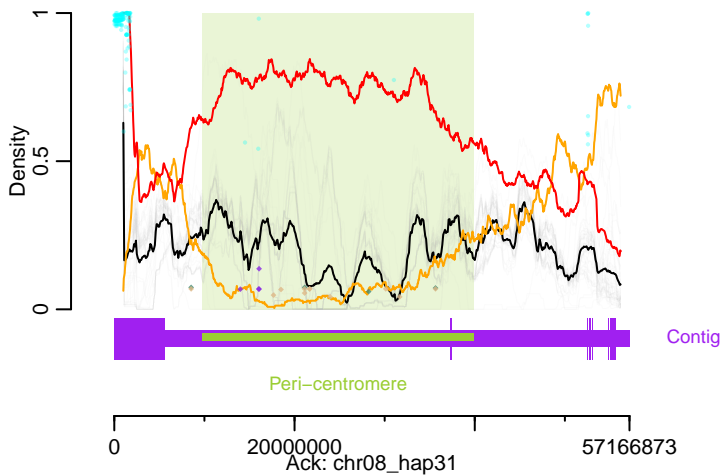

Supplement: Supplementary file 5 — Supplementary Figs. 3–14, 20, 21, 23–27 and 34–44. [file 41586_2025_8843_MOESM5_ESM.zip › suppl_figure_3_to_14/suppl_figure_10_chr08/suppl_figure_10az_31_F_Ack.pdf]

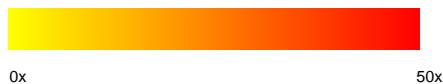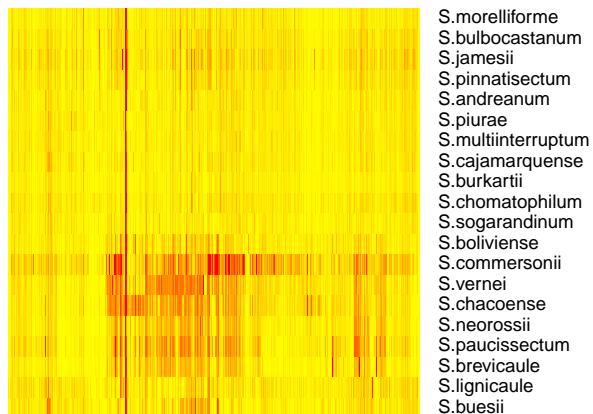

— TE — Gene — Non-aln • TanRep ♦ CenRep ■ rDNA

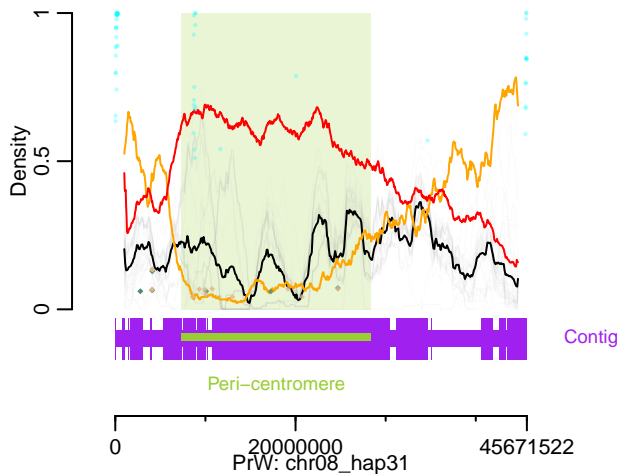

Supplement: Supplementary file 5 — Supplementary Figs. 3–14, 20, 21, 23–27 and 34–44. [file 41586_2025_8843_MOESM5_ESM.zip › suppl_figure_3_to_14/suppl_figure_10_chr08/suppl_figure_10ax_31_D_PrW.pdf]

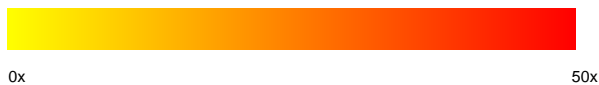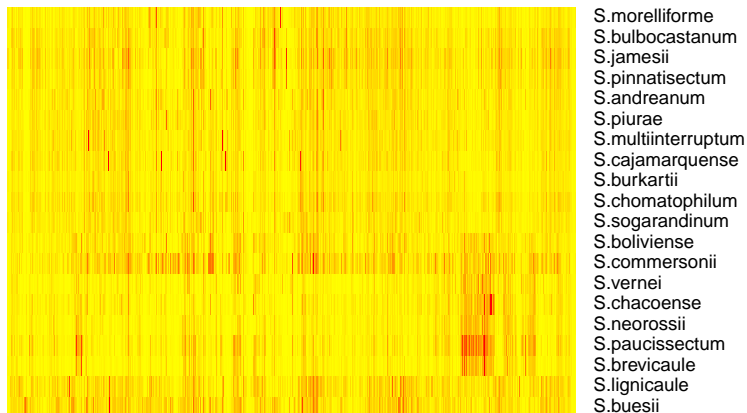

— TE — Gene — Non-aln • TanRep ♦ CenRep ■ rDNA

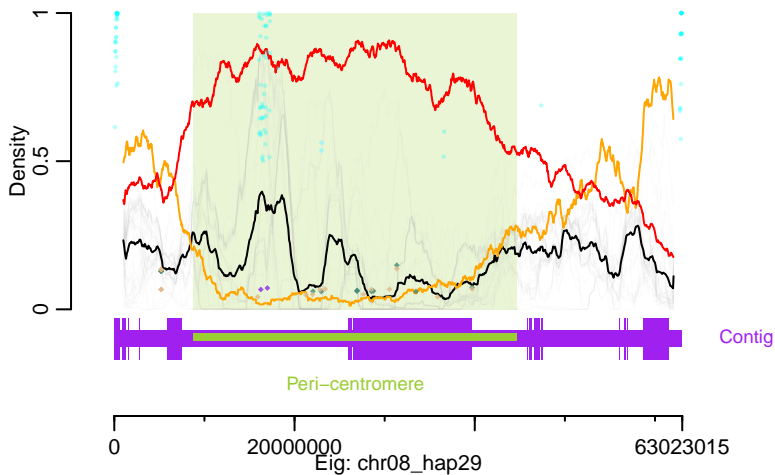

Supplement: Supplementary file 5 — Supplementary Figs. 3–14, 20, 21, 23–27 and 34–44. [file 41586_2025_8843_MOESM5_ESM.zip › suppl_figure_3_to_14/suppl_figure_10_chr08/suppl_figure_10ac_29_C_EgH.pdf]

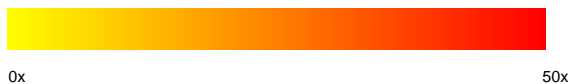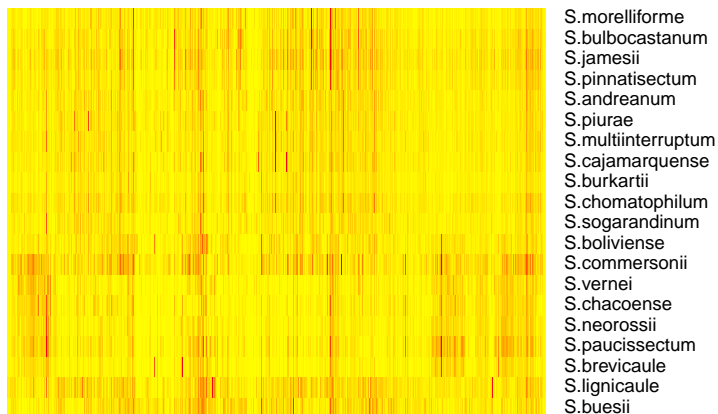

— TE — Gene — Non-aln • TanRep ♦ CenRep ■ rDNA

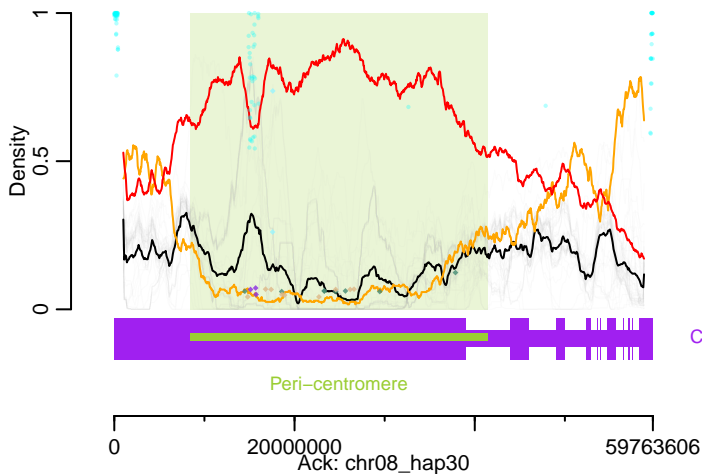

Supplement: Supplementary file 5 — Supplementary Figs. 3–14, 20, 21, 23–27 and 34–44. [file 41586_2025_8843_MOESM5_ESM.zip › suppl_figure_3_to_14/suppl_figure_10_chr08/suppl_figure_10ap_30_F_Ack.pdf]

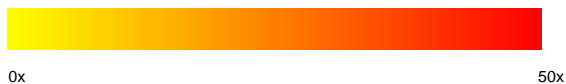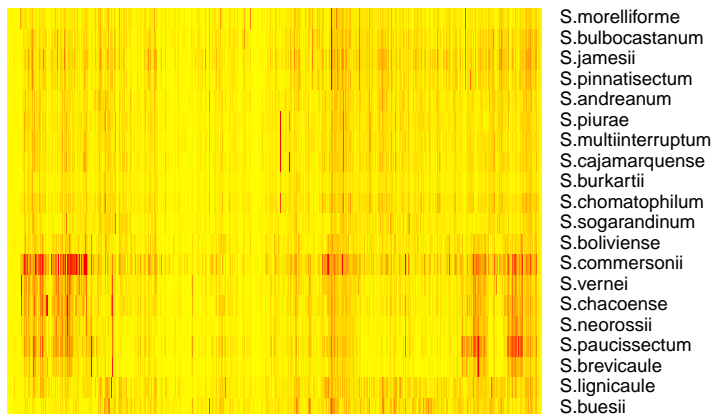

— TE — Gene — Non-aln • TanRep ♦ CenRep ■ rDNA

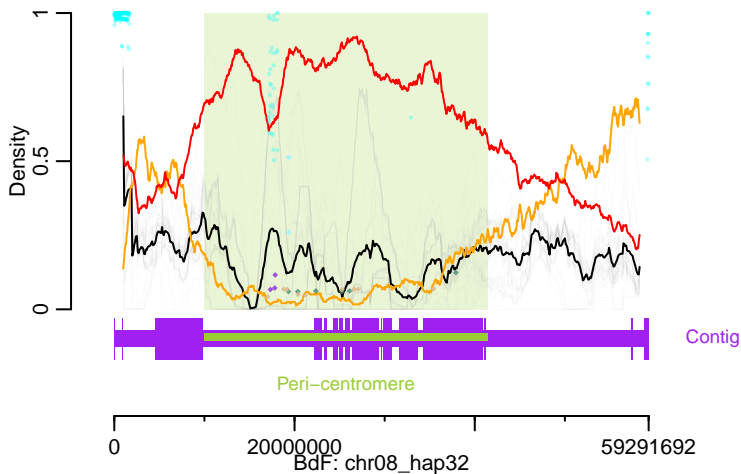

Supplement: Supplementary file 5 — Supplementary Figs. 3–14, 20, 21, 23–27 and 34–44. [file 41586_2025_8843_MOESM5_ESM.zip › suppl_figure_3_to_14/suppl_figure_10_chr08/suppl_figure_10bf_32_B_BdF.pdf]

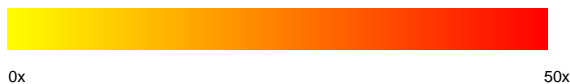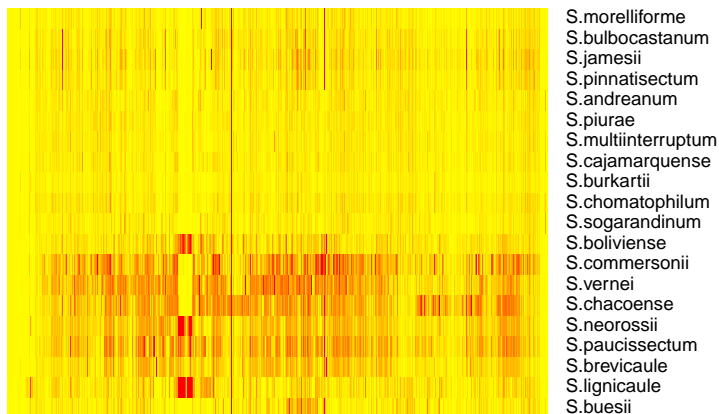

— TE — Gene — Non-aln • TanRep ♦ CenRep ■ rDNA

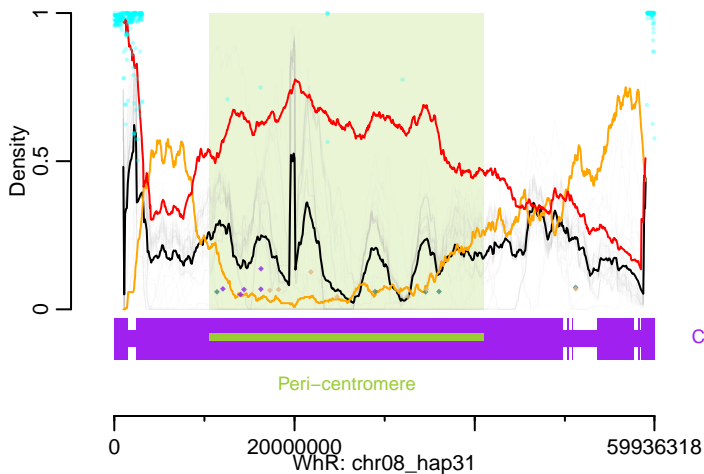

Supplement: Supplementary file 5 — Supplementary Figs. 3–14, 20, 21, 23–27 and 34–44. [file 41586_2025_8843_MOESM5_ESM.zip › suppl_figure_3_to_14/suppl_figure_10_chr08/suppl_figure_10aw_31_A_WhR.pdf]

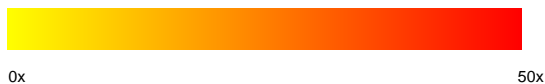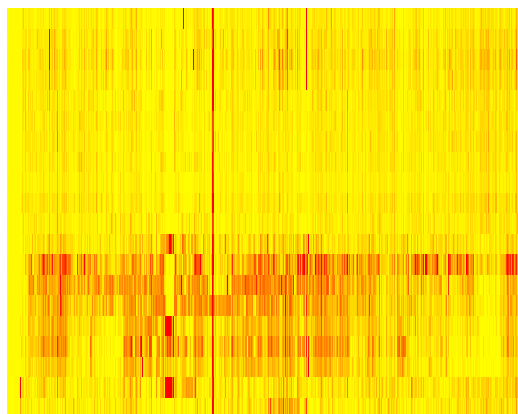

— TE — Gene — Non-aln • TanRep ♦ CenRep ■ rDNA

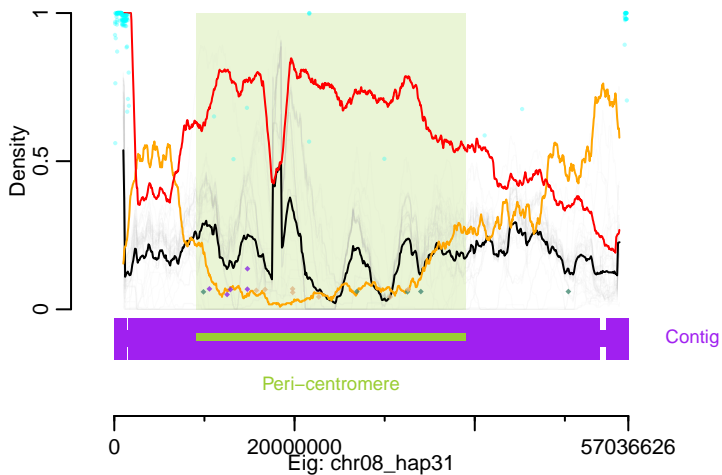

Supplement: Supplementary file 5 — Supplementary Figs. 3–14, 20, 21, 23–27 and 34–44. [file 41586_2025_8843_MOESM5_ESM.zip › suppl_figure_3_to_14/suppl_figure_10_chr08/suppl_figure_10au_31_C_EgH.pdf]

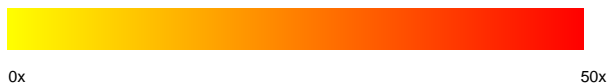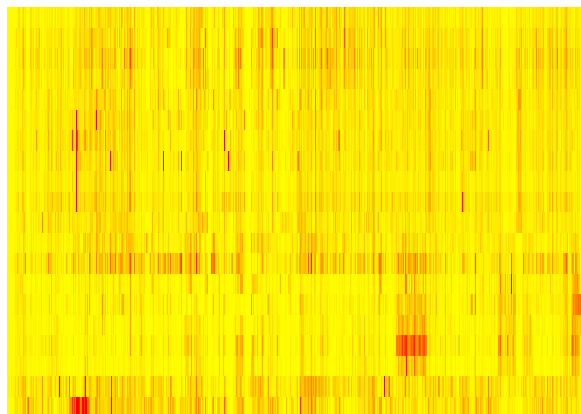

— TE — Gene — Non-aln • TanRep ♦ CenRep ■ rDNA

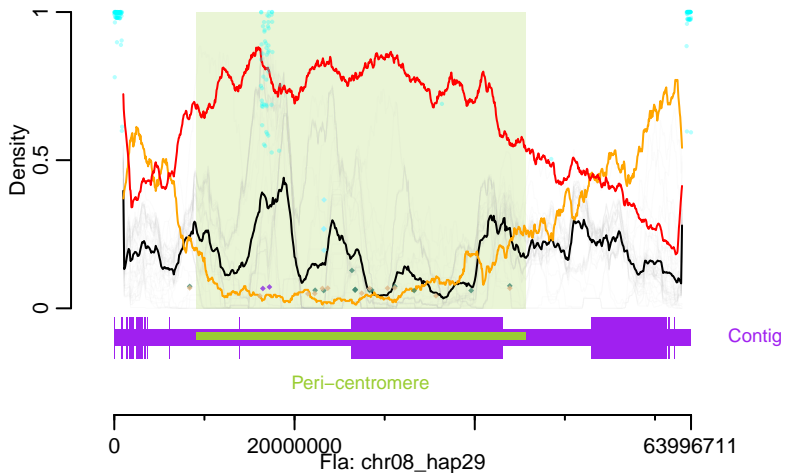

Supplement: Supplementary file 5 — Supplementary Figs. 3–14, 20, 21, 23–27 and 34–44. [file 41586_2025_8843_MOESM5_ESM.zip › suppl_figure_3_to_14/suppl_figure_10_chr08/suppl_figure_10ag_29_G_Fla.pdf]

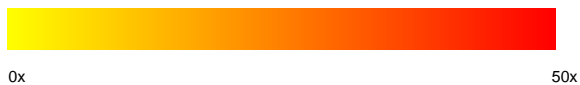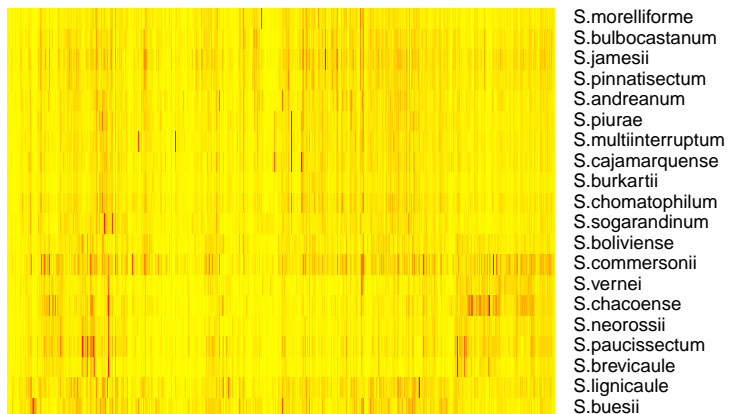

— TE — Gene — Non-aln • TanRep ♦ CenRep ■ rDNA

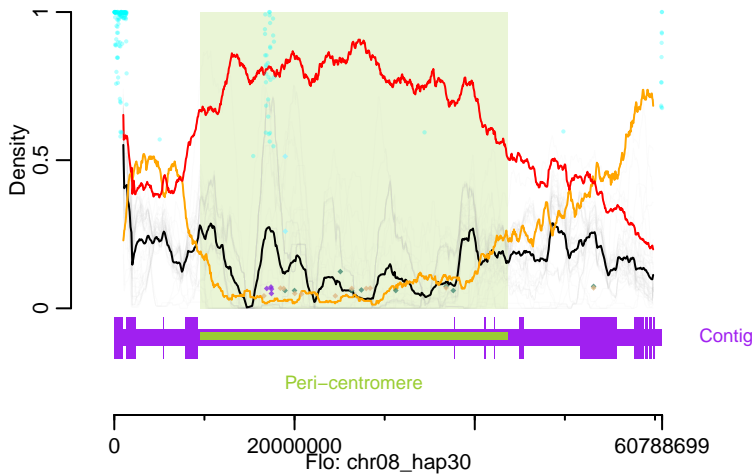

Supplement: Supplementary file 5 — Supplementary Figs. 3–14, 20, 21, 23–27 and 34–44. [file 41586_2025_8843_MOESM5_ESM.zip › suppl_figure_3_to_14/suppl_figure_10_chr08/suppl_figure_10ao_30_E_Flo.pdf]

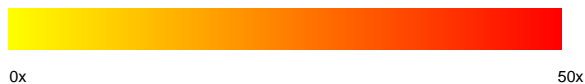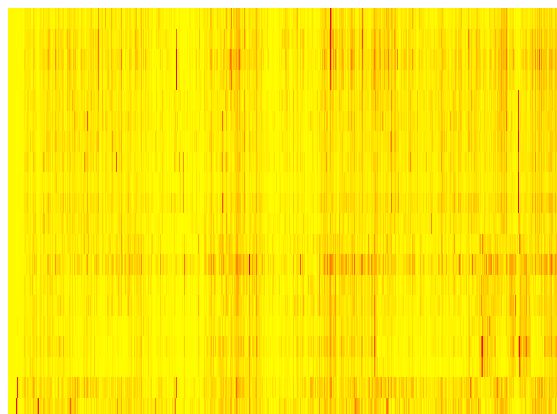

— TE — Gene — Non-aln • TanRep ♦ CenRep ■ rDNA

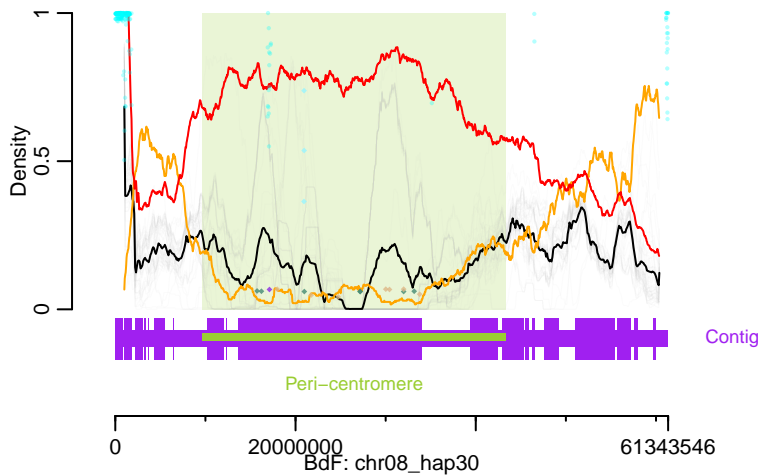

Supplement: Supplementary file 5 — Supplementary Figs. 3–14, 20, 21, 23–27 and 34–44. [file 41586_2025_8843_MOESM5_ESM.zip › suppl_figure_3_to_14/suppl_figure_10_chr08/suppl_figure_10al_30_B_BdF.pdf]

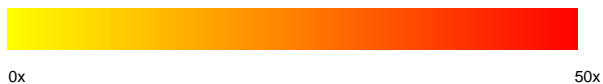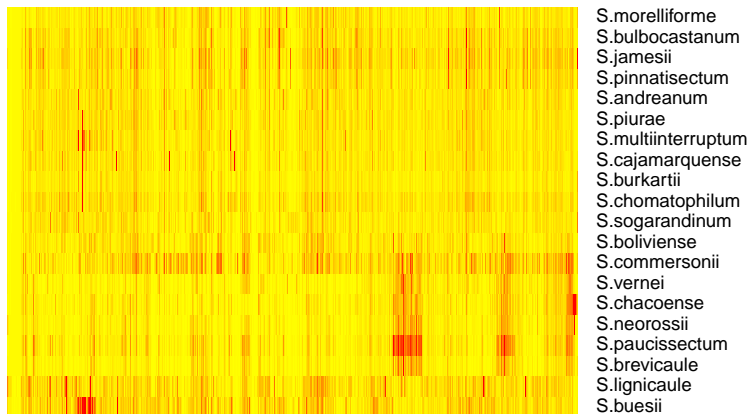

— TE — Gene — Non-aln • TanRep ♦ CenRep ■ rDNA

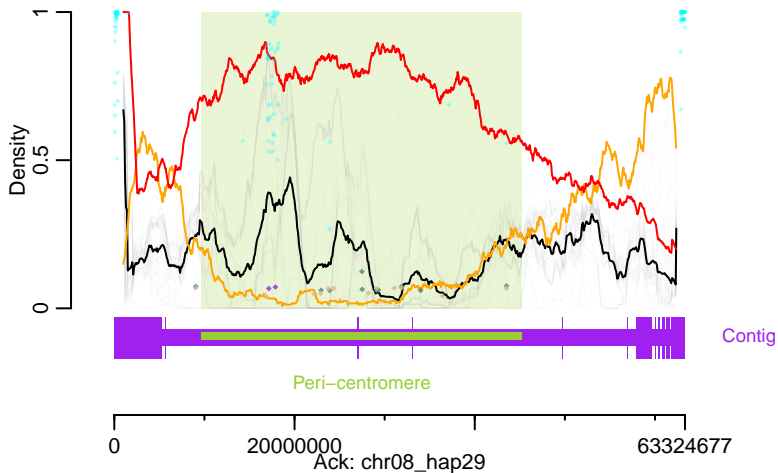

Supplement: Supplementary file 5 — Supplementary Figs. 3–14, 20, 21, 23–27 and 34–44. [file 41586_2025_8843_MOESM5_ESM.zip › suppl_figure_3_to_14/suppl_figure_10_chr08/suppl_figure_10af_29_F_Ack.pdf]

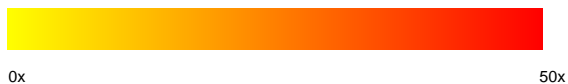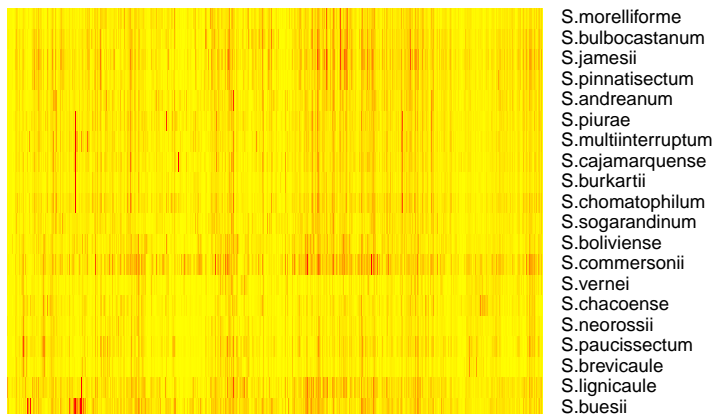

— TE — Gene — Non-aln ● TanRep ♦ CenRep ■ rDNA

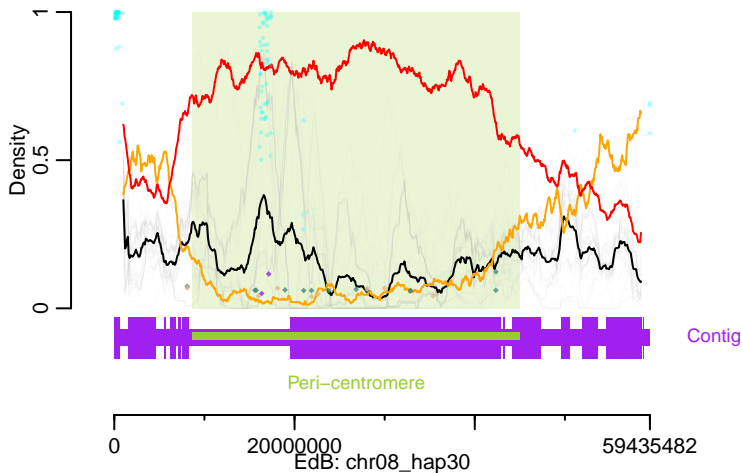

Supplement: Supplementary file 5 — Supplementary Figs. 3–14, 20, 21, 23–27 and 34–44. [file 41586_2025_8843_MOESM5_ESM.zip › suppl_figure_3_to_14/suppl_figure_10_chr08/suppl_figure_10at_30_J_EdB.pdf]

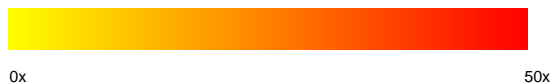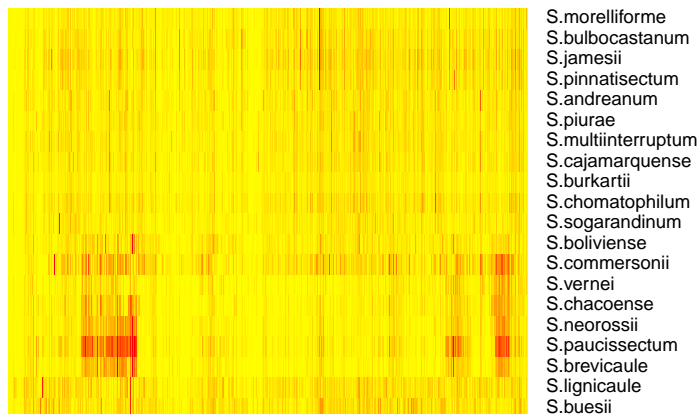

— TE — Gene — Non-aln • TanRep ♦ CenRep ■ rDNA

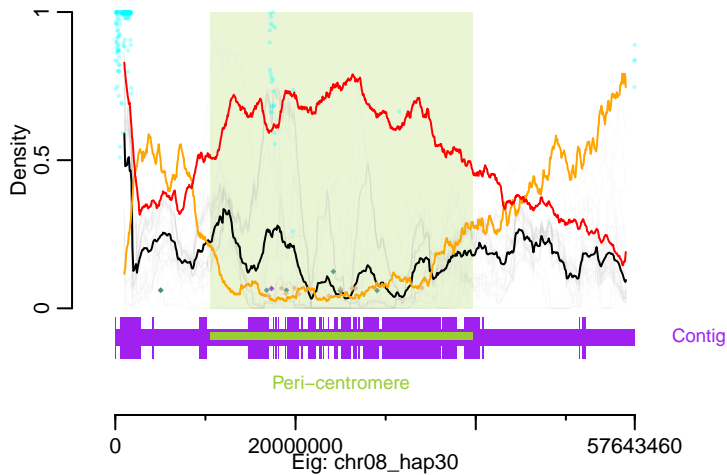

Supplement: Supplementary file 5 — Supplementary Figs. 3–14, 20, 21, 23–27 and 34–44. [file 41586_2025_8843_MOESM5_ESM.zip › suppl_figure_3_to_14/suppl_figure_10_chr08/suppl_figure_10am_30_C_EgH.pdf]

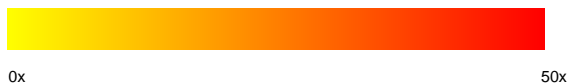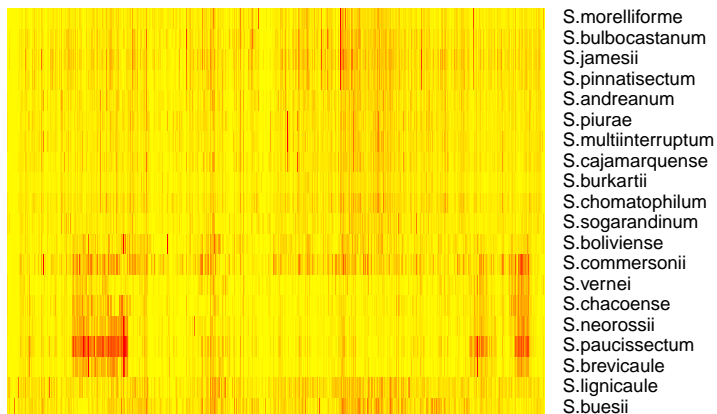

— TE — Gene — Non-aln ● TanRep ♦ CenRep ■ rDNA

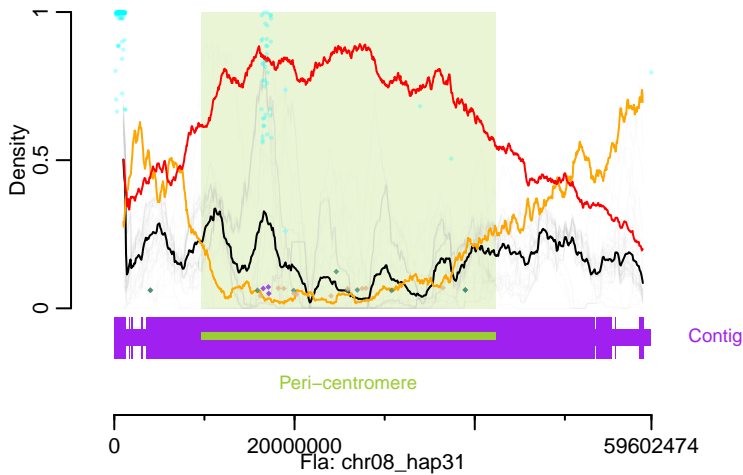

Supplement: Supplementary file 5 — Supplementary Figs. 3–14, 20, 21, 23–27 and 34–44. [file 41586_2025_8843_MOESM5_ESM.zip › suppl_figure_3_to_14/suppl_figure_10_chr08/suppl_figure_10ba_31_G_Fla.pdf]

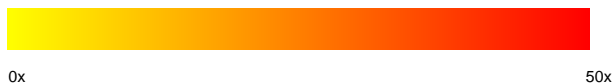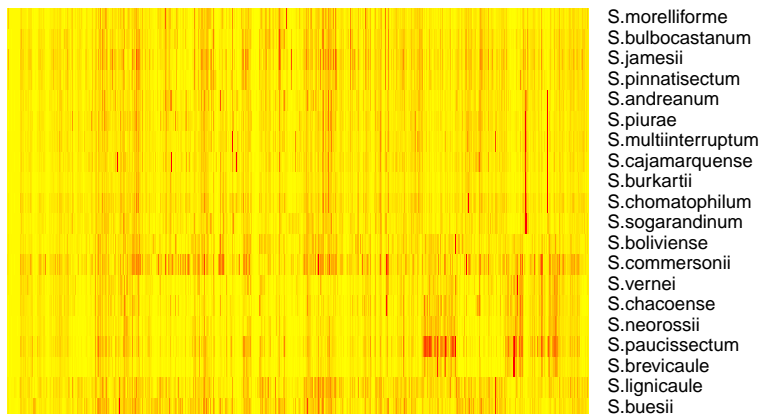

— TE — Gene — Non-aln • TanRep ♦ CenRep ■ rDNA

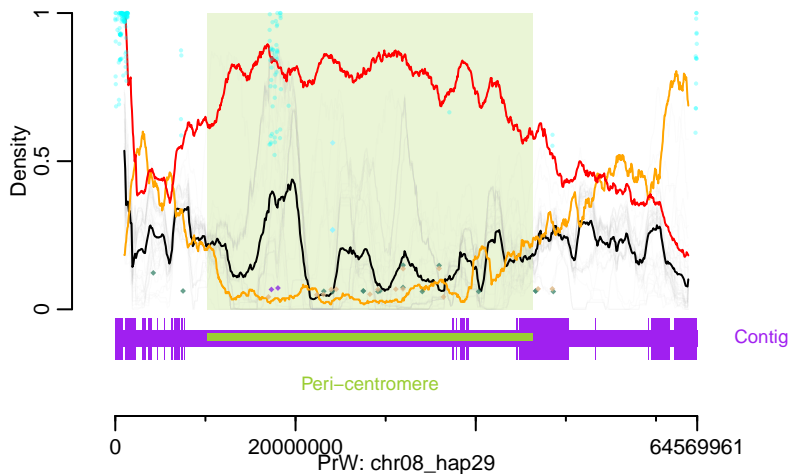

Supplement: Supplementary file 5 — Supplementary Figs. 3–14, 20, 21, 23–27 and 34–44. [file 41586_2025_8843_MOESM5_ESM.zip › suppl_figure_3_to_14/suppl_figure_10_chr08/suppl_figure_10ad_29_D_PrW.pdf]

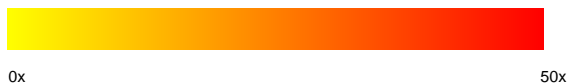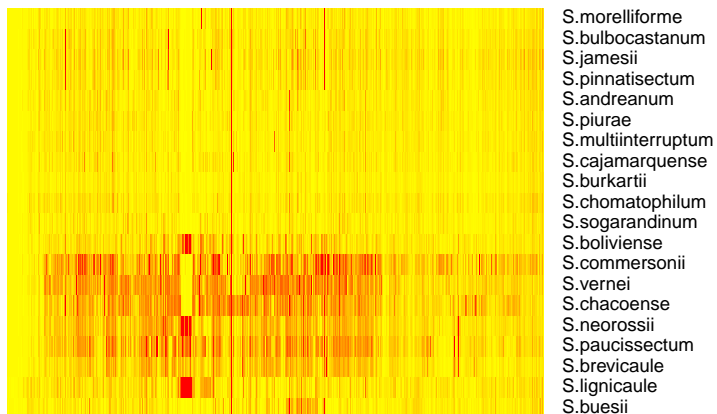

— TE — Gene — Non-aln ● TanRep ♦ CenRep ■ rDNA

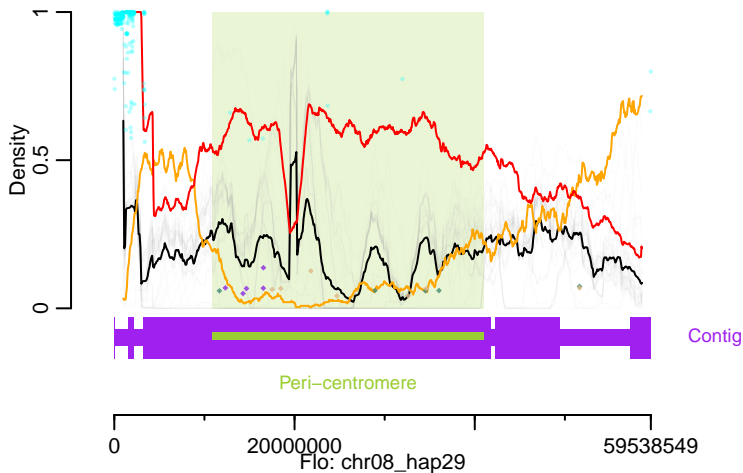

Supplement: Supplementary file 5 — Supplementary Figs. 3–14, 20, 21, 23–27 and 34–44. [file 41586_2025_8843_MOESM5_ESM.zip › suppl_figure_3_to_14/suppl_figure_10_chr08/suppl_figure_10ae_29_E_Flo.pdf]

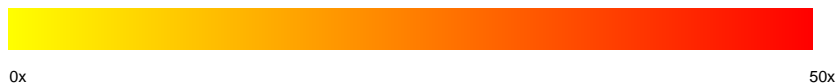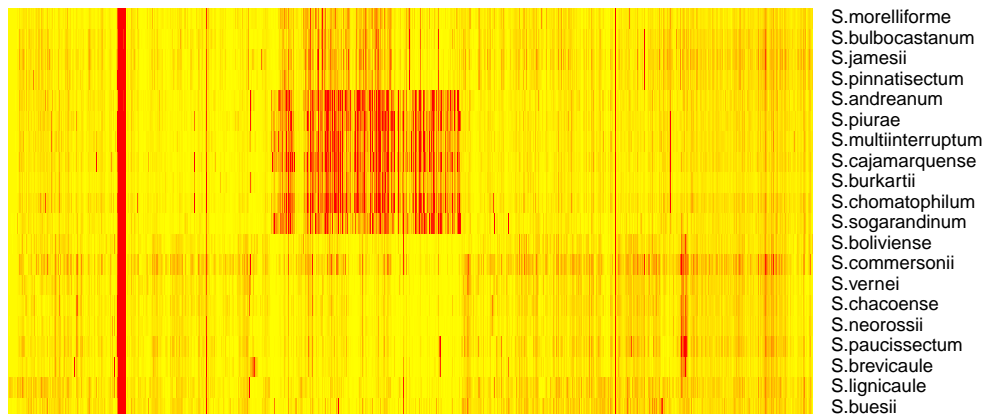

— TE — Gene — Non-aln • TanRep ♦ CenRep ■ rDNA

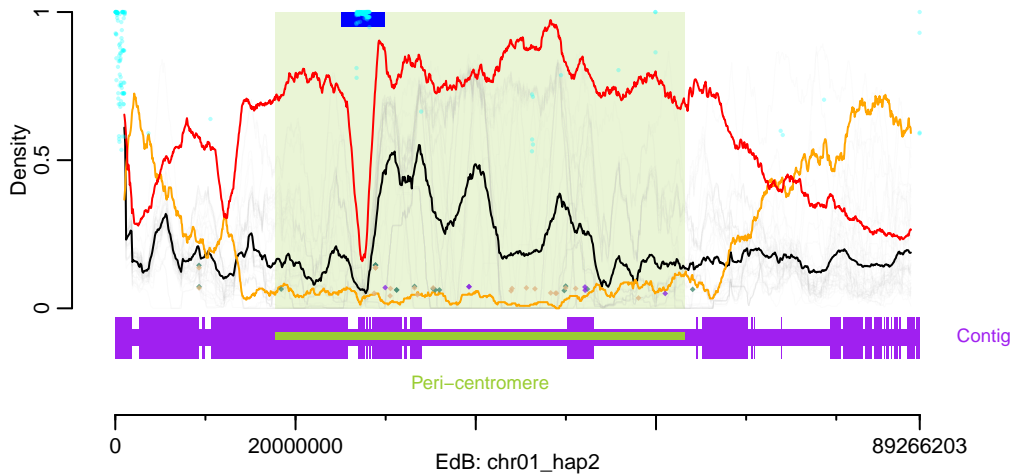

Supplement: Supplementary file 5 — Supplementary Figs. 3–14, 20, 21, 23–27 and 34–44. [file 41586_2025_8843_MOESM5_ESM.zip › suppl_figure_3_to_14/suppl_figure_3_chr01/suppl_figure_3at_2_J_EdB.pdf]

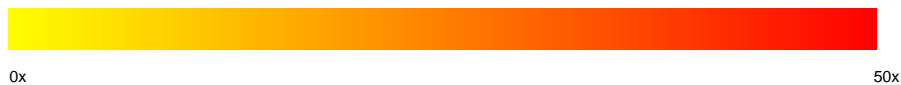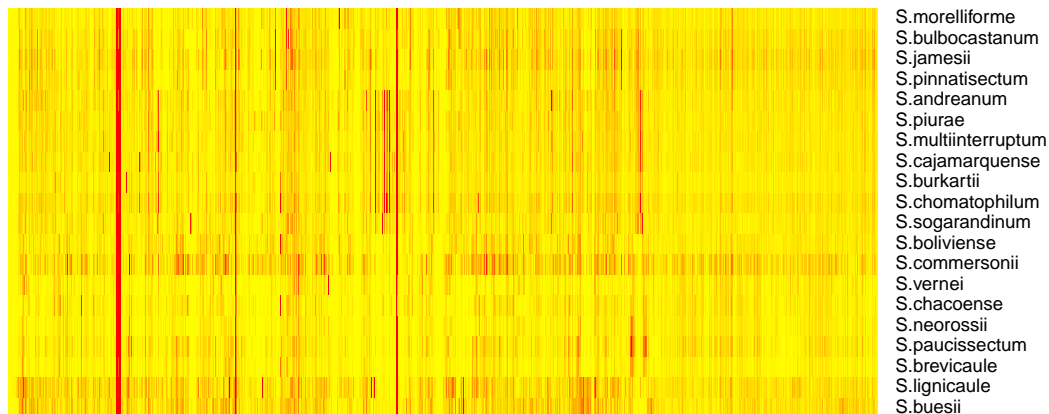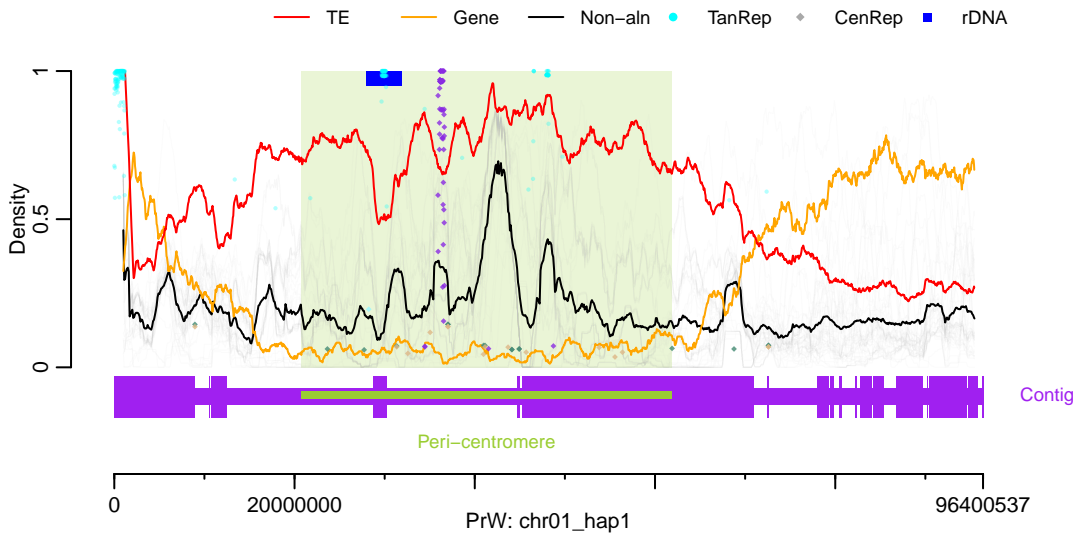

Supplement: Supplementary file 5 — Supplementary Figs. 3–14, 20, 21, 23–27 and 34–44. [file 41586_2025_8843_MOESM5_ESM.zip › suppl_figure_3_to_14/suppl_figure_3_chr01/suppl_figure_3ad_1_D_PrW.pdf]

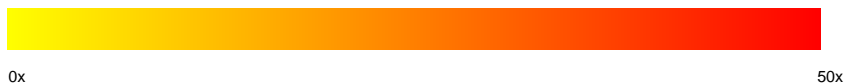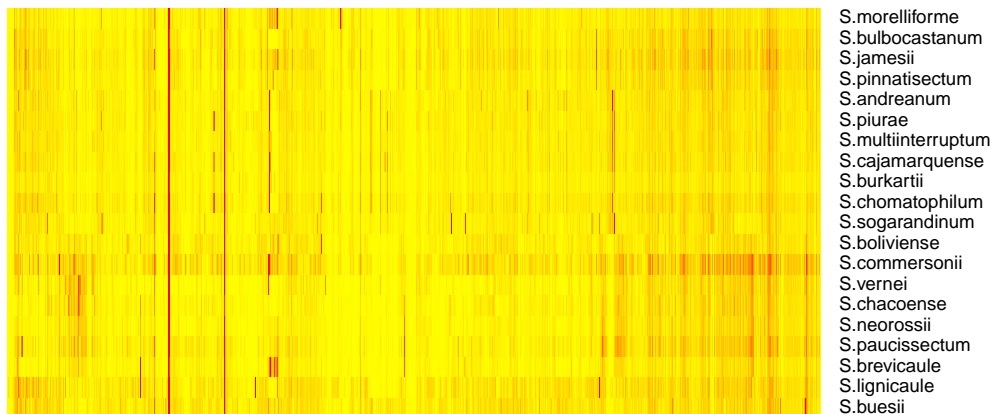

— TE — Gene — Non-aln • TanRep ♦ CenRep ■ rDNA

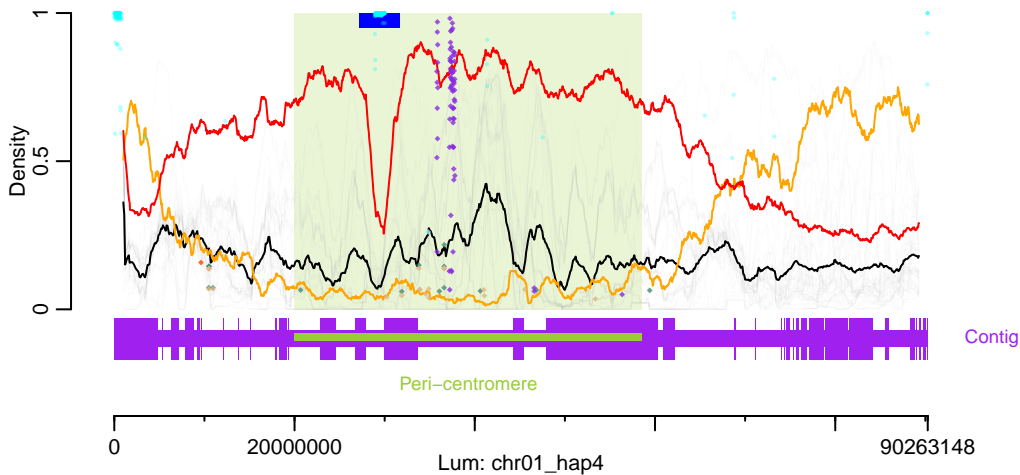

Supplement: Supplementary file 5 — Supplementary Figs. 3–14, 20, 21, 23–27 and 34–44. [file 41586_2025_8843_MOESM5_ESM.zip › suppl_figure_3_to_14/suppl_figure_3_chr01/suppl_figure_3bm_4_I_Lum.pdf]

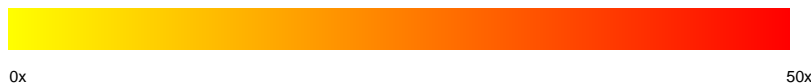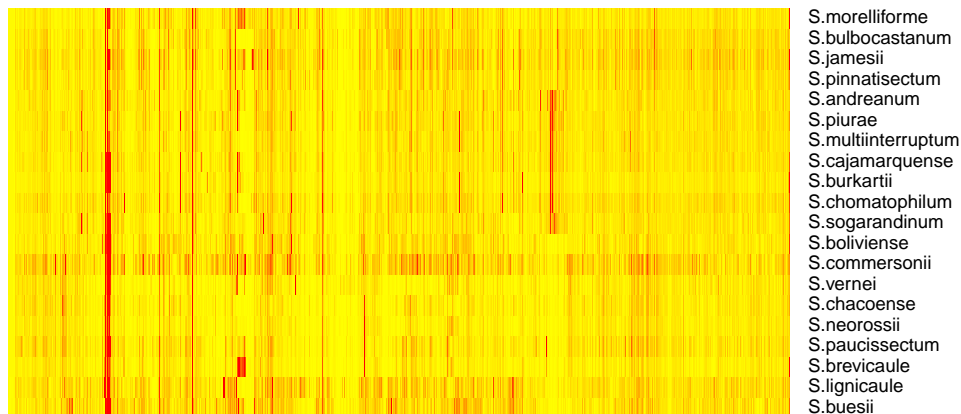

— TE — Gene — Non-aln • TanRep ♦ CenRep ■ rDNA

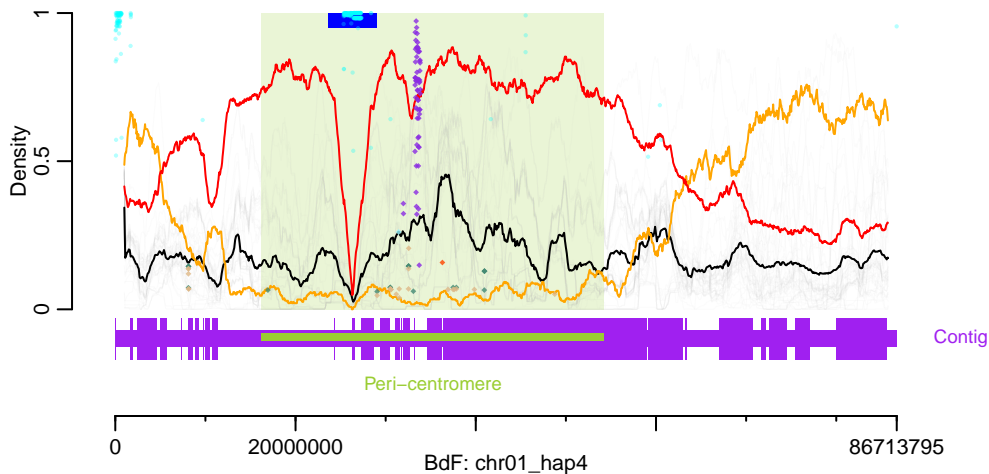

Supplement: Supplementary file 5 — Supplementary Figs. 3–14, 20, 21, 23–27 and 34–44. [file 41586_2025_8843_MOESM5_ESM.zip › suppl_figure_3_to_14/suppl_figure_3_chr01/suppl_figure_3bf_4_B_BdF.pdf]

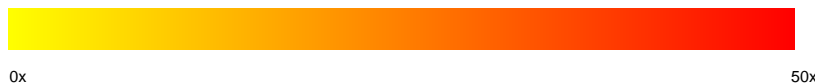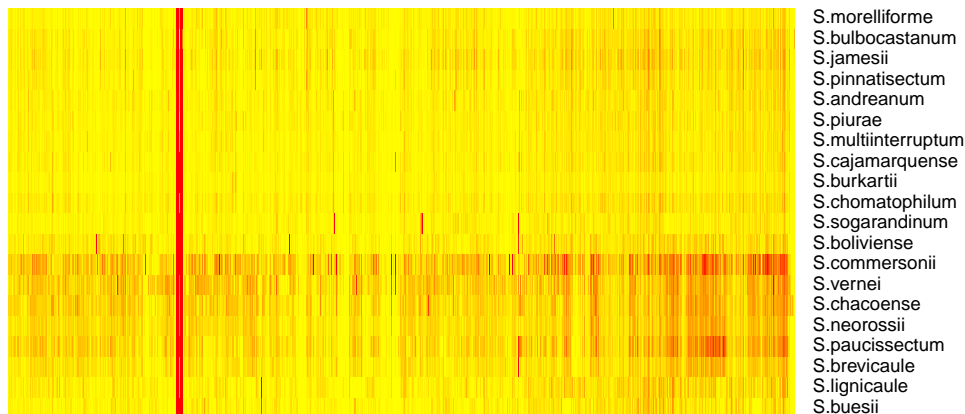

— TE — Gene — Non-aln • TanRep ♦ CenRep ■ rDNA

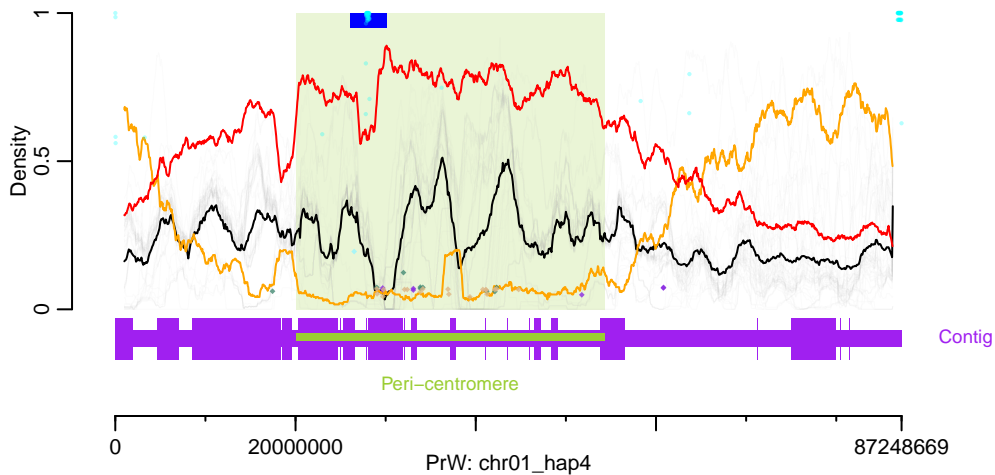

Supplement: Supplementary file 5 — Supplementary Figs. 3–14, 20, 21, 23–27 and 34–44. [file 41586_2025_8843_MOESM5_ESM.zip › suppl_figure_3_to_14/suppl_figure_3_chr01/suppl_figure_3bh_4_D_PrW.pdf]

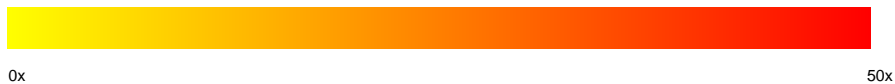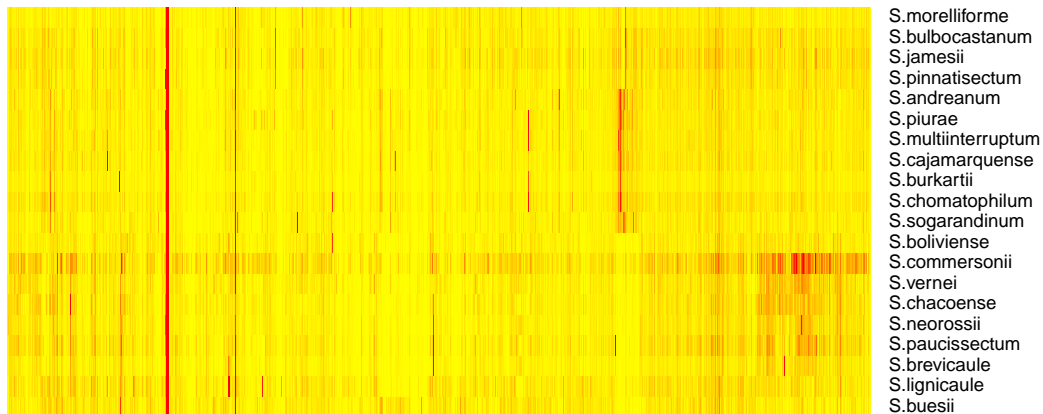

— TE — Gene — Non-aln • TanRep ♦ CenRep ■ rDNA

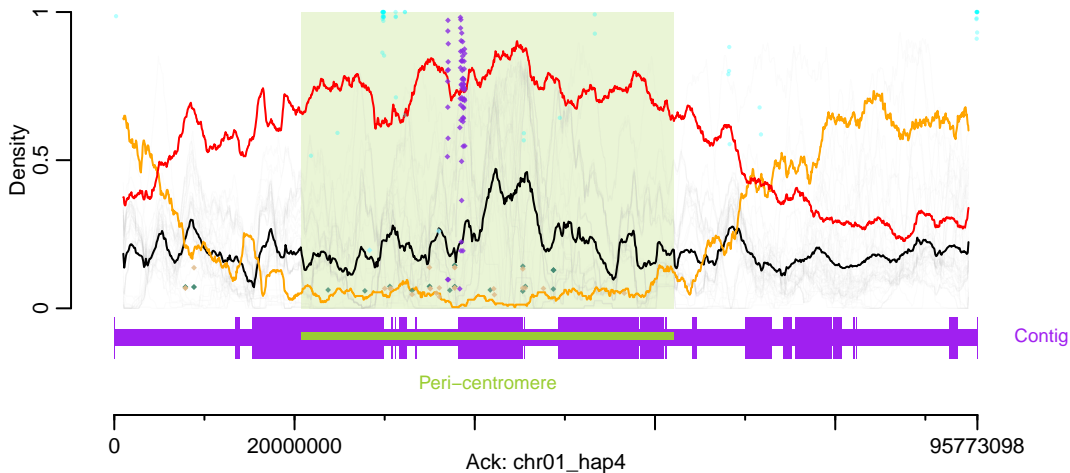

Supplement: Supplementary file 5 — Supplementary Figs. 3–14, 20, 21, 23–27 and 34–44. [file 41586_2025_8843_MOESM5_ESM.zip › suppl_figure_3_to_14/suppl_figure_3_chr01/suppl_figure_3bj_4_F_Ack.pdf]

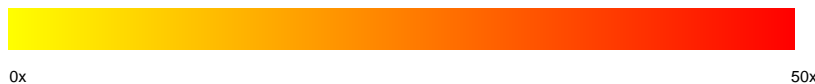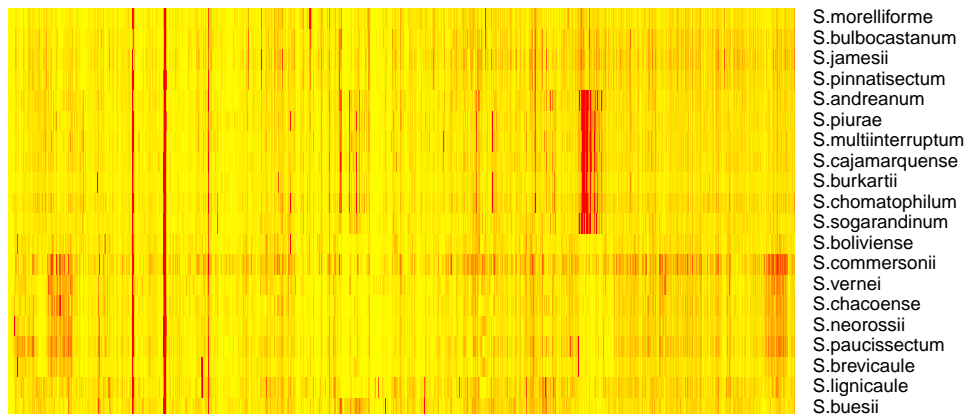

— TE — Gene — Non-aln • TanRep ♦ CenRep ■ rDNA

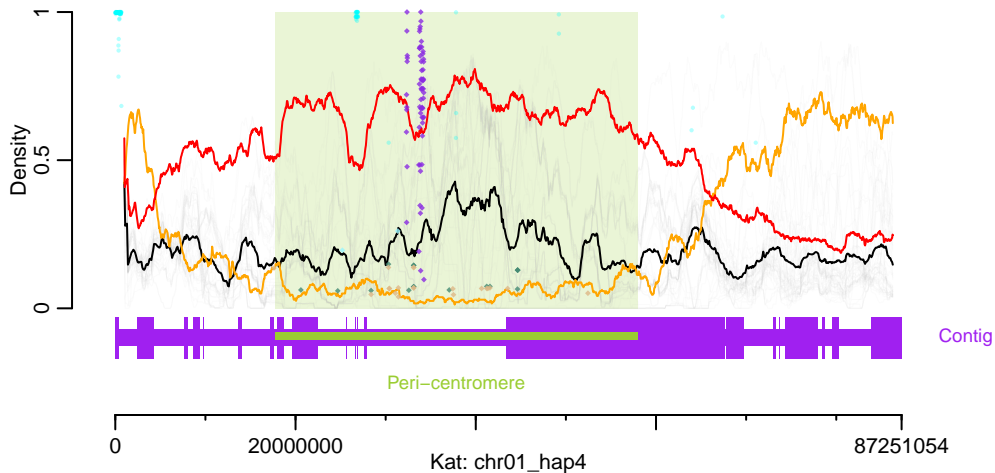

Supplement: Supplementary file 5 — Supplementary Figs. 3–14, 20, 21, 23–27 and 34–44. [file 41586_2025_8843_MOESM5_ESM.zip › suppl_figure_3_to_14/suppl_figure_3_chr01/suppl_figure_3bl_4_H_Kat.pdf]

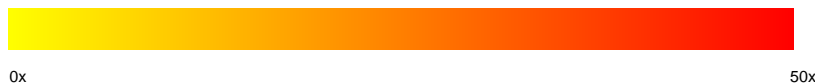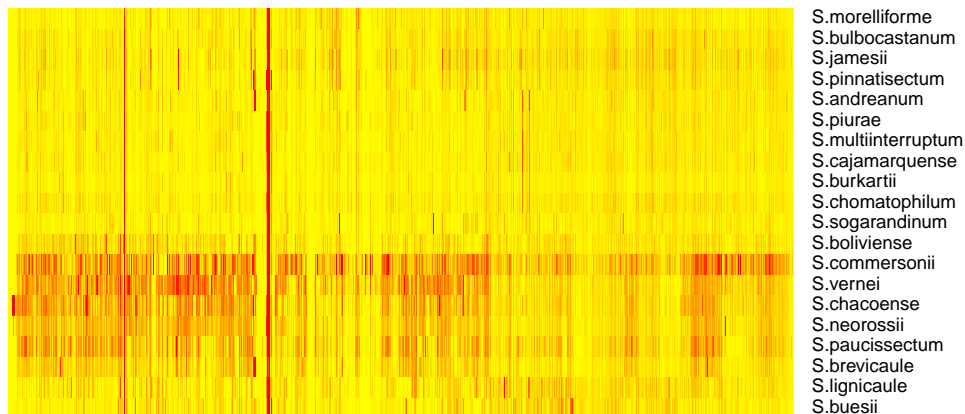

— TE — Gene — Non-aln • TanRep ♦ CenRep ■ rDNA

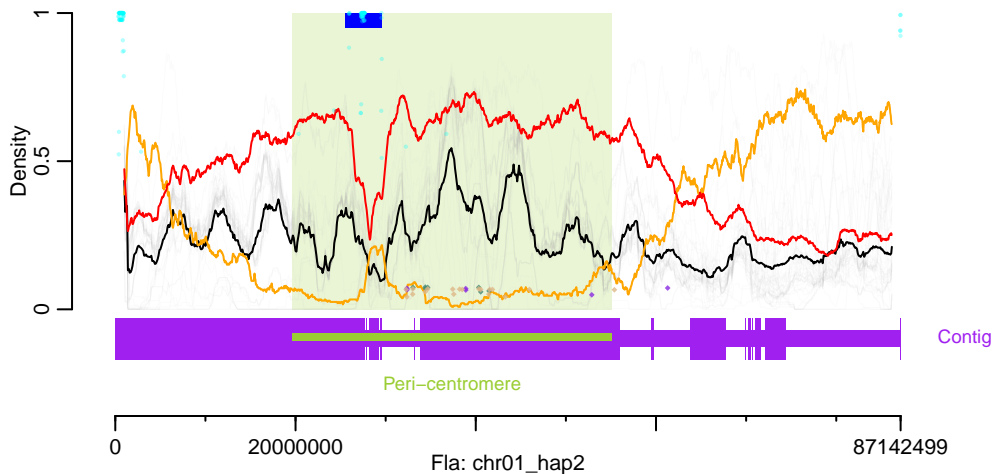

Supplement: Supplementary file 5 — Supplementary Figs. 3–14, 20, 21, 23–27 and 34–44. [file 41586_2025_8843_MOESM5_ESM.zip › suppl_figure_3_to_14/suppl_figure_3_chr01/suppl_figure_3aq_2_G_Fla.pdf]

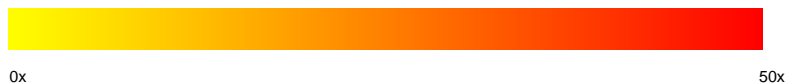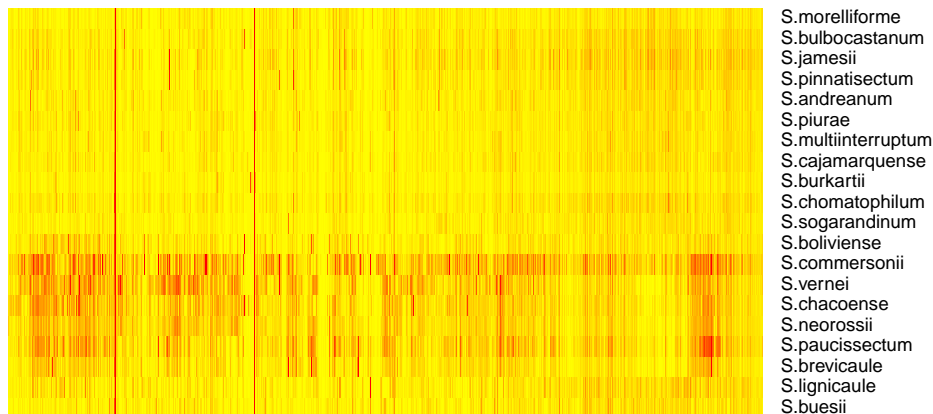

— TE — Gene — Non-aln • TanRep ♦ CenRep ■ rDNA

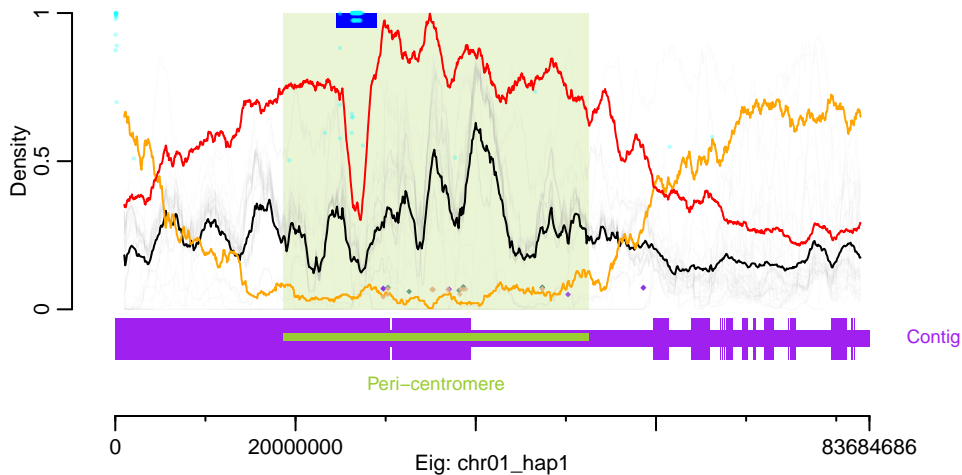

Supplement: Supplementary file 5 — Supplementary Figs. 3–14, 20, 21, 23–27 and 34–44. [file 41586_2025_8843_MOESM5_ESM.zip › suppl_figure_3_to_14/suppl_figure_3_chr01/suppl_figure_3ac_1_C_EgH.pdf]

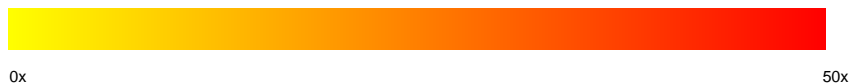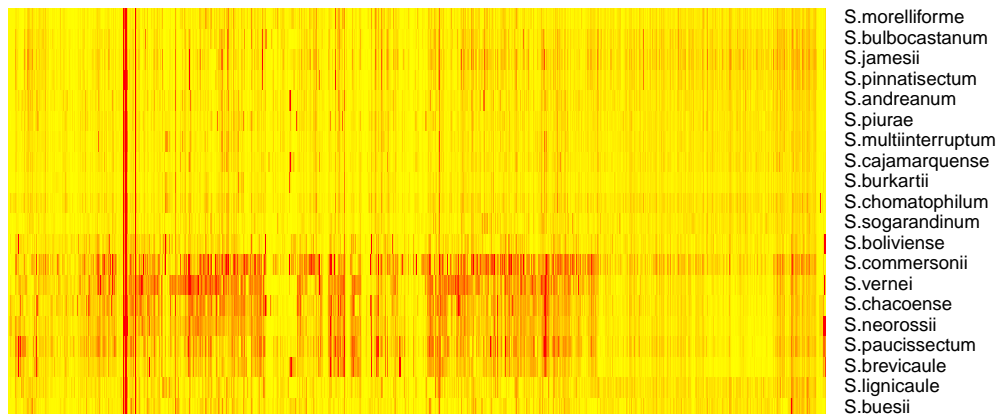

— TE — Gene — Non-aln • TanRep ♦ CenRep ■ rDNA

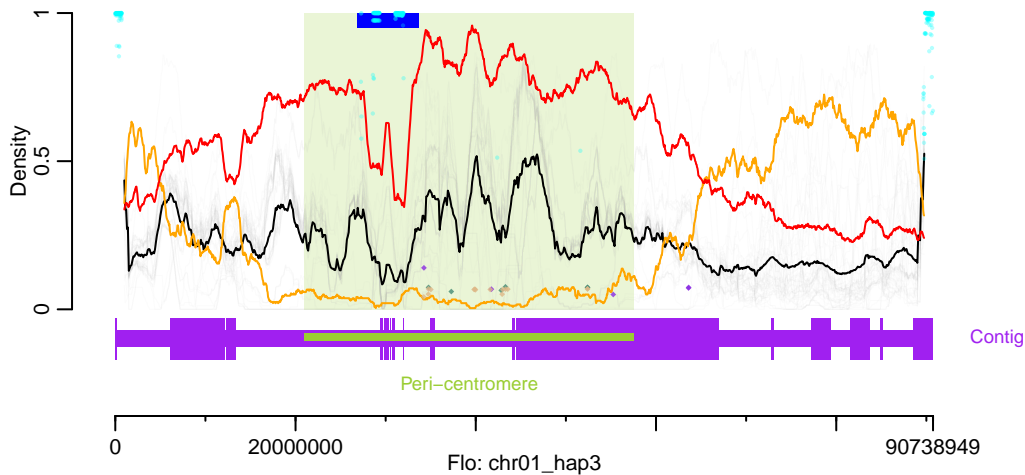

Supplement: Supplementary file 5 — Supplementary Figs. 3–14, 20, 21, 23–27 and 34–44. [file 41586_2025_8843_MOESM5_ESM.zip › suppl_figure_3_to_14/suppl_figure_3_chr01/suppl_figure_3ay_3_E_Flo.pdf]

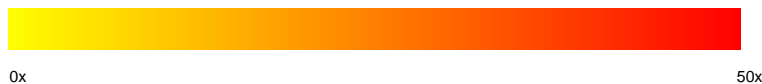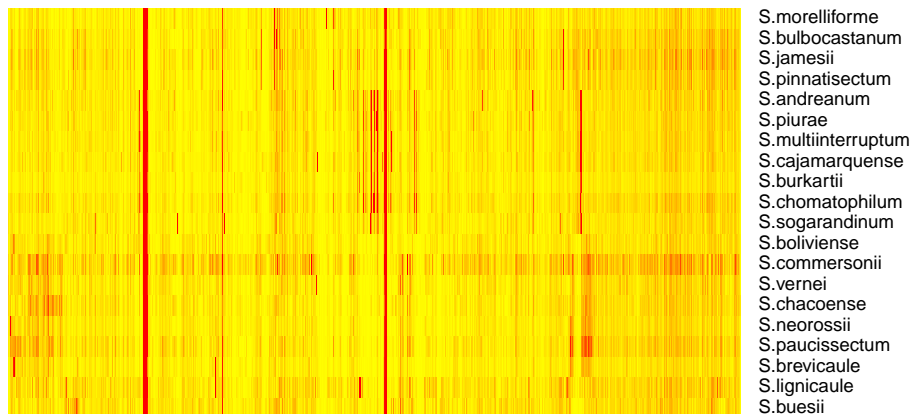

— TE — Gene — Non-aln • TanRep ♦ CenRep ■ rDNA

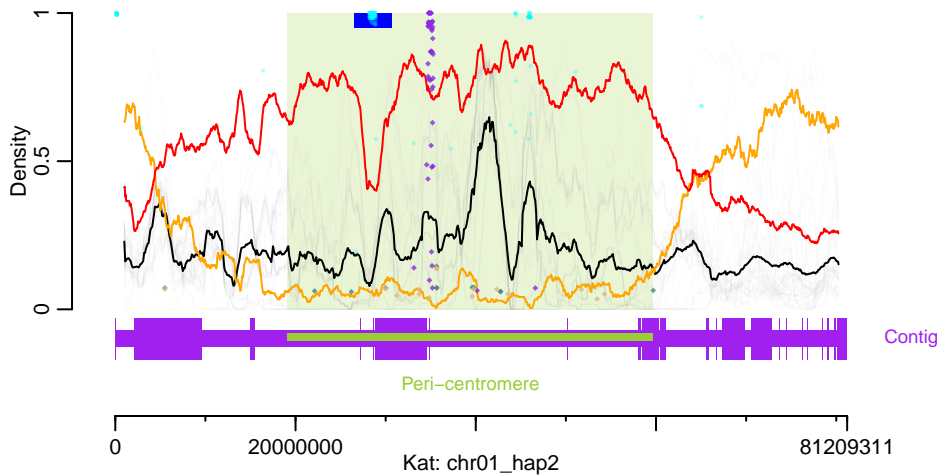

Supplement: Supplementary file 5 — Supplementary Figs. 3–14, 20, 21, 23–27 and 34–44. [file 41586_2025_8843_MOESM5_ESM.zip › suppl_figure_3_to_14/suppl_figure_3_chr01/suppl_figure_3ar_2_H_Kat.pdf]

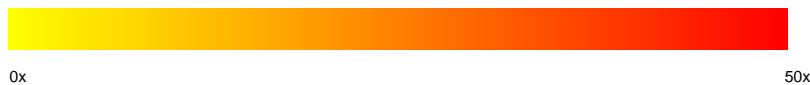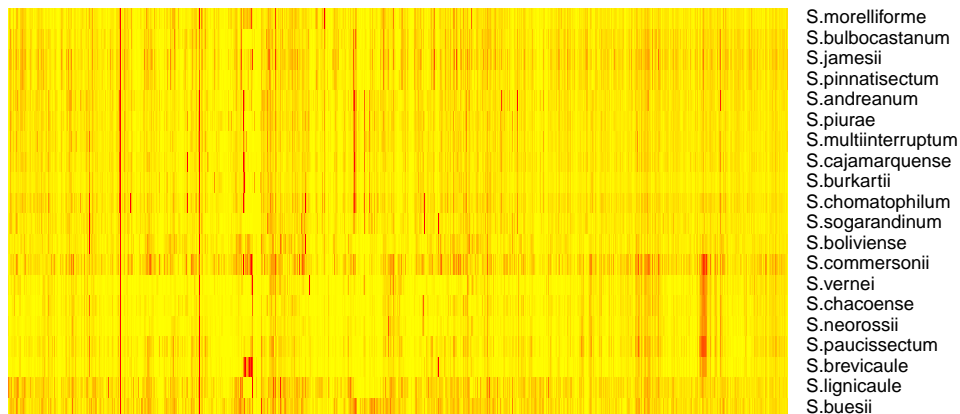

— TE — Gene — Non-aln • TanRep ♦ CenRep ■ rDNA

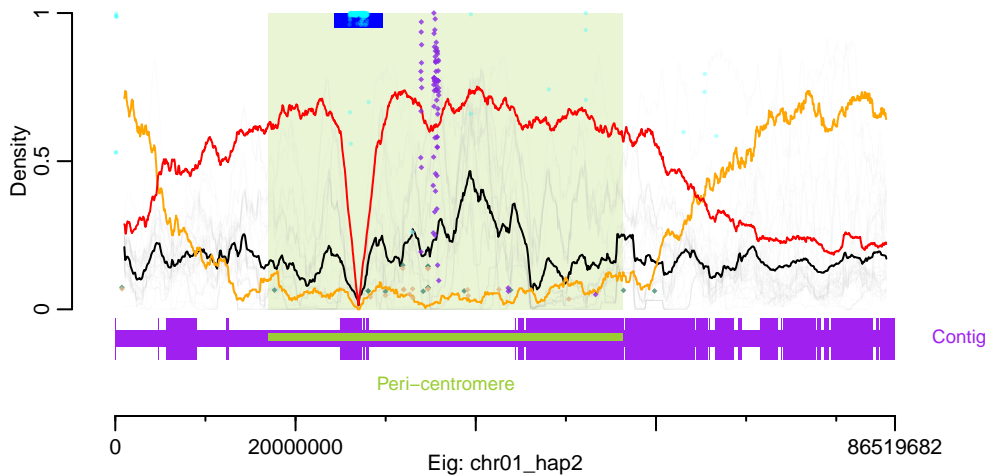

Supplement: Supplementary file 5 — Supplementary Figs. 3–14, 20, 21, 23–27 and 34–44. [file 41586_2025_8843_MOESM5_ESM.zip › suppl_figure_3_to_14/suppl_figure_3_chr01/suppl_figure_3am_2_C_EgH.pdf]

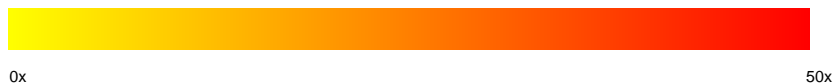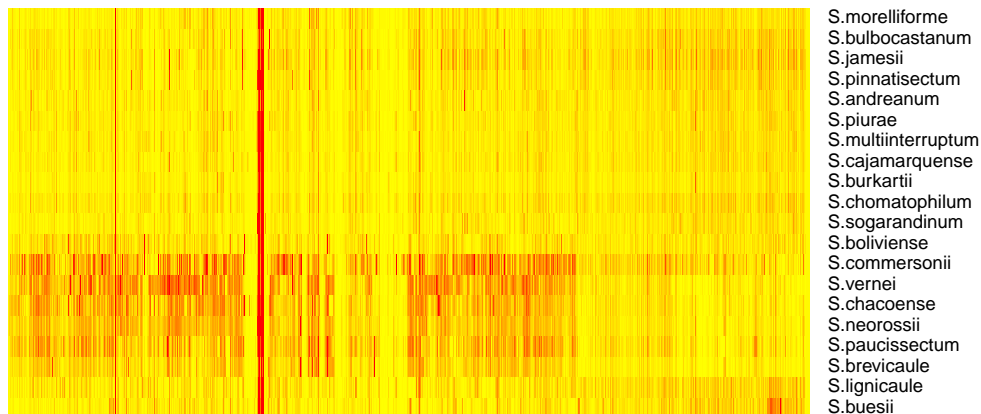

— TE — Gene — Non-aln • TanRep ♦ CenRep ■ rDNA

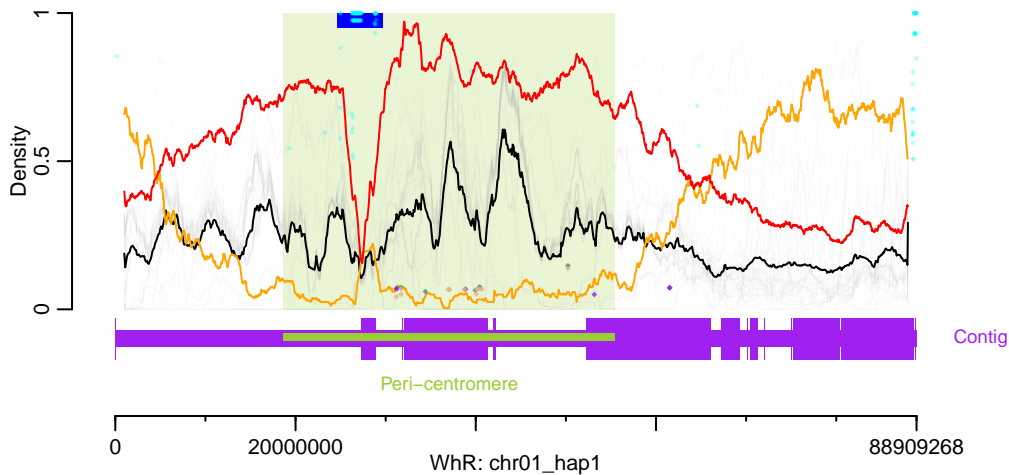

Supplement: Supplementary file 5 — Supplementary Figs. 3–14, 20, 21, 23–27 and 34–44. [file 41586_2025_8843_MOESM5_ESM.zip › suppl_figure_3_to_14/suppl_figure_3_chr01/suppl_figure_3aa_1_A_WhR.pdf]

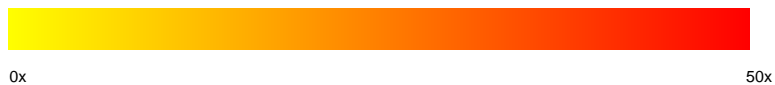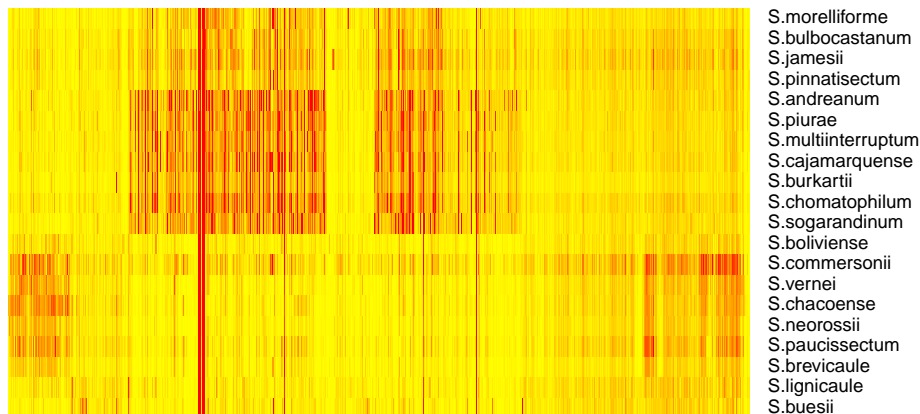

— TE — Gene — Non-aln • TanRep ♦ CenRep ■ rDNA

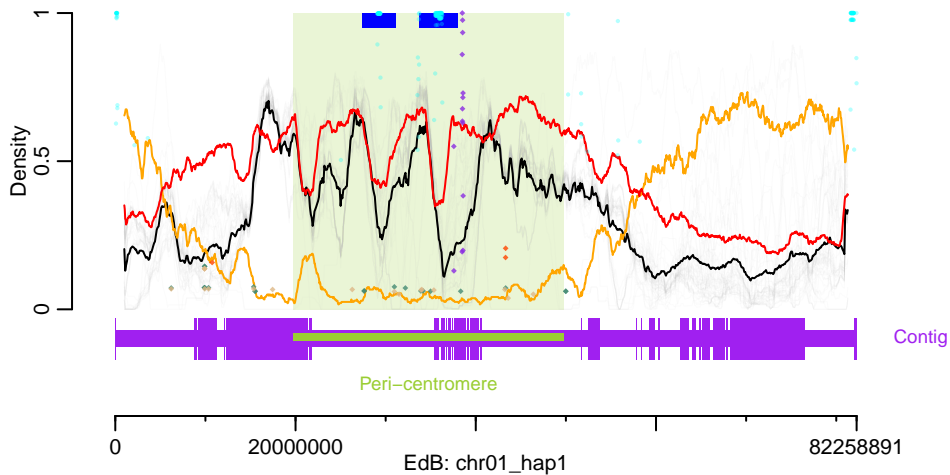

Supplement: Supplementary file 5 — Supplementary Figs. 3–14, 20, 21, 23–27 and 34–44. [file 41586_2025_8843_MOESM5_ESM.zip › suppl_figure_3_to_14/suppl_figure_3_chr01/suppl_figure_3aj_1_J_EdB.pdf]

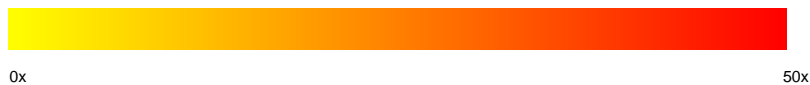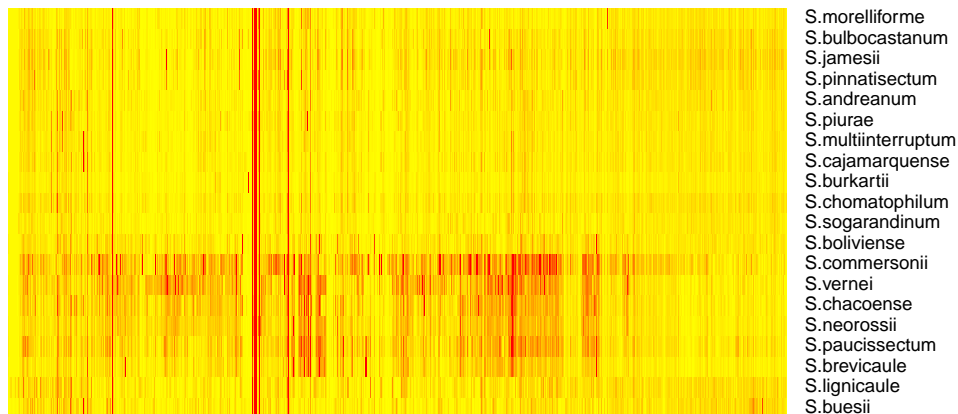

— TE — Gene — Non-aln • TanRep ♦ CenRep ■ rDNA

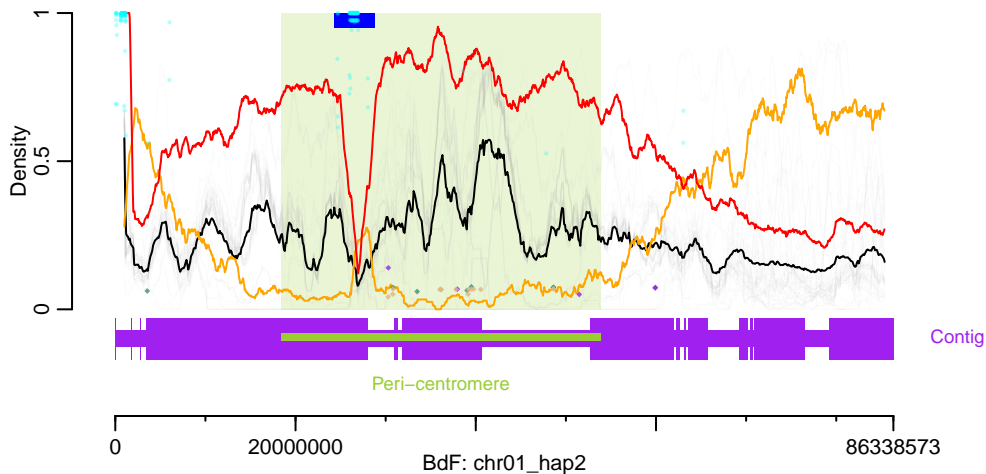

Supplement: Supplementary file 5 — Supplementary Figs. 3–14, 20, 21, 23–27 and 34–44. [file 41586_2025_8843_MOESM5_ESM.zip › suppl_figure_3_to_14/suppl_figure_3_chr01/suppl_figure_3al_2_B_BdF.pdf]

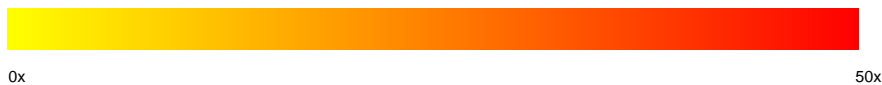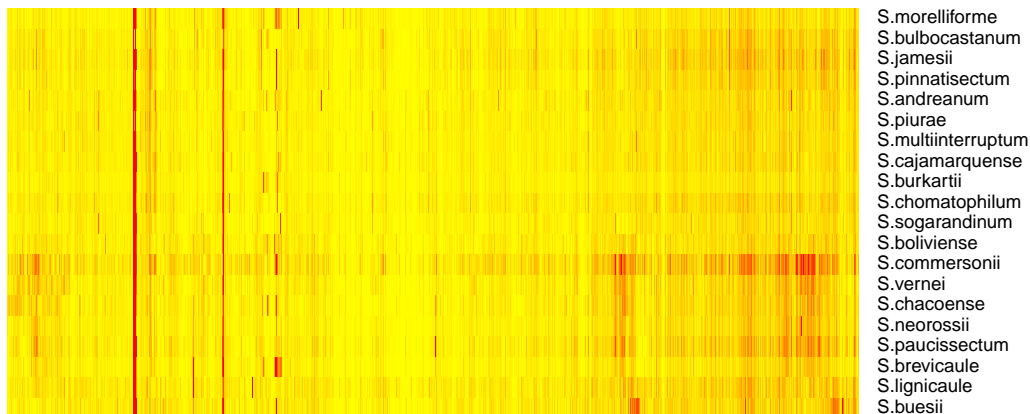

— TE — Gene — Non-aln • TanRep ♦ CenRep ■ rDNA

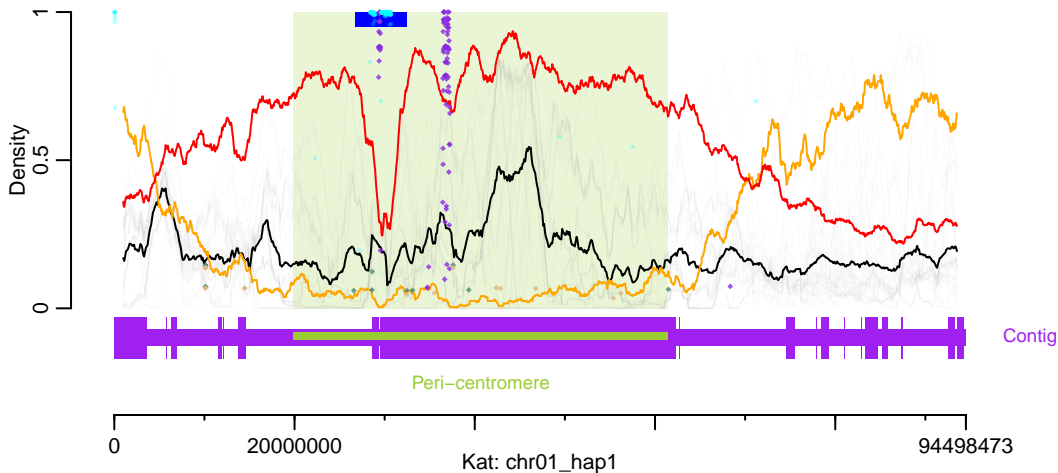

Supplement: Supplementary file 5 — Supplementary Figs. 3–14, 20, 21, 23–27 and 34–44. [file 41586_2025_8843_MOESM5_ESM.zip › suppl_figure_3_to_14/suppl_figure_3_chr01/suppl_figure_3ah_1_H_Kat.pdf]

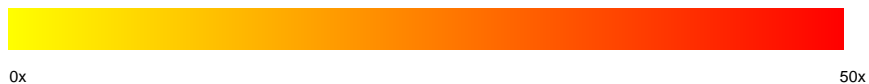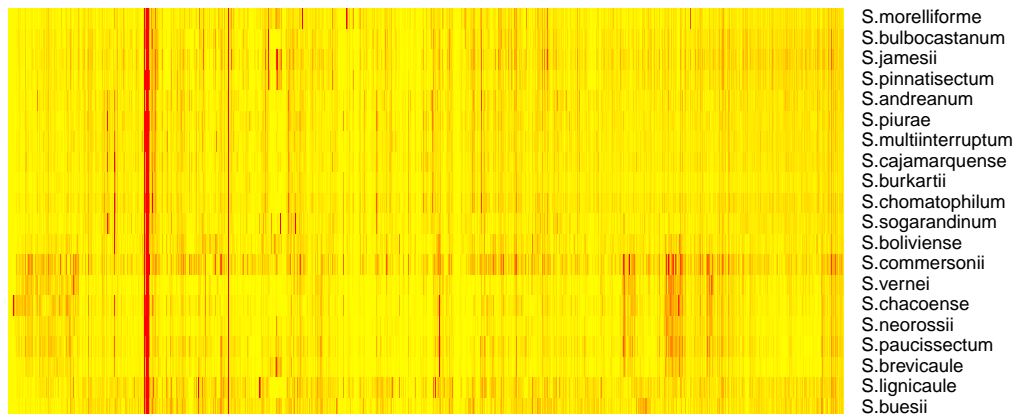

— TE — Gene — Non-aln • TanRep ♦ CenRep ■ rDNA

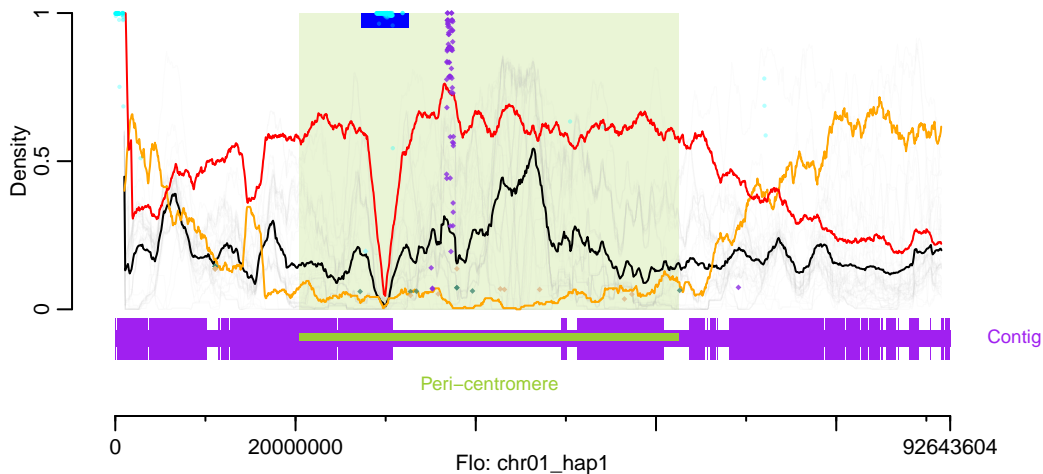

Supplement: Supplementary file 5 — Supplementary Figs. 3–14, 20, 21, 23–27 and 34–44. [file 41586_2025_8843_MOESM5_ESM.zip › suppl_figure_3_to_14/suppl_figure_3_chr01/suppl_figure_3ae_1_E_Flo.pdf]

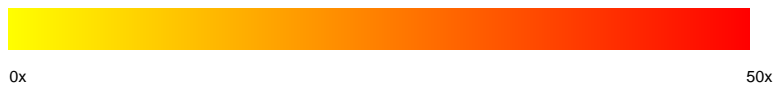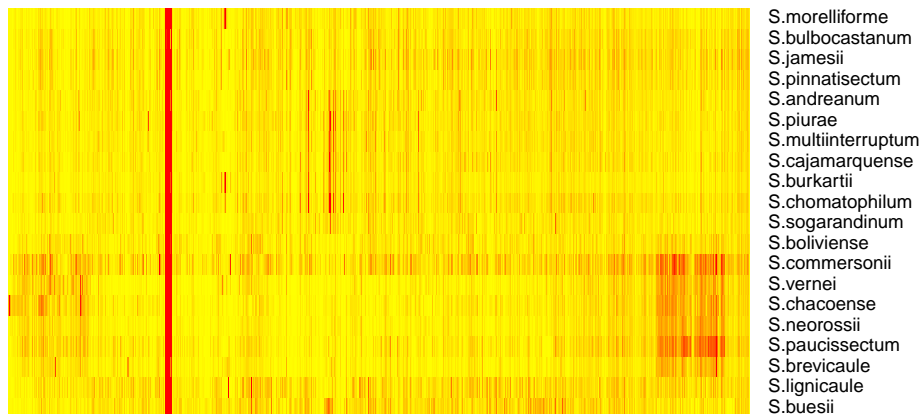

— TE — Gene — Non-aln • TanRep ♦ CenRep ■ rDNA

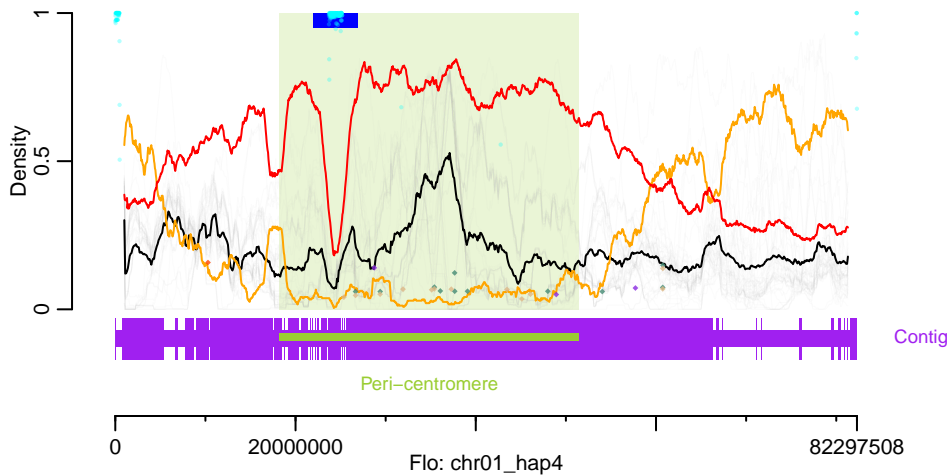

Supplement: Supplementary file 5 — Supplementary Figs. 3–14, 20, 21, 23–27 and 34–44. [file 41586_2025_8843_MOESM5_ESM.zip › suppl_figure_3_to_14/suppl_figure_3_chr01/suppl_figure_3bi_4_E_Flo.pdf]

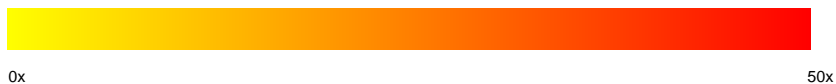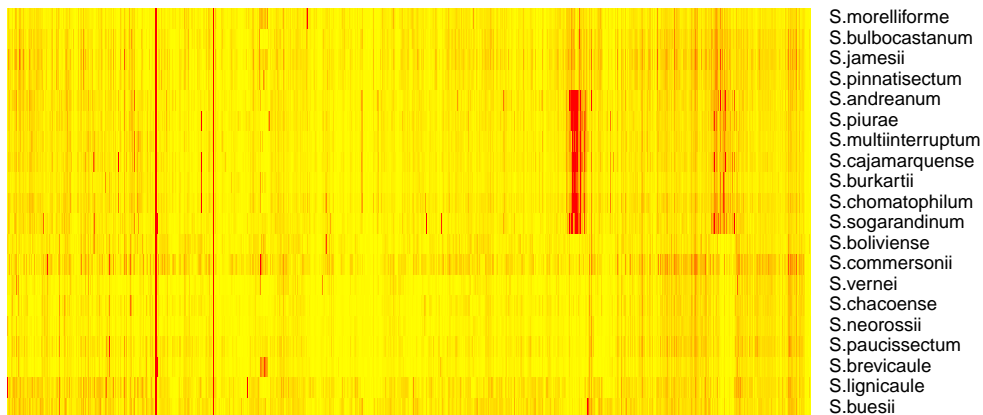

— TE — Gene — Non-aln ● TanRep ♦ CenRep ■ rDNA

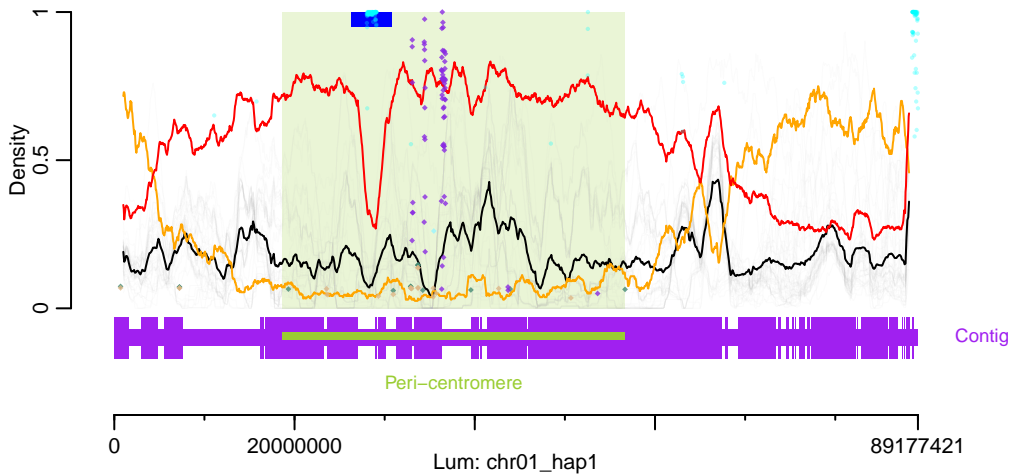

Supplement: Supplementary file 5 — Supplementary Figs. 3–14, 20, 21, 23–27 and 34–44. [file 41586_2025_8843_MOESM5_ESM.zip › suppl_figure_3_to_14/suppl_figure_3_chr01/suppl_figure_3ai_1_I_Lum.pdf]

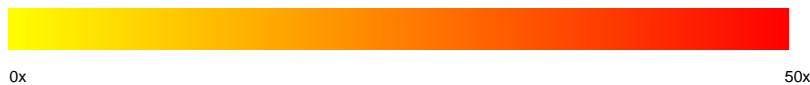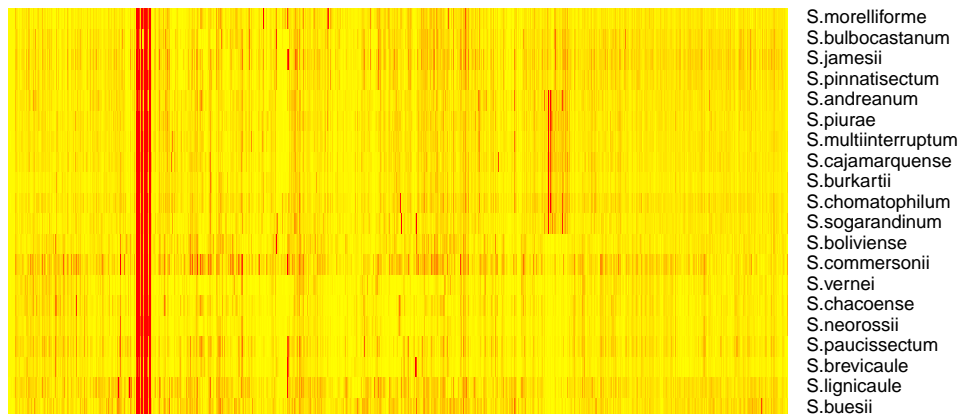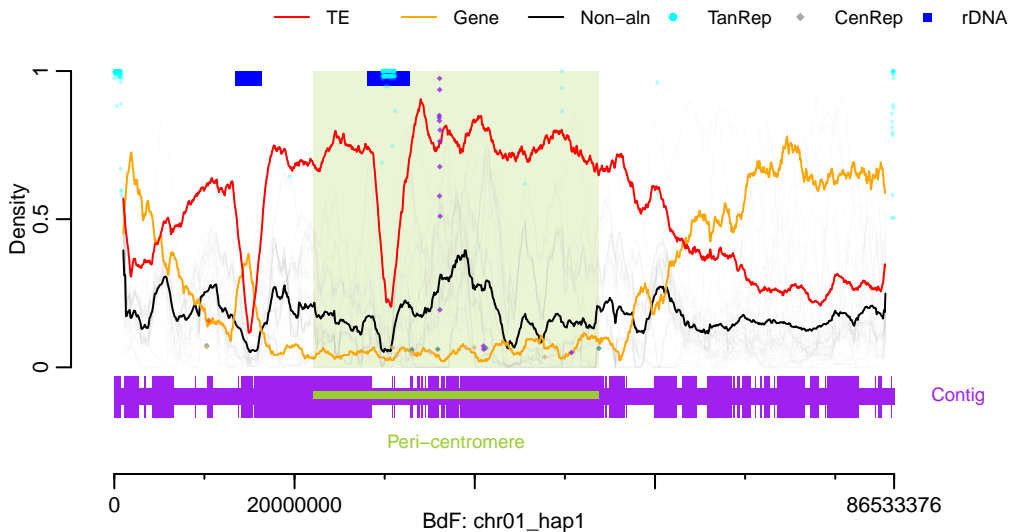

Supplement: Supplementary file 5 — Supplementary Figs. 3–14, 20, 21, 23–27 and 34–44. [file 41586_2025_8843_MOESM5_ESM.zip › suppl_figure_3_to_14/suppl_figure_3_chr01/suppl_figure_3ab_1_B_BdF.pdf]

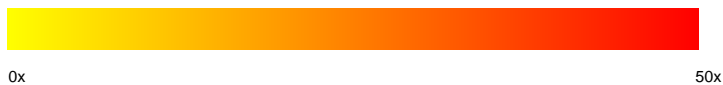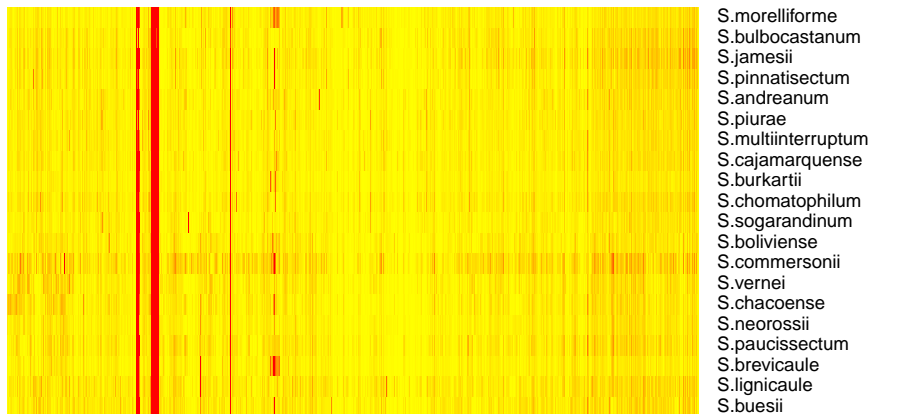

— TE — Gene — Non-aln • TanRep ♦ CenRep ■ rDNA

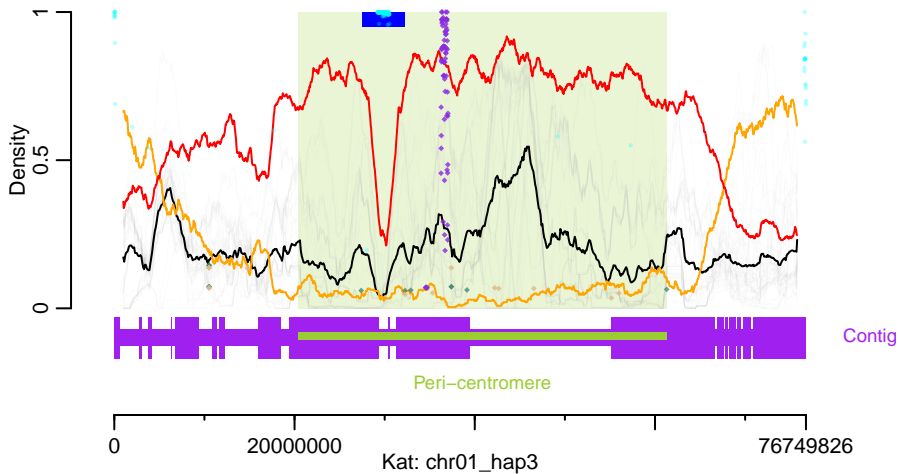

Supplement: Supplementary file 5 — Supplementary Figs. 3–14, 20, 21, 23–27 and 34–44. [file 41586_2025_8843_MOESM5_ESM.zip › suppl_figure_3_to_14/suppl_figure_3_chr01/suppl_figure_3bb_3_H_Kat.pdf]

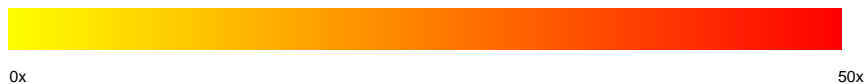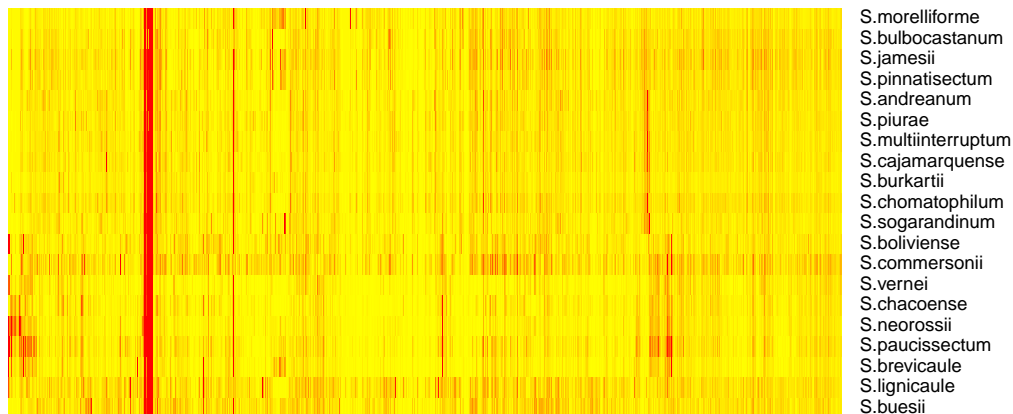

— TE — Gene — Non-aln • TanRep ♦ CenRep ■ rDNA

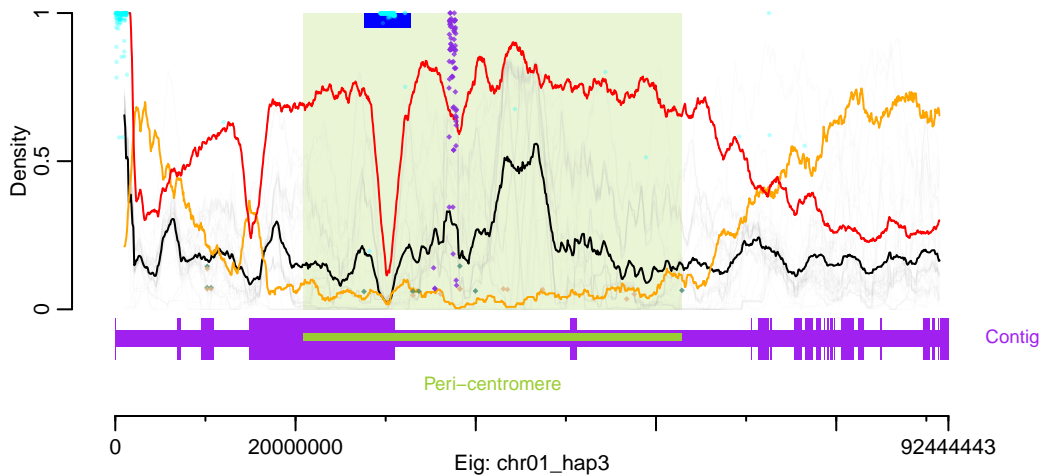

Supplement: Supplementary file 5 — Supplementary Figs. 3–14, 20, 21, 23–27 and 34–44. [file 41586_2025_8843_MOESM5_ESM.zip › suppl_figure_3_to_14/suppl_figure_3_chr01/suppl_figure_3au_3_C_EgH.pdf]

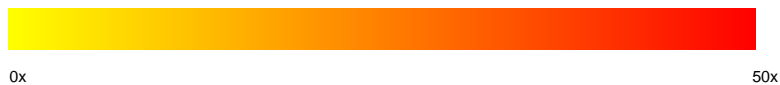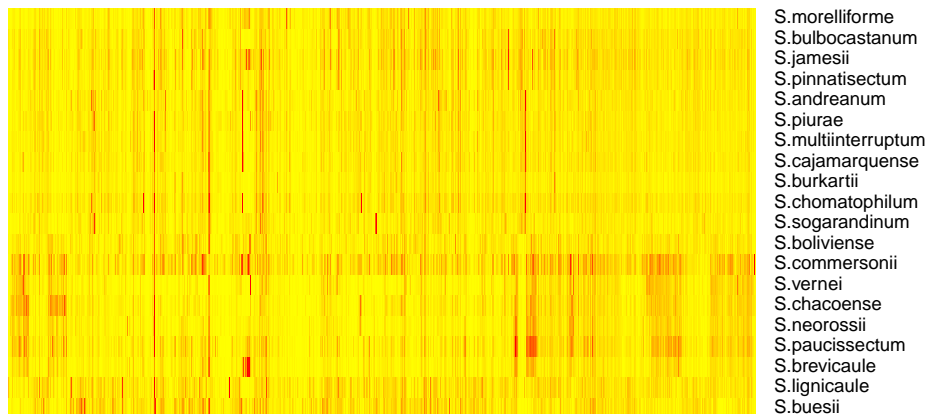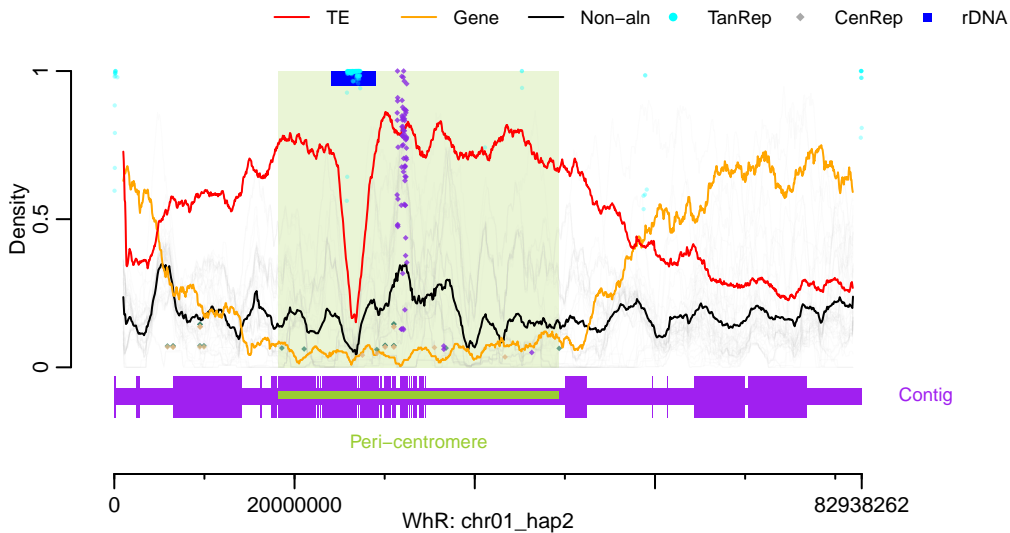

Supplement: Supplementary file 5 — Supplementary Figs. 3–14, 20, 21, 23–27 and 34–44. [file 41586_2025_8843_MOESM5_ESM.zip › suppl_figure_3_to_14/suppl_figure_3_chr01/suppl_figure_3ak_2_A_WhR.pdf]

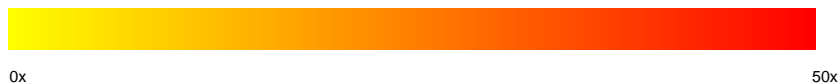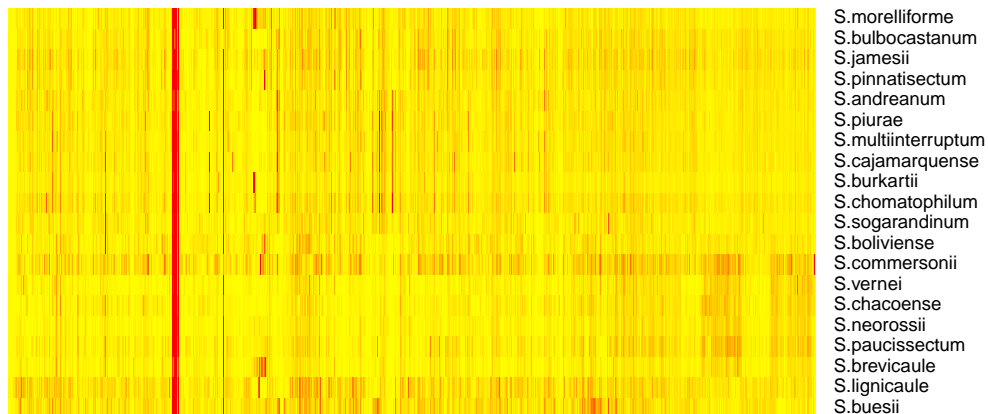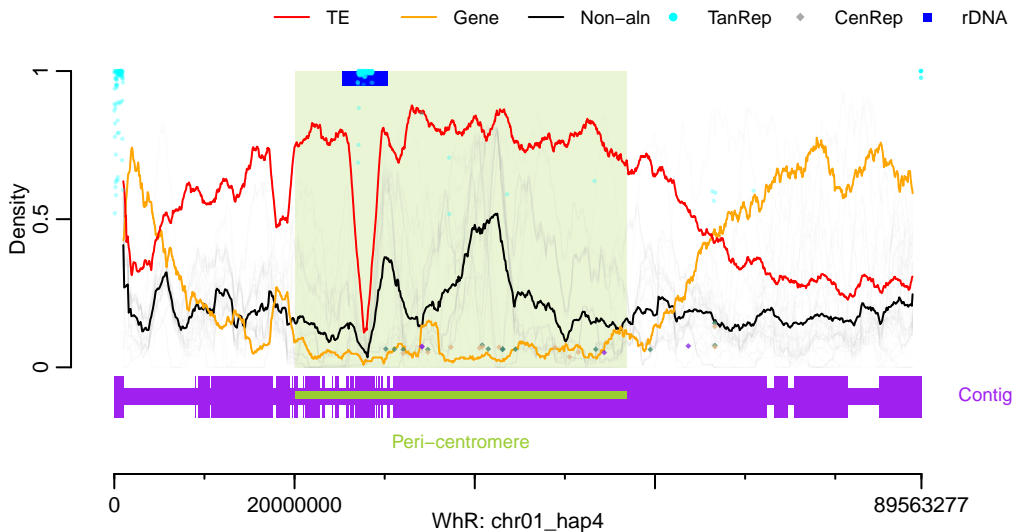

Supplement: Supplementary file 5 — Supplementary Figs. 3–14, 20, 21, 23–27 and 34–44. [file 41586_2025_8843_MOESM5_ESM.zip › suppl_figure_3_to_14/suppl_figure_3_chr01/suppl_figure_3be_4_A_WhR.pdf]

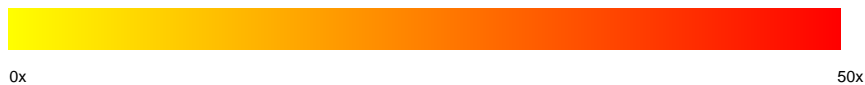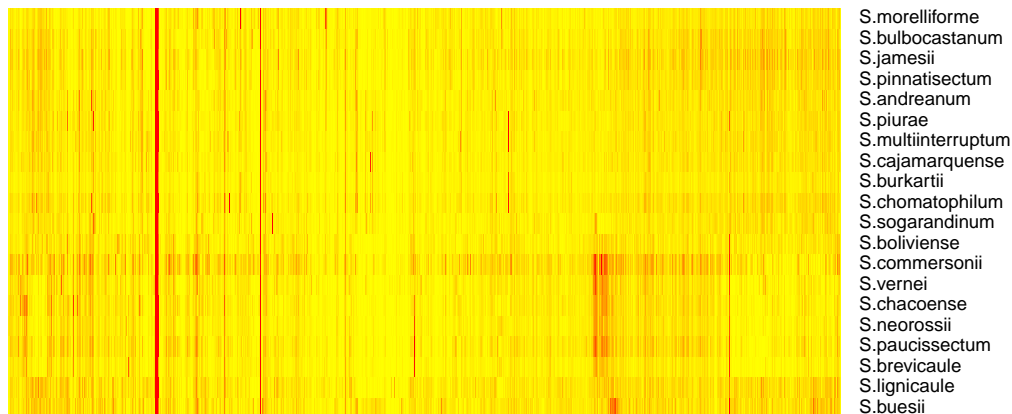

— TE — Gene — Non-aln • TanRep ♦ CenRep ■ rDNA

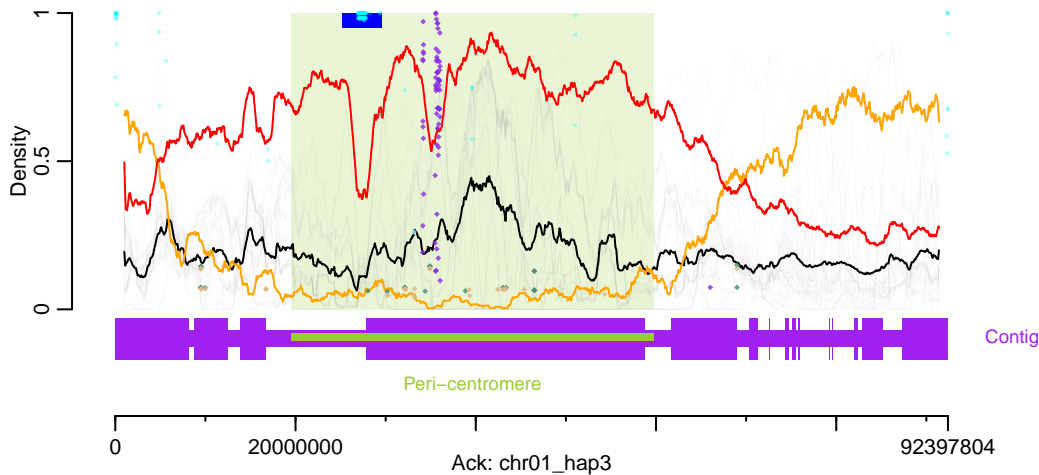

Supplement: Supplementary file 5 — Supplementary Figs. 3–14, 20, 21, 23–27 and 34–44. [file 41586_2025_8843_MOESM5_ESM.zip › suppl_figure_3_to_14/suppl_figure_3_chr01/suppl_figure_3az_3_F_Ack.pdf]

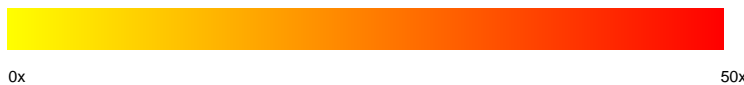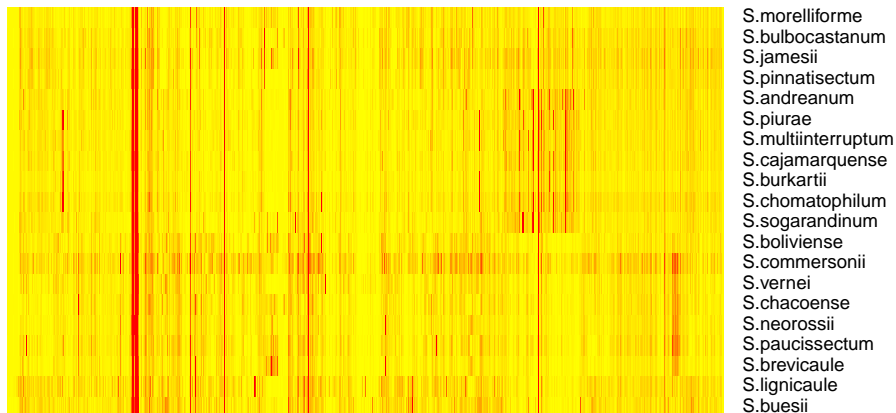

— TE — Gene — Non-aln • TanRep ♦ CenRep ■ rDNA

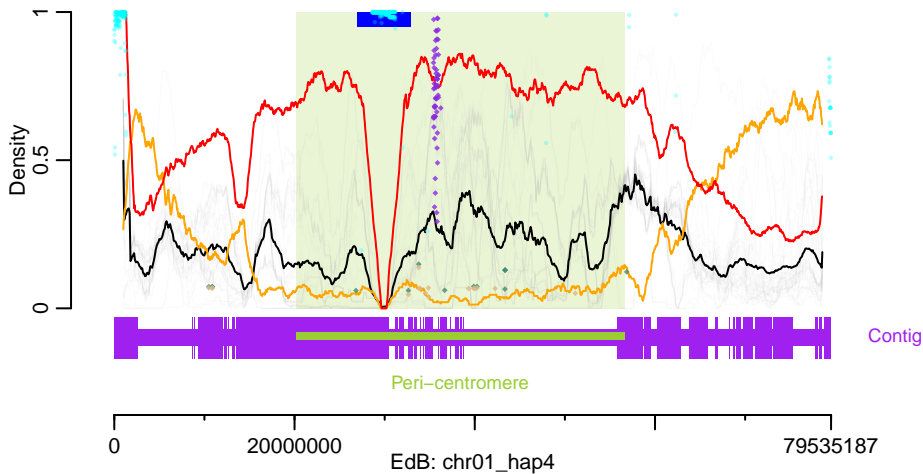

Supplement: Supplementary file 5 — Supplementary Figs. 3–14, 20, 21, 23–27 and 34–44. [file 41586_2025_8843_MOESM5_ESM.zip › suppl_figure_3_to_14/suppl_figure_3_chr01/suppl_figure_3bn_4_J_EdB.pdf]

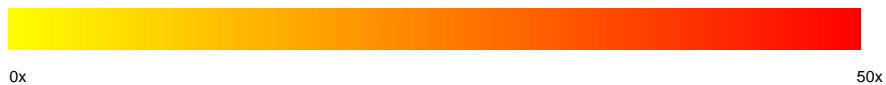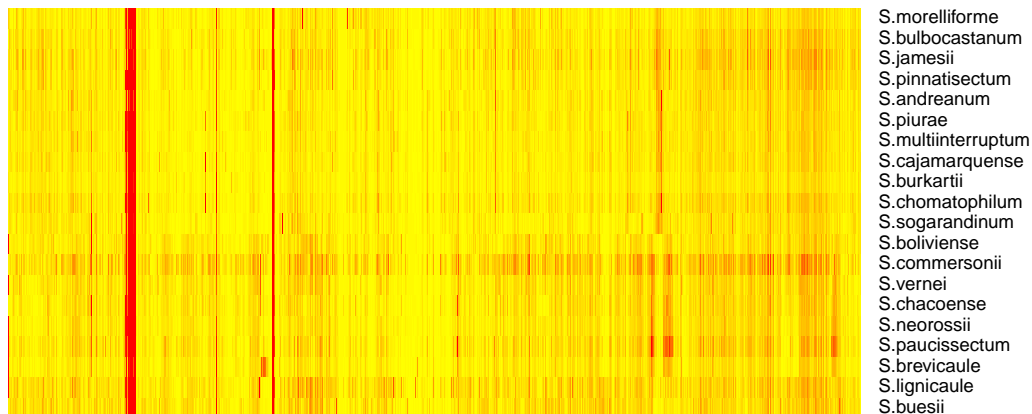

— TE — Gene — Non-aln • TanRep ♦ CenRep ■ rDNA

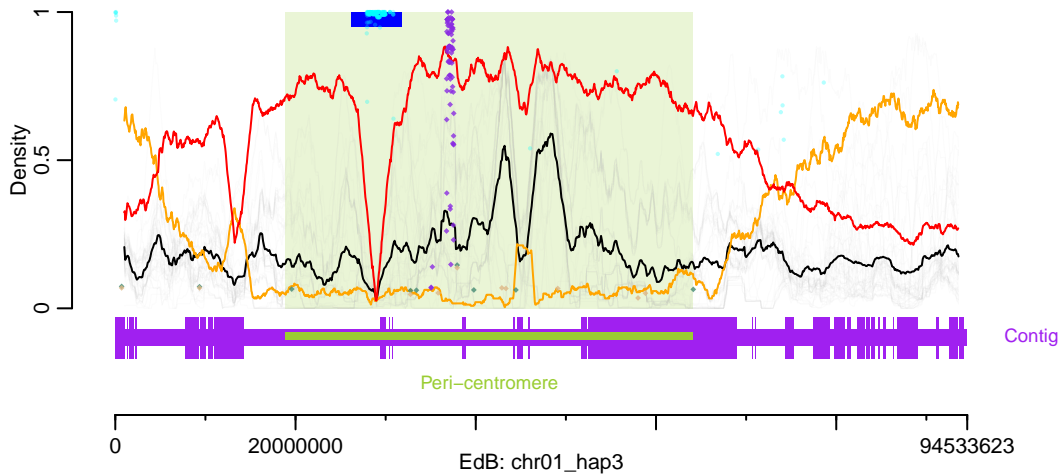

Supplement: Supplementary file 5 — Supplementary Figs. 3–14, 20, 21, 23–27 and 34–44. [file 41586_2025_8843_MOESM5_ESM.zip › suppl_figure_3_to_14/suppl_figure_3_chr01/suppl_figure_3bd_3_J_EdB.pdf]

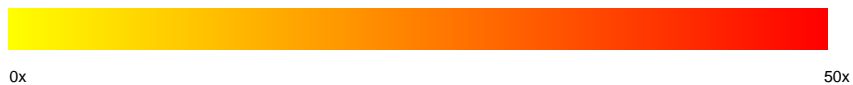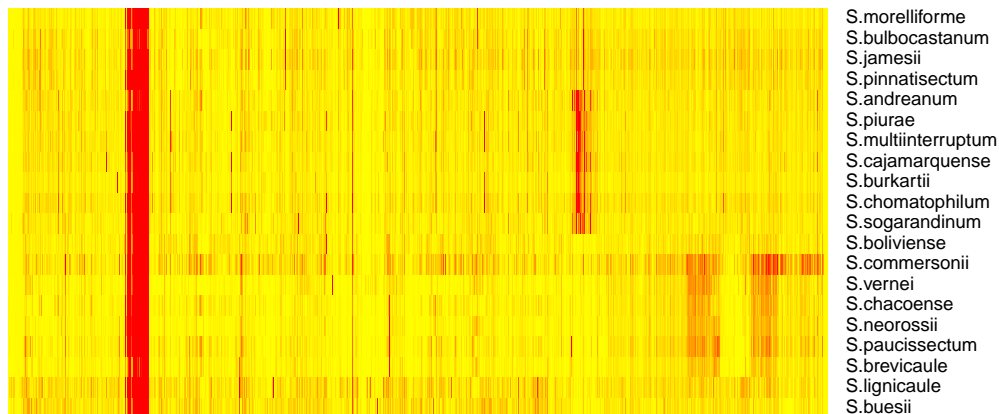

— TE — Gene — Non-aln • TanRep ♦ CenRep ■ rDNA

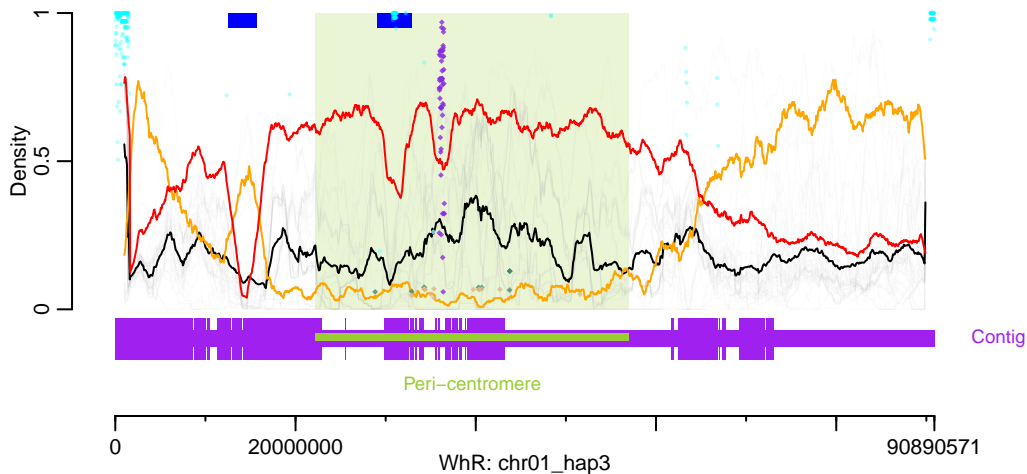

Supplement: Supplementary file 5 — Supplementary Figs. 3–14, 20, 21, 23–27 and 34–44. [file 41586_2025_8843_MOESM5_ESM.zip › suppl_figure_3_to_14/suppl_figure_3_chr01/suppl_figure_3aw_3_A_WhR.pdf]

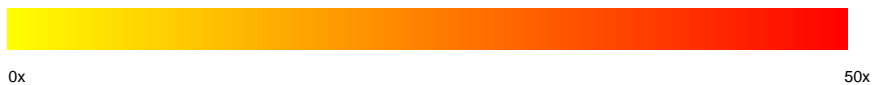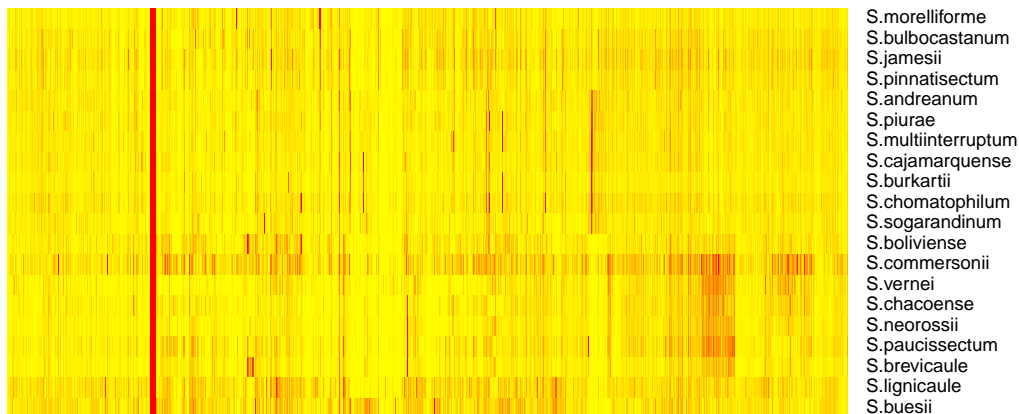

— TE — Gene — Non-aln • TanRep ♦ CenRep ■ rDNA

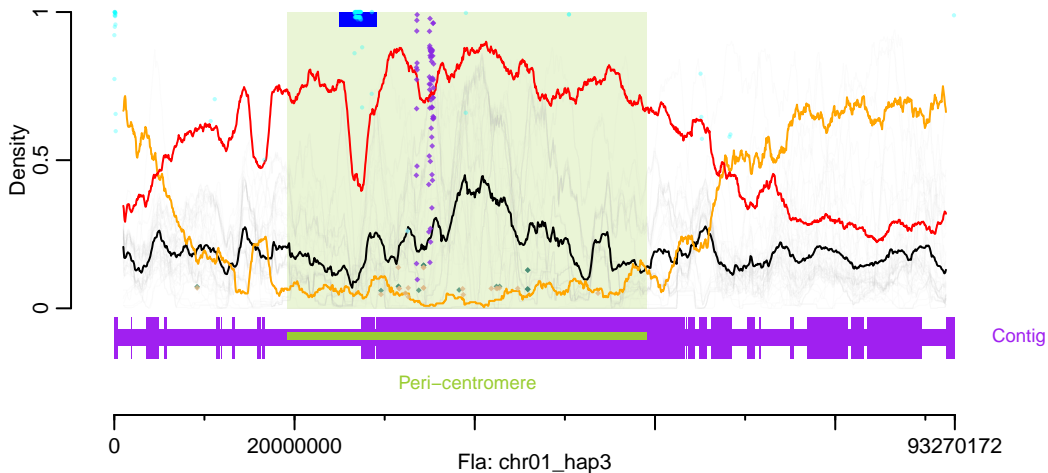

Supplement: Supplementary file 5 — Supplementary Figs. 3–14, 20, 21, 23–27 and 34–44. [file 41586_2025_8843_MOESM5_ESM.zip › suppl_figure_3_to_14/suppl_figure_3_chr01/suppl_figure_3ba_3_G_Fla.pdf]

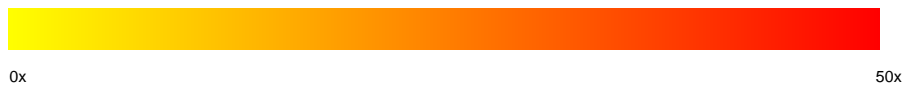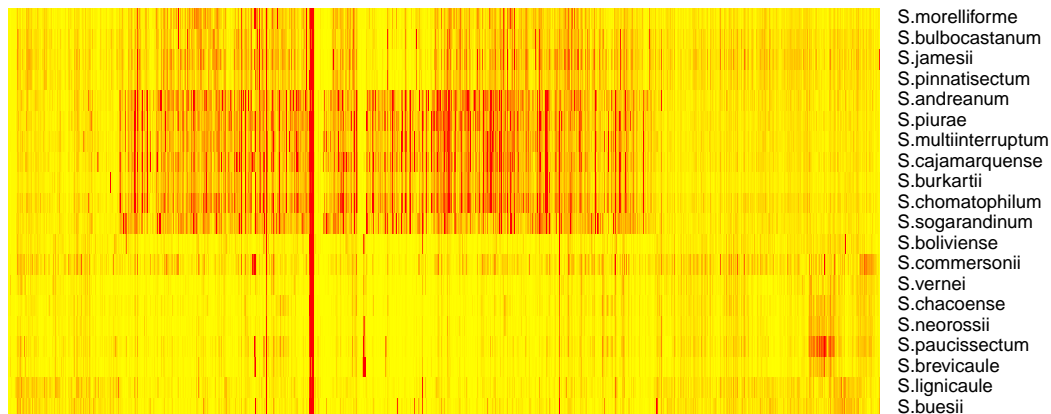

— TE — Gene — Non-aln • TanRep ♦ CenRep ■ rDNA

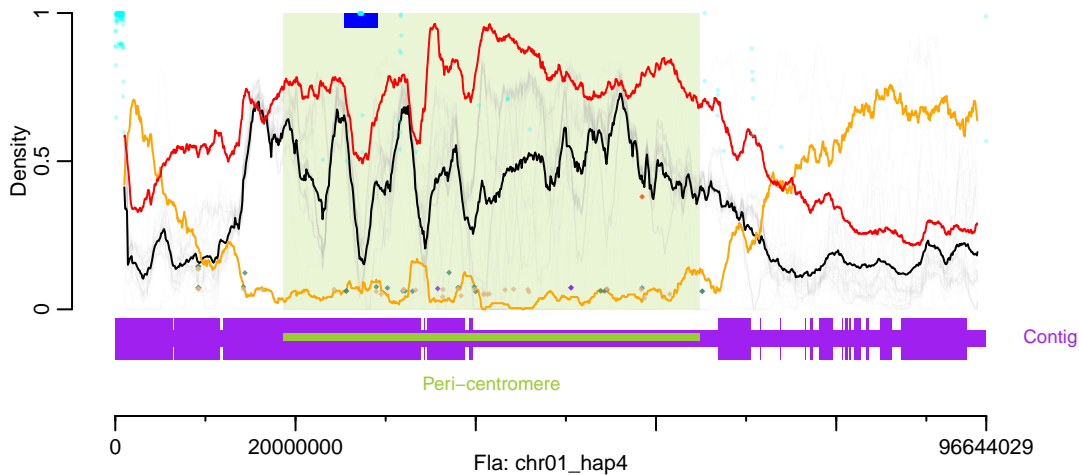

Supplement: Supplementary file 5 — Supplementary Figs. 3–14, 20, 21, 23–27 and 34–44. [file 41586_2025_8843_MOESM5_ESM.zip › suppl_figure_3_to_14/suppl_figure_3_chr01/suppl_figure_3bk_4_G_Fla.pdf]

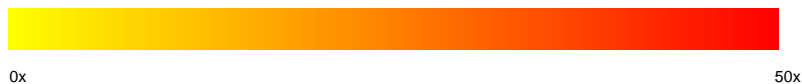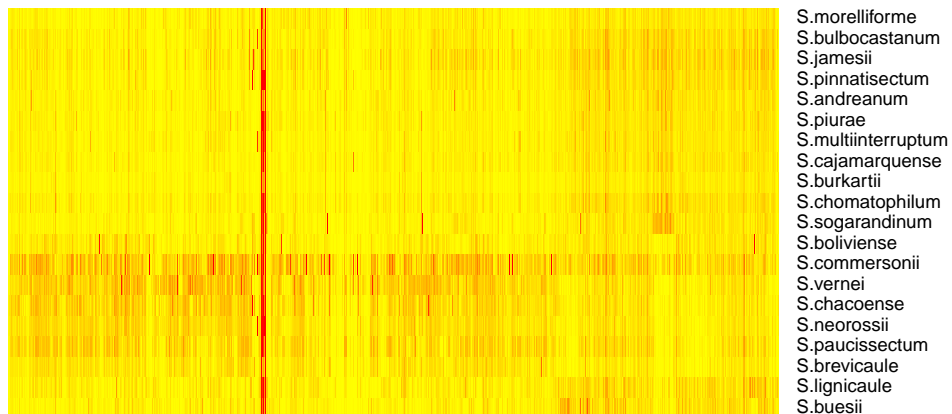

— TE — Gene — Non-aln • TanRep ♦ CenRep ■ rDNA

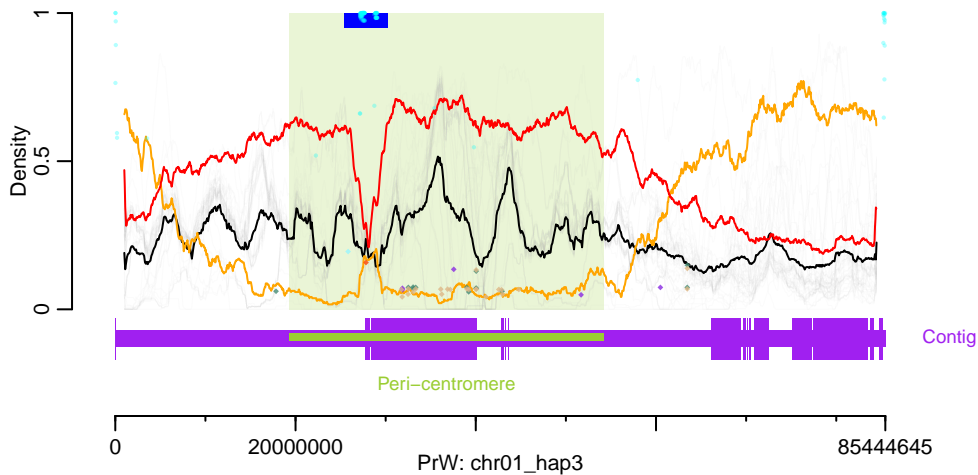

Supplement: Supplementary file 5 — Supplementary Figs. 3–14, 20, 21, 23–27 and 34–44. [file 41586_2025_8843_MOESM5_ESM.zip › suppl_figure_3_to_14/suppl_figure_3_chr01/suppl_figure_3ax_3_D_PrW.pdf]

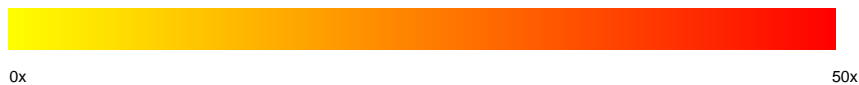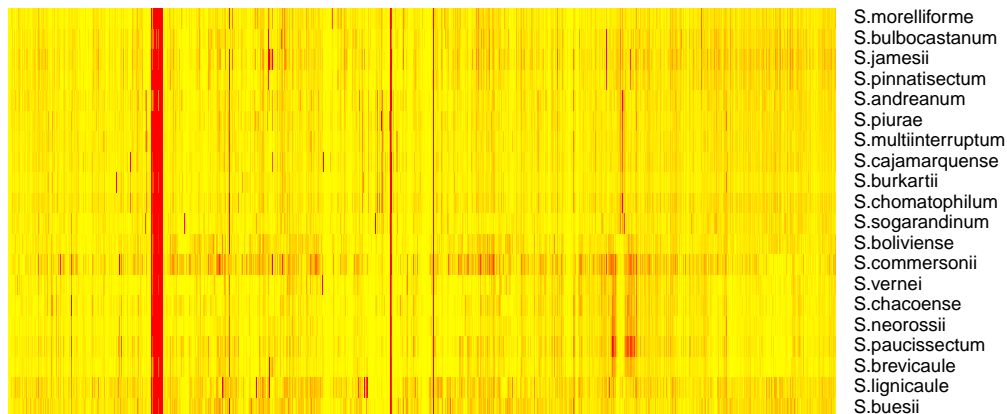

— TE — Gene — Non-aln ● TanRep ◆ CenRep ■ rDNA

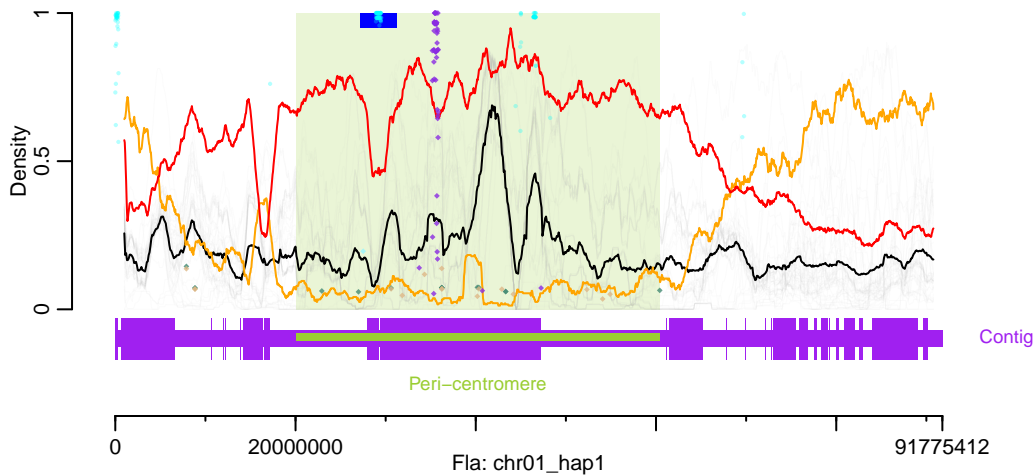

Supplement: Supplementary file 5 — Supplementary Figs. 3–14, 20, 21, 23–27 and 34–44. [file 41586_2025_8843_MOESM5_ESM.zip › suppl_figure_3_to_14/suppl_figure_3_chr01/suppl_figure_3ag_1_G_Fla.pdf]

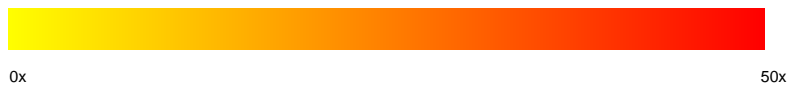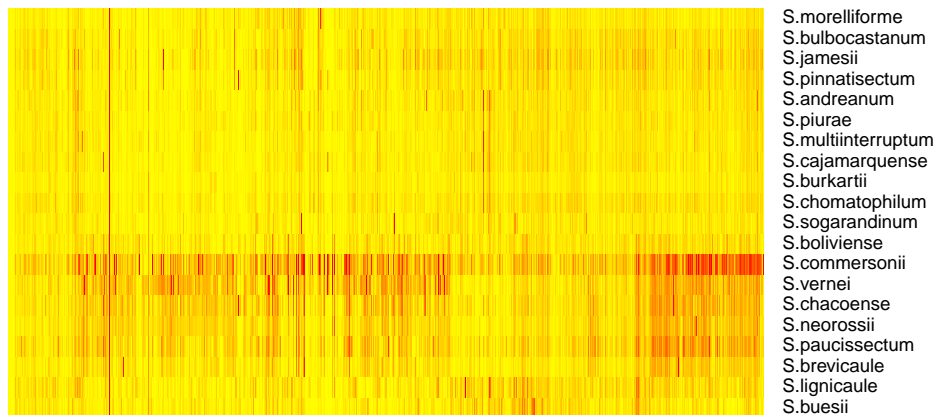

— TE — Gene — Non-aln • TanRep ♦ CenRep ■ rDNA

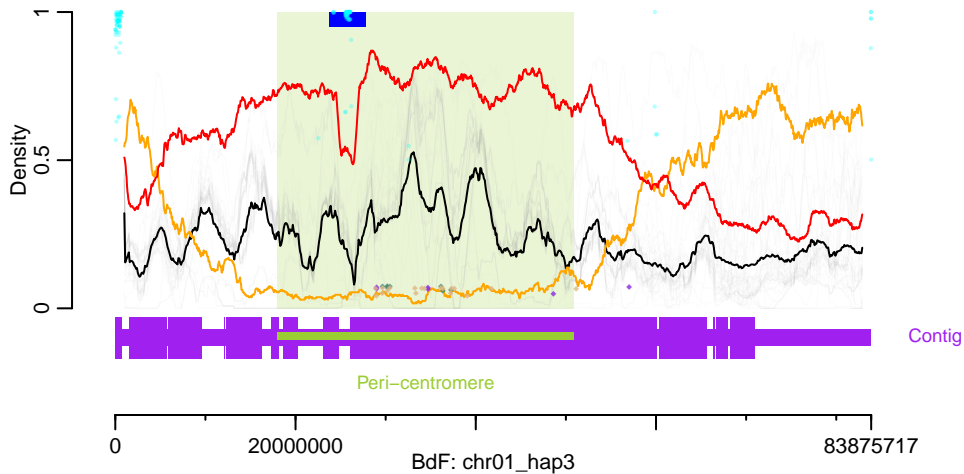

Supplement: Supplementary file 5 — Supplementary Figs. 3–14, 20, 21, 23–27 and 34–44. [file 41586_2025_8843_MOESM5_ESM.zip › suppl_figure_3_to_14/suppl_figure_3_chr01/suppl_figure_3av_3_B_BdF.pdf]

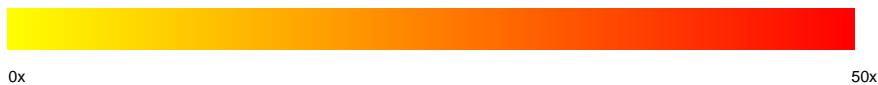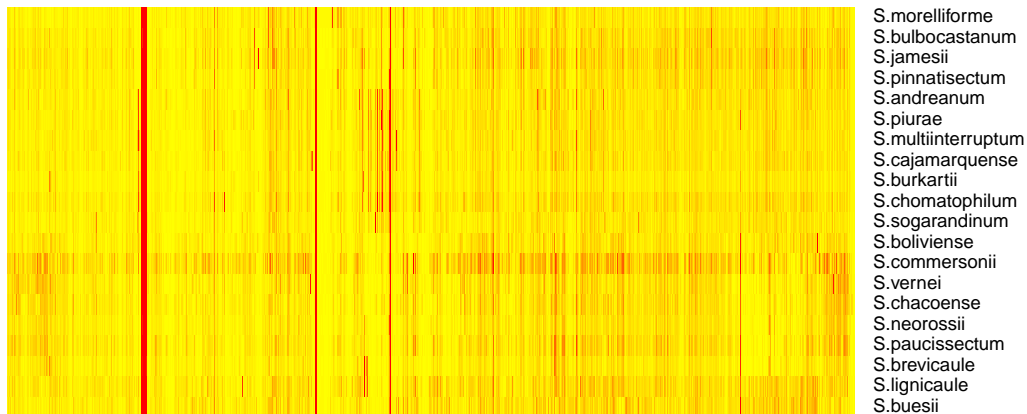

— TE — Gene — Non-aln • TanRep ♦ CenRep ■ rDNA

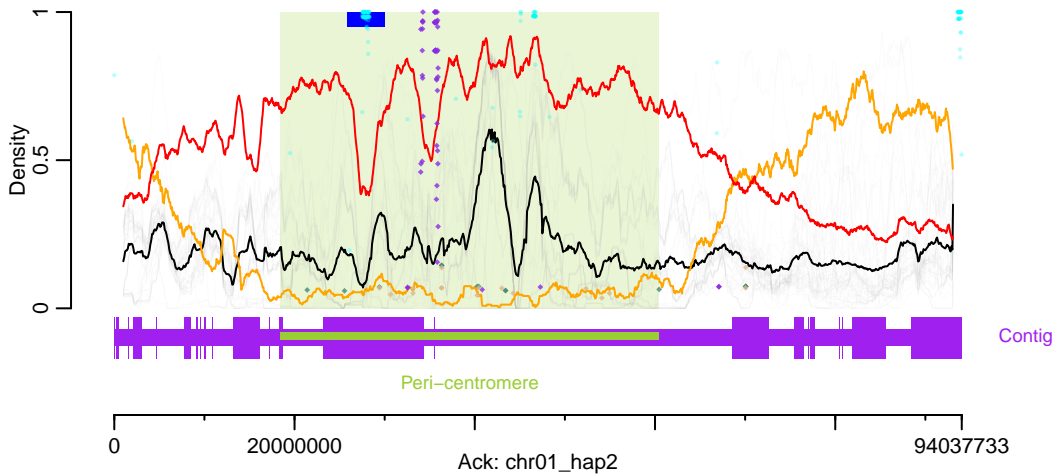

Supplement: Supplementary file 5 — Supplementary Figs. 3–14, 20, 21, 23–27 and 34–44. [file 41586_2025_8843_MOESM5_ESM.zip › suppl_figure_3_to_14/suppl_figure_3_chr01/suppl_figure_3ap_2_F_Ack.pdf]

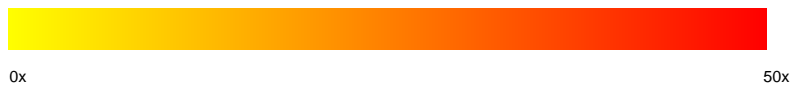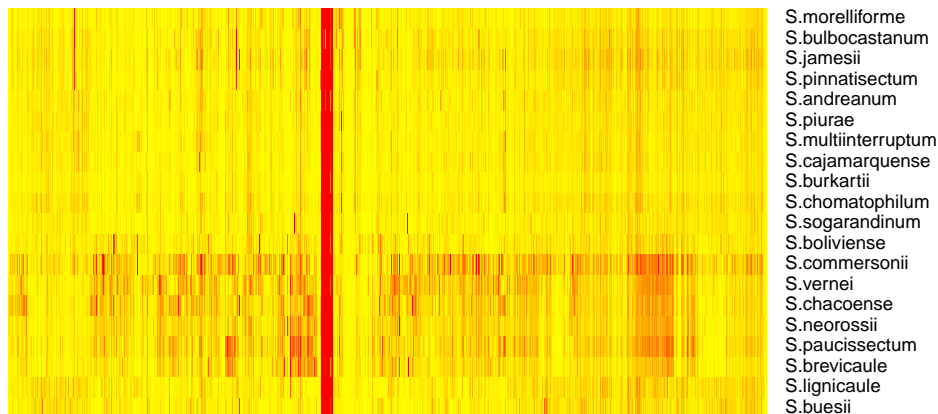

— TE — Gene — Non-aln • TanRep ♦ CenRep ■ rDNA

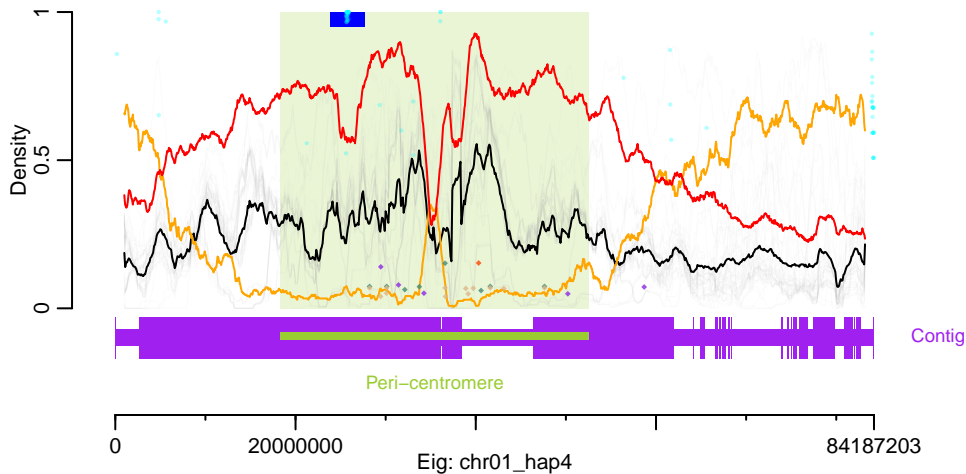

Supplement: Supplementary file 5 — Supplementary Figs. 3–14, 20, 21, 23–27 and 34–44. [file 41586_2025_8843_MOESM5_ESM.zip › suppl_figure_3_to_14/suppl_figure_3_chr01/suppl_figure_3bg_4_C_EgH.pdf]

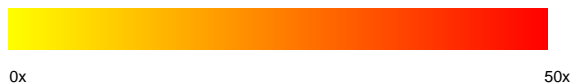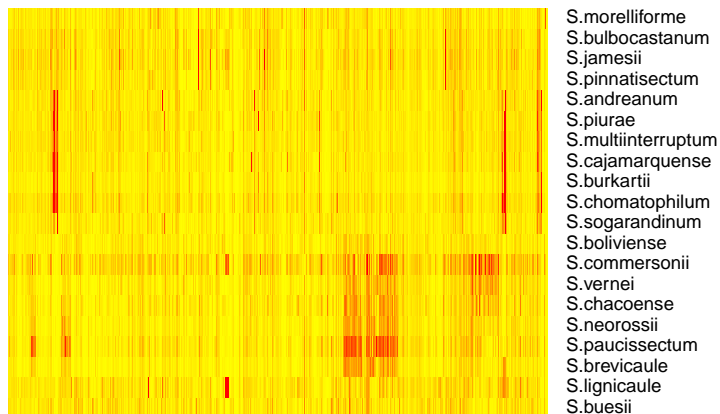

— TE — Gene — Non-aln • TanRep ♦ CenRep ■ rDNA

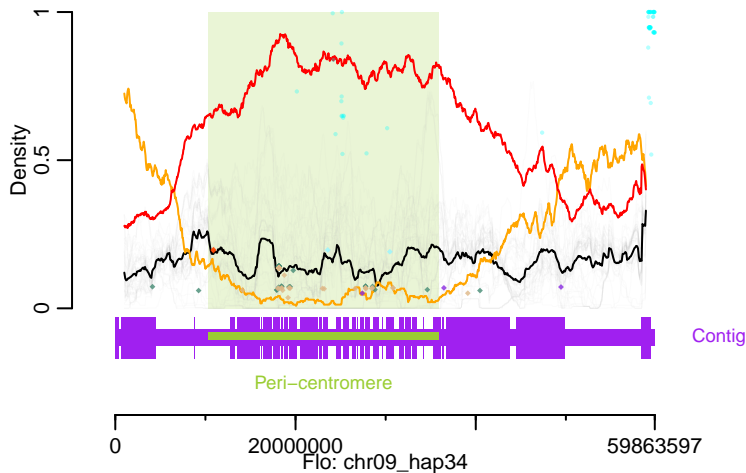

Supplement: Supplementary file 5 — Supplementary Figs. 3–14, 20, 21, 23–27 and 34–44. [file 41586_2025_8843_MOESM5_ESM.zip › suppl_figure_3_to_14/suppl_figure_11_chr09/suppl_figure_11ao_34_E_Flo.pdf]

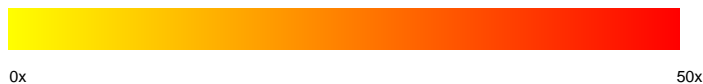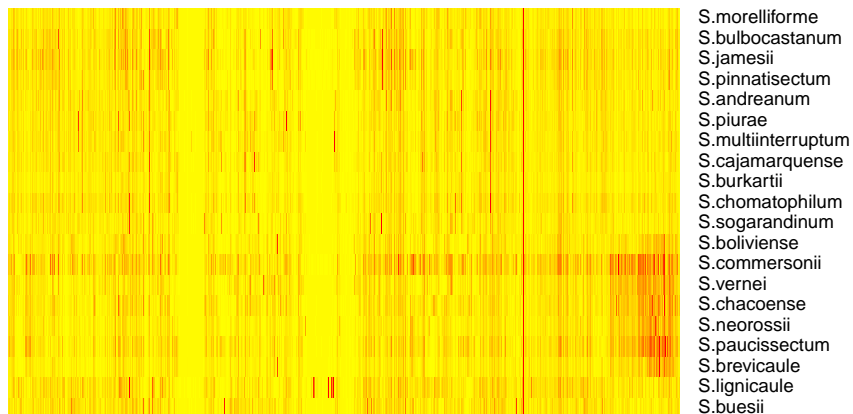

— TE — Gene — Non-aln • TanRep ♦ CenRep ■ rDNA

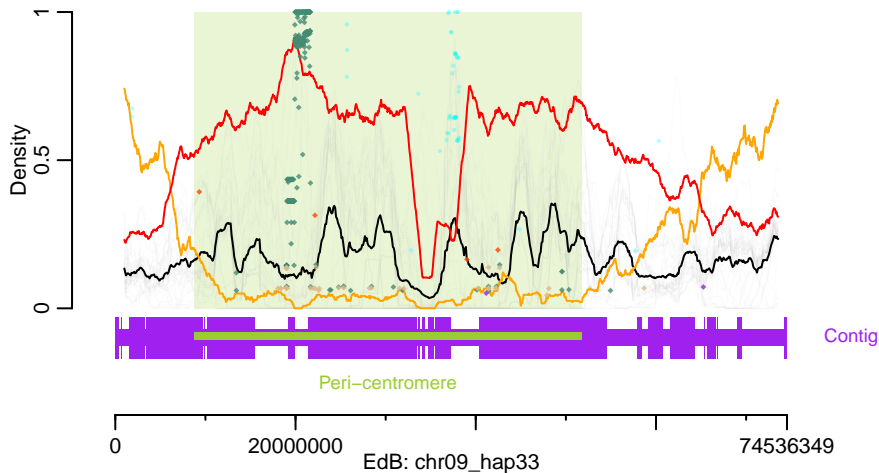

Supplement: Supplementary file 5 — Supplementary Figs. 3–14, 20, 21, 23–27 and 34–44. [file 41586_2025_8843_MOESM5_ESM.zip › suppl_figure_3_to_14/suppl_figure_11_chr09/suppl_figure_11aj_33_J_EdB.pdf]

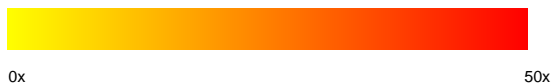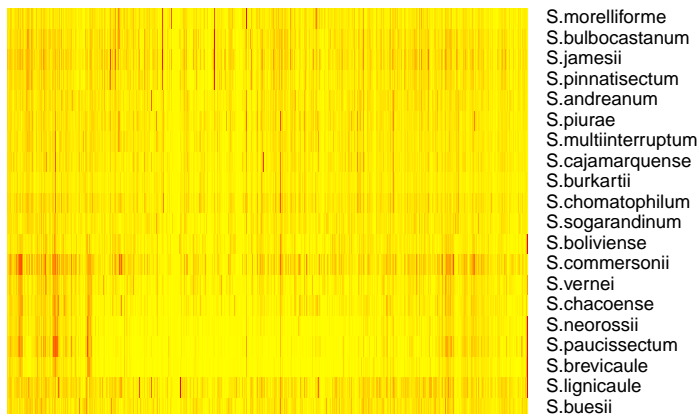

— TE — Gene — Non-aln ● TanRep ♦ CenRep ■ rDNA

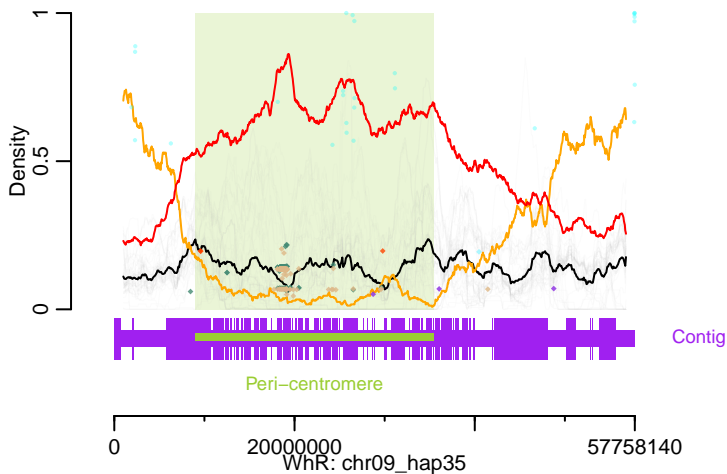

Supplement: Supplementary file 5 — Supplementary Figs. 3–14, 20, 21, 23–27 and 34–44. [file 41586_2025_8843_MOESM5_ESM.zip › suppl_figure_3_to_14/suppl_figure_11_chr09/suppl_figure_11aw_35_A_WhR.pdf]

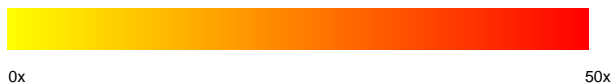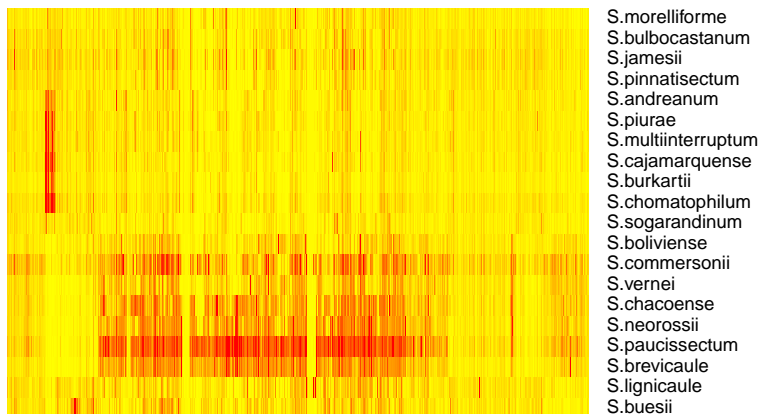

— TE — Gene — Non-aln • TanRep ♦ CenRep ■ rDNA

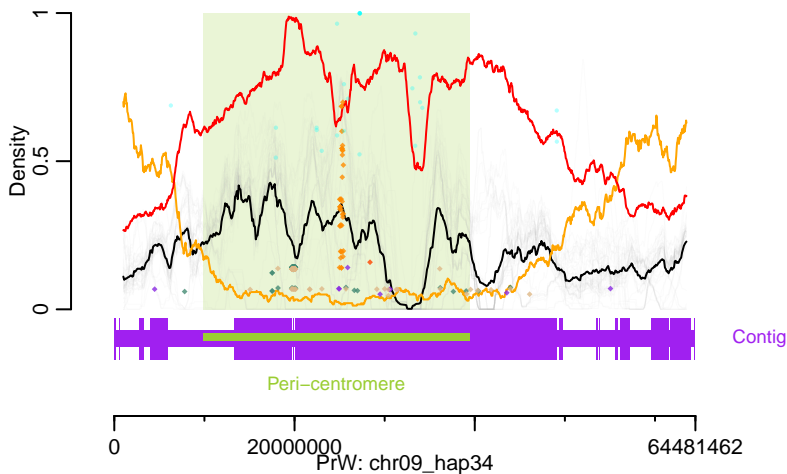

Supplement: Supplementary file 5 — Supplementary Figs. 3–14, 20, 21, 23–27 and 34–44. [file 41586_2025_8843_MOESM5_ESM.zip › suppl_figure_3_to_14/suppl_figure_11_chr09/suppl_figure_11an_34_D_PrW.pdf]

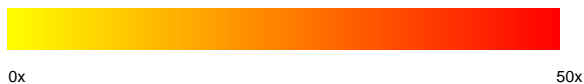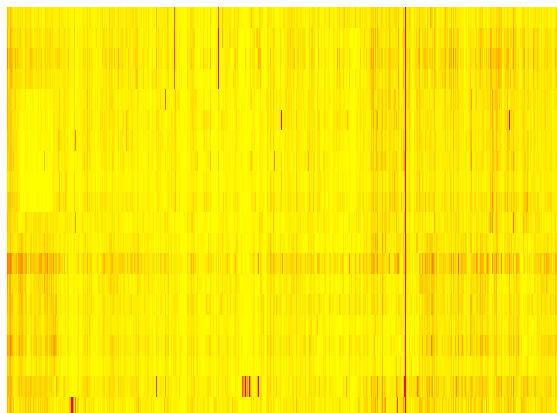

— TE — Gene — Non-aln ● TanRep ♦ CenRep ■ rDNA

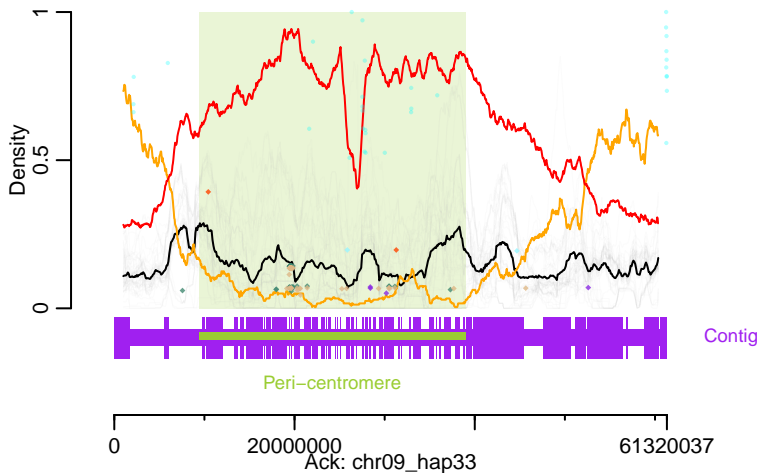

Supplement: Supplementary file 5 — Supplementary Figs. 3–14, 20, 21, 23–27 and 34–44. [file 41586_2025_8843_MOESM5_ESM.zip › suppl_figure_3_to_14/suppl_figure_11_chr09/suppl_figure_11af_33_F_Ack.pdf]

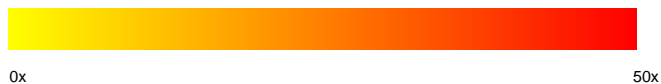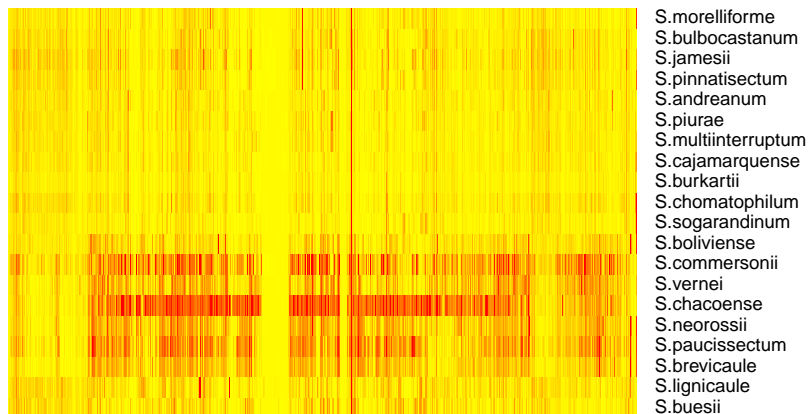

— TE — Gene — Non-aln • TanRep ♦ CenRep ■ rDNA

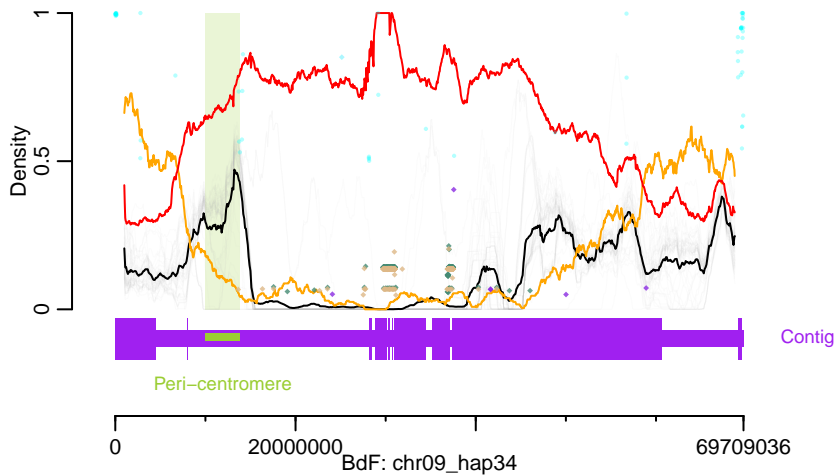

Supplement: Supplementary file 5 — Supplementary Figs. 3–14, 20, 21, 23–27 and 34–44. [file 41586_2025_8843_MOESM5_ESM.zip › suppl_figure_3_to_14/suppl_figure_11_chr09/suppl_figure_11al_34_B_BdF.pdf]

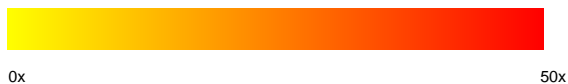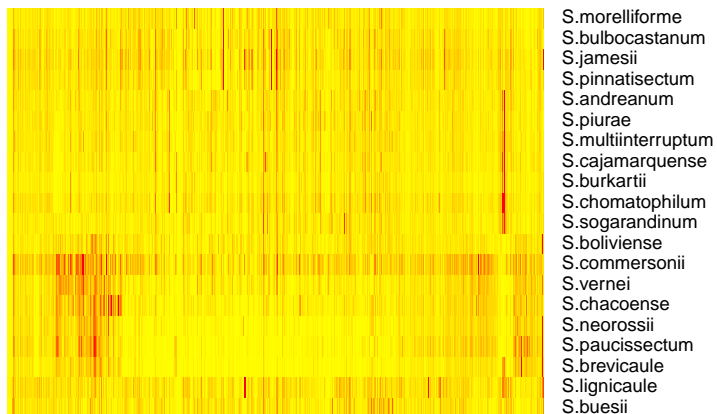

— TE — Gene — Non-aln • TanRep ♦ CenRep ■ rDNA

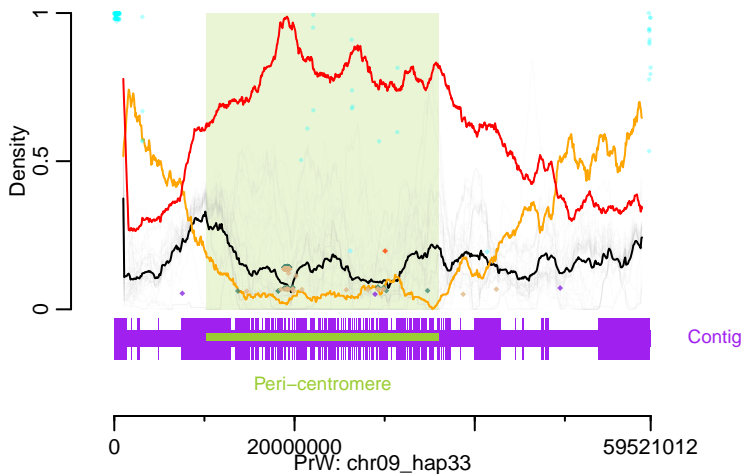

Supplement: Supplementary file 5 — Supplementary Figs. 3–14, 20, 21, 23–27 and 34–44. [file 41586_2025_8843_MOESM5_ESM.zip › suppl_figure_3_to_14/suppl_figure_11_chr09/suppl_figure_11ad_33_D_PrW.pdf]

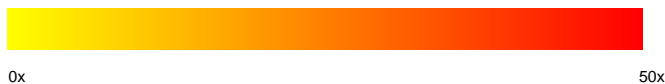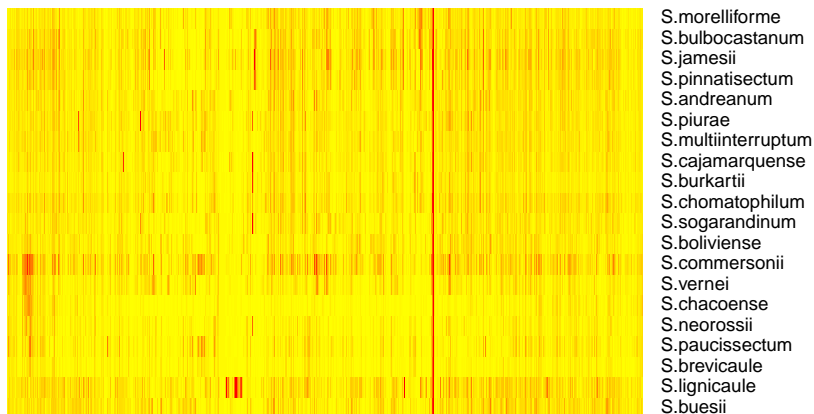

— TE — Gene — Non-aln • TanRep ♦ CenRep ■ rDNA

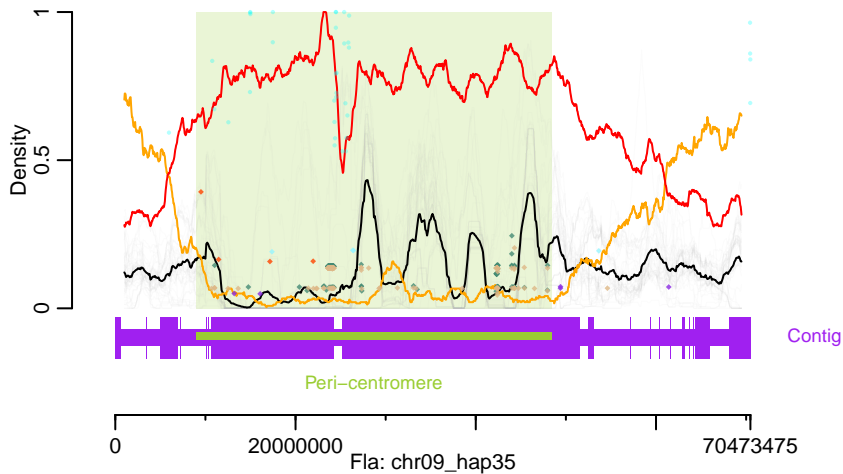

Supplement: Supplementary file 5 — Supplementary Figs. 3–14, 20, 21, 23–27 and 34–44. [file 41586_2025_8843_MOESM5_ESM.zip › suppl_figure_3_to_14/suppl_figure_11_chr09/suppl_figure_11ba_35_G_Fla.pdf]

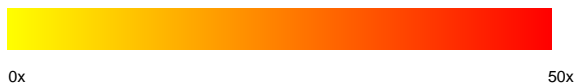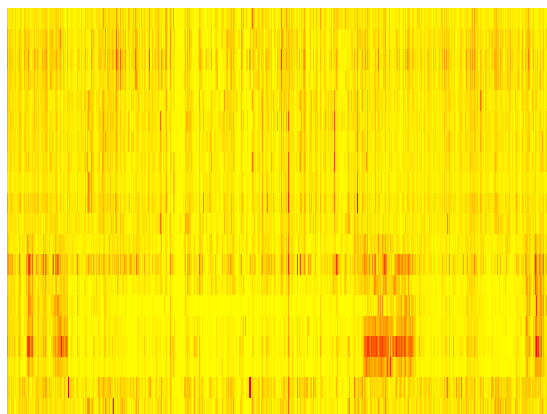

— TE — Gene — Non-aln • TanRep ♦ CenRep ■ rDNA

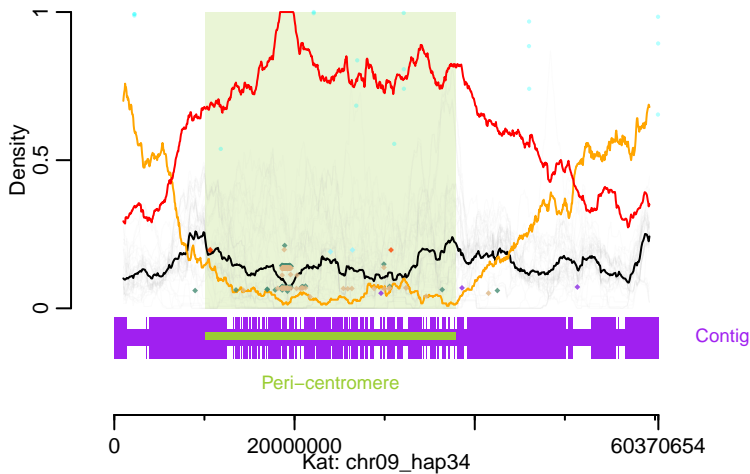

Supplement: Supplementary file 5 — Supplementary Figs. 3–14, 20, 21, 23–27 and 34–44. [file 41586_2025_8843_MOESM5_ESM.zip › suppl_figure_3_to_14/suppl_figure_11_chr09/suppl_figure_11ar_34_H_Kat.pdf]

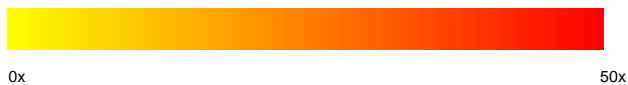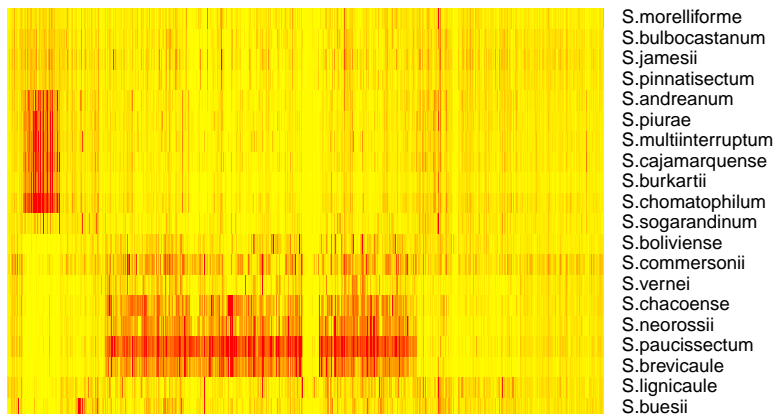

— TE — Gene — Non-aln • TanRep ♦ CenRep ■ rDNA

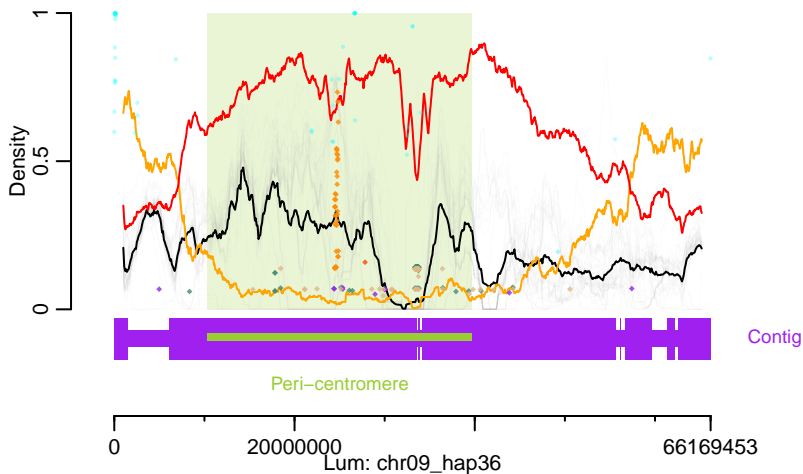

Supplement: Supplementary file 5 — Supplementary Figs. 3–14, 20, 21, 23–27 and 34–44. [file 41586_2025_8843_MOESM5_ESM.zip › suppl_figure_3_to_14/suppl_figure_11_chr09/suppl_figure_11bm_36_I_Lum.pdf]

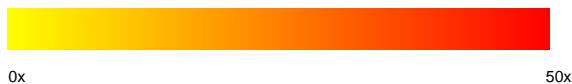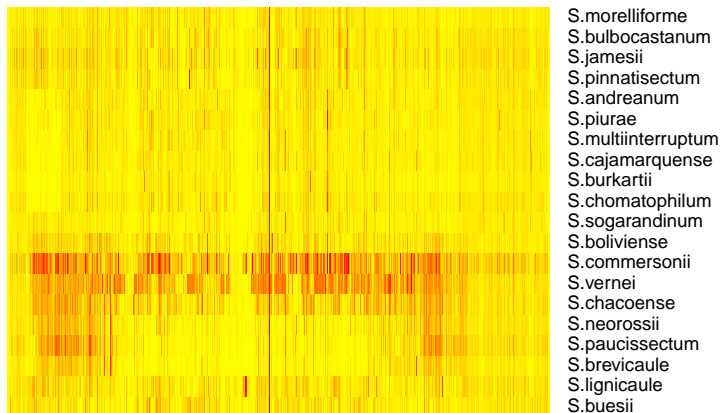

— TE — Gene — Non-aln • TanRep ♦ CenRep ■ rDNA

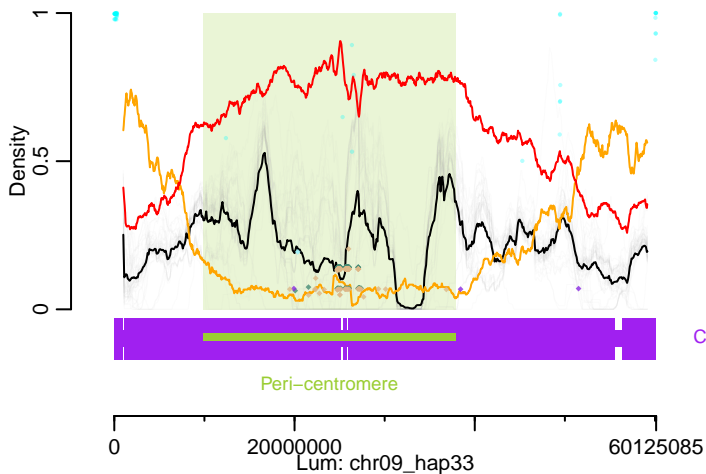

Supplement: Supplementary file 5 — Supplementary Figs. 3–14, 20, 21, 23–27 and 34–44. [file 41586_2025_8843_MOESM5_ESM.zip › suppl_figure_3_to_14/suppl_figure_11_chr09/suppl_figure_11ai_33_I_Lum.pdf]

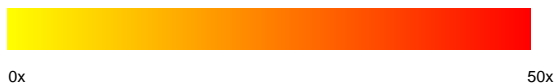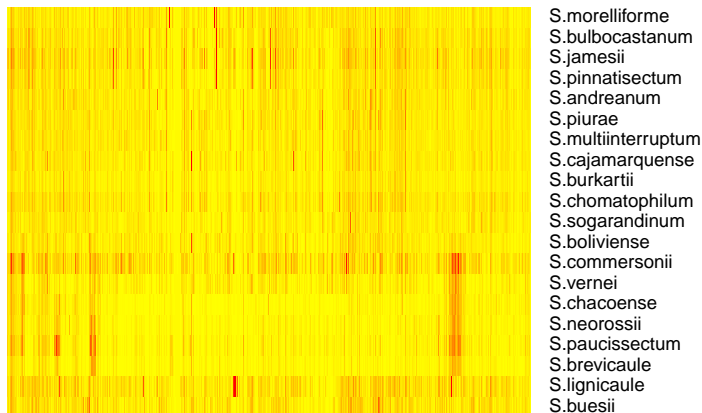

— TE — Gene — Non-aln ● TanRep ♦ CenRep ■ rDNA

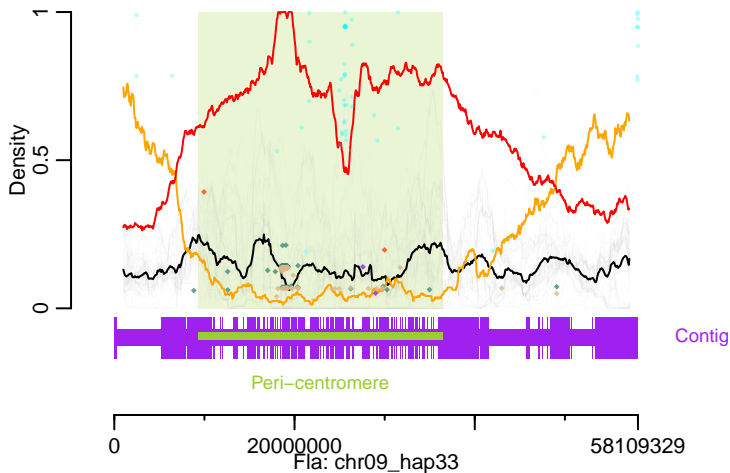

Supplement: Supplementary file 5 — Supplementary Figs. 3–14, 20, 21, 23–27 and 34–44. [file 41586_2025_8843_MOESM5_ESM.zip › suppl_figure_3_to_14/suppl_figure_11_chr09/suppl_figure_11ag_33_G_Fla.pdf]

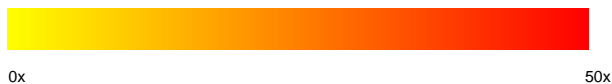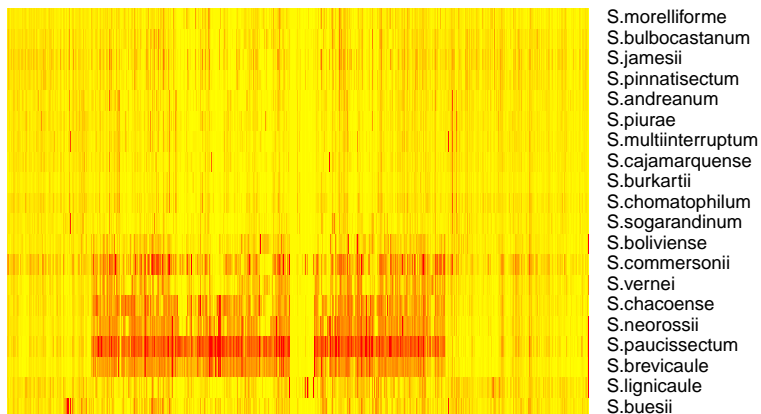

— TE — Gene — Non-aln • TanRep ♦ CenRep ■ rDNA

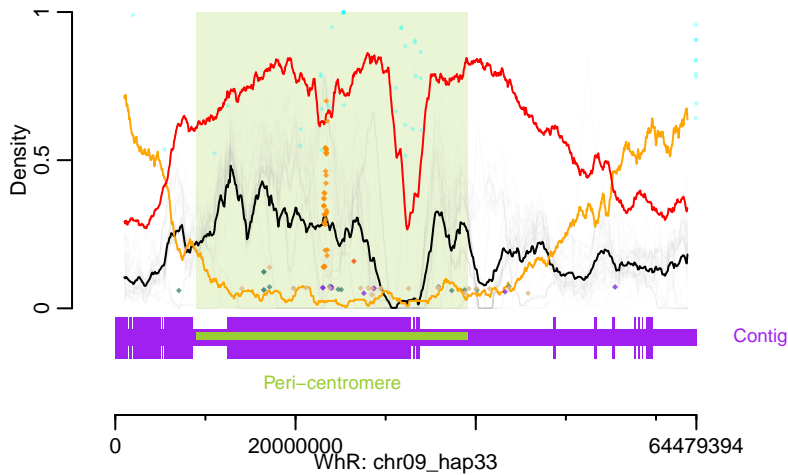

Supplement: Supplementary file 5 — Supplementary Figs. 3–14, 20, 21, 23–27 and 34–44. [file 41586_2025_8843_MOESM5_ESM.zip › suppl_figure_3_to_14/suppl_figure_11_chr09/suppl_figure_11aa_33_A_WhR.pdf]

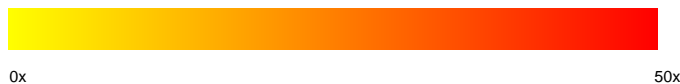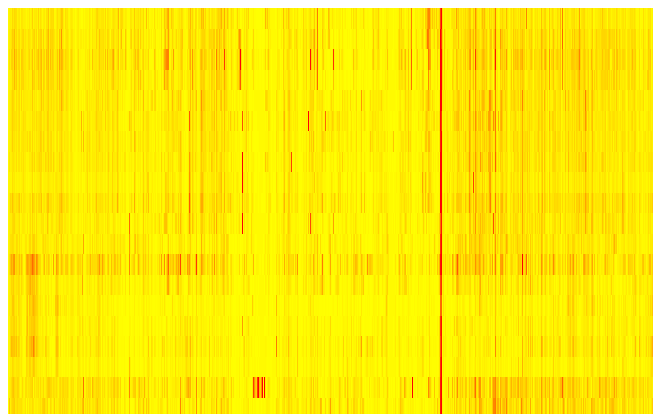

— TE — Gene — Non-aln • TanRep ♦ CenRep ■ rDNA

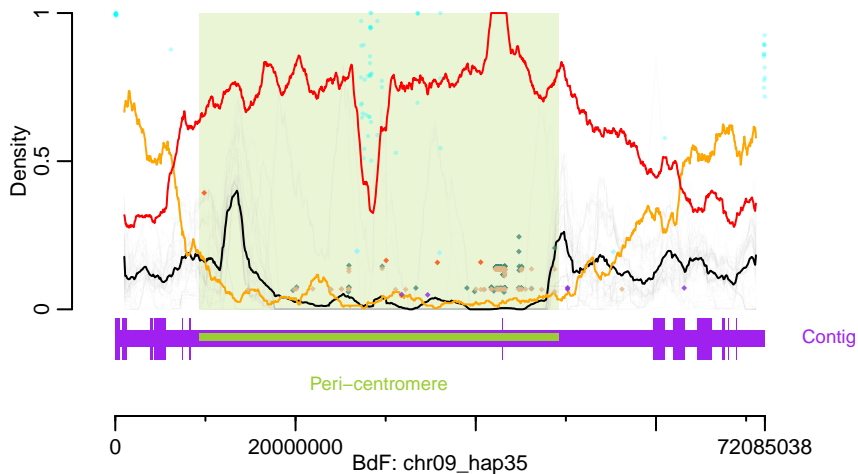

Supplement: Supplementary file 5 — Supplementary Figs. 3–14, 20, 21, 23–27 and 34–44. [file 41586_2025_8843_MOESM5_ESM.zip › suppl_figure_3_to_14/suppl_figure_11_chr09/suppl_figure_11av_35_B_BdF.pdf]

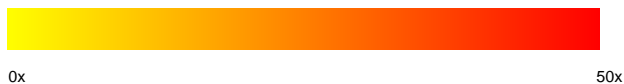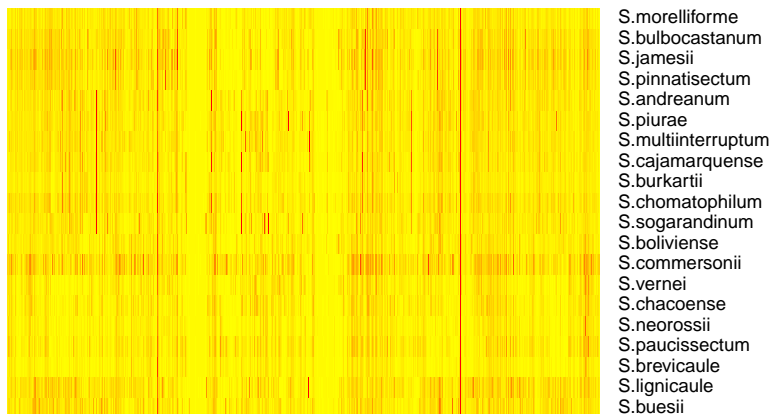

— TE — Gene — Non-aln • TanRep ♦ CenRep ■ rDNA

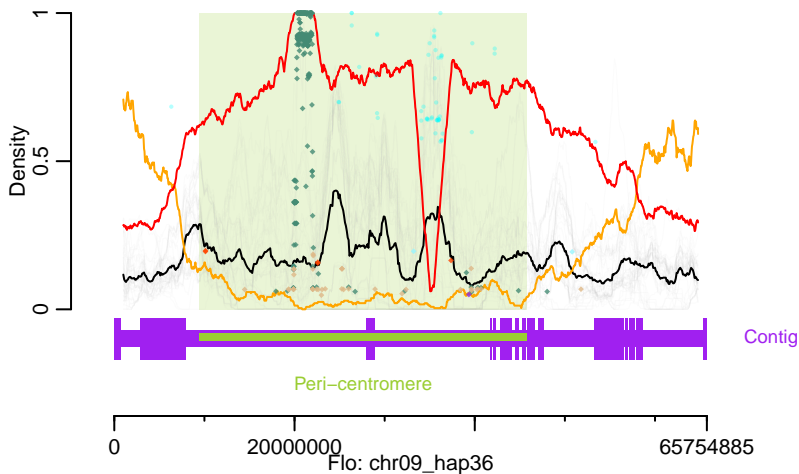

Supplement: Supplementary file 5 — Supplementary Figs. 3–14, 20, 21, 23–27 and 34–44. [file 41586_2025_8843_MOESM5_ESM.zip › suppl_figure_3_to_14/suppl_figure_11_chr09/suppl_figure_11bi_36_E_Flo.pdf]

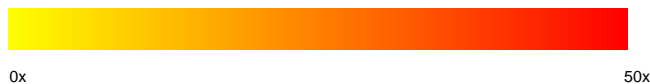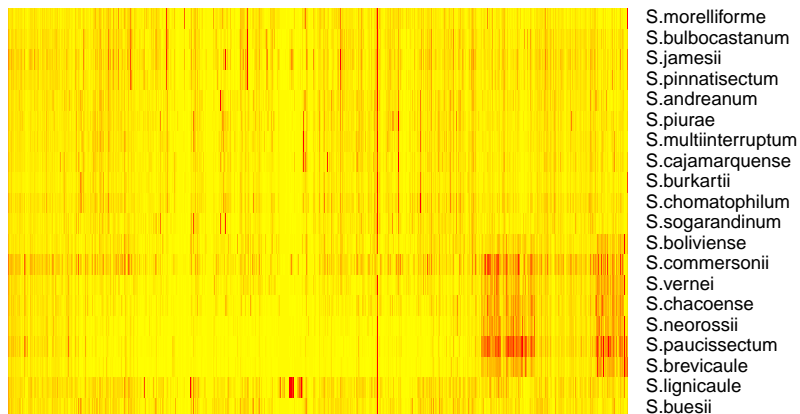

— TE — Gene — Non-aln • TanRep ♦ CenRep ■ rDNA

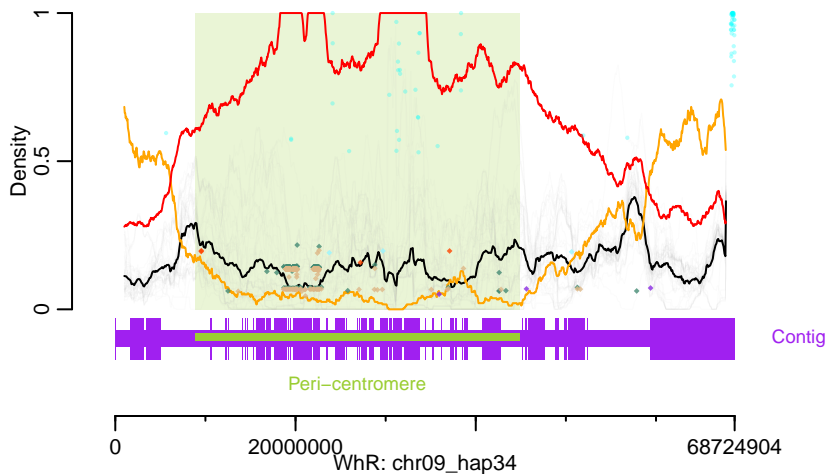

Supplement: Supplementary file 5 — Supplementary Figs. 3–14, 20, 21, 23–27 and 34–44. [file 41586_2025_8843_MOESM5_ESM.zip › suppl_figure_3_to_14/suppl_figure_11_chr09/suppl_figure_11ak_34_A_WhR.pdf]

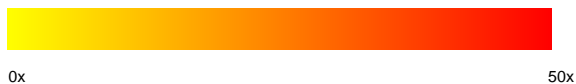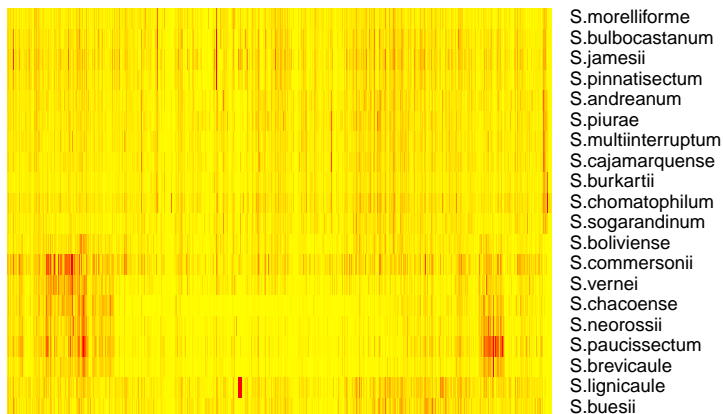

— TE — Gene — Non-aln • TanRep ♦ CenRep ■ rDNA

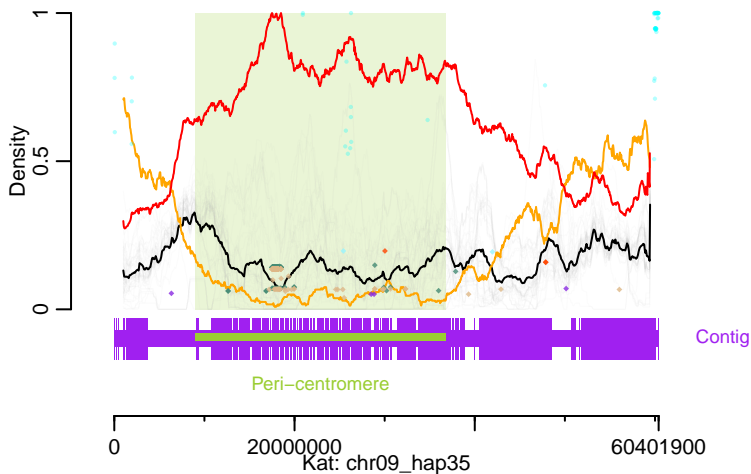

Supplement: Supplementary file 5 — Supplementary Figs. 3–14, 20, 21, 23–27 and 34–44. [file 41586_2025_8843_MOESM5_ESM.zip › suppl_figure_3_to_14/suppl_figure_11_chr09/suppl_figure_11bb_35_H_Kat.pdf]

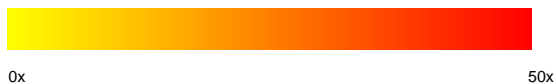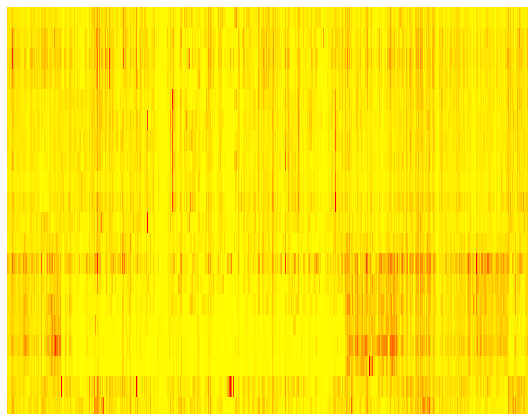

— TE — Gene — Non-aln • TanRep ♦ CenRep ■ rDNA

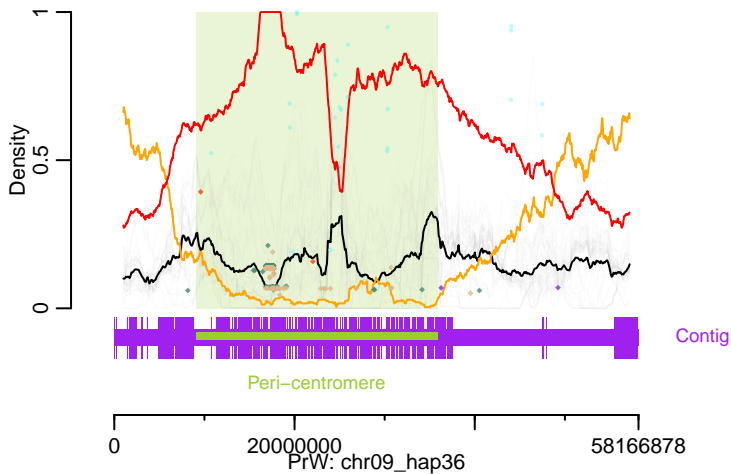

Supplement: Supplementary file 5 — Supplementary Figs. 3–14, 20, 21, 23–27 and 34–44. [file 41586_2025_8843_MOESM5_ESM.zip › suppl_figure_3_to_14/suppl_figure_11_chr09/suppl_figure_11bh_36_D_PrW.pdf]

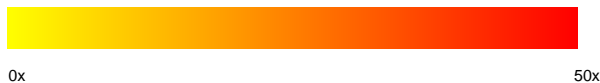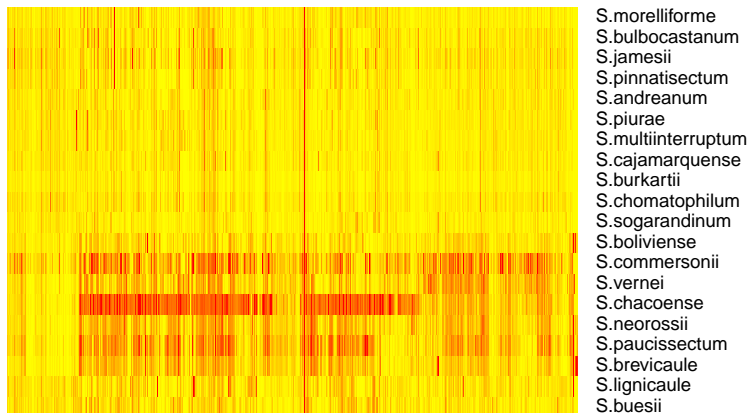

— TE — Gene — Non-aln • TanRep ♦ CenRep ■ rDNA

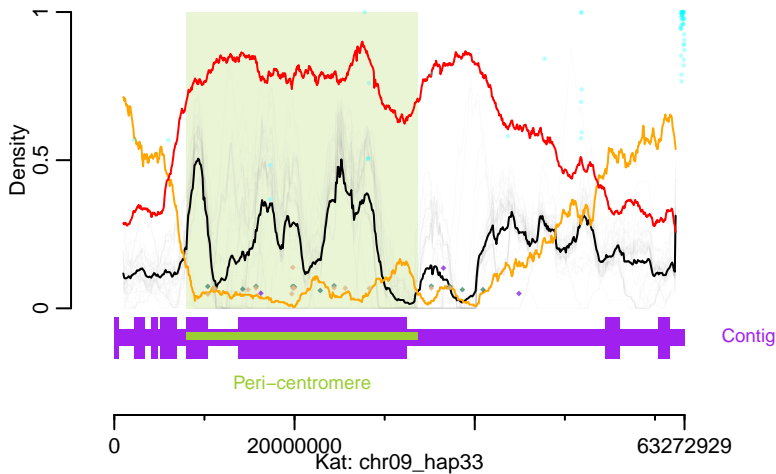

Supplement: Supplementary file 5 — Supplementary Figs. 3–14, 20, 21, 23–27 and 34–44. [file 41586_2025_8843_MOESM5_ESM.zip › suppl_figure_3_to_14/suppl_figure_11_chr09/suppl_figure_11ah_33_H_Kat.pdf]

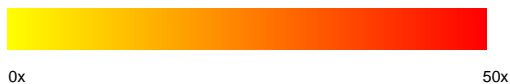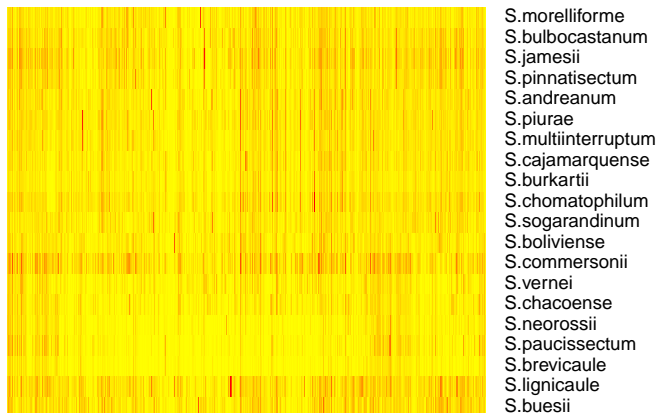

— TE — Gene — Non-aln ● TanRep ♦ CenRep ■ rDNA

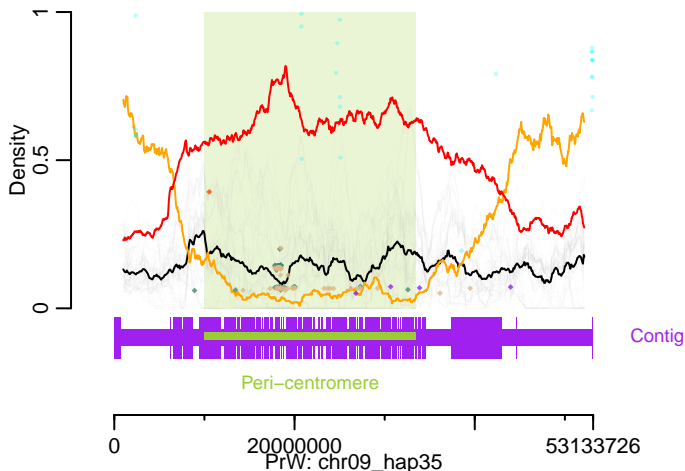

Supplement: Supplementary file 5 — Supplementary Figs. 3–14, 20, 21, 23–27 and 34–44. [file 41586_2025_8843_MOESM5_ESM.zip › suppl_figure_3_to_14/suppl_figure_11_chr09/suppl_figure_11ax_35_D_PrW.pdf]

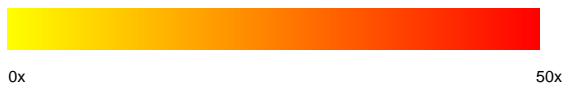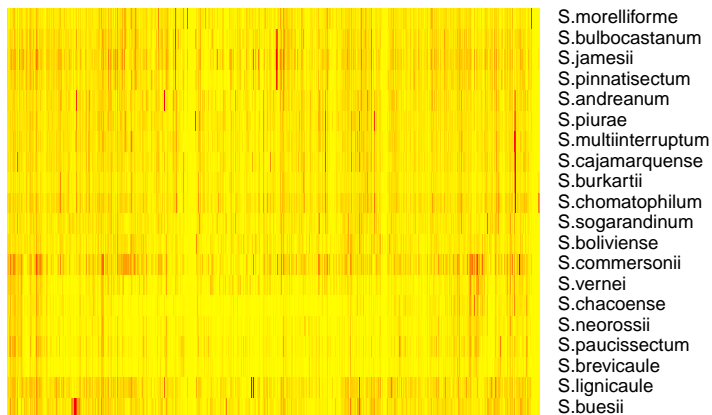

— TE — Gene — Non-aln • TanRep ♦ CenRep ■ rDNA

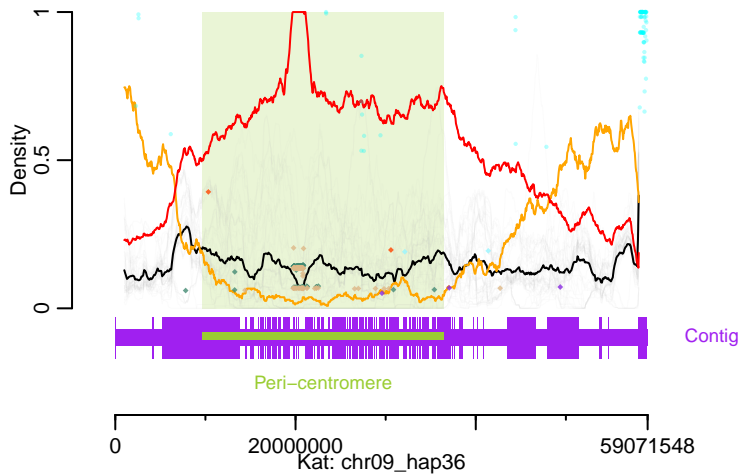

Supplement: Supplementary file 5 — Supplementary Figs. 3–14, 20, 21, 23–27 and 34–44. [file 41586_2025_8843_MOESM5_ESM.zip › suppl_figure_3_to_14/suppl_figure_11_chr09/suppl_figure_11bl_36_H_Kat.pdf]

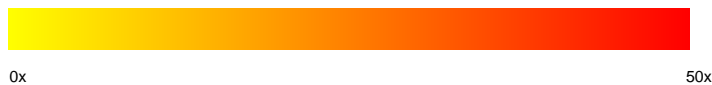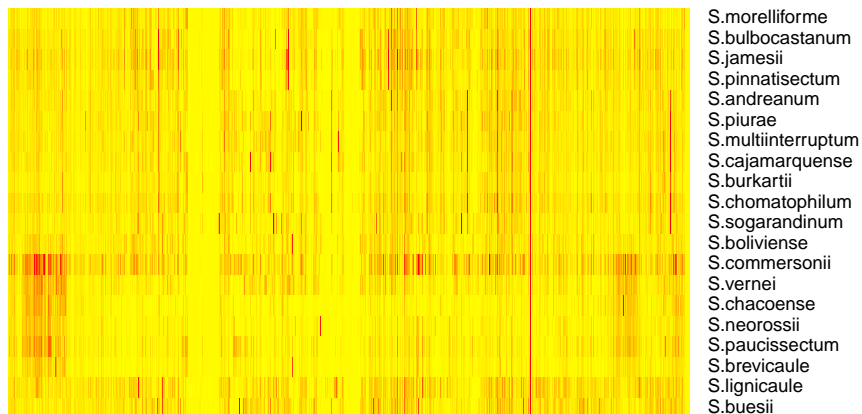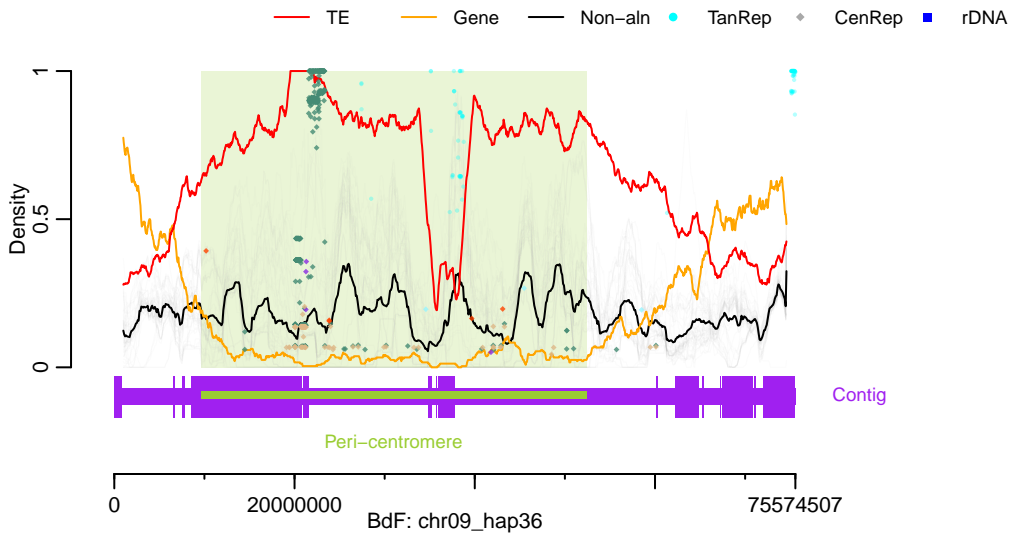

Supplement: Supplementary file 5 — Supplementary Figs. 3–14, 20, 21, 23–27 and 34–44. [file 41586_2025_8843_MOESM5_ESM.zip › suppl_figure_3_to_14/suppl_figure_11_chr09/suppl_figure_11bf_36_B_BdF.pdf]

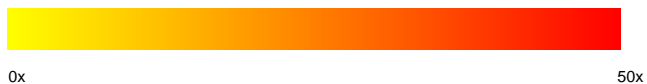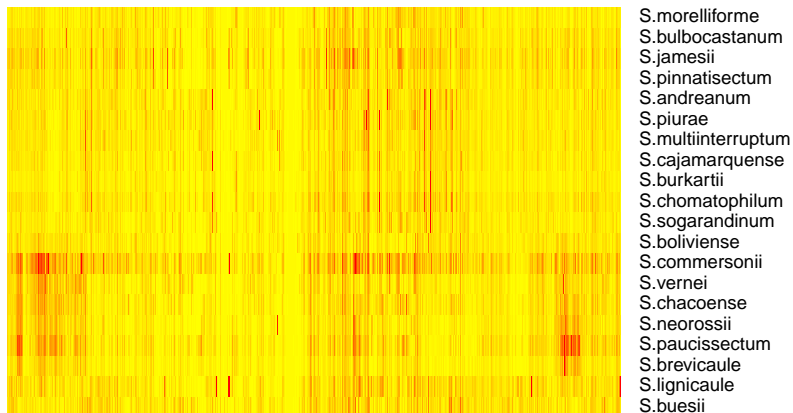

— TE — Gene — Non-aln • TanRep ♦ CenRep ■ rDNA

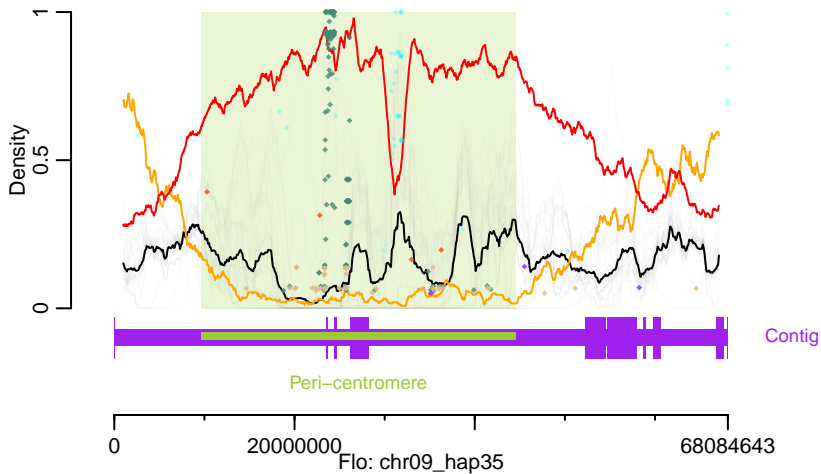

Supplement: Supplementary file 5 — Supplementary Figs. 3–14, 20, 21, 23–27 and 34–44. [file 41586_2025_8843_MOESM5_ESM.zip › suppl_figure_3_to_14/suppl_figure_11_chr09/suppl_figure_11ay_35_E_Flo.pdf]

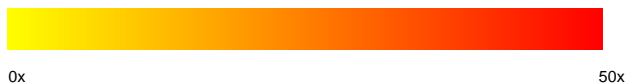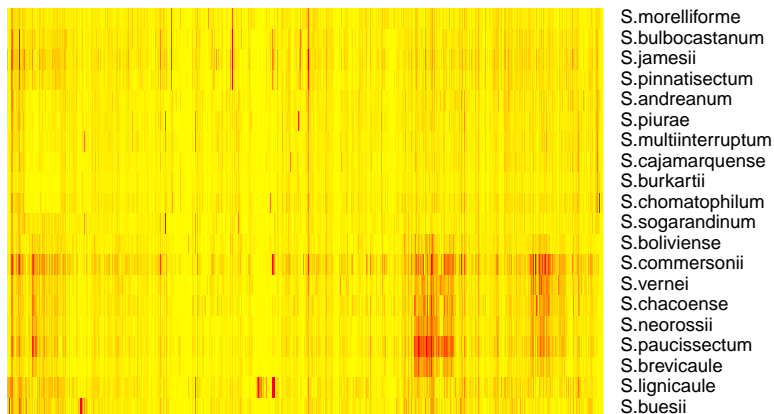

— TE — Gene — Non-aln • TanRep ♦ CenRep ■ rDNA

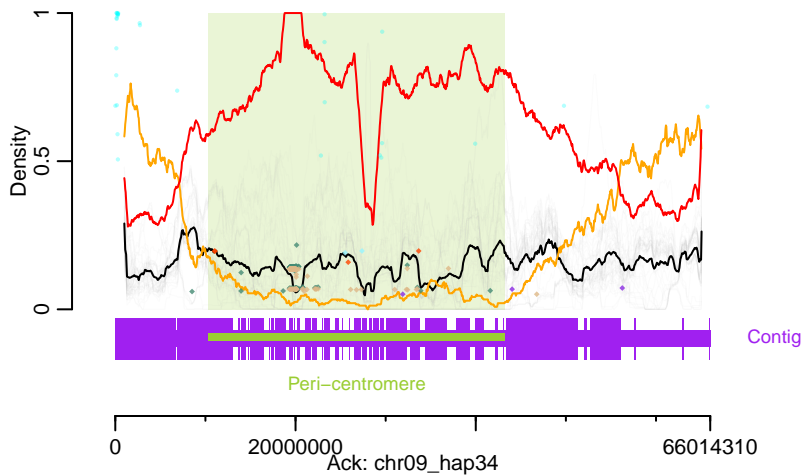

Supplement: Supplementary file 5 — Supplementary Figs. 3–14, 20, 21, 23–27 and 34–44. [file 41586_2025_8843_MOESM5_ESM.zip › suppl_figure_3_to_14/suppl_figure_11_chr09/suppl_figure_11ap_34_F_Ack.pdf]

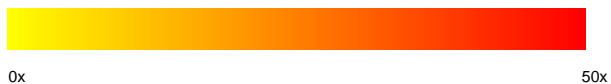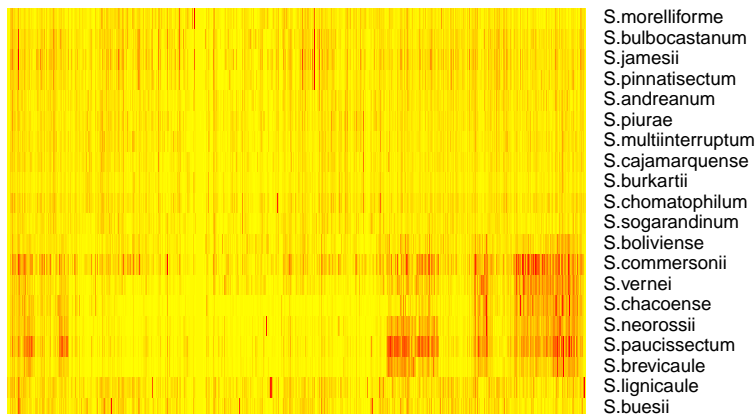

— TE — Gene — Non-aln • TanRep ♦ CenRep ■ rDNA

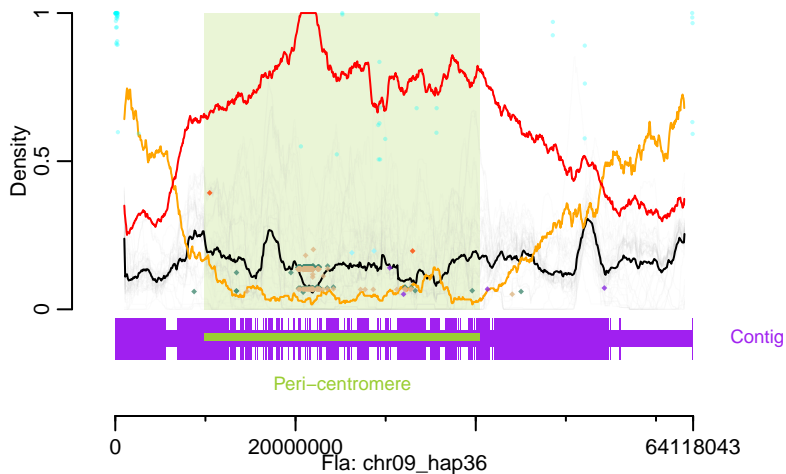

Supplement: Supplementary file 5 — Supplementary Figs. 3–14, 20, 21, 23–27 and 34–44. [file 41586_2025_8843_MOESM5_ESM.zip › suppl_figure_3_to_14/suppl_figure_11_chr09/suppl_figure_11bk_36_G_Fla.pdf]

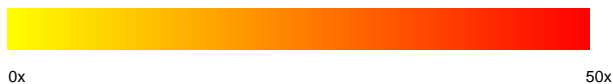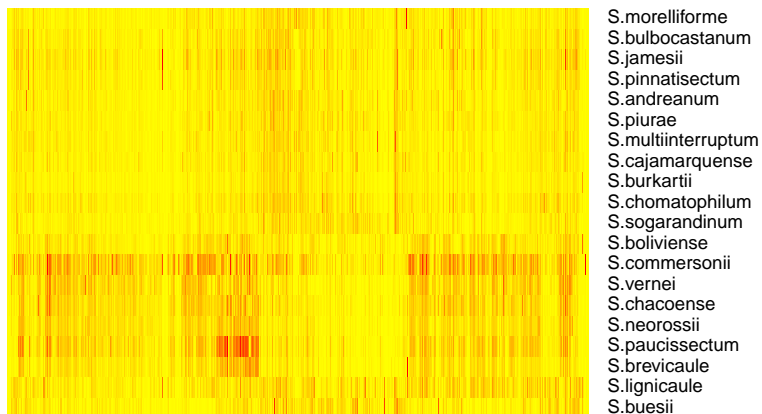

— TE — Gene — Non-aln • TanRep ♦ CenRep ■ rDNA

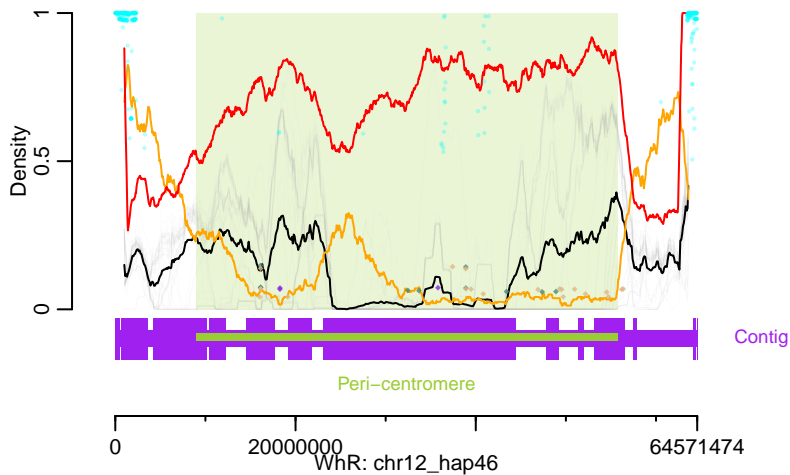

Supplement: Supplementary file 5 — Supplementary Figs. 3–14, 20, 21, 23–27 and 34–44. [file 41586_2025_8843_MOESM5_ESM.zip › suppl_figure_3_to_14/suppl_figure_14_chr12/suppl_figure_14ak_46_A_WhR.pdf]

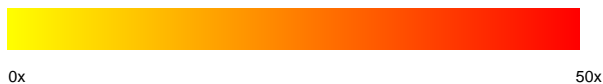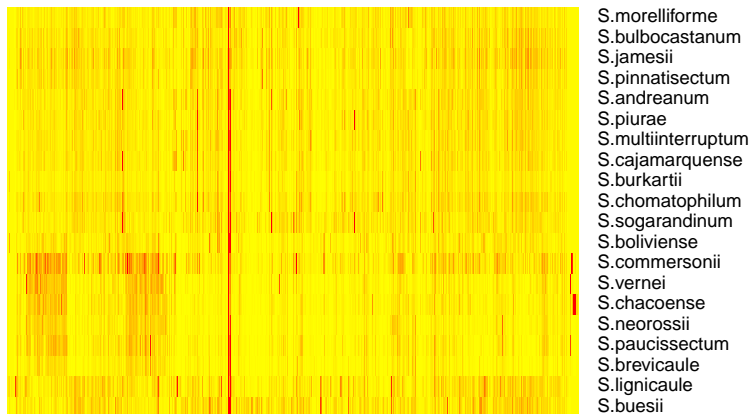

TE Gene Non-aln TanRep CenRep rDNA

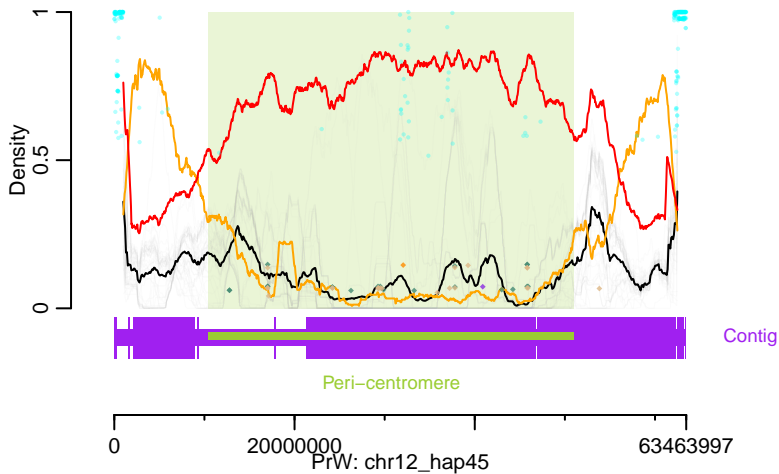

Supplement: Supplementary file 5 — Supplementary Figs. 3–14, 20, 21, 23–27 and 34–44. [file 41586_2025_8843_MOESM5_ESM.zip › suppl_figure_3_to_14/suppl_figure_14_chr12/suppl_figure_14ad_45_D_PrW.pdf]

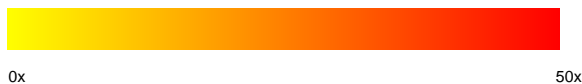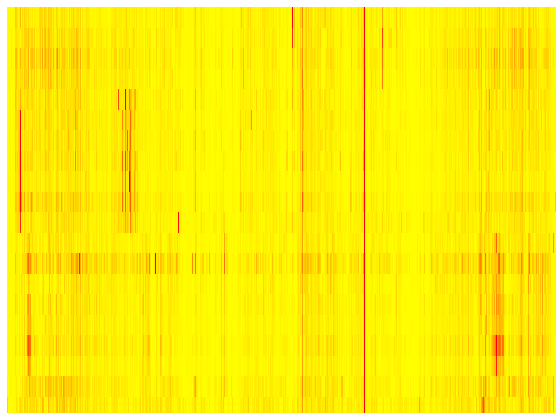

— TE — Gene — Non-aln • TanRep ♦ CenRep ■ rDNA

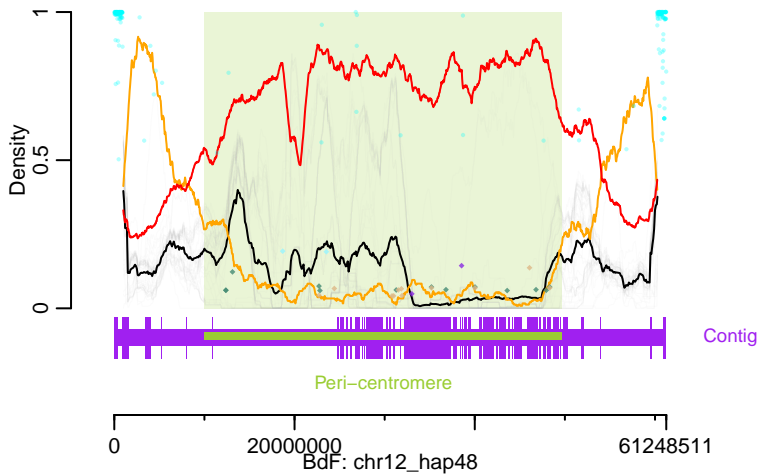

Supplement: Supplementary file 5 — Supplementary Figs. 3–14, 20, 21, 23–27 and 34–44. [file 41586_2025_8843_MOESM5_ESM.zip › suppl_figure_3_to_14/suppl_figure_14_chr12/suppl_figure_14bf_48_B_BdF.pdf]

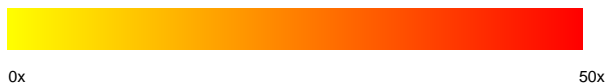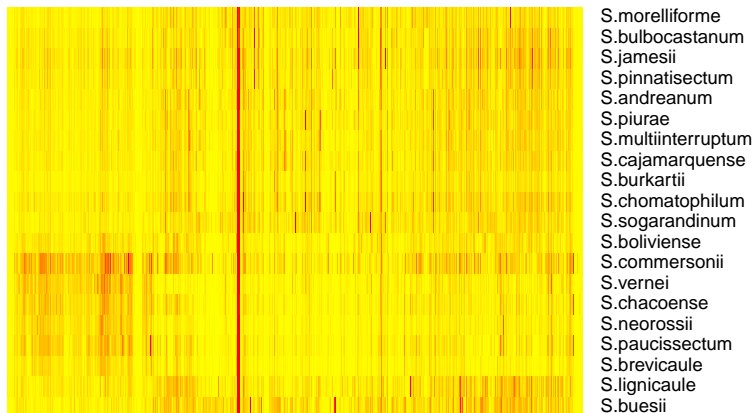

— TE — Gene — Non-aln • TanRep ♦ CenRep ■ rDNA

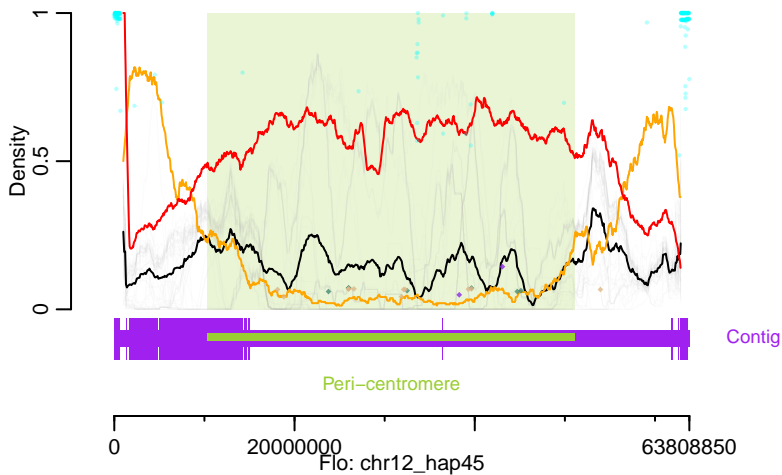

Supplement: Supplementary file 5 — Supplementary Figs. 3–14, 20, 21, 23–27 and 34–44. [file 41586_2025_8843_MOESM5_ESM.zip › suppl_figure_3_to_14/suppl_figure_14_chr12/suppl_figure_14ae_45_E_Flo.pdf]

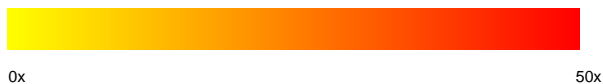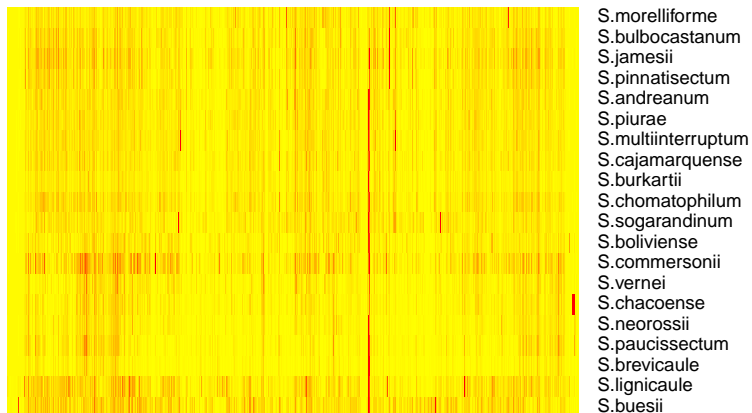

— TE — Gene — Non-aln • TanRep ♦ CenRep ■ rDNA

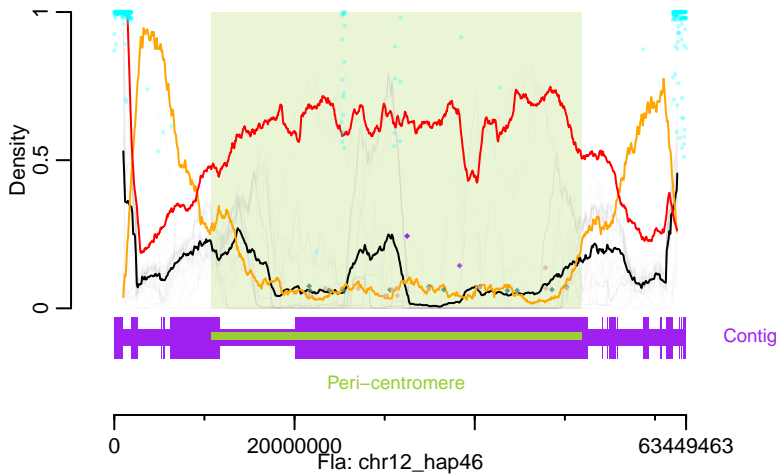

Supplement: Supplementary file 5 — Supplementary Figs. 3–14, 20, 21, 23–27 and 34–44. [file 41586_2025_8843_MOESM5_ESM.zip › suppl_figure_3_to_14/suppl_figure_14_chr12/suppl_figure_14aq_46_G_Fla.pdf]

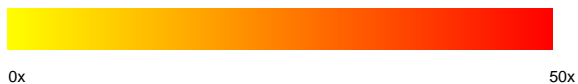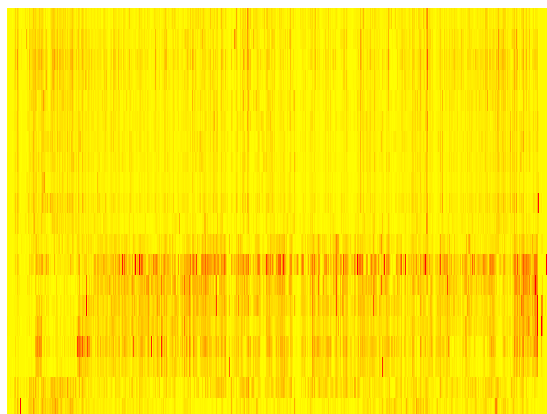

— TE — Gene — Non-aln • TanRep ♦ CenRep ■ rDNA

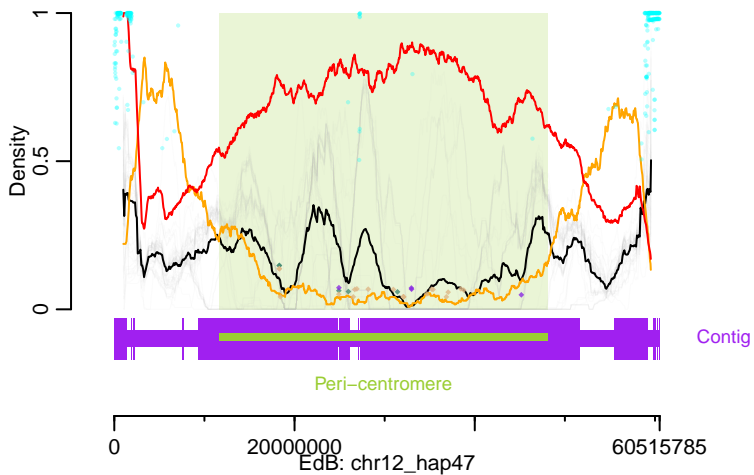

Supplement: Supplementary file 5 — Supplementary Figs. 3–14, 20, 21, 23–27 and 34–44. [file 41586_2025_8843_MOESM5_ESM.zip › suppl_figure_3_to_14/suppl_figure_14_chr12/suppl_figure_14bd_47_J_EdB.pdf]

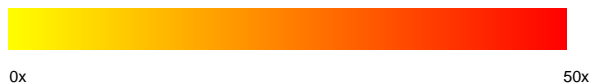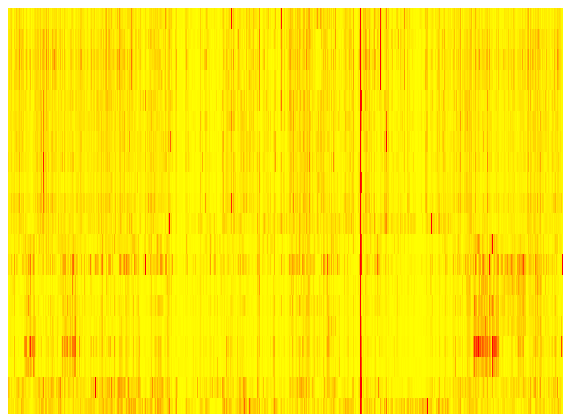

— TE — Gene — Non-aln • TanRep ♦ CenRep ■ rDNA

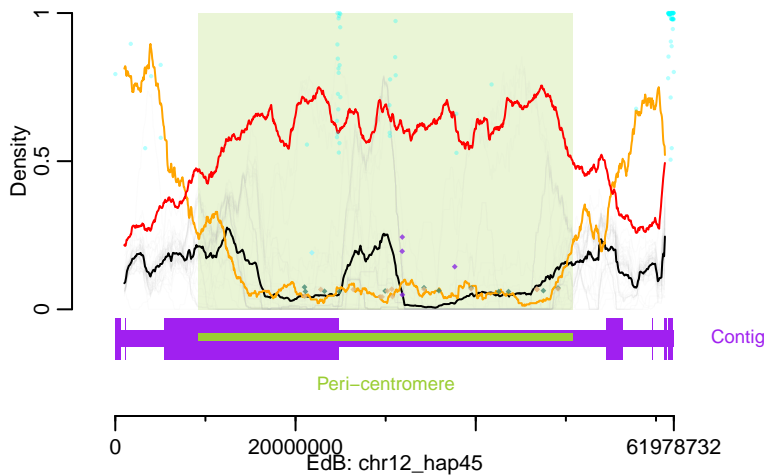

Supplement: Supplementary file 5 — Supplementary Figs. 3–14, 20, 21, 23–27 and 34–44. [file 41586_2025_8843_MOESM5_ESM.zip › suppl_figure_3_to_14/suppl_figure_14_chr12/suppl_figure_14aj_45_J_EdB.pdf]

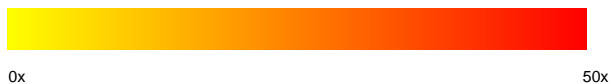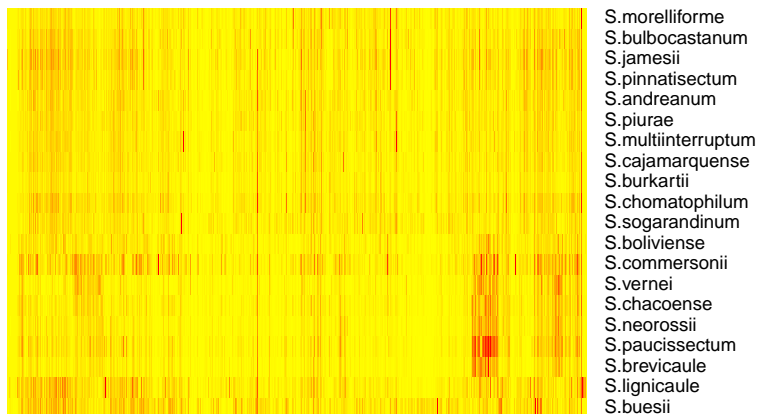

— TE — Gene — Non-aln • TanRep ♦ CenRep ■ rDNA

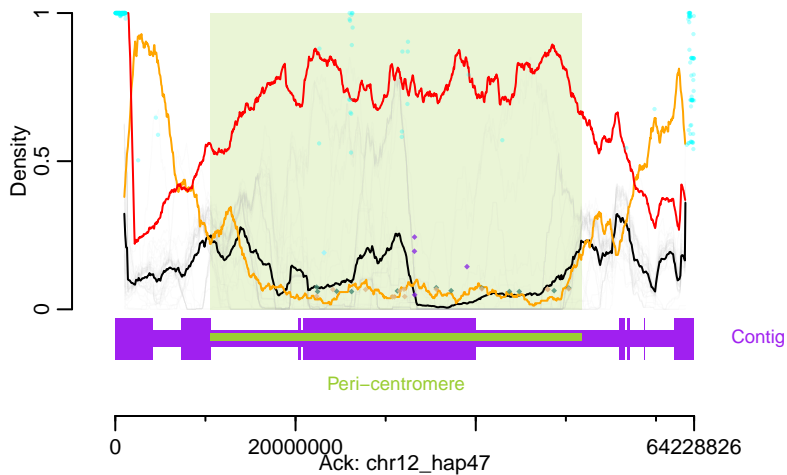

Supplement: Supplementary file 5 — Supplementary Figs. 3–14, 20, 21, 23–27 and 34–44. [file 41586_2025_8843_MOESM5_ESM.zip › suppl_figure_3_to_14/suppl_figure_14_chr12/suppl_figure_14az_47_F_Ack.pdf]

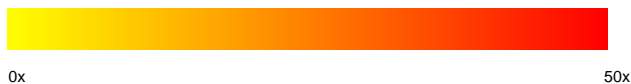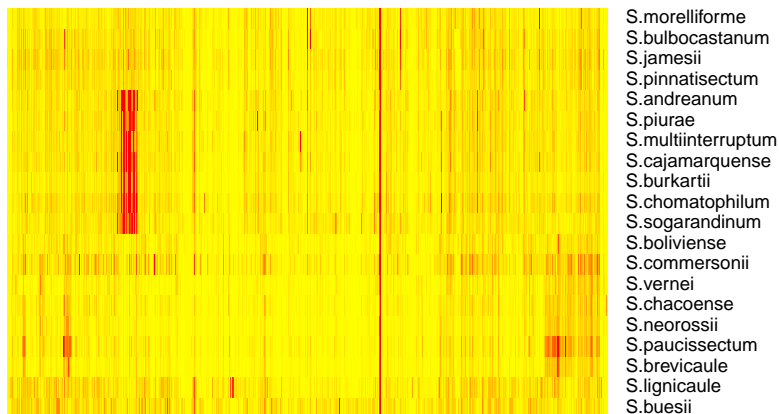

— TE — Gene — Non-aln • TanRep ♦ CenRep ■ rDNA

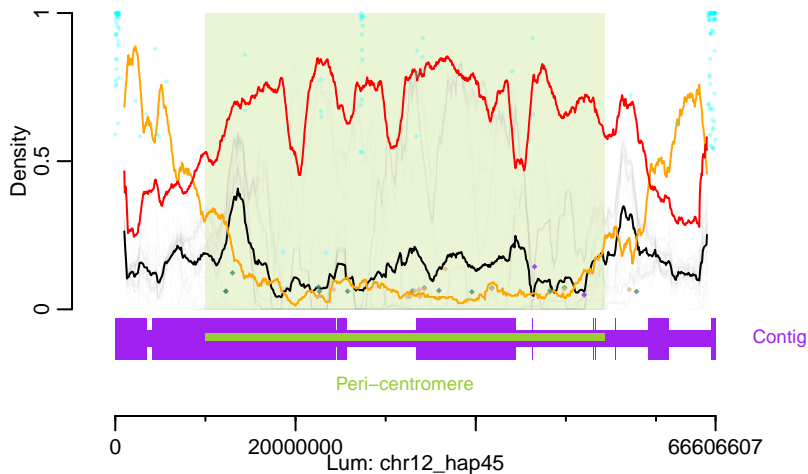

Supplement: Supplementary file 5 — Supplementary Figs. 3–14, 20, 21, 23–27 and 34–44. [file 41586_2025_8843_MOESM5_ESM.zip › suppl_figure_3_to_14/suppl_figure_14_chr12/suppl_figure_14ai_45_I_Lum.pdf]

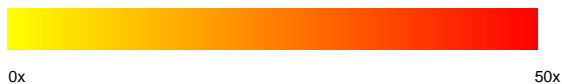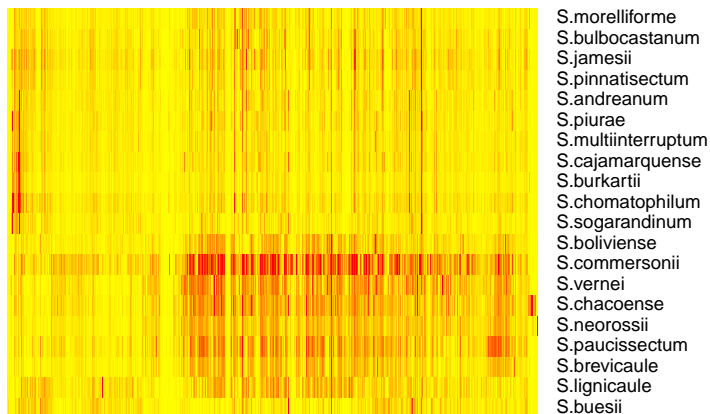

— TE — Gene — Non-aln • TanRep ♦ CenRep ■ rDNA

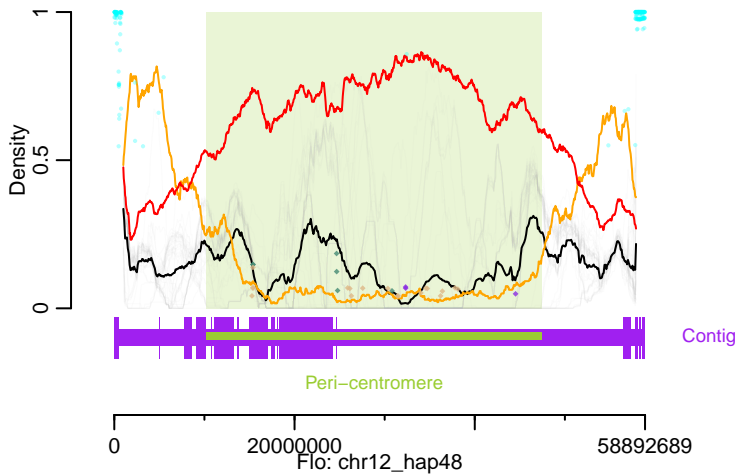

Supplement: Supplementary file 5 — Supplementary Figs. 3–14, 20, 21, 23–27 and 34–44. [file 41586_2025_8843_MOESM5_ESM.zip › suppl_figure_3_to_14/suppl_figure_14_chr12/suppl_figure_14bi_48_E_Flo.pdf]

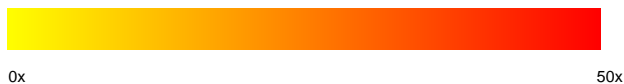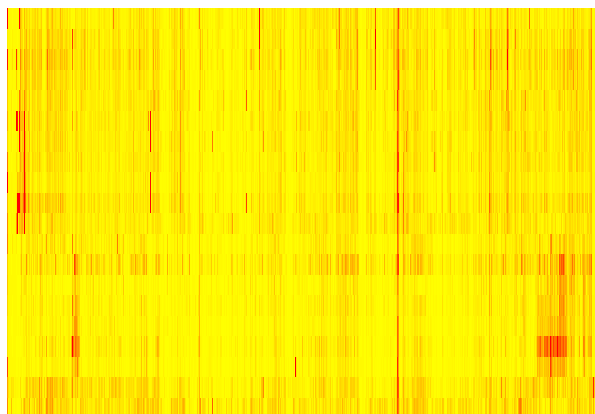

— TE — Gene — Non-aln • TanRep ♦ CenRep ■ rDNA

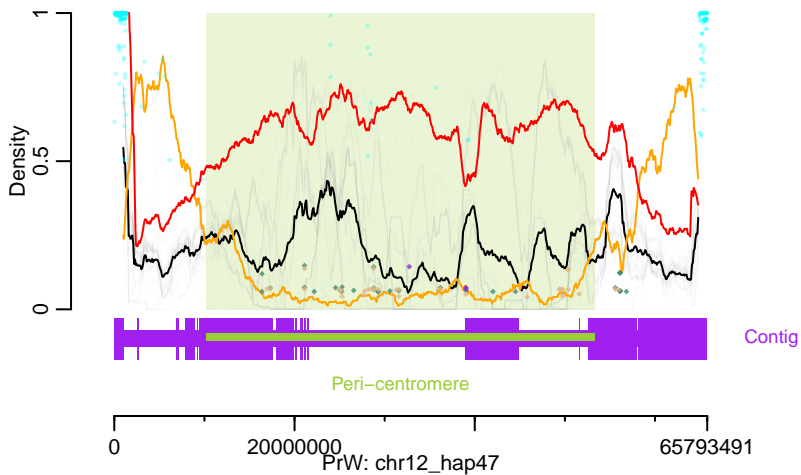

Supplement: Supplementary file 5 — Supplementary Figs. 3–14, 20, 21, 23–27 and 34–44. [file 41586_2025_8843_MOESM5_ESM.zip › suppl_figure_3_to_14/suppl_figure_14_chr12/suppl_figure_14ax_47_D_PrW.pdf]

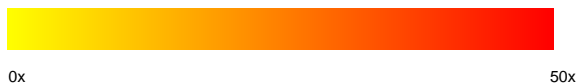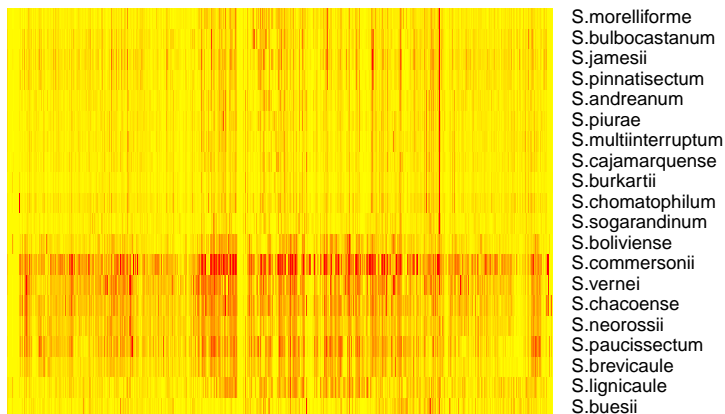

— TE — Gene — Non-aln • TanRep ♦ CenRep ■ rDNA

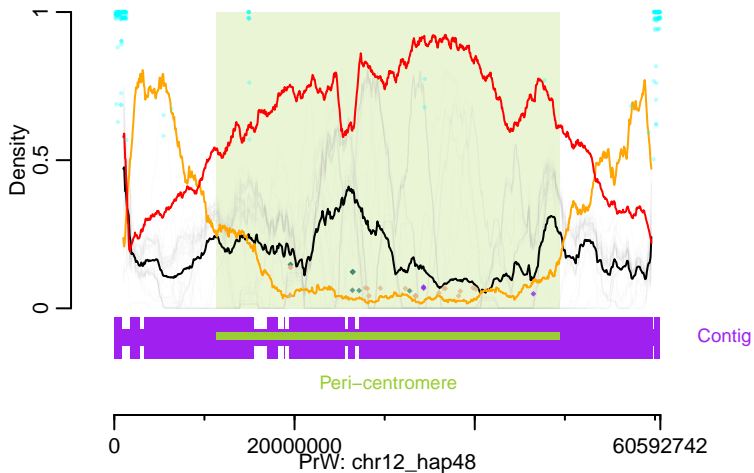

Supplement: Supplementary file 5 — Supplementary Figs. 3–14, 20, 21, 23–27 and 34–44. [file 41586_2025_8843_MOESM5_ESM.zip › suppl_figure_3_to_14/suppl_figure_14_chr12/suppl_figure_14bh_48_D_PrW.pdf]

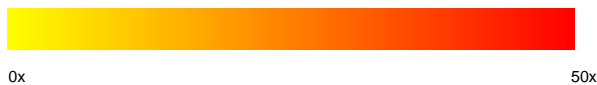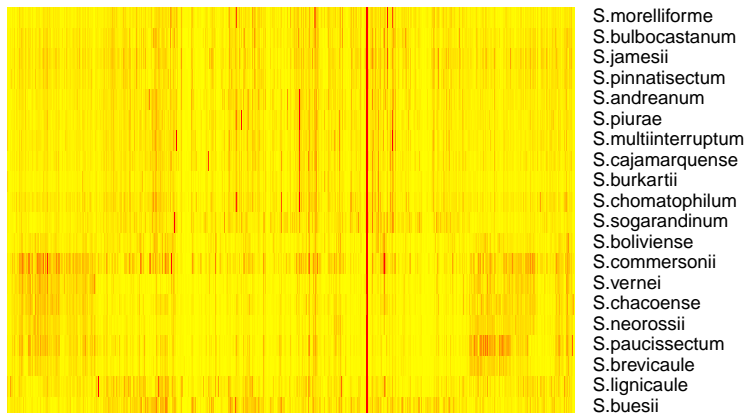

— TE — Gene — Non-aln • TanRep ♦ CenRep ■ rDNA

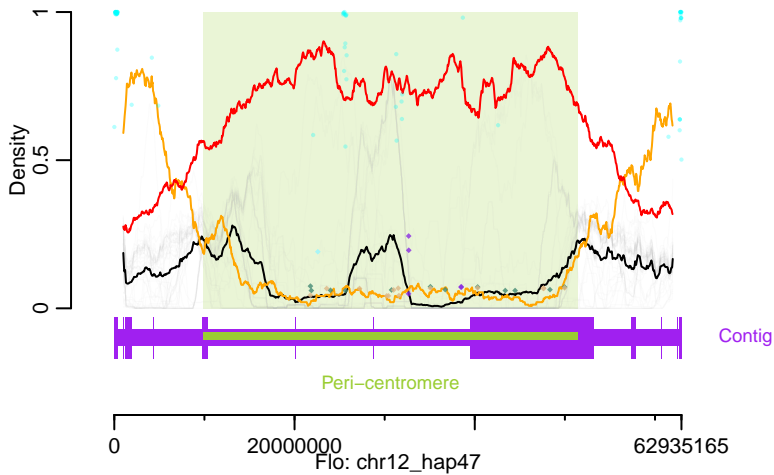

Supplement: Supplementary file 5 — Supplementary Figs. 3–14, 20, 21, 23–27 and 34–44. [file 41586_2025_8843_MOESM5_ESM.zip › suppl_figure_3_to_14/suppl_figure_14_chr12/suppl_figure_14ay_47_E_Flo.pdf]

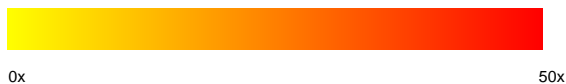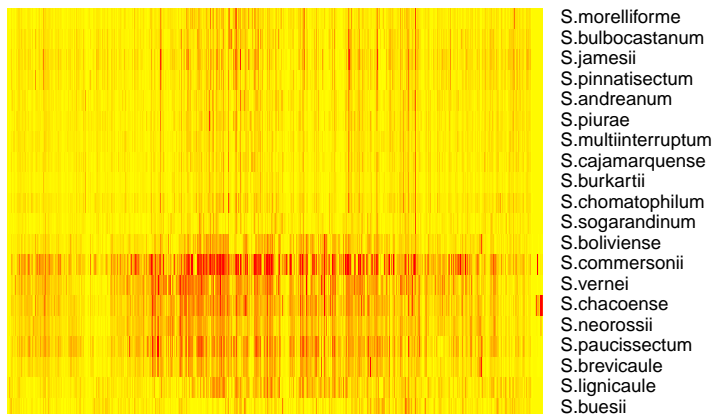

— TE — Gene — Non-aln • TanRep ♦ CenRep ■ rDNA

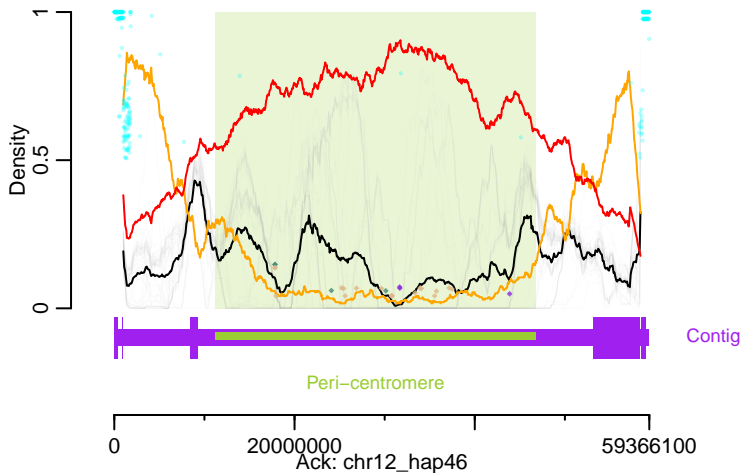

Supplement: Supplementary file 5 — Supplementary Figs. 3–14, 20, 21, 23–27 and 34–44. [file 41586_2025_8843_MOESM5_ESM.zip › suppl_figure_3_to_14/suppl_figure_14_chr12/suppl_figure_14ap_46_F_Ack.pdf]

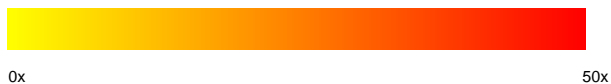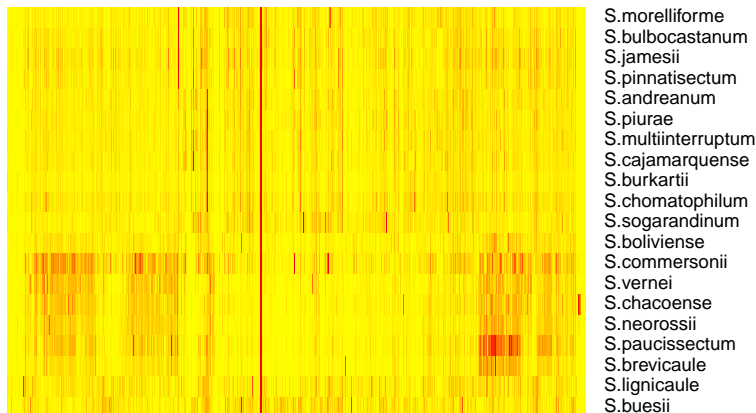

— TE — Gene — Non-aln • TanRep ♦ CenRep ■ rDNA

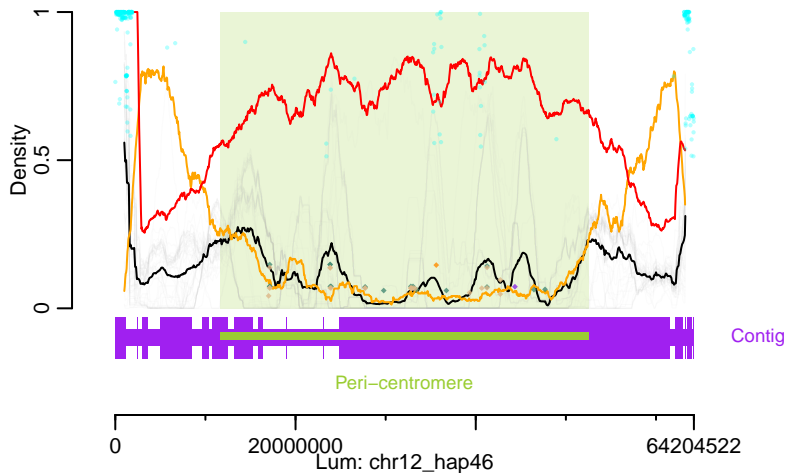

Supplement: Supplementary file 5 — Supplementary Figs. 3–14, 20, 21, 23–27 and 34–44. [file 41586_2025_8843_MOESM5_ESM.zip › suppl_figure_3_to_14/suppl_figure_14_chr12/suppl_figure_14as_46_I_Lum.pdf]

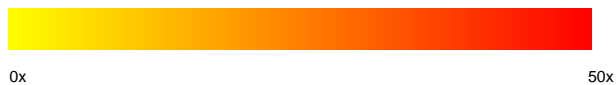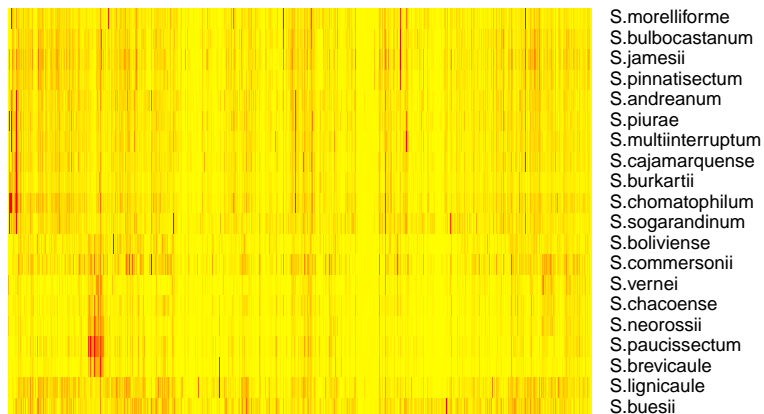

— TE — Gene — Non-aln • TanRep ♦ CenRep ■ rDNA

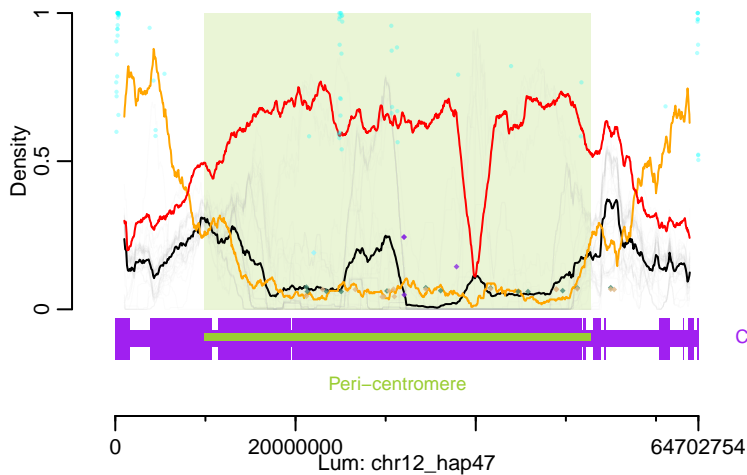

Supplement: Supplementary file 5 — Supplementary Figs. 3–14, 20, 21, 23–27 and 34–44. [file 41586_2025_8843_MOESM5_ESM.zip › suppl_figure_3_to_14/suppl_figure_14_chr12/suppl_figure_14bc_47_I_Lum.pdf]

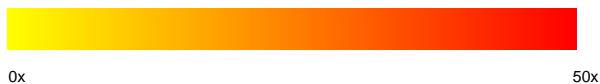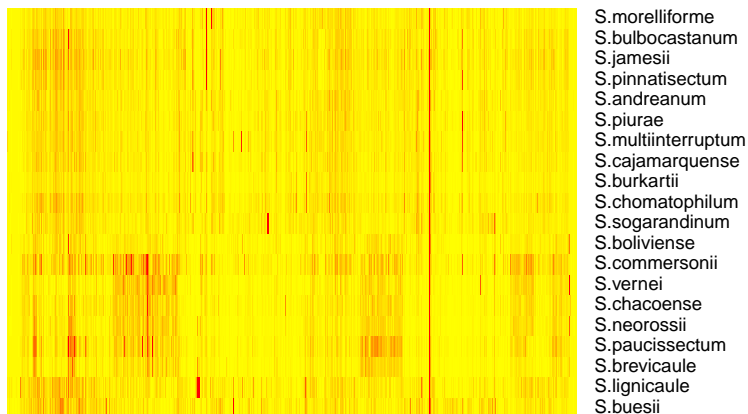

— TE — Gene — Non-aln • TanRep ♦ CenRep ■ rDNA

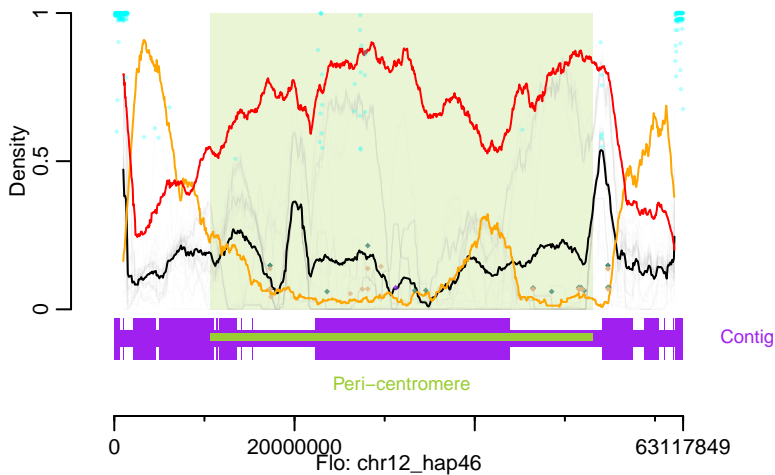

Supplement: Supplementary file 5 — Supplementary Figs. 3–14, 20, 21, 23–27 and 34–44. [file 41586_2025_8843_MOESM5_ESM.zip › suppl_figure_3_to_14/suppl_figure_14_chr12/suppl_figure_14ao_46_E_Flo.pdf]
